# Supplementary material for: Natural selection drives the evolution of mitogenomes in Acrossocheilus
Source: PLoS One. 2022 Oct 13;17(10):e0276056. doi: 10.1371/journal.pone.0276056 (PMC9560497; doi:10.1371/journal.pone.0276056)
Supplement: S1 Table — (PDF) [file pone.0276056.s001.pdf]

**S1 Table. Details of site models used in CodeML.**

| Model | np | Ln L | Estimates of parameters | | | | Model compared | LRT P-value |
|---|---|---|---|---|---|---|---|---|
| | | | p: | 0.94554 | 0.05446 | 0.00000 | | |
| M2a | 53 | -50190.789940 | ω: | 0.03049 | 1.00000 | 46.01009 | | |
| | | | p: | 0.94554 | 0.05446 | | | |
| M1a | 51 | -50190.789782 | ω: | 0.03049 | 1.00000 | | M1a vs. M2a | 0.999842012 |
| | | | p0=0.97848 | p=0.01149 | q=0.06501 | | | |
| M8 | 53 | -50028.911973 | (p1= 0.02152) | ω= 1.00000 | | | | |
| M7 | 51 | -50078.073078 | p=0.00698 | | q=0.03699 | | M7 vs.M8 | 0.000000000 |
| | | | p0=0.99408 | p=0.13178 | q=2.07789 | | | |
| M8a | 52 | -49997.390828 | (p1= 0.00592) | ω= 1.00000 | | | M8a vs.M8 | 0.000000000 |
