# Supplementary material for: Natural selection drives the evolution of mitogenomes in Acrossocheilus
Source: PLoS One. 2022 Oct 13;17(10):e0276056. doi: 10.1371/journal.pone.0276056 (PMC9560497; doi:10.1371/journal.pone.0276056)
Supplement: S2 Table — (PDF) [file pone.0276056.s002.pdf]

S2 Table. Standard bioclimatic variables for the *Acrossocheilus* individuals in fourteen localities.

	S1	S2	S3	S4	S5	S6	S7	S8	S9	S10	S11	S12	S13	S14
Bio1	24.1	20.9	18.5	19.8	16.7	18.5	18.5	20.8	19.4	18.6	15.7	17.7	16.6	18.0
Bio2	8.3	8.6	8.5	8.0	8.4	8.5	8.5	8.6	9.4	7.0	7.9	8.5	7.3	7.1
Bio3	45.8	30.7	30.5	30.1	28.6	30.5	30.5	34.5	34.1	25.2	24.8	29.1	27.9	26.6
Bio4	364.0	713.9	723.5	678.8	778.3	723.5	723.5	599.9	646.0	763.2	876.1	759.3	697.8	710.9
Bio5	32.1	34.5	31.9	33.3	31.2	31.9	31.9	32.1	32.6	32.6	31.4	31.9	29.8	32.0
Bio6	14.0	6.4	4.1	6.6	1.9	4.1	4.1	7.2	5.2	4.9	-0.3	2.6	3.5	5.3
Bio7	18.1	28.1	27.8	26.7	29.3	27.8	27.8	24.9	27.4	27.7	31.7	29.3	26.3	26.7
Bio8	27.2	24.4	25.3	22.6	20.8	25.3	25.3	24.0	22.8	27.7	19.8	21.2	24.2	26.5
Bio9	19.1	13.6	9.0	13.3	6.6	9.0	9.0	14.6	12.5	11.7	6.6	14.0	7.5	8.8
Bio10	27.9	29.1	26.8	27.8	25.9	26.8	26.8	27.4	26.8	27.9	26.2	26.5	24.9	26.5
Bio11	19.1	11.8	9.0	11.3	6.6	9.0	9.0	12.9	11.0	9.3	4.6	8.0	7.5	8.8
Bio12	1513.0	1524.0	1336.0	1517.0	1227.0	1336.0	1336.0	2044.0	1705.0	1652.0	1695.0	1919.0	1591.0	1076.0
Bio13	282.0	259.0	232.0	249.0	209.0	232.0	232.0	389.0	295.0	249.0	294.0	342.0	411.0	180.0
Bio14	15.0	38.0	34.0	38.0	33.0	34.0	34.0	33.0	40.0	41.0	47.0	52.0	15.0	22.0
Bio15	80.6	64.0	62.9	55.0	56.6	62.9	62.9	71.6	63.1	54.6	56.1	64.6	102.2	68.5
Bio16	754.0	734.0	623.0	633.0	533.0	623.0	623.0	993.0	802.0	660.0	756.0	933.0	992.0	512.0
Bio17	56.0	128.0	124.0	133.0	120.0	124.0	124.0	128.0	145.0	147.0	169.0	181.0	57.0	72.0
Bio18	549.0	531.0	553.0	552.0	457.0	553.0	553.0	827.0	583.0	619.0	600.0	626.0	940.0	512.0
Bio19	56.0	186.0	124.0	181.0	120.0	124.0	124.0	184.0	203.0	173.0	213.0	256.0	57.0	72.0

Bio1, annual mean temperature; Bio2, mean diurnal range (mean of monthly (max temp – min temp)); Bio3, isothermality (Bio2/Bio7)(*100); Bio4, temperature seasonality (standard deviation *100); Bio5, max temperature of warmest month; Bio6, min temperature of coldest month; Bio7, temperature annual range (Bio5-Bio6); Bio8, mean temperature of wettest quarter; Bio9, mean temperature of driest quarter; Bio10, mean temperature of warmest quarter; Bio11, mean temperature of coldest quarter; Bio12, annual precipitation; Bio13, precipitation of wettest month; Bio14, precipitation of driest month; Bio15, precipitation seasonality (Coefficient of variation); Bio16, precipitation of wettest quarter; Bio17, precipitation of driest quarter; Bio18, precipitation of warmest quarter; Bio19, precipitation of coldest quarter.
