# Supplementary material for: Natural selection drives the evolution of mitogenomes in Acrossocheilus
Source: PLoS One. 2022 Oct 13;17(10):e0276056. doi: 10.1371/journal.pone.0276056 (PMC9560497; doi:10.1371/journal.pone.0276056)
Supplement: S3 Table — (PDF) [file pone.0276056.s003.pdf]

S3 Table. Significant changes in amino acid properties detected in TreeSAAP.

Gene	z-score	AA-score	P-value	Significant properties (P < 0.001)
ATP6	8.428	8	<0.001	Equilibrium constant (ionization of COOH)
COX1	13.006	8	<0.001	Equilibrium constant (ionization of COOH)
COX2	4.644	8	<0.001	Equilibrium constant (ionization of COOH)
	3.541	6	<0.001	Alpha-helical tendencies
COX3	6.737	8	<0.001	Equilibrium constant (ionization of COOH)
CYTB	12.308	8	<0.001	Equilibrium constant (ionization of COOH)
	-3.108	7	<0.001	Turn tendencies
ND1	10.009	8	<0.001	Equilibrium constant (ionization of COOH)
	3.704	7	<0.001	Solvent accessible reduction ratio
	-3.241	7	<0.001	Turn tendencies
ND2	12.536	8	<0.001	Equilibrium constant (ionization of COOH)
	-3.403	8	<0.001	Alpha-helical tendencies
	-3.454	8	<0.001	Hydropathy
	-3.627	8	<0.001	Coil tendencies
	-3.221	7	<0.001	Total non-bonded energy
	-3.268	7	<0.001	Chromatographic index
	-3.398	7	<0.001	Mean r.m.s. fluctuation displacement
	-3.448	7	<0.001	Buriedness
	-3.678	7	<0.001	Turn tendencies
	-3.748	7	<0.001	Polarity
	3.294	6	<0.001	Power to be at the C-terminal
	-3.185	6	<0.001	Chromatographic index
	-3.617	6	<0.001	Total non-bonded energy
	-3.978	6	<0.001	Isoelectirc point
ND3	7.607	7	<0.001	Solvent accessible reduction ratio
	4.885	7	<0.001	Thermodynamic transfer hydrophobicity
	6.591	6	<0.001	Equilibrium constant (ionization of COOH)
	5.812	6	<0.001	Buriedness
	5.145	6	<0.001	Surrounding hydrophobicity
	3.12	6	<0.001	Alpha-helical tendencies
ND4	13.47	8	<0.001	Equilibrium constant (ionization of COOH)
	-3.729	8	<0.001	Coil tendencies
	-3.275	7	<0.001	Buriedness
	-3.516	7	<0.001	Polarity
	-3.949	7	<0.001	Mean r.m.s. fluctuation displacement
	-3.988	7	<0.001	Turn tendencies
	-3.116	6	<0.001	Beta-structure tendencies
	-3.172	6	<0.001	Mean r.m.s. fluctuation displacement
	-3.212	6	<0.001	Solvent accessible reduction ratio
	-3.222	6	<0.001	Total non-bonded energy
	-3.511	6	<0.001	Chromatographic index
	-4.037	6	<0.001	Isoelectirc point

ND4L	4.468	8	<0.001	Equilibrium constant (ionization of COOH)
	3.454	8	<0.001	Isoelectirc point
ND5	10.983	8	<0.001	Equilibrium constant (ionization of COOH)
	-3.159	8	<0.001	Polarity
	-3.253	8	<0.001	Short and medium range non-banded energy
	-3.377	8	<0.001	Average number of surrounding residues
	-3.547	8	<0.001	Solvent accessible reduction ratio
	-3.831	8	<0.001	Turn tendencies
	-3.991	8	<0.001	Hydropathy
	-4.148	8	<0.001	Coil tendencies
	6.359	7	<0.001	Solvent accessible reduction ratio
	3.183	7	<0.001	Thermodynamic transfer hydrophobicity
	-3.433	7	<0.001	Total non-bonded energy
	-3.576	7	<0.001	Surrounding hydrophobicity
	-3.643	7	<0.001	Chromatographic index
	-3.785	7	<0.001	Mean r.m.s. fluctuation displacement
	-3.95	7	<0.001	Buriedness
	-4.204	7	<0.001	Polarity
ND6	5.927	8	<0.001	Equilibrium constant (ionization of COOH)
	-3.125	6	<0.001	Total non-bonded energy
