# Supplementary material for: Natural selection drives the evolution of mitogenomes in Acrossocheilus
Source: PLoS One. 2022 Oct 13;17(10):e0276056. doi: 10.1371/journal.pone.0276056 (PMC9560497; doi:10.1371/journal.pone.0276056)
Supplement: S1 Dataset — (DOCX) [file pone.0276056.s004.docx]

**ATP6**

>A._barbodon

ATGATAGTAAGTTTTTTCGACCAATTCGCAAGCCCATCCTACCTAGGAATCCCACTAATTGCCATCGCAATCGCCCTGCCCTGAATACTCTACCCGACCCCATCATCTCGATGAATTAACAACCGACTTATTACAGCCCAAGAATGATTTATTAACCGCTCCACAAACCAATTAATGATACCACTAAGCATAGAAGGACATAAATGAGCACTACTACTAACTTCGCTAATAATCTTCTTAATTACAATCAACATGCTAGGCCTACTACCCTACACCTTCACCCCCACAACACAACTATCACTCAACATGGGATTCGCTGTACCACTTTGACTTGCTACAGTGATCATCGGAATACGAAACCAACCAACAGTCGCACTAGGACACCTGCTCCCAGAAGGAACACCCATCCCACTGATTCCAGTACTAATTATCATCGAAACAATTAGTCTATTTATTCGACCACTAGCCCTAGGAGTTCGACTTACAGCCAACCTAACTGCAGGTCACCTACTAATCCAACTTATTGCCACCGCCGTATTTGTCCTTCTACCAATTATGCCAACAGTAGCAATCCTAACTGCCGCCGTACTCTTCCTACTCACACTACTAGAAGTTGCAGTTGCAATGATCCAAGCCTACGTATTTGTTCTTCTTCTAAGCCTATATCTACAAGAAAACACT

>A._beijiangensis_1

ATGATAGTAAGTTTTTTCGACCAATTTGCAAGCCCAGTTTACCTGGGGATCCCGCTAATTGCCATCGCAATTGCCCTTCCCTGAGTACTCTACCCAACCCCCTCATCTCGATGAATTAACAACCGACTTATTACAGCCCAAGAATGGTTTATTAACCGCTCCACAAACCAACTAATAACTCCACTGAATGCAGAAGGACATAAATGAGCACTTCTATTAACTTCATTAATAATCTTCCTGATTACAATCAACATACTGGGCCTACTACCTTACACATTCACACCCACAACACAACTATCACTCAACATGGGATTCGCCGTACCTTTATGACTTGCCACAGTTATTATTGGAATGCGAAACCAACCAACAGTCGCACTAGGACATCTACTCCCAGAAGGGACACCTATTCCACTGATTCCAGTACTGATCATCATCGAAACAATTAGCCTATTTATTCGACCACTAGCCCTAGGAGTTCGACTTACAGCCAACCTAACCGCAGGCCACCTACTAATCCAACTCATCGCCACCGCCGTATTTGTTCTTCTACCAATGATACCAGCAGTAGCAATTTTAACTGCCGCCGTACTTTTTCTACTTACATTGCTAGAAGTTGCAGTTGCAATAATCCAAGCCTATGTATTTGTACTTCTCTTAAGCCTATACCTTCAAGAAAACACT

>A._beijiangensis_2

ATGATAGTAAGTTTTTTCGACCAATTTGCAAGCCCAGTTTACCTGGGAATCCCACTAATTGCCATCGCAATTGCCCTTCCCTGAGCACTCTACCCAACCCCCACATCTCGATGAATTAACAACCGACTTACTACAGCCCAAGAATGGTTTATTAACCGCTCCACAAACCAACTAATAATACCACTGAGTGCAGAAGGACATAAATGAGCACTCCTATTAACTTCATTAATAATTTTCCTGATTACAATCAACATACTAGGCCTACTACCTTACACATTCACACCCACAACACAACTATCACTCAACATAGGGTTCGCCGTACCCTTATGACTTGCCACAGTTATTATTGGGATGCGAAACCAACCAACAGTCGCACTAGGACATCTACTCCCAGAAGGGACACCCATTCCACTGATCCCAGTATTAATTATCATCGAAACAATTAGCCTATTTATTCGACCACTAGCCCTAGGAGTCCGACTTACAGCCAACTTAACCGCAGGTCACCTACTAATCCAACTCATCGCCACCGCCGTATTTGTTCTTCTACCAATAATACCAACAGTAGCAATCTTAACCGCCGCCGTACTTTTTCTACTCACATTGCTAGAAGTTGCAGTTGCAATAATCCAAGCCTATGTATTTGTACTTCTCTTAAGCCTATACCTTCAAGAAAACACT

>A._fasciatus

ATGATAGTAAGTTTTTTCGACCAATTTGAAAGCCCAATCTACCTAGGAATCCCATTAATTGCCATCGCGATTGCCCTTCCCTGAGTACTCTACCCAACCCCCACATCTCGATGAATTAACAACCGACTTATTACAGCTCAAGAATGGTTTATTAACCGCTCCACAAATCAACTAATAACACCACTAAGCGTAGAAGGACATAAATGAGCACTCCTATTAACCTCACTAATAATTTTCCTAATTACAATCAACATGCTAGGCCTCCTACCCTACACCTTTACACCCACAACACAACTATCACTTAATATAGGATTCGCCGTGCCCTTATGACTTGCTACAGTTATCATTGGAATGCGAAACCAACCAACAGTCGCACTAGGACACCTACTCCCAGAAGGAACACCCATCCCACTGATTCCAGTACTAATTATCATTGAAACAATTAGCCTATTTATTCGACCATTAGCCCTTGGAGTCCGACTTACAGCCAACCTAACTGCAGGCCACCTACTAATTCAACTCATCGCCACTGCCGTATTTGTCCTCCTACCAATAATGCCAACAGTAGCAGTCCTAACTGCCGCCGTGCTTTTTCTTCTAACACTACTAGAAGTTGCAGTTGCAATAATCCAAGCCTATGTGTTTGTCCTTCTTCTAAGTCTATATCTTCAAGAAAACACT

>A._hemispinus

ATGATAGCAAGTTTTTTCGACCAATTTGAAAGCCCTATTTACCTAGGAGTCCCACTAATCGCCATCGCAATTGCTCTTCCCTGAGTACTTTACCCAACCCCTACATCTCGATGAATTAACAACCGACTTATCACAGCCCAAGAATGATTTATTAATCGCTCCACAAATCAACTAATAACACCACTAAGCGTAGAAGGACATAAATGAGCACTCCTATTAACTTCACTAATAATTTTCCTGATTACAATCAATATGCTAGGCCTTCTACCCTACACCTTCACACCTACAACACAACTTTCACTTAATATAGGATTCGCCGTACCCCTGTGACTTGCTACAGTTATCATTGGAATGCGAAACCAACCAACAATCGCTCTAGGACATCTACTCCCAGAAGGGACACCTATCCCACTGATTCCAGTACTAATTATTATTGAAACAATCAGCCTATTTATTCGACCGCTAGCCCTTGGAGTTCGACTTACAGCCAACCTGACCGCAGGTCACCTACTAATTCAACTTATCGCCACCGCCGTATTTGTCCTCCTACCAATAATACCAACAGTGGCAGTCCTGACTGCCGCCGTACTTTTCCTTCTTACACTATTGGAAGTTGCAGTTGCAATGATCCAAGCCTATGTGTTCGTACTTCTCCTAAGCCTATACCTTCAAGAAAACACT

>A._iridescens

ATGATAGTAAGTTTTTTCGACCAATTCGCAAGCCCATCCTACCTAGGAATCCCACTAATTGCCATCGCAATCGCCCTGCCCTGAATACTCTACCCGACCCCATCATCTCGATGAATTAACAACCGACTTATTACAGCCCAAGAATGATTTATTAACCGCTCCACAAACCAATTAATGATACCACTGAGCATAGAAGGACATAAATGAGCACTACTACTAACTTCGCTAATAATCTTCTTAATCACAATCAACATGCTAGGCCTACTACCCTACACCTTCACCCCCACAACACAACTGTCACTCAACATGGGATTCGCTGTACCACTTTGACTTGCTACAGTGATTATTGGAATACGAAACCAACCAACAGTCGCACTAGGACACCTGCTCCCAGAAGGAACACCCATCCCACTGATTCCAGTACTAATTATCATCGAAACAATTAGCCTATTTATTCGACCACTAGCCCTAGGAGTTCGACTTACAGCCAACCTAACTGCAGGTCACCTACTAATCCAACTTATTGCCACCGCCGTATTTGTCCTTCTACCAATAATACCAACAGTAGCAATCCTAACTGCCGCCGTACTTTTCCTACTCACACTACTAGAAGTTGCAGTTGCAATGATCCAAGCCTACGTATTTGTACTTCTTCTAAGCCTATATCTACAAGAAAACACT

>A._jishouensis

ATGATAGTAAGTTTTTTCGACCAATTTGAAAGCCCTATTTACCTAGGGATCCCACTAATTGCCATCGCAATTGCTCTTCCCTGAGTACTTTACCCAACCCCCACATCTCGATGAATCAACAACCGACTTATCACAGCTCAAGAATGGTTTATTAACCGCTCCACAAATCAACTAATAATACCACTGAGCGTAAAAGGACACAAATGAGCACTTCTATTGGCTTCATTAATAATCTTCCTGATTACAATTAATATACTAGGCCTTCTACCCTACACCTTCACACCCACAACACAACTATCACTTAACATAGGATTCGCCGTGCCCCTGTGACTTGCCACAGTTATTATTGGAATGCGAAACCAACCAACAATCGCACTAGGACATCTACTCCCAGAAGGGACACCCATCCCACTGATTCCAGTACTAATTATTATTGAAACAATCAGCCTATTTATTCGACCACTAGCCCTTGGAGTTCGACTTACAGCCAACCTGACCGCAGGCCATCTACTAATTCAACTTATTGCTACCGCCGTATTTGTCCTCCTACCAATAATACCAACAGTGGCAGTCCTAACTGCCGCCGTGCTTTTCCTTCTTACACTGCTGGAAGTTGCAGTTGCAATAATCCAAGCCTATGTCTTTGTACTTCTCCTAAGCCTATACCTTCAAGAGAACACT

>A._kreyenbergii_1

ATGATAGCAAGTTTTTTCGACCAATTTGAAAGCCCGATCTGCCTAGGAATCCCACTAATTGCCATCGCAATTGCTCTTCCCTGAGTACTTTACCCAACCCCTACATCTCGATGAATTAACAACCGACTTATTACAGCTCAAGAATGATTTATTAACCGCTCCACAAATCAACTAATAACACCACTAAGTGTAGAAGGACATAAATGAGCACTTCTATTAACCTCATTAATAATTTTCCTAATTACAATCAACATGTTAGGCCTTCTACCCTACACCTTTACACCCACCACACAACTATCACTTAATATAGGATTCGCTGTACCCTTATGACTTGCTACAGTTATTATTGGGATGCGAAACCAACCAACAATCGCACTAGGACATCTACTCCCAGAAGGAACACCCATCCCACTGATTCCAGTACTAATTATCATTGAAACAATCAGCCTATTTATTCGACCACTAGCCCTCGGAGTCCGACTTACAGCTAACCTAACCGCAGGTCACCTACTAATTCAACTCATCGCCACTGCCGTATTTGTCCTCCTACCAATGATACCGACAGTAGCAATCCTAACTGCCGCCGTACTTTTTCTTCTCACACTACTGGAAGTTGCAGTTGCAATAATTCAAGCCTATGTATTTGTCCTTCTTCTAAGTCTGTATCTTCAAGAAAACACT

>A._kreyenbergii_2

ATGATAGTAAGTTTTTTCGACCAATTTGAAAGCCCAATCTACCTAGGAATCCCCCTAATTGCCATCGCAATTGCTCTTCCCTGAGTACTTTATCCAACCCCCACATCTCGATGAATCAATAACCGACTTATTACAGCTCAAGAATGATTTATTAACCGCTCCACAAATCAACTAATAACACCACTAAGTGTAGAAGGACATAAATGAGCACTTCTATTAACCTCATTAATAATTTTCCTAATTACAATCAACATGCTAGGCCTCCTACCCTACACCTTTACACCCACAACACAACTGTCACTTAATATAGGATTCGCCGTACCCTTATGACTTGCTACAGTTATTATCGGAATGCGAAACCAACCAACAATCGCACTAGGACATCTACTCCCAGAAGGCACACCCATCCCACTGATTCCAGTACTAATTATCATTGAAACAATCAGCCTATTTATTCGACCGCTAGCCCTTGGAGTCCGACTTACAGCCAACCTAACCGCAGGTCACCTACTAATTCAACTCATCGCCACCGCCGTATTTGTCCTCCTACCAATAATACCAACAGTAGCAATCCTAACTGCCGCCGTACTCTTTCTTCTTACACTGCTGGAAGTTGCAGTTGCAATAATCCAAGCCTATGTGTTTGTCCTTCTTCTAAGTCTATATCTTCAAGAAAACACT

>A._longipinnis

ATGATAGTAAGTTTTTTCGACCAATTCGCAAGCCCATCCTACCTAGGAATCCCACTAATTGCCATCGCAATCGCCCTACCCTGAATACTCTACCCGACCCCATCATCTCGATGAATTAACAACCGACTTATTACAGCCCAAGAATGATTTATTAACCGCTCCACAAACCAATTAATGATACCACTGAGCATAGAAGGACATAAATGAGCACTACTACTAACTTCGCTAATAATCTTCTTAATCACAATCAACATGTTAGGCCTACTACCCTACACCTTCACCCCCACAACACAACTATCACTCAACATGGGATTCGCTGTACCACTTTGACTTGCTACAGTGATTATTGGAATACGAAACCAACCAACAGTCGCATTAGGACACCTGCTCCCAGAAGGAACACCCATCCCACTGATTCCAGTACTAATTATCATCGAAACAATTAGCCTATTTATTCGACCACTAGCCCTAGGAGTTCGACTTACAGCCAACCTAACTGCAGGTCACCTACTAATCCAACTTATTGCCACCGCCGTATTTGTCCTTCTACCAATAATGCCAACAGTAGCAATCCTAACTGCCGCCGTACTTTTCCTACTCACACTACTAGAAGTTGCAGTTGCAATGATCCAAGCCTACGTATTTGTACTTCTTCTAAGCCTATATCTACAAGAAAACACT

>A._monticola_1

ATGATAGCAAGCTTCTTCGATCAATTTGCAAGCCCCTCATACCTGGGAATCCCACTAATTGCCATCGCAATTGCCCTGCCCTGAGTACTTTACCCGACCCCATCATCCCGATGAATTAATAACCGACTCATCACAGCCCAAGAATGATTCATTAACCGCTCCACAAATCAACTAATACTACCACTAAGTGTAGAAGGACATAAATGAGCACTACTATTGACTTCACTAATAATTTTCTTAATTACAATTAATATGCTAGGCCTACTGCCATATACTTTTACACCCACAACACAACTATCACTTAATATAGGATTTGCTGTACCATTATGACTTGCCACGGTAATTATCGGAATACGAAACCAACCAACAGTTGCACTAGGACACCTTCTGCCAGAAGGAACACCTATCCTACTGATCCCAGTACTAATTATCATCGAAACAATTAGCCTATTTATCCGACCACTAGCCCTAGGAGTTCGGCTTACAGCCAACCTAACCGCAGGCCACCTGCTAATCCAACTTATCGCCACTGCCGTATTTGTCCTCTTACCTATAATACCGACAGTGGCAATTTTAACTGCCGCCGTACTATTTCTTCTTACACTACTAGAAGTGGCAGTTGCAATAATCCAAGCCTATGTATTCGTTCTTCTTTTAAGCCTATACCTGCAAGAAAACACT

>A._monticola_2

ATGATAGCAAGCTTCTTCGATCAATTTGCAAGCCCCTCATACCTGGGAATCCCACTAATTGCCATCGCAATTGCCCTGCCCTGAGTACTTTACCCGACCCCATCATCCCGATGAATTAATAACCGACTCATCACAGCCCAAGAATGATTCATTAACCGCTCCACAAATCAACTAATACTACCACTAAGTGTAGAAGGACATAAATGAGCACTACTATTGACTTCACTAATAATTTTCTTAATTACAATTAATATGCTAGGCCTACTGCCATATACTTTTACACCCACAACACAACTATCACTTAATATAGGATTTGCTGTACCATTATGACTTGCCACGGTAATTATCGGAATACGAAACCAACCAACAGTTGCACTAGGACACCTTCTGCCAGAAGGAACACCTATCCTACTGATCCCAGTACTAATTATCATCGAAACAATTAGCCTATTTATCCGACCACTAGCCCTAGGAGTTCGGCTTACAGCCAACCTAACCGCAGGCCACCTGCTAATCCAACTTATCGCCACTGCCGTATTTGTCCTCTTACCTATAATACCGACAGTGGCAATTTTAACTGCCGCCGTACTATTTCTTCTTACACTACTAGAAGTGGCAGTTGCAATAATCCAAGCCTATGTATTCGTTCTTCTTTTAAGCCTATACCTGCAAGAAAACACT

>A._paradoxus_1

ATGATAGTAAGTTTTTTCGACCAATTTGCAAGCCCAATTTATTTAGGGATTCCACTAATCGCCATCGCAATTGCCCTTCCCTGGGTACTCTACCCAACCCCCTCATCTCGATGAATTAACAACCGACTTATTTCAGCCCAAGAATGATTTATTAACCGCTCCACGAACCAACTAATAATACCACTAAGTGTAGAAGGACATAAATGAGCACTCCTATTAACTTCACTATTAATCTTCCTGATCACGATCAACATACTGGGTCTATTACCTTATACATTTACACCCACAACACAACTATCACTCAACATAGGATTCGCCGTACCCTTATGACTTGCTACAGTTATTATTGGAATGCGAAACCAACCAACAGTTGCACTAGGACATCTTCTCCCAGAAGGGACACCCATCCCACTGATTCCAGTACTAATTATCATCGAAACAATTAGCCTATTTATTCGACCACTGGCCCTAGGAGTTCGACTTACAGCCAACCTAACCGCAGGCCACCTACTAATCCAACTCATCGCCACCGCCGTATTTGTTCTCATACCGATGATACCAACAGTAGCAATCCTAACTGCCGCCGTACTTTTTCTACTTACACTACTAGAAGTTGCAGTTGCAATAATCCAAGCCTATGTATTTGTACTTCTCTTAAGCCTATACCTTCAAGAAAACACT

>A._paradoxus_2

ATGATAGTAAGTTTTTTCGACCAATTTGAAAGCCCTATTTACCTAGGGATCCCACTAATTGCCATCGCAATTGCTCTTCCCTGAGTACTTTACCCAACCCCCACATCTCGATGAATCAACAACCGACTTATCACAGCTCAAGAATGGTTTATTAACCGCTCCACAAATCAACTAATAATACCACTGAGCGTAAAAGGACACAAATGAGCACTTCTATTGGCTTCATTAATAATCTTCCTGATTACAATTAATATACTAGGCCTTCTACCCTACACCTTCACACCCACAACACAACTATCACTTAACATAGGATTCGCCGTGCCCCTGTGACTTGCCACAGTTATTATTGGAATGCGAAACCAACCAACAATCGCACTAGGACATCTACTCCCAGAAGGGACACCCATCCCACTGATTCCAGTACTAATTATTATTGAAACAATCAGCCTATTTATTCGACCACTAGCCCTTGGAGTTCGACTTACAGCCAACCTGACCGCAGGCCATCTACTAATTCAACTTATTGCTACCGCCGTATTTGTCCTCCTACCAATAATACCAACAGTGGCAGTCCTAACTGCCGCCGTGCTTTTCCTTCTTACACTGCTGGAAGTTGCAGTTGCAATAATCCAAGCCTATGTCTTTGTACTTCTCCTAAGCCTATACCTTCAAGAGAACACT

>A._parallens_1

ATGATAGCAAGTTTTTTCGACCAATTTGAAAGCCCTGTTTACCTAGGAGTCCCACTAATCGCCATCGCAATTGCTCTTCCCTGAGTACTTTACCCAACCCCCACATCTCGATGGATTAACAACCGACTTATTACAGCCCAAGAATGATTTATTAACCGCTCCACAAATCAACTAATAACACCACTAAGCGTAGAAGGACATAAATGAGCACTTCTATTAACTTCATTAATAATTTTCCTGATTACAATCAATATGCTGGGCCTTCTACCCTACACCTTCACACCTACAACACAACTATCACTTAATATAGGATTCGCCGTACCCCTGTGACTTGCTACAGTTATTATTGGAATGCGAAACCAACCAACAATCGCACTAGGACATCTACTCCCAGAAGGGACACCTATCCCACTGATTCCAGTACTAATTATTATTGAAACAATCAGCCTATTTATTCGACCGCTAGCCCTTGGAGTTCGACTTACAGCCAACCTGACCGCAGGTCACCTACTAATTCAACTTATCGCCACCGCCGTATTTGTCCTCCTGCCAATAATACCAACAGTGGCAGTCCTGACTGCCGCCGTGCTTTTCCTTCTTACACTATTGGAAGTTGCAGTTGCAATAATCCAAGCCTATGTGTTTGTACTTCTCCTAAGCCTGTACCTTCAAGAAAACACT

>A._parallens_2

ATGATAGCAAGTTTTTTCGACCAATTTGAAAGCCCTATTTACCTGGGAATCCCACTAATCACCATCGCAATTGCTCTTCCCTGAGTACTTTACCCAACCCCCACATCTCGATGAATTAACAACCGACTTATTACAGCTCAAGAATGATTTATTAACCGCTCCACAAATCAACTAATAACACCACTAAGTGTACAAGGACATAAATGAGCACTTCTATTAACTTCGTTAATAATTTTCCTGATTACAATCAATATGCTAGGCCTTCTGCCCTACACCTTCACACCTACAACACAACTATCACTTAATATAGGATTCGCCGTGCCCCTGTGACTTGCTACAGTTATTATTGGAATGCGAAACCAACCAACAGTCGCACTAGGACATCTACTCCCAGAAGGGACACCTATCCCACTGATTCCAGTACTAATCATTATTGAAACAATCAGCCTATTTATTCGACCACTAGCCCTTGGAGTTCGACTTACAGCCAACCTGACCGCAGGTCACCTACTAATTCAACTTATCGCCACCGCCGTATTTGTCCTCCTGCCAATAATACCAACAGTGGCAATCCTGACTGCCGCCGTGCTTTTCCTTCTTACACTATTGGAAGTTGCAGTTGCAATGATCCAAGCCTATGTGTTTGTACTTCTCCTAAGCCTATACCTTCAAGAAAACACT

>A._parallens_3

ATGATAGCAAGTTTTTTCGACCAATTTGAAAGCCCTATTTACCTAGGAGTCCCACTAATCGCCATCGCAATTGCTCTTCCCTGAGTACTTTACCCAACCCCCACATCTCGATGAATTAACAACCGACTTATTACAGCCCAAGAATGATTTATTAACCGCTCCACAAATCAACTAATAACACCACTAAGCGTAGAAGGACATAAATGAGCACTTCTATTAACTTCATTAATAATTTTCCTGATTACAATCAATATGCTAGGCCTTCTACCCTACACCTTCACACCTACAACACAACTATCACTTAATATAGGATTCGCCGTACCTCTGTGACTTGCTACAGTTATTATTGGAATGCGAAACCAACCAACAATCGCACTAGGACATCTACTTCCAGAAGGGACACCTATCCCACTGATTCCAGTACTAATTATTATTGAAACAATCAGCCTATTTATTCGACCGCTAGCCCTTGGAGTTCGACTTACAGCCAACCTGACCGCAGGTCACCTACTAATTCAACTTATCGCCACCGCCGTATTTGTCCTCCTGCCAATAATACCAACAGTGGCAGTCCTGACTGCCGCCGTGCTTTTCCTTCTTACACTATTGGAAGTTGCAGTTGCAATAATCCAAGCCTATGTGTTTGTACTTCTCCTAAGCCTATACCTTCAAGAAAACACT

>A._parallens_4

ATGATAGCAAGTTTTTTCGACCAATTTGAAAGCCCTATTTACCTAGGAGTCCCACTAATCGCCATCGCAATTGCTCTTCCCTGAGTACTTTACCCAACCCCCACATCTCGATGAATTAACAACCGACTTATTACAGCCCAAGAATGATTTATTAACCGCTCCACAAATCAACTAATAACACCACTAAGCGTAGAAGGACATAAATGAGCACTTCTATTAACTTCATTAATAATTTTCCTGATTACAATCAATATGCTAGGCCTTCTACCCTACACCTTCACACCTACAACACAACTATCACTTAATATAGGATTCGCCGTACCCCTGTGACTTGCTACAGTTATTATTGGAATGCGAAACCAACCAACAATCGCACTAGGACATCTACTTCCAGAAGGGACACCTATCCCACTGATTCCAGTACTAATTATTATTGAAACAATCAGCCTATTTATTCGACCGCTAGCCCTTGGAGTTCGACTTACAGCCAACCTGACCGCAGGTCACCTACTAATTCAACTTATCGCCACCGCCGTATTTGTCCTCCTGCCAATAATACCAACAGTGGCAGTCCTGACTGCCGCCGTGCTTTTCCTTCTTACACTATTGGAAGTTGCAGTTGCAATAATCCAAGCCTATGTGTTTGTACTTCTCCTAAGCCTATACCTTCAAGAAAACACT

>A._spinifer

ATGATAGTAAGTTTTTTCGACCAATTTGAAAGCCCAATCTTCCTAGGGATCCCGCTAATTGCCATCGCAATTGCCCTTCCCTGAGTACTCTACCCAACCCCCTCATCTCGATGAATTAACAACCGACTTATTACAGCCCAAGAATGGTTTATTACCCGCTCCACAAACCAACTAATAACACCACTAAGTGCAGAAGGACATAAATGAGCACTTCTATTAACTTCATTAATAATCTTCCTGATTACAATCAACATACTGGGCCTACTACCTTACACATTCACACCCACAACACAACTATCACTCAACATAGGATTCGCCGTACCCTTATGACTTGCCACAGTTATTATTGGAATGCGAAACCAACCAACAGTCGCACTAGGACATCTACTCCCAGAAGGGACACCCATCCCACTGATCCCAGTACTAATCATCATCGAAACAATTAGCCTATTTATTCGACCGCTAGCCCTAGGAGTTCGACTTACAGCCAACCTAACCGCAGGCCACCTACTAATCCAACTCATCGCCACTGCCGTATTTGTCCTTCTACCAATAATACCAACAGTAGCAATCTTAACTGCCGCCGTACTTTTTCTACTCACATTGCTAGAAGTTGCAGTTGCAATAATCCAAGCCTATGTATTTGTACTTCTCTTAAGCCTATACCTTCAAGAAAACACT

>A._stenotaeniatus

ATGATAGTAAGTTTTTTCGACCAATTTGAAAGCCCAATCTTCCTAGGGATCCCGCTAATTGCCATTGCAATTGCCCTTCCCTGAGTACTCTACCCAGCCCCCGCATCTCGATGAATTAACAACCGACTTATTACAGCCCAAGAATGGTTTATTACCCGCTCCACAAACCAACTAATAATACCACTAAGTGCAGAAGGACATAAATGAGCACTTCTATTAGCTTCATTAATAATCTTCCTGATTACAATCAACATACTGGGCCTACTACCTTACACATTCACACCCACAACACAACTATCACTCAACATAGGATTCGCTGTACCCTTATGACTTGCCACAGTTATTATTGGAATGCGAAACCAACCAACAGTCGCACTAGGACATCTACTCCCAGAAGGGACACCCATTCCACTGATCCCAGTACTAATCATCATCGAAACAATTAGCCTATTTATTCGACCGCTAGCCCTAGGAGTTCGACTTACAGCCAACCTAACCGCAGGCCACCTACTAATCCAACTCATCGCCACTGCCGTATTTGTTCTTCTACCAATGATACCAACAGTAGCAATCTTAACTGCCGCCGTACTTTTTCTACTCACATTGCTAGAAGTTGCAGTTGCAATAATCCAAGCCTATGTATTTGTACTTCTCTTAAGCCTATACCTTCAAGAAAACACT

>A._wenchowensis_1

ATGATAGTAAGTTTTTTCGACCAATTTGAAAGCCCAATCTACCTAGGAATCCCACTAATTGCCATCGCAATTGCTCTTCCCTGAGTACTCTACCCGACCCCTACATCTCGATGAATTAACAACCGACTTATTACAGCTCAAGAATGGTTTATTAACCGCTCCACAAATCAACTAATGACACCACTAAGTGTAGAAGGACATAAATGGGCACTCCTATTAACCTCATTAATAATCTTCCTAATTACAATCAATATGCTAGGCCTTCTACCCTACACCTTTACACCCACAACGCAACTATCACTTAATATAGGATTCGCCGTGCCCTTATGACTTGCTACAGTTATTATTGGAATGCGAAACCAACCAACAATCGCACTAGGACATCTACTCCCAGAAGGAACACCTATCCCACTGATTCCGGTACTAATTATCATTGAAACAATCAGCCTATTTATTCGACCACTAGCCCTTGGAGTCCGACTTACAGCCAACCTAACCGCAGGCCACCTACTAATTCAACTTATCGCCACCGCTGTATTTGTCCTCCTACCAATAATGCCAACAGTAGCAATCCTAACTGCCGCCGTGCTTTTTCTTCTAACACTACTAGAAGTAGCAGTTGCAATAATCCAAGCCTATGTATTTGTCCTCCTTCTAAGTCTTTATCTTCAAGAAAACACT

>A._wenchowensis_2

ATGATAGTAAGTTTTTTCGACCAATTTGAAAGCCCAATCTACCTAGGAATCCCACTAATTGCCATTGCAATTGCTCTTCCCTGAGTACTCTACCCAACCCCCACATCTCGATGAATTAACAACCGACTCATTACAGCTCAAGAATGGTTTATTAACCGCTCCACAAATCAACTAATAACACCACTAAGTGTAGAAGGACATAAATGGGCACTCCTATTAACTTCATTAATAATCTTCCTAATTACAATCAATATGCTAGGCCTCCTACCCTACACCTTTACACCGACAACACAACTATCACTTAATATAGGATTCGCCGTGCCCCTATGACTTGCTACAGTTATTATTGGAATGCGAAACCAACCAACAGTCGCACTAGGACACCTACTCCCAGAAGGGACACCCATCCCACTGATTCCAGTACTAATTATCATTGAAACAATTAGCCTATTTATTCGACCGCTAGCCCTTGGAGTCCGACTTACAGCCAACCTAACCGCCGGCCACCTACTAATCCAACTCATCGCCACCGCCGTATTTGTCCTCCTACCAATAATACCAACAGTAGCAGTCCTAACTGCCGCCGTGCTCTTTCTTCTAACACTACTAGAAGTTGCAGTTGCAATAATCCAAGCCTATGTGTTTGTCCTTCTTCTAAGTCTATATCTTCAAGAAAACACT

>A._wuyiensis

ATGATAGTAAGTTTTTTCGACCAATTTGCAAGCCCAATTTATTTAGGGATCCCACTAATCGCCGTCGCAATTGCCCTTCCCTGGGTACTCTACCCGACCCCCTCATCTCGATGAATTAACAACCGACTTATTTCAGCCCAAGAATGATTTATTAACCGCTCCACAAACCAACTAATAATACCACTAAGTGTAGAAGGACATAAATGAGCACTCCTATTAACTTCGCTATTAATCTTCCTAATCACGATCAACATACTGGGTCTATTACCTTATACATTTACACCCACAACACAACTATCACTCAACATAGGATTCGCCGTACCCTTATGACTTGCTACAGTTATTATTGGAATGCGAAACCAACCAACAGTTGCACTAGGACATCTTCTCCCAGAAGGGACACCCATCCCACTGATTCCAGTACTAATTATCATCGAAACAATTAGCCTATTTATTCGACCACTGGCCCTAGGAGTTCGACTTACAGCCAACCTAACCGCAGGCCACCTACTAATCCAACTCATCGCCACCGCCGTATTTGTTCTCATACCGATGATACCAACAGTAGCAATCCTAACTGCCACCGTACTTTTTCTACTTACACTACTAGAAGTTGCAGTTGCAATAATCCAAGCCTATGTATTTGTACTTCTCTTAAGCCTATACCTTCAAGAAAACACT

>A._yunnanensis_1

ATGATAACAAGCTTCTTCGACCAATTTGCAAGCCCATCATACCTAGGGATTCCGCTAATTGCCATTGCAATTGCCCTGCCTTGAGTGCTCTACCCGACCCCATCATCCCGATGAATTAATAACCGACTTATTACAGCTCAAGAATGATTTATTAACCGCTCCACAAATCAACTAATGCTACCACTAAGCGTAGAAGGACATAAATGAGCACTACTACTAACTTCACTAATAATCTTCTTAATTACAATTAATATGCTAGGACTACTACCTTATACCTTTACACCCACAACACAACTATCACTTAATATAGGATTTGCTGTACCACTATGACTTGCCACAGTAATTATTGGAATGCGGAACCAACCAACAATCGCACTAGGACACCTTCTGCCAGAAGGGACACCCATTCTACTGATCCCAGTACTAATTATCATCGAAACAATTAGCCTATTTATCCGACCACTAGCCCTGGGAGTTCGACTTACAGCCAACCTAACCGCGGGCCACTTACTAATCCAACTTATCGCCACCGCCGTATTTGTTCTTTTACCAATGATACCAACAGTAGCAATTCTGACTGCCGCCGTCCTATTTCTACTTACACTACTAGAAGTAGCAGTCGCAATAATCCAAGCCTATGTATTTGTACTTCTTTTAAGCCTGTACCTACAAGAAAACACT

>A._yunnanensis_2

ATGATAACAAGCTTCTTCGACCAATTTGCAAGCCCATCATACCTAGGAATTCCGCTAATTGCCGTTGCAATTGCCCTGCCTTGAGTACTCTACCCGACCCCATCATCCCGATGAATTAATAACCGACTTATCACAGCTCAAGAGTGATTTATTAACCGCTCCACAAATCAACTAATGCTCCCACTAAGCGTAGAAGGACATAAATGAGCACTACTACTAACCTCACTAATAATCTTCTTAATTACAATTAATATGCTAGGGCTACTACCTTATACCTTTACACCCACAACACAACTATCACTTAATATAGGATTTGCTGTACCACTATGACTTGCCACAGTAATTATTGGAATGCGGAACCAACCAACAGTCGCACTAGGGCACCTTCTGCCAGAAGGGACACCCATTCCACTGATCCCAGTACTAATCATCATCGAAACAATTAGCCTATTTATCCGACCACTAGCCCTGGGAGTTCGACTTACAGCCAACCTAACCGCGGGCCATTTACTAATCCAACTTATCGCCACCGCCGTATTTGTTCTTTTACCAATAATACCAACAGTAGCAATTCTAACTGCCGCCGTCTTATTTCTTCTTACGCTACTAGAAGTAGCAGTCGCAATAATCCAAGCCTATGTATTTGTACTTCTTTTAAGCCTGTACCTACAAGAAAACACT

>Onychostoma_barbatulum

ATGATAGCGAGCTTCTTCGACCAATTTGCAAGCCCTTCCTATTTAGGAATTCCACTAATTGCCATCGCAATCGCCTTACCTTGAGTACTATACCCAACCCCATCATCTCGTTGAATTAATAACCGACTTATTACAGCCCAAGAATGGTTTATTAACCGCTCCACAAATCAACTAATAATACCACTGAGCATAGAAGGACACAAATGAGCACTACTACTAACATCATTAATAATTTTTTTAATTACAATTAATATACTAGGCCTACTACCCTACACCTTCACACCCACAACACAGTTATCACTCAACATAGGATTCGCCGTACCACTGTGACTTGCCACAGTAATCATTGGAATGCGAAATCAACCTACAGTCGCACTAGGACACCTTCTTCCAGAAGGGACACCTATCCCACTAATCCCCGTACTTATTATCATCGAAACAATCAGCCTGTTTATCCGACCACTAGCCCTAGGGGTTCGACTTACAGCCAATCTAACCGCAGGTCACCTATTAATCCAACTTATCGCCACCGCTGTATTTGTTCTTCTACCAATAATACCTACAGTAGCAATCCTCACTGCCGCCGTACTATTCCTGCTTACACTTCTAGAAGTAGCAGTTGCAATAATCCAAGCTTATGTATTTGTACTTCTTTTAAGCCTATACCTACAAGAAAACACT

>Onychostoma_meridionale

ATGATAGCAAGCTTCTTCGATCAATTTGCCAGCCCCTCCTTCCTAGGAATACCATTAATTGCCGTCGCAATTGCACTGCCTTGAGTACTCTACCCAACCCCGTCATCTCGATGAATTAACAACCGACTTATTACAGCCCAAGAATGATTTATTAACCGCTCCACAAATCAATTAATAACACCACTAAGTGTAGAAGGACATAAATGAGCACTACTACTAACTTCGCTAATAATCTTCTTAATCACAATTAATATGTTAGGCCTACTGCCCTACACCTTCACACCCACAACACAACTATCACTCAACATAGGATTCGCTGTGCCACTATGACTTGCTACTGTGATTATTGGAATGCGAAATCAACCAACAGTTGCACTAGGACACCTCCTTCCAGAAGGAACACCCATCCCGCTAATCCCAGTACTAATTATCATCGAAACAATTAGCCTATTTATTCGACCTCTAGCCCTTGGGGTTCGACTTACAGCCAATTTAACCGCAGGCCACCTACTCATCCAACTTATCGCCACCGCTGTATTTGTTCTACTACCAATAATGCCAACAGTCGCAATTTTAACTGCTGCCGTACTCTTCCTGCTTACACTATTAGAAGTTGCAGTTGCAATAATCCAAGCCTATGTATTTGTACTTCTTTTAAGCCTGTATCTACAAGAAAACACC

>Onychostoma_gerlachi

ATGATAGCAAGCTTCTTCGATCAATTCGCCAGCCCCTCCTTCCTAGGAGTCCCACTAATTGCCATCGCAATTGCACTGCCTTGAGTACTTTACCCAACCCCATCATCTCGATGAATTAATAACCGACTTATCACAGCCCAAGAATGATTTATTAACCGCTCTACAAATCAATTAATAACGCCACTGAGTGTAGAAGGACATAAATGAGCACTACTACTAACCTCACTAATAATTTTCTTAATTACAATTAACATGCTAGGCCTGCTACCCTACACCTTCACACCCACAACACAACTATCACTCAACATGGGATTCGCCGTCCCGCTATGGCTCGCCACAGTAATTATTGGAATGCAAAATCAACCAACAGTCGCGCTAGGACATCTTCTCCCAGAAGGAACACCCATCCCACTAATCCCAGTACTAATTATCATCGAAACAATTAGCTTATTTATTCGACCTCTCGCCTTAGGGGTTCGACTTACAGCCAACTTAACCGCAGGCCACCTACTCATCCAACTTATCGCCACCGCCGTATTTGTCCTCCTGCCAATGATACCAACAGTAGCAATTTTAACTGCTGCCGTACTCTTCCTGCTCACACTACTAGAAGTTGCAGTTGCGATAATTCAAGCCTATGTATTTGTACTTCTTTTAAGCCTATATCTACAAGAAAACACC

>Spinibarbus_denticulatus

ATGATAGTAAGCTTCTTCGACCAATTTGCAAGCCCCTCACACCTAGGAATCCCACTTATTGCTATTGCAATCGCACTCCCCTGAGTACTTTATCCAACCCCATCATCTCGATGAATTAATAACCGACTCATTACAATCCAAGGATGATTTATTAACCGATTCACAAACCAATTAATACTTCCACTGAATGTAGGAGGCCATAAATGAGCACTACTACTAACCTCGTTAATAATCTTCTTACTGACAATCAATATGCTAGGCCTGTTACCATACACCTTCACGCCCACAACACAACTATCACTTAATATAGGATTCGCCGTACCATTATGACTTGCTACAGTGATTATTGGGATACGGAACCAACCAACGGTTGCACTAGGACATCTACTACCAGAAGGAACACCTATCCCATTAATCCCTGTATTAATTATTATCGAAACAATCAGCCTGTTTATCCGACCCTTAGCCCTAGGGGTTCGACTCACAGCCAACTTAACCGCAGGTCACCTATTAATCCAACTCATCGCCACAGCCGTATTTGTTCTCCTACCAATAATACCAACAGTAGCAATTCTAACCGCTACCGTACTTTTTCTACTTACACTACTAGAAGTTGCAGTAGCAATAATTCAAGCTTATGTATTTGTACTCCTTTTAAGCCTATACCTACAAGAAAACGTT

>Spinibarbus_hollandi

ATGATAGTAAGCTTCTTCGACCAATTTGCAAGCCCCTCACACCTAGGAATCCCGCTTATTGCTATTGCAATCGCACTGCCCTGAGTACTTTACCCAACCCCCTCATCTCGATGAATTAATAATCGACTTATTACAGTCCAAGGATGACTTATTAACCGATTCACAAACCAACTAATACTTCCACTGAATGTAAAAGGTCATAAATGAGCACTACTACTAACCTCATTAATAATCTTCTTACTAACAATTAATATGCTAGGCCTATTACCATACACCTTTACACCCACGACACAACTATCACTTAATATAGGATTCGCTATACCACTATGACTTGCTACAGTAATTATTGGGATACGGAACCAACCAACGATTGCACTAGGACATCTATTACCAGAAGGAACACCTATCCCCTTAATCCCTGTATTAATTATTATCGAAACAATCAGCCTGTTTATCCGGCCTTTGGCCCTAGGGGTTCGACTCACAGCCAACTTAACCGCAGGTCACCTATTAATTCAACTCATCGCCACAGCCGTGTTTACCCTCCTACCAATAATACCAACAGTAGCAATTCTAACCGCCACCGTACTTTTTCTACTTACATTATTAGAAATTGCAGTAGCAATAATTCAAGCTTATGTATTTGTACTCCTTTTAAGCCTATATCTACAAGAAAACGTT

>Spinibarbus_sinensis

ATGATAGTAAGCTTCTTCGACCAATTTGCAAGCCCATCATATCTAGGAATCCCACTAATTGCCATCGCAATTGCTCTACCCTGAGTACTTTATCCAACCCCATCATCTCGATGGATTAACAACCGACTTATCACGGTCCAAGGATGATTTATTAACCGATTCACGAACCAACTAATACTTCCACTAAATGTAGGAGGACATAAATGAGCACTACTACTAGCTTCATTAATAATCTTTTTAATTACAATTAATATGCTAGGCCTACTACCATATACCTTCACACCAACAACGCAACTATCACTTAACATAGGATTTGCCGTACCACTATGACTCGCTACGGTAATTATTGGAATGCGGAATCAACCAACAGTTGCATTAGGACACCTATTACCAGAAGGAACACCTATCCTACTGATCCCAGTACTAATTATTATCGAAACAATTAGCCTATTTATCCGACCGCTAGCTCTAGGAGTTCGACTCACAGCTAACCTGACCGCAGGTCACCTATTAATTCAACTCATCGCCACAGCTGTATTTGTTCTCCTGCCAATAATACCAACAGTAGCAATCTTAACTGCTACTGTACTCTTTTTACTCACATTATTAGAAGTCGCAGTAGCAATAATTCAAGCTTATGTGTTTGTGCTTCTTCTAAGCCTATATTTACAAGAAAACGTT

**ATP8**

>A._barbodon

ATGCCACAATTAAACCCCGGCCCTTGATTCATAATCTTAGTATTCTCTTGACTAATTTTCTTAACCATTATTCCAACCAAAATCTTAAGCCACACCACACCAAATGAACCAACCCCAGTAAGTGCTGAAAAACACAAGACTGAATACTGAGACTGACCATGATAG

>A._beijiangensis_1

ATGCCACAACTAAACCCCGGCCCTTGATTCATGATTTTAACATTCTCTTGACTTATCTTCTTAACTATTATCCCAACCAAAATCTTAAACCACACTACACCAAATGAACCGACCTCAGTAAGTGCTGAAAAACACAAAGCTGAGTACTGAGACTGACCATGATAG

>A._beijiangensis_2

ATGCCACAACTAAACCCCGGCCCTTGATTTATGATTTTAACATTCTCTTGACTTATCTTCTTAACTATTATCCCAACCAAAATCTTAAACCACACCACACCAAATGAACCGACCTCAGTAAGTGCTGAAAAACACAAAGCTGAGTACTGAGACTGACCATGATAG

>A._fasciatus

ATGCCACAATTAAACCCCGGCCCTTGATTCATGATTTTAGTATTCTCTTGACTTATTTTCTTAACTGTTATTCCAACTAAAATCTTAAACCACACCACACCAAATGAACCATCCTTAGTAAGTGCTGAAAAACACAAAACTGAGTACTGAGACTGACCATGATAG

>A._hemispinus

ATGCCACAATTAAACCCCGGCCCTTGATTCATGATTTTAGTGTTCTCCTGACTAATTTTCTTAACCATTATCCCAACTAAAATCTTAAACCACACCACACCAAATGAACCAACCTTAGTAAGTGCTGAAAAACACAAAACTGAGTACTGAGACTGACCATGATAG

>A._iridescens

ATGCCACAATTAAACCCCGGCCCTTGATTCATAATCTTAGTATTCTCTTGGCTAATTTTCTTAACCATTATTCCAACCAAAATCTTAAACCACACCACACCAAATGAACCGACCCCAGTAAGTGCTGAAAAACACAAGACTGAATACTGAGACTGACCATGATAG

>A._jishouensis

ATGCCACAATTAAACCCCGGCCCTTGATTCATGATTTTAGTATTCTCTTGACTTATTTTCCTAACTATTATTCCAACTAAAATTTTAAACCATACTACACCAAATGAACCGACCTTAGTAAGTGCTGAAAAACACAAAACTGAGTACTGAGACTGACCATGATAG

>A._kreyenbergii_1

ATGCCACAATTAAACCCCGGCCCTTGATTCATGATTTTAGTATTCTCTTGACTTATTTTCTTAACTATTATTCCAACTAAAATCTTAAACCACACTACACCAAATGAACCATCCTTAGTGAGTGCTGAAAAACACAAAACTGAGTACTGAGACTGACCATGATAG

>A._kreyenbergii_2

ATGCCACAATTAAACCCCGGCCCTTGATTCATGATTTTAGTATTCTCTTGACTTATTTTCTTAACTATTATTCCAACTAAAATCTTAAACCACACTACACCAAATGAACCATCCTTAGTGAGTGCTGAAAAACACAAAACTGAGTACTGAGACTGACCATGATAG

>A._longipinnis

ATGCCACAATTAAACCCCGGCCCTTGATTCATAATCTTAGTATTCTCCTGACTAATTTTCTTAACCATTATTCCAACCAAAATCTTAAACCACACCACACCAAATGAACCGACCCCAGTAAGTGCTGAAAAACACAAGACTGAATACTGAGACTGACCATGATAG

>A._monticola_1

ATGCCACAATTAAACCCCGGCCCTTGATTCATAATTTTAGTGTTTTCTTGATTAATCTTCTTAACTATTATCCCAACCAAAATCTTAAACCATACCTCACCAAACGAACCAACCCCAGTAAGTGCTGAAAAACACAAAACTGAATACTGAGACTGACCATGATAG

>A._monticola_2

ATGCCACAATTAAACCCCGGCCCTTGATTCATAATTTTAGTGTTTTCTTGATTAATCTTCTTAACTATTATCCCAACCAAAATCTTAAACCATACCTCACCAAACGAACCAACCCCAGTAAGTGCTGAAAAACACAAAACTGAATACTGAGACTGACCATGATAG

>A._paradoxus_1

ATGCCACAACTAAACCCCGGCCCTTGATTCATAATTCTAGTATTCTCTTGACTTATCTTCTTAATTATTATACCAACCAAAATCTTAAGCCACACCACACCAAATGAACCAACCTCAGTAAGTGCTGAAAAACACAAGACTGAGTACTGAGACTGACCATGATAG

>A._paradoxus_2

ATGCCACAATTAAACCCCGGCCCTTGATTCATGATTTTAGTATTCTCTTGACTTATTTTCCTAACTATTATTCCAACTAAAATTTTAAACCATACTACACCAAATGAACCGACCTTAGTAAGTGCTGAAAAACACAAAACTGAGTACTGAGACTGACCATGATAG

>A._parallens_1

ATGCCACAATTAAACCCCGGCCCTTGATTCATGATTTTAGTGTTCTCCTGACTAATTTTCTTAACCACTATCCCAACTAAAATCTTAAACCACACCACACCAAATGAACCAACCTTAGTAAGTGCTGAAAAACACAAAACTGAGTACTGAGACTGACCATGATAG

>A._parallens_2

ATGCCACAATTAAACCCCGGCCCTTGACTCATGATTTTAGTGTTCTCCTGACTAATTTTCTTAACCATTATCCCAACTAAAATCTTAAACCACACCACACCAAATGAACCAACCTTAGTAAGTGCTGAAAAACACAAAACTGAGTACTGAGACTGACCATGATAG

>A._parallens_3

ATGCCACAATTAAACCCCGGCCCCTGATTCATGATTTTAGTGTTCTCCTGACTAATTTTCTTAACCATTATCCCAACTAAAATCTTAAACCACACCACACCAAATGAACCAACCTTAGTAAGTGCTGAAAAACACAAAACTGAGTACTGAGACTGACCATGATAG

>A._parallens_4

ATGCCACAATTAAACCCCGGCCCTTGATTCATGATTTTAGTGTTCTCCTGACTAATTTTCTTAACCATTATCCCAACTAAAATCTTAAACCACACCACACCAAATGAACCAACCTTAGTAAGTGCTGAAAAACACAAAACTGAGTACTGAGACTGACCATGATAG

>A._spinifer

ATGCCACAACTAAACCCCGGCCCTTGATTCATGATTTTAGCATTCTCTTGACTTATCTTCTTAACTATTATCCCAACCAAAATCTTAAACCACACTACACCAAATGAACCGACCTCAGTAAGTGCTGAAAAACACAAAGCTGAGTACTGAGACTGACCATGATAG

>A._stenotaeniatus

ATGCCACAACTAAACCCCGGCCCTTGATTCATGATCTTAGCATTCTCTTGACTTATCTTCTTAACTATTATCCCAACCAAAATCTTAAACCACACTACACCAAATGAACCGACCTCAGTAAGTGCTGAAAAACACAAAGCTGAGTACTGAGACTGACCATGATAG

>A._wenchowensis_1

ATGCCACAATTAAACCCCGGCCCCTGATTCATGATTTTAGTATTCTCTTGACTTATTTTCTTAACTATTATTCCAACTAAAATCTTAAACCACACCACACCAAATGAACCATCCTTAGTAAGTGCTGAAAAACACAAAACTGAGTACTGAGACTGACCATGATAG

>A._wenchowensis_2

ATGCCACAATTAAACCCCGGCCCTTGATTCATGATTTTAGTATTCTCTTGACTTATTTTCTTAACTGTTATTCCAACTAAAATCTTAAACCACACCACACCAAATGAACCATCCTTAGTAAGTGCTGAAAAACACAAAACTGAGTACTGAGACTGACCATGATAG

>A._wuyiensis

ATGCCACAACTAAACCCCGGCCCTTGATTTATAATTCTAGTATTCTCTTGACTTATCTTCTTAATTATTATACCAACCAAAATCTTAAGCCACACCACACCAAATGAACCAACCTCAGTAAGTGCTGAAAAACACAAGACTGAGTACTGAGACTGACCATGATAG

>A._yunnanensis_1

ATGCCACAATTAAACCCCGGCCCTTGATTCATAATTTTAGTATTCTCTTGATTAATCTTCTTAACCATTATCCCAACCAAAATCTTAAACCACACCTCACCAAATGAACCAACCCCAGTAAGTGCTGAAAAACACAAAACTGAATACTGAGACTGACCATGATAA

>A._yunnanensis_2

ATGCCACAATTAAACCCCGGCCCTTGATTCATAATTTTAGTATTCTCTTGATTAATCTTCTTAACTATTATCCCAACCAAAATCTTAAACCACACCTCACCAAATGAACCAACCCCAGTAAGTGCTGAAAAACACAAAACTGAATACTGAGACTGACCATGATAA

>Onychostoma_barbatulum

ATGCCACAATTAAACCCCGGCCCTTGATTTATGATTTTAGTATTCTCCTGATTAGTTTTCCTAATTATTATTCCAACCAAAACCTTAAACCACACCACACCAAATAAACCAACCCCAGTAAGTGCTGAAAAACACAAAACTGAACACTGAGACTGACCATGATAG

>Onychostoma_meridionale

ATGCCACAATTAAACCCCGGCCCTTGATTCATAATTTTAATATTCTCTTGACTAGTTTTCTTAACTATTATCCCAACCAAAATCTTAAACCACACTACACCAAATGAACCAACCCCGGTGAGTGCCGAAAAACACAAAACTGAATACTGAGACTGACCATGATAG

>Onychostoma_gerlachi

ATGCCACAATTAAACCCCGGCCCTTGATTCATAATTTTAATGTTCTCTTGACTAGTTTTCTTAACTATTATCCCAACCAAAATCTTAAATCACACTACACCAAATGAACCAACCCCAGTGAGTGCCGAAAAACACAAAACTGAATACTGAGACTGACCATGATAG

>Spinibarbus_denticulatus

ATGCCACAATTAAACCCCGGCCCTTGATTCGCAATTTTGATATTTTCTTGACTAATTTTTTTAACTGTTATCCCAACTAAAATCTTAAACCACATTTCACCAAATGAACTAACCCCAGTAAGTGCTGAAAAACACAAAACTGAATCCTGAGACTGACCATGATAG

>Spinibarbus_hollandi

ATGCCACAATTAAACCCCAGCCCTTGATTTGCAATTTTGGTATTTTCTTGACTAATCTTTCTAACTGTTGTCCCAACTAAAATCTTAAACCACATTTCACCAAATGAACCAACCCCAGTAAGTGCTGAAAAACACAAAACTGAATCCTGAGACTGACCATGATAG

>Spinibarbus_sinensis

ATGCCACAATTAAACCCCGGCCCTTGATTCGCAATTTTAATATTTTCCTGACTAATTTTCCTAACTATCATCCCAACTAAAATCTTAAACCACATTTCACCAAACGAACCAACCCCAGTAAGTGCTGAAAAACACAAAACTGAATCCTGAGACTGACCATGATAG

**COX1**

>A._barbodon

GTGGCAATCACACGCTGATTCTTCTCTACCAACCACAAAGACATTGGTACCCTTTATCTCGTATTTGGTGCCTGAGCCGGAATAGTGGGAACTGCCCTAAGCCTTTTAATTCGGGCCGAGCTAAGTCAACCCGGGTCACTTCTAGGTGATGATCAAATCTATAATGTTATCGTAACCGCCCACGCCTTTGTTATAATTTTCTTTATAGTAATACCTATCCTCATTGGAGGGTTCGGAAATTGACTCGTACCGCTAATAATTGGGGCCCCCGACATAGCATTCCCACGAATAAATAATATAAGTTTCTGACTACTACCCCCATCATTCCTGCTACTACTAGCCTCTTCTGGTGTTGAGGCGGGAGCCGGAACAGGGTGAACAGTTTACCCGCCCCTTGCAGGAAACCTAGCTCACGCAGGGGCATCAGTAGACCTAACAATCTTCTCACTCCACTTAGCGGGTGTTTCATCTATCCTGGGAGCAATCAACTTCATCACCACAATTATTAACATGAAGCCCCCAGCAATCTCCCAGTACCAAACACCCCTATTCGTCTGATCCGTACTCGTAACTGCCGTTCTCCTTCTCCTGTCACTTCCCGTCTTAGCTGCTGGAATCACAATACTCCTGACAGATCGGAACCTCAACACCACATTCTTTGACCCAGCAGGTGGGGGAGACCCAATCCTTTACCAACACTTATTCTGGTTCTTCGGCCACCCAGAAGTTTACATCCTTATCCTCCCAGGATTTGGGATTATTTCACATGTCGTAGCCTATTATTCCGGTAAAAAAGAACCATTTGGCTACATAGGAATGGTTTGGGCCATAATGGCCATCGGCCTTCTAGGATTTATCGTATGAGCCCATCACATATTTACCGTCGGAATAGACGTAGACACTCGCGCGTATTTTACATCTGCAACAATAATCATCGCAATCCCAACAGGTGTAAAAGTATTTAGCTGACTGGCCACGCTTCACGGAGGGTCAATCAAATGAGAAACACCTATACTGTGAGCCCTGGGGTTCATTTTTCTATTTACAGTAGGCGGACTCACAGGAATCGTCCTGTCTAACTCATCACTTGATATTGTCCTTCACGACACCTATTATGTAGTCGCACATTTCCACTACGTACTATCTATAGGTGCTGTATTTGCTATTATAGCAGCCTTTGTACACTGATTCCCCCTACTAACTGGCTACACCCTCCACAGCGCCTGAACAAAAATTCACTTCGCAGTCATATTTATTGGAGTTAACCTTACATTTTTCCCACAACACTTCCTAGGCCTAGCAGGCATGCCACGACGATACTCCGACTACCCAGATGCCTATGCCCTGTGAAATACAATTTCATCTATTGGTTCACTAATCTCATTGGTAGCGGTAATTATGTTCTTATTTATCCTATGAGAAGCCTTTACCGCTAAACGAGAAGTACTATCTGTAGAACTGACTACGACAAACGTAGAATGACTCCACGGGTGCCCCCCTCCCTACCACACATACGAAGAACCAGCATTTGTTCAAGTTCAATCAAAC

>A._beijiangensis_1

GTGGCAATCACACGCTGATTCTTCTCTACCAATCACAAAGACATTGGCACCCTTTATCTTGTATTCGGTGCCTGAGCCGGAATAGTAGGAACTGCCCTAAGCCTCCTGATCCGGGCCGAGCTTAGTCAACCTGGATCACTTCTTGGTGATGACCAAATTTATAATGTTATCGTAACTGCTCACGCATTCGTTATAATCTTCTTTATAGTAATACCCATCCTCATTGGAGGATTTGGGAATTGACTCGTACCACTAATAATTGGAGCCCCAGACATAGCGTTTCCACGAATAAATAATATAAGCTTCTGGCTACTGCCCCCATCATTCCTGCTTTTATTAGCTTCTTCTGGCGTTGAGGCTGGAGCTGGGACAGGGTGGACAGTCTACCCTCCCCTTGCAGGAAACCTAGCTCACGCAGGAGCATCAGTAGACCTAACAATCTTCTCATTACACTTAGCAGGTGTTTCATCAATCCTTGGAGCAATCAACTTCATCACCACAATTATTAACATGAAACCCCCGGCCATCTCCCAATATCAAACACCCCTGTTCGTTTGATCCGTACTTGTAACTGCCGTTCTTCTTCTCCTATCACTGCCTGTTCTAGCTGCTGGAATTACAATGCTCCTAACAGATCGAAATCTCAACACCACATTCTTTGATCCAGCAGGAGGAGGAGACCCAATCCTCTACCAACACTTATTCTGATTCTTCGGCCACCCAGAAGTCTACATCCTTATTCTCCCAGGGTTCGGAATTATTTCTCACGTTGTAGCCTACTACTCCGGCAAAAAAGAACCGTTCGGCTACATAGGAATAGTCTGAGCTATGATGGCTATCGGCCTCCTGGGGTTTATCGTATGAGCTCACCACATATTTACTGTTGGAATAGACGTAGACACCCGCGCATACTTTACATCTGCAACAATAATTATTGCAATCCCAACAGGTGTAAAAGTGTTTAGCTGACTGGCCACGCTTCACGGGGGATCAATCAAATGAGAAACACCTATACTATGAGCCCTAGGGTTCATTTTCCTGTTTACAGTCGGTGGGCTCACAGGAATTGTCCTATCCAACTCATCACTTGATATTGTTCTTCACGACACTTATTATGTAGTCGCACATTTCCACTATGTACTATCTATGGGGGCTGTATTTGCCATCATAGCAGCCTTTGTACACTGATTCCCGCTACTGACCGGATATACCCTTCACAGCGCCTGAACAAAAATCCATTTTATAGTCATATTCATCGGAGTTAACCTCACATTCTTCCCACAACACTTCCTTGGCCTGGCAGGTATGCCACGACGATATTCTGACTACCCGGATGCCTACGCCCTATGAAACACAGTCTCATCTATTGGATCACTAATCTCACTAGTAGCAGTAGTTATATTCCTATTTATCCTATGAGAAGCCTTCACCGCCAAACGAGAAGTGTTATCTGTAGAACTAACAATAACAAATGTAGAATGACTTCACGGCTGCCCCCCTCCCTATCACACATACGAGGAACCAGCATTCGTTCAAGTTCAATTAAAT

>A._beijiangensis_2

GTGGCAATCACACGCTGATTCTTCTCTACCAATCACAAAGACATTGGCACCCTTTATCTTGTATTCGGTGCCTGAGCCGGAATAGTAGGAACTGCCCTAAGCCTCCTGATTCGGGCCGAGCTTAGCCAGCCTGGATCACTTCTTGGTGATGACCAAATTTATAATGTTATCGTAACTGCTCACGCATTCGTTATAATCTTCTTTATAGTAATACCTATCCTCATTGGGGGATTTGGGAACTGACTCGTGCCACTAATAATTGGAGCCCCAGACATAGCGTTTCCACGAATAAATAATATAAGCTTCTGACTACTGCCCCCATCATTCCTGCTTTTATTAGCTTCTTCTGGCGTTGAGGCTGGAGCTGGGACAGGATGAACAGTCTACCCTCCCCTTGCAGGAAACCTAGCTCACGCAGGAGCATCAGTAGACCTAACAATCTTCTCATTACACTTAGCAGGTGTTTCATCAATCCTTGGGGCAATCAACTTCATCACCACAATTATTAACATGAAACCCCCGGCCATCTCTCAATATCAAACACCCCTGTTCGTTTGATCCGTACTTGTAACTGCCGTTCTTCTTCTCCTATCACTGCCTGTTCTAGCTGCTGGAATTACAATGCTCCTAACAGATCGAAATCTAAACACCACATTCTTTGACCCGGCAGGAGGAGGAGACCCAATCCTCTACCAACATTTATTCTGATTCTTTGGCCACCCAGAAGTCTACATCCTTATTCTCCCAGGGTTCGGAATTATTTCTCACGTTGTAGCCTACTACTCCGGCAAAAAAGAACCGTTCGGCTACATGGGAATAGTCTGAGCTATAATGGCTATCGGCCTCCTGGGGTTTATCGTGTGAGCCCACCACATATTCACTGTTGGAATAGACGTAGATACCCGCGCATACTTTACATCTGCAACAATAATTATTGCAATCCCAACAGGTGTGAAAGTGTTTAGCTGACTGGCCACGCTTCACGGAGGATCAATCAAATGAGAAACACCTATACTATGGGCCCTAGGGTTCATTTTCCTGTTTACAGTCGGTGGACTCACAGGAATTGTCCTATCCAACTCATCACTTGATATTGTTCTTCACGACACTTATTATGTAGTCGCACATTTCCACTATGTATTATCTATGGGGGCTGTATTTGCCATTATAGCAGCCTTTGTGCACTGATTCCCACTACTGACCGGATACACCCTTCACAGCGCCTGAACAAAAATCCATTTTACAATCATGTTCATCGGAGTTAACCTCACATTCTTCCCACAACACTTCCTTGGCCTAGCAGGTATGCCACGACGATATTCTGACTACCCGGATGCCTACGCCCTATGAAACACAGTTTCATCTATTGGATCACTAATCTCACTAGTAGCAGTAATTATATTCCTATTTATCCTATGAGAAGCCTTCACCGCCAAACGAGAAGTGTTATCTGTAGAACTAACAATAACAAATGTAGAATGACTTCACGGCTGCCCCCCTCCCTATCACACATACGAGGAACCAGCATTCGTTCAAGTTCAATTAAAT

>A._fasciatus

GTGGCAATCACGCGCTGATTCTTCTCTACCAACCACAAAGACATTGGTACCCTTTATCTCGTATTTGGTGCCTGAGCCGGAATAGTGGGAACTGCCCTAAGCCTCCTAATTCGGGCCGAGCTTAGTCAACCCGGATCACTTCTCGGTGATGATCAAATTTACAATGTTATCGTAACTGCTCACGCATTTGTAATAATCTTCTTTATAGTAATACCCATCCTCATCGGAGGATTTGGAAATTGACTCGTACCACTAATAATCGGAGCCCCAGACATAGCATTCCCGCGAATAAATAATATAAGCTTCTGACTCCTACCCCCATCATTCCTGCTCTTACTAGCTTCTTCTGGTGTTGAAGCCGGGGCCGGAACAGGATGAACAGTCTACCCACCCCTGGCAGGAAACCTGGCTCATGCAGGAGCATCAGTAGATCTAACAATCTTCTCACTACATTTAGCAGGTGTTTCATCGATCCTCGGGGCAATCAACTTCATCACCACAATTATTAATATGAAACCCCCAGCCATTTCCCAATATCAAACACCTCTGTTCGTCTGATCCGTACTCGTAACTGCCGTTCTTCTCCTCCTGTCATTACCTGTTTTAGCTGCCGGAATTACAATACTCCTAACAGATCGAAACCTCAACACCACATTCTTTGACCCGGCAGGAGGGGGAGACCCAATCCTCTACCAACACCTGTTTTGATTCTTTGGCCATCCAGAAGTTTACATCCTTATCCTCCCAGGATTTGGAATCATCTCTCATGTTGTAGCCTACTACTCTGGTAAAAAGGAACCATTTGGTTACATGGGGATAGTTTGAGCCATAATGGCTATCGGTCTTCTAGGGTTTATCGTATGAGCTCACCATATATTCACCGTTGGAATAGACGTTGACACCCGCGCATACTTTACATCTGCAACAATAATCATTGCAATCCCAACAGGTGTAAAAGTGTTTAGCTGACTAGCCACACTCCACGGAGGATCAATCAAATGAGAAACACCCATACTATGAGCCCTAGGATTCATTTTCCTGTTTACAGTGGGGGGACTCACAGGAATTGTCTTATCTAATTCATCACTTGATATTGTTCTTCATGACACTTATTATGTAGTCGCACATTTCCACTATGTATTATCTATGGGTGCTGTATTTGCCATTATAGCAGCCTTTGTACATTGATTCCCGTTACTAACCGGATACACCCTTCACAGCACCTGAACAAAAATCCACTTCACAGTCATATTTGCTGGGGTAAACCTCACATTCTTCCCACAACACTTCCTAGGCCTAGCGGGTATGCCACGACGATATTCTGACTACCCAGACGCCTACGCCCTATGAAACACAATTTCATCTATTGGATCACTAATCTCATTAGTAGCAGTAATTATGTTCTTATTTATTCTATGAGAAGCCTTCACCGCTAAACGAGAAGTACTATTTGTTGAACTAACAACAACAAATGTAGAGTGACTTCACGGCTGCCCCCCTCCCTACCACACATACGAGGAACCAGCATTCGTTCAAGTTCAATCAAAC

>A._hemispinus

GTGGCAATCACGCGCTGATTCTTCTCTACCAACCACAAAGACATTGGTACCCTCTATCTCGTATTTGGTGCCTGAGCCGGAATAGTAGGAACTGCCCTAAGCCTCCTAATTCGGGCCGAGCTAAGTCAACCCGGATCACTTCTCGGTGATGATCAAATTTACAATGTTATCGTAACTGCTCACGCATTTGTTATAATCTTCTTTATAGTAATACCCATCCTTATCGGGGGATTTGGAAATTGACTCGTGCCACTAATAATTGGAGCCCCCGACATAGCATTTCCACGAATAAATAACATAAGCTTCTGACTACTACCCCCATCATTCCTACTCTTACTAGCTTCTTCCGGCGTTGAAGCTGGGGCTGGGACAGGATGAACAGTCTACCCACCCCTAGCAGGGAACCTAGCCCACGCAGGGGCATCAGTAGATCTAACAATCTTCTCACTACACTTAGCAGGTGTTTCATCAATCCTTGGGGCAATCAACTTCATCACCACAATTATTAATATGAAACCCCCAGCCATCTCCCAATATCAAACACCTCTATTCGTCTGATCCGTACTTGTAACTGCCGTTCTTCTTCTCCTATCACTGCCTGTTTTAGCTGCTGGAATTACAATACTCCTAACAGATCGAAACCTTAACACCACATTCTTTGATCCGGCAGGAGGAGGAGACCCCATCCTTTACCAACACCTATTCTGATTCTTCGGCCATCCGGAGGTTTACATCCTTATCCTCCCAGGATTTGGAATTATCTCCCATGTTGTAGCCTACTACTCAGGTAAAAAGGAACCATTTGGTTACATGGGAATAGTTTGAGCCATAATGGCCATCGGCCTTCTAGGGTTCATCGTATGAGCTCATCACATATTCACCGTTGGAATGGACGTTGACACCCGCGCATACTTTACGTCCGCAACAATAATCATTGCAATCCCAACAGGTGTAAAAGTATTTAGCTGACTAGCCACGCTCCACGGAGGATCAATCAAATGAGAAACACCTATACTATGAGCCCTGGGGTTCATTTTCCTGTTCACAGTAGGTGGGCTCACAGGAATCGTCTTATCTAACTCATCACTTGATATTGTTCTCCATGATACCTATTATGTAGTCGCACATTTCCACTACGTACTATCCATGGGTGCTGTATTCGCCATTATAGCAGCCTTTGTGCACTGATTCCCACTACTAACCGGGTACACCCTTCATAGCACCTGAACAAAAATCCACTTCACAGTCATATTTATTGGAGTTAACCTTACATTCTTCCCACAACATTTCCTAGGCCTAGCGGGCATACCACGACGATACTCTGACTACCCAGATGCCTACGCCCTATGAAACACAATCTCATCTATTGGGTCGCTAATCTCATTAGTAGCAGTAATTATGTTCTTATTTATTCTATGAGAAGCCTTCACCGCTAAACGAGAAGTTCTATCTGTAGAACTAACAACAACGAATGTAGAATGACTCCATGGCTGCCCCCCTCCCTACCACACATACGAAGAACCAGCATTCGTTCAAGTTCAATCAAAC

>A._iridescens

GTGGCAATCACACGCTGATTCTTCTCTACCAACCACAAAGACATTGGTACCCTTTATCTCGTATTTGGTGCCTGAGCCGGAATAGTGGGAACTGCCCTAAGCCTCCTAATTCGGGCCGAGCTAAGTCAACCCGGGTCACTTCTAGGTGATGATCAAATCTATAATGTTATCGTGACCGCCCACGCCTTTGTTATAATTTTCTTTATAGTAATACCTATCCTCATTGGGGGATTCGGAAATTGACTCGTACCGCTGATAATTGGGGCCCCCGATATAGCATTCCCACGAATAAATAATATAAGCTTCTGACTACTACCCCCGTCATTCCTGCTACTGCTAGCCTCTTCTGGTGTTGAAGCAGGAGCCGGAACAGGGTGGACAGTTTACCCGCCCCTTGCAGGAAACCTAGCTCACGCAGGGGCATCAGTAGACCTAACAATCTTCTCACTCCACTTAGCAGGTGTTTCATCCATCCTGGGGGCAATCAACTTCATCACCACAATTATTAACATGAAACCCCCAGCTATCTCCCAGTACCAAACACCTCTATTCGTCTGATCCGTACTTGTAACTGCCGTTCTCCTTCTCCTGTCACTGCCCGTCTTAGCTGCTGGAATCACAATACTTCTAACAGATCGGAACCTCAACACCACATTCTTTGACCCAGCAGGTGGGGGAGATCCAATCCTTTACCAACACTTATTCTGGTTCTTCGGCCACCCAGAAGTTTACATCCTCATCCTCCCAGGATTTGGGATTATTTCACATGTCGTAGCCTATTATTCCGGTAAAAAAGAACCATTTGGTTACATGGGAATGGTTTGGGCCATAATGGCCATCGGCCTTCTAGGATTTATCGTATGGGCCCATCACATGTTCACCGTTGGAATAGACGTAGACACTCGCGCGTATTTTACATCTGCAACAATAATCATCGCAATCCCAACAGGTGTAAAAGTATTTAGCTGACTGGCCACGCTTCACGGAGGGTCAATCAAATGAGAAACACCCATGCTGTGAGCCCTGGGGTTCATTTTTCTATTCACAGTAGGCGGACTCACAGGAATCGTCCTGTCTAACTCATCACTTGACATTGTCCTTCACGACACTTATTATGTAGTCGCACATTTCCACTACGTACTATCCATAGGTGCTGTATTTGCTATTATAGCAGCCTTTGTACACTGATTCCCCCTACTAACTGGCTACACCCTCCACAGCGCCTGAACAAAAATTCACTTCGCAGTTATGTTTATTGGAGTTAACCTTACATTTTTCCCACAACACTTCCTAGGCCTAGCAGGCATGCCACGACGATACTCCGACTACCCAGATGCCTATGCCCTGTGAAATACAATTTCATCTATTGGTTCGCTAATCTCATTAGTAGCGGTAATTATGTTCTTATTTATTTTATGAGAAGCCTTTACCGCTAAACGAGAAGTACTATCTGTAGAACTGACTACAACAAACGTAGAATGACTCCACGGATGCCCCCCTCCCTACCACACATACGAAGAACCAGCATTTGTTCAAGTTCAATCAAAC

>A._jishouensis

GTGGCAATCACACGCTGATTCTTCTCTACCAACCACAAAGACATTGGTACCCTTTATCTCGTATTTGGTGCCTGAGCCGGAATAGTGGGAACTGCCCTAAGCCTCCTAATTCGGGCCGAGCTAAGTCAACCTGGATCACTTCTCGGTGATGATCAAATTTACAATGTTATCGTAACTGCCCACGCATTTGTTATAATCTTCTTTATAGTAATACCCATCCTTATTGGGGGATTTGGAAACTGACTCGTACCACTAATAATTGGAGCCCCCGACATGGCATTCCCACGAATAAATAACATAAGCTTCTGATTACTACCCCCATCATTCCTGCTTTTACTAGCTTCTTCCGGTGTTGAGGCTGGAGCCGGGACAGGGTGAACAGTCTACCCACCCCTAGCAGGAAACCTAGCCCACGCAGGGGCATCAGTAGACTTAACAATCTTCTCACTTCATTTAGCAGGTGTTTCATCAATCCTCGGAGCAATCAACTTCATCACCACAATTATTAATATGAAACCCCCAGCTATCTCCCAGTATCAAACACCTCTATTCGTCTGATCCGTACTTGTAACTGCCGTTCTTCTTCTCCTGTCGCTGCCTGTTTTAGCTGCTGGGATTACAATACTCCTAACAGATCGAAACCTCAACACCACATTCTTTGACCCAGCAGGGGGAGGAGACCCCATCCTTTACCAACACCTATTCTGATTCTTTGGTCATCCAGAAGTTTACATCCTTATCCTCCCAGGATTTGGAATTATCTCTCATGTTGTAGCCTACTACTCCGGCAAAAAGGAACCATTTGGTTACATGGGAATAGTTTGAGCCATAATGGCCATCGGTCTTCTAGGGTTTATCGTATGAGCTCACCACATATTCACCGTTGGAATAGACGTTGATACCCGTGCATACTTTACATCTGCAACAATAATCATTGCAATCCCAACAGGTGTAAAAGTGTTTAGTTGACTAGCCACGCTCCACGGAGGATCAATCAAATGAGAAACACCTATACTATGAGCCCTGGGGTTCATTTTCCTGTTTACAGTAGGCGGACTCACAGGAATTGTCCTATCTAATTCATCACTTGATATTGTTCTTCATGACACTTATTATGTAGTCGCACATTTCCACTACGTACTATCCATGGGTGCTGTATTCGCCATTATGGCAGCCTTTGTGCACTGATTCCCACTACTAACCGGATACACCCTCCACAGCACCTGAACAAAAATCCACTTCACAGTTATATTTATTGGAGTTAACCTTACATTCTTCCCACAACACTTCCTAGGCCTAGCGGGCATGCCACGGCGATATTCTGACTACCCAGATGCCTACGCCCTATGAAATACAATCTCATCTATTGGATCGCTAATCTCATTAGTGGCAGTAATTATATTCTTATTTATCCTATGAGAAGCCTTCACCGCTAAACGAGAAGTACTATCTGTAGAACTAACAACAACAAATGTAGAATGACTTCACGGCTGCCCCCCTCCCTACCATACATACGAAGAACCAGCATTCGTTCAAGTTCAATCAAAC

>A._kreyenbergii_1

GTGGCAATCACGCGCTGATTCTTCTCTACCAACCACAAAGACATTGGTACCCTTTATCTAGTATTTGGTGCCTGAGCCGGAATAGTAGGAACCGCTTTAAGCCTCCTCATCCGAGCTGAACTTAGTCAACCCGGATCACTTCTAGGTGATGACCAAATTTACAATGTAATTGTTACCGCCCACGCCTTCGTAATAATTTTCTTTATAGTAATGCCTATCCTCATTGGAGGATTCGGAAACTGACTTGTACCCCTGATAATCGGAGCCCCAGACATGGCATTCCCACGAATAAATAATATAAGCTTCTGACTTCTTCCCCCATCATTCCTGTTACTACTAGCTTCCTCTGGTGTTGAAGCCGGAGCTGGCACCGGATGGACAGTATACCCCCCTCTTGCAGGGAACCTGGCCCACGCAGGAGCATCAGTAGACCTAACAATTTTCTCACTACATTTAGCAGGTGTTTCATCAATCCTGGGGGCAATCAACTTCATTACTACAACCATTAACATAAAACCTCCAGCCATTTCCCAATACCAAACACCCCTATTTGTTTGATCCGTACTTGTAACCGCCGTCCTCCTTCTCCTATCACTACCTGTTCTAGCTGCCGGTATTACAATGCTTTTAACAGATCGAAATCTCAACACCACATTCTTTGATCCAGCAGCGGGAGGAGACCCAATTCTCTATCAACACTTATTCTGATTCTTTGGTCACCCAGAAGTCTACATTTTAATCCTTCCAGGATTTGGAATCATCTCTCACGTTGTAGCCTATTATTCAGGTAAAAAAGAACCATTTGGTTATATAGGAATAGTATGAGCCATAATGGCCATTGGCCTCCTAGGGTTCATTGTATGAGCCCACCATATGTTTACTGTCGGAATGGACGTAGACACCCGTGCATATTTTACATCCGCAACAATAATCATCGCAATTCCAACGGGTGTAAAAGTATTTAGCTGACTGGCTACACTTCACGGAGGATCAATTAAATGAGAAACACCAATACTATGAGCCCTAGGATTCATTTTCCTGTTTACAGTGGGAGGACTTACAGGAATTGTCCTCTCTAATTCATCACTTGATATTGTTCTCCACGACACCTATTATGTAGTAGCACATTTCCACTATGTACTATCAATGGGTGCCGTATTCGCAATTATGGCAGCCTTTGTACACTGATTCCCCCTACTAACAGGGTACACTCTACATAGCGCTTGAACAAAAATCCACTTTGGGGTTATATTTATTGGAGTTAACCTCACATTCTTCCCACAACACTTCCTGGGTCTAGCAGGAATACCACGACGGTATTCTGATTATCCAGACGCTTATGCCCTATGAAATACAGTATCATCTATCGGATCCCTAATCTCCCTAGTAGCGGTAATTATGTTCCTATTTATTCTATGAGAAGCCTTCGCCGCTAAACGAGAAGTGTTATCTGTAGAACTAACAATAACAAATGTGGAATGACTCCATGGCTGCCCCCCTCCTTACCACACATACGAGGAACCAGCATTTGTTCAAATTCAATCAAAT

>A._kreyenbergii_2

GTGGCAATCACGCGCTGATTCTTTTCTACTAACCACAAAGACATTGGTACCCTTTATCTAGTATTTGGTGCCTGAGCCGGAATAGTGGGAACTGCCTTAAGCCTCCTAATTCGGGCCGAGCTTAGTCAACCCGGATCACTTCTCGGTGATGATCAAATTTACAATGTTATCGTAACTGCTCACGCATTTGTTATAATCTTCTTTATAGTAATACCCATCCTCATCGGGGGATTTGGAAATTGACTTGTGCCTCTAATAATCGGAGCCCCGGACATAGCATTCCCGCGAATAAATAACATAAGCTTCTGATTACTACCCCCATCATTCCTGCTTTTATTAGCTTCTTCTGGTGTTGAAGCTGGGGCAGGAACAGGGTGGACAGTCTACCCGCCCCTAGCAGGAAACCTGGCCCATGCAGGAGCATCAGTAGATCTAACAATCTTCTCACTACATTTAGCAGGTGTTTCATCAATCCTTGGGGCGATCAACTTCATCACTACAATTATTAACATGAAACCCCCAGCCATCTCCCAATACCAAACACCCCTGTTCGTCTGATCCGTACTTGTAACTGCTGTTCTTCTTCTCCTGTCACTACCTGTTTTAGCCGCCGGAATTACAATACTCCTAACAGATCGAAACCTCAACACCACATTCTTTGATCCGGCAGGAGGAGGAGACCCTATCCTCTACCAACACTTATTCTGATTCTTTGGTCACCCAGAAGTTTACATCCTTATCCTCCCAGGATTTGGAATCATCTCTCATGTTGTAGCCTATTACTCGGGTAAAAAAGAACCATTTGGTTATATAGGAATAGTTTGAGCCATAATGGCCATTGGTCTTCTAGGATTTATCGTGTGAGCTCATCATATATTTACCGTTGGAATAGACGTTGATACCCGCGCATACTTTACATCTGCAACAATAATCATTGCAATCCCAACAGGTGTAAAAGTGTTTAGCTGACTAGCCACACTCCACGGAGGATCAATCAAATGAGAAACACCCATACTATGAGCCCTGGGATTTATTTTCCTGTTTACAGTAGGGGGACTCACAGGAATTGTCCTATCTAATTCATCACTTGATATTGTTCTTCATGACACTTATTATGTAGTCGCACATTTCCACTATGTATTATCTATGGGTGCTGTATTTGCCATTATAGCAGCCTTTGTACACTGATTCCCATTACTAACCGGATATACCCTTCACAGCACTTGAACAAAAATCCACTTCACAGTTATATTTGCTGGAGTTAACCTTACATTCTTCCCACAACACTTCCTAGGCCTAGCAGGCATGCCACGACGATATTCTGACTACCCAGATGCCTACGCCCTATGAAACACAATTTCATCTATTGGATCACTAATCTCATTAGTAGCAGTAATTATGTTCTTATTCATTCTATGAGAAGCCTTCACCGCTAAACGAGAAGTACTATTTGTCGAACTAACAACAACAAATGTAGAGTGACTTCACGGCTGCCCCCCTCCCTACCACACATACGAGGAACCAGCATTCGTTCAAGTTCAATCAAAC

>A._longipinnis

GTGGCAATCACACGCTGATTCTTCTCTACCAACCACAAAGACATTGGTACCCTTTATCTCGTATTTGGTGCCTGAGCCGGAATAGTGGGAACTGCCCTAAGCCTCCTAATTCGGGCCGAGCTAAGTCAACCCGGGTCACTTCTAGGTGATGATCAAATCTATAATGTTATCGTGACCGCCCACGCTTTTGTTATAATTTTCTTTATAGTAATACCTATCCTCATTGGGGGATTCGGAAATTGACTCGTACCGCTAATAATTGGGGCCCCCGATATAGCATTCCCACGAATAAATAATATAAGCTTCTGACTACTACCCCCATCATTCCTGCTACTGCTAGCCTCTTCTGGTGTTGAAGCAGGAGCCGGAACAGGGTGAACAGTTTACCCGCCCCTTGCAGGAAACCTAGCTCACGCAGGGGCATCAGTAGACCTAACAATCTTCTCACTCCACTTAGCAGGTGTTTCATCTATCCTGGGAGCAATCAACTTCATCACCACAATTATTAACATGAAGCCCCCAGCTATCTCCCAGTACCAAACACCTCTATTCGTCTGATCCGTACTTGTAACTGCCGTTCTCCTTCTCCTGTCACTGCCCGTCTTAGCTGCTGGAATCACAATACTTCTAACAGATCGGAACCTCAACACCACATTCTTTGACCCAGCAGGTGGGGGAGATCCAATCCTTTACCAACACTTATTCTGGTTCTTCGGCCACCCAGAAGTTTACATCCTCATCCTCCCAGGATTTGGGATTATTTCACATGTCGTAGCCTATTATTCCGGTAAAAAAGAACCATTTGGTTACATGGGAATGGTTTGGGCCATAATGGCCATCGGCCTTCTAGGATTTATCGTATGGGCCCATCACATGTTCACCGTTGGAATAGACGTAGACACTCGCGCGTATTTTACATCTGCAACAATAATCATCGCAATCCCAACAGGTGTAAAAGTATTTAGCTGACTGGCCACGCTTCACGGAGGGTCAATCAAATGAGAAACACCCATACTGTGAGCCCTGGGGTTCATTTTTCTATTCACAGTAGGCGGACTCACAGGAATCGTCCTGTCTAACTCATCACTTGACATTGTCCTTCACGACACTTATTATGTAGTCGCACATTTCCACTACGTACTATCCATAGGTGCTGTATTTGCTATTATAGCAGCCTTTGTACACTGATTCCCCCTACTAACTGGCTACACCCTCCACAGCGCCTGAACAAAAATTCACTTCGCAGTCATGTTTATTGGAGTTAACCTTACATTTTTCCCACAACACTTCCTAGGCCTAGCAGGCATGCCACGACGATACTCCGACTACCCAGATGCCTATGCCCTGTGAAATACAATTTCATCTATTGGTTCGCTAATCTCATTAGTAGCGGTAATTATGTTCTTATTTATTCTATGAGAAGCCTTTACTGCTAAACGAGAAGTACTATCTGTAGAACTGACTACAACAAACGTAGAATGACTCCACGGATGCCCCCCTCCCTACCACACATACGAAGAACCAGCATTTGTTCAAGTTCAATCAAAC

>A._monticola_1

GTGGCAATCACGCGCTGATTCTTCTCTACTAACCAGAAAGACATTGGTACCCTCTATCTGGTATTTGGTGCGTGAGCCGGTATAGTGGGTACCGCCCTAAGCCTCCTAATTCGGGCTGAACTAAGTCAACCCGGGTCGCTTTTAGGCGATGACCAAATTTACAATGTTATTGTTACTGCCCACGCCTTTGTTATAATTTTGTTTATAGTAATGCCTATCCTTATTGGAGGTTTTGGAAAGTGGCTCGTACCTCTAATGATTGGAGCCCCAGATATGGCATTCCCACGAATGAACAACATAAGTTTCTGACTACTGCCCCCATCATTCCTGCTACTACTAGCCTCTTCTGGTGTTGAAGCTGGAGCCGGGACAGGATGAACAGTCTACCCACCCCTTGCAGGAAACCTAGCCCACGCAGGTGCATCAGTAGACCTAACAATTTTCTCGCTGCACTTAGCAGGTGTCTCATCAATCCTTGGCGCAATTAACTTCATCACTACAATTATTAACATGAAACCCCCAGCCATCTCTCAATACCATACACCTCTGTTCGTCTGATCCGTGCTTGTGACCGCTGTACTCCTTCTCCTATCACTTCCAGTCCTAGCCGCTGGGATCACTATACTCCTTACAGATCGAAACCTCAACACCACATTCTTCGATCCGGCAGGGGGAGGGGACCCAATCCTCTACCAACATCTATTCTGATTCTTCGGTCACCCAGAAGTCTACATCCTCATCCTCCCGGGGTTCGGAATTATTTCTCACGTCGTAGCCTACTATTCCGGTAAAAAAGAACCATTTGGCTACATAGGGATAGTTTGAGCTATAATAGCCATTGGCCTCCTAGGGTTTATCGTATGGGCCCACCACATATTTACCGTCGGAATAGACGTGGACACTCGTGCATACTTTACATCTGCAACAATAATCATCGCAATCCCAACAGGTGTTAAAGTCTTCAGCTGACTGGCCACACTCCACGGAGGGTCAATCAAATGAGAAACACCTTTACTATGAGCCCTGGGATTCATCTTCCTATTTACAGTGGGTGGACTCACAGGAATTGTCCTGTCCAATTCATCACTTGACATCGTTCTTCACGACACTTATTATGTAGTTGCACATTTCCACTACGTATTATCTATAGGCGCTGTATTTGCTATTATAGCAGCCTTCGTTCACTGATTCCCCCTATTAACTGGGTACACCCTCCATAGCGCCTGAACAAAAATCCACTTCGCAGTCATATTTATTGGGGTTAACCTTACGTTCTTCCCACAACACTTCCTCGGACTAGCGGGCATGCCACGACGATACTCTGACTACCCAGACGCCTATGCTCTATGAAACACAATATCATCTATCGGATCACTAATTTCATTAGTGGCGGTAATCATATTCTTATTTATTCTATGAGAAGCCTTCACCGCTAAACGTGAAGTACTATCTGTAGAACTAACAACAACAAACGTAGAATGACTCCACGGCTGCCCCCCACCCTACCACACATACGAGGAACCAGCATTCGTTCAAGTTCAATCAAAC

>A._monticola_2

GTGGCAATCACGCGCTGATTCTTCTCTACTAACCACAAAGACATTGGTACCCTCTATCTCGTATTTGGTGCCTGAGCCGGAATAGTGGGAACCGCCCTAAGCCTCCTAATTCGGGCTGAACTAAGTCAACCCGGGTCGCTTTTAGGCGATGACCAAATTTACAATGTTATTGTTACTGCCCACGCCTTTGTTATAATTTTCTTTATAGTAATGCCTATCCTTATTGGAGGATTTGGAAACTGGCTCGTACCACTAATGATTGGAGCCCCAGATATGGCATTCCCACGAATGAACAACATAAGTTTCTGACTACTGCCCCCATCATTCCTGCTACTACTAGCCTCTTCTGGTGTTGAAGCTGGAGCCGGGACAGGATGAACAGTCTACCCACCCCTTGCAGGAAACCTAGCCCACGCAGGAGCATCAGTAGACCTAACAATTTTCTCGCTCCACTTAGCAGGTGTCTCATCAATCCTTGGGGCAATTAACTTCATCACTACAATTATTAACATGAAACCCCCAGCCATCTCTCAATACCAAACACCTCTGTTCGTCTGATCCGTGCTTGTCACCGCTGTACTCCTTCTCCTATCACTTCCTGTCCTAGCCGCTGGGATCACAATACTCCTAACAGATCGAAACCTCAACACCACATTCTTTGATCCGGCAGGGGGAGGGGACCCAATCCTCTACCAACATCTATTCTGATTCTTCGGTCACCCAGAAGTCTACATCCTCATCCTCCCGGGGTTCGGAATTATTTCTCACGTCGTAGCCTACTATTCCGGTAAAAAAGAACCATTTGGCTACATAGGGATAGTTTGAGCTATAATAGCCATTGGCCTCCTAGGGTTTATCGTATGGGCCCACCACATATTTACCGTCGGAATAGACGTGGACACTCGTGCATACTTTACATCTGCAACAATAATCATCGCAATCCCAACAGGTGTTAAAGTCTTCAGCTGACTGGCCACACTCCACGGAGGGTCAATCAAATGAGAAACACCTTTACTATGAGCCCTGGGATTCATCTTCCTATTTACAGTGGGTGGACTCACAGGAATTGTCCTGTCCAATTCATCACTTGACATCGTTCTTCACGACACTTATTATGTAGTTGCACATTTCCACTACGTATTATCTATAGGCGCTGTATTTGCTATTATAGCAGCCTTCGTTCACTGATTCCCCCTATTAACTGGGTACACCCTCCATAGCGCCTGAACAAAAATCCACTTCGCAGTCATATTTATTGGGGTTAACCTTACGTTCTTCCCACAACACTTCCTCGGACTAGCGGGCATGCCACGACGATACTCTGACTACCCAGACGCCTATGCTCTATGAAACACAATATCATCTATCGGATCACTAATTTCATTAGTGGCGGTAATCATATTCTTATTTATTCTATGAGAAGCCTTCACCGCTAAACGAGAAGTACTATCTGTAGAACTAACAACAACAAACGTAGAATGACTCCACGGCTGCCCCCCTCCCTACCACACATACGAGGAACCAGCATTCGTTCAAGTTCAATCAAAC

>A._paradoxus_1

GTGGCAATCACACGCTGATTCTTCTCTACCAACCACAAAGACATTGGCACCCTTTATCTCGTATTTGGTGCCTGAGCCGGAATAGTAGGAACTGCCCTAAGCCTTCTGATTCGAGCCGAGCTTAGTCAGCCTGGATCGCTTCTTGGTGATGATCAAATTTATAATGTTATCGTAACTGCTCACGCATTTGTTATAATCTTCTTTATAGTAATACCCATCCTTATTGGAGGATTTGGAAATTGACTCGTGCCACTAATAATTGGAGCCCCAGACATAGCATTTCCACGAATAAATAACATAAGCTTCTGACTACTGCCCCCATCATTCCTGCTTTTATTAGCTTCTTCTGGTGTTGAAGCTGGAGCCGGGACAGGGTGAACAGTCTATCCACCCCTTGCAGGAAACCTGGCTCACGCAGGGGCATCAGTAGACCTAACAATCTTCTCGTTACACTTAGCAGGTGTCTCATCAATCCTTGGGGCAATCAACTTCATCACCACAATTATTAACATGAAACCCCCAGCTATTTCCCAATATCAAACGCCCCTGTTTGTCTGATCCGTACTTGTAACTGCCGTTCTTCTTCTCCTTTCACTGCCCGTCTTAGCTGCCGGCATTACAATACTCCTAACAGATCGAAATCTCAACACCACATTCTTTGATCCCGCAGGAGGAGGAGACCCAATCCTTTACCAACACTTATTCTGATTCTTCGGCCACCCAGAAGTTTATATCCTCATCCTCCCAGGATTCGGAATTATTTCTCATGTTGTAGCCTACTACTCCGGTAAAAAAGAGCCGTTTGGCTACATAGGAATAGTTTGAGCCATGATGGCCATCGGCCTTCTAGGATTTATCGTATGAGCTCACCACATATTCACCGTTGGAATAGACGTAGATACTCGCGCATACTTTACATCTGCAACAATAATCATTGCAATCCCAACAGGTGTAAAAGTGTTTAGCTGACTGGCTACGCTTCACGGAGGATCAATCAAATGAGAAACACCCATACTATGAGCCCTAGGGTTCATTTTCTTATTTACAGTCGGTGGGCTTACAGGAATCGTCCTATCCAACTCGTCACTCGACATTGTTCTTCACGACACTTATTATGTAGTTGCACATTTCCATTACGTACTATCTATAGGCGCTGTATTTGCCATCATAGCAGCCTTCGTACACTGATTCCCGCTACTAACCGGATACACCCTCCACAGTACCTGAACAAAAATCCATTTTACAGTCATGTTTATTGGAGTCAACCTCACATTCTTCCCACAACACTTCCTTGGTTTAGCAGGTATGCCACGGCGATATTCTGACTACCCAGATGCCTACGCCCTGTGAAACACAGTCTCATCTTTTGGGTCACTAATCTCATTAGTAGCAGTAGTTATATTCTTATTCATCCTATGAGAAGCCTTCACCGCCAAACGAGAAGTATTATCTGTCGAACTAACAATAACAAATGTAGAATGACTTCACGGCTGCCCCCCTCCCTATCACACATACGAGGAACCAGCATTTGTACAAGTTCAATCAAAT

>A._paradoxus_2

GTGGCAATCACACGCTGATTCTTCTCTACCAACCACAAAGACATTGGTACCCTTTATCTCGTATTTGGTGCCTGAGCCGGAATAGTGGGAACTGCCCTAAGCCTCCTAATTCGGGCCGAGCTAAGTCAACCTGGATCACTTCTCGGTGATGATCAAATTTACAATGTTATCGTAACTGCCCACGCATTTGTTATAATCTTCTTTATAGTAATACCCATCCTTATTGGGGGATTTGGAAACTGACTCGTACCACTAATAATTGGAGCCCCCGACATGGCATTCCCACGAATAAATAACATAAGCTTCTGATTACTACCCCCATCATTCCTGCTTTTACTAGCTTCTTCCGGTGTTGAGGCTGGAGCCGGGACAGGGTGAACAGTCTACCCACCCCTAGCAGGAAACCTAGCCCACGCAGGGGCATCAGTAGACTTAACAATCTTCTCACTTCATTTAGCAGGTGTTTCATCAATCCTCGGAGCAATCAACTTCATCACCACAATTATTAATATGAAACCCCCAGCTATCTCCCAGTATCAAACACCTCTATTCGTCTGATCCGTACTTGTAACTGCCGTTCTTCTTCTCCTGTCGCTGCCTGTTTTAGCTGCTGGGATTACAATACTCCTAACAGATCGAAACCTCAACACCACATTCTTTGACCCGGCAGGGGGAGGAGACCCCATCCTTTACCAACACCTATTCTGATTCTTTGGTCATCCAGAAGTTTACATCCTTATCCTCCCAGGATTTGGAATTATCTCTCATGTTGTAGCCTACTACTCCGGCAAAAAGGAACCATTTGGTTACATGGGAATAGTTTGAGCCATAATGGCCATCGGTCTTCTAGGGTTTATCGTATGAGCTCACCACATATTCACCGTTGGAATAGACGTTGATACCCGTGCATACTTTACATCTGCAACAATAATCATTGCAATCCCAACAGGTGTAAAAGTGTTTAGTTGACTAGCCACGCTCCACGGAGGATCAATCAAATGAGAAACACCTATACTATGAGCCCTGGGGTTCATTTTCCTGTTTACAGTAGGCGGACTCACAGGAATTGTCCTATCTAACTCATCACTTGATATTGTTCTTCATGACACTTATTATGTAGTCGCACATTTCCACTACGTACTATCCATGGGTGCTGTATTCGCCATTATGGCAGCCTTTGTGCACTGATTCCCACTACTAACCGGATACACCCTCCACAGCACCTGAACAAAAATCCACTTCACAGTTATATTTATTGGAGTTAACCTTACATTCTTCCCACAACACTTCCTAGGCCTAGCGGGCATGCCACGGCGATATTCTGACTACCCAGATGCCTACGCCCTATGAAATACAATCTCATCTATTGGATCGCTAATCTCATTAGTGGCAGTAATTATATTCTTATTTATCCTATGAGAAGCCTTCACCGCTAAACGAGAAGTACTATCTGTAGAACTAACAACAACAAATGTAGAATGACTTCACGGCTGCCCCCCTCCCTACCATACATACGAAGAACCAGCATTCGTTCAAGTTCAATCAAAC

>A._parallens_1

GTGGCAATCACGCGCTGATTCTTCTCTACCAACCACAAAGACATTGGTACCCTCTATCTCGTATTTGGTGCCTGAGCCGGAATAGTAGGAACTGCCCTAAGCCTTCTAATTCGGGCCGAGCTAAGTCAACCCGGATCACTTCTCGGTGATGATCAAATTTACAATGTTATCGTAACTGCTCACGCATTTGTTATAATCTTCTTTATAGTAATACCCATCCTTATTGGGGGATTTGGAAATTGACTCGTACCACTAATAATCGGAGCCCCCGACATAGCATTTCCACGAATAAATAACATAAGCTTCTGACTACTGCCCCCATCATTCCTGCTCTTACTAGCTTCTTCCGGCGTTGAAGCTGGGGCTGGGACAGGATGAACAGTCTACCCACCCCTAGCAGGAAACCTGGCCCACGCAGGGGCATCAGTAGATCTAACAATCTTCTCATTACACTTAGCAGGTGTTTCGTCAATCCTTGGGGCAATCAACTTCATCACCACAATTATTAATATGAAACCCCCAGCCATCTCCCAGTATCAAACACCTCTATTCGTCTGATCCGTACTTGTAACTGCCGTTCTTCTTCTCCTGTCACTGCCTGTTTTAGCTGCTGGAATTACAATACTCCTAACAGATCGAAACCTTAACACCACATTCTTTGATCCGGCAGGAGGAGGAGACCCCATCCTCTACCAACACCTATTCTGATTCTTCGGACACCCGGAAGTTTACATCCTCATCCTCCCAGGATTTGGAATTATCTCCCATGTTGTAGCCTATTACTCAGGTAAAAAGGAACCATTTGGTTACATGGGAATAGTTTGAGCCATAATGGCCATCGGCCTTCTAGGGTTTATCGTATGAGCTCATCACATATTCACCGTTGGGATAGACGTTGACACCCGCGCATACTTTACGTCTGCAACAATAATCATTGCAATCCCAACAGGTGTGAAAGTGTTTAGCTGACTAGCCACGCTCCACGGAGGATCAATCAAATGAGAAACACCTATACTATGAGCCCTGGGGTTCATTTTCCTGTTCACAGTAGGTGGGCTCACAGGAATCGTCTTATCTAATTCATCACTTGATATTGTTCTCCACGACACCTACTATGTAGTCGCACATTTCCACTATGTACTATCCATGGGTGCTGTATTCGCCATTATAGCAGCCTTTGTGCACTGATTCCCACTACTAACCGGGTACACCCTTCATAGCACCTGAACAAAAATCCACTTCACAGTTATATTTATTGGAGTTAACCTTACATTCTTCCCACAACACTTCCTAGGCCTAGCGGGCATGCCACGACGATATTCTGACTACCCAGATGCCTACGCCCTATGAAACACAATCTCATCTATCGGATCGCTAATCTCATTAGTAGCAGTAATTATGTTCTTATTTATTCTATGAGAAGCCTTCACCGCTAAACGAGAAGTTCTATCTGTAGAACTAACAACAACAAATGTAGAATGACTCCATGGCTGCCCCCCTCCCTACCACACATACGAAGAGCCAGCATTCGTTCAAGTTCAATCAAAC

>A._parallens_2

GTGGCAATCACGCGCTGATTCTTCTCTACCAACCACAAAGACATTGGTACCCTCTATCTCGTATTTGGTGCCTGAGCCGGAATAGTAGGAACTGCTCTAAGCCTCCTAATTCGGGCCGAGCTAAGTCAACCCGGATCACTTCTCGGTGATGATCAAATTTACAATGTCATCGTAACTGCTCACGCATTTGTTATAATCTTCTTTATAGTAATACCCATCCTTATTGGGGGATTTGGAAATTGACTCGTGCCACTTATAATCGGGGCCCCTGACATAGCATTTCCACGAATAAATAACATAAGCTTCTGACTACTACCCCCATCATTCCTGCTCTTACTAGCTTCTTCCGGCGTTGAAGCTGGGGCTGGGACAGGATGAACAGTCTACCCACCCCTAGCAGGAAACCTAGCCCACGCAGGGGCATCAGTAGATCTAACAATCTTCTCATTACACTTAGCAGGTGTTTCATCAATCCTTGGGGCAATCAACTTCATCACCACAATTATTAATATGAAGCCCCCAGCCATCTCCCAGTACCAAACACCTCTATTCGTCTGATCCGTACTTGTGACTGCCGTTCTTCTTCTCCTGTCACTGCCTGTTCTAGCTGCTGGAATTACAATACTCCTAACAGATCGAAACCTTAACACCACATTCTTTGACCCGGCAGGAGGAGGAGACCCCATCCTTTACCAACACCTATTCTGATTCTTCGGCCACCCGGAAGTTTACATCCTCATCCTCCCAGGATTTGGAATCATCTCCCACGTTGTAGCCTACTACTCAGGTAAAAAGGAACCATTTGGTTACATGGGAATAGTATGAGCCATAATGGCCATCGGCCTTCTAGGATTTATCGTATGAGCTCATCACATGTTTACCGTTGGAATAGACGTTGACACCCGCGCATACTTTACGTCTGCAACAATAATCATTGCAATCCCAACAGGTGTAAAAGTGTTTAGCTGACTAGCCACGCTCCACGGAGGATCAATCAAATGAGAAACACCCATACTATGAGCCCTGGGGTTCATTTTCCTGTTCACAGTAGGCGGACTCACAGGAATCGTCTTATCTAATTCATCACTCGATATTGTTCTCCATGACACCTATTATGTAGTCGCACATTTCCACTATGTATTATCCATGGGTGCTGTATTCGCCATTATAGCAGCCTTTGTGCACTGATTCCCACTACTAACCGGGTACACCCTTCATAGCACCTGAACAAAAATCCACTTCACAGTTATATTTATTGGAGTTAACCTTACATTCTTCCCACAACACTTCCTAGGCCTAGCAGGCATACCACGACGATATTCTGACTACCCAGATGCCTACGCCCTATGAAACACAATCTCATCTATTGGATCATTAATCTCATTAGTAGCAGTAATTATGTTCTTATTTATTTTATGAGAAGCCTTCACCGCTAAACGAGAAGTACTATCTGTAGAACTAACAACAACAAATGTAGAATGACTCCACGGCTGCCCCCCTCCCTACCACACATACGAAGAACCAGCATTCGTTCAAGTTCAATCAAAC

>A._parallens_3

GTGGCAATCACGCGCTGATTCTTCTCTACCAACCACAAAGACATTGGTACCCTCTATCTCGTATTTGGTGCCTGAGCCGGAATAGTAGGAACTGCCCTAAGCCTTCTAATTCGGGCCGAGCTAAGTCAACCCGGATCACTTCTCGGTGATGATCAAATTTACAATGTTATCGTAACTGCTCACGCATTTGTTATAATCTTCTTTATAGTAATACCCATCCTTATTGGGGGATTTGGAAATTGACTCGTGCCACTAATAATCGGAGCCCCCGACATAGCATTTCCACGAATAAATAACATAAGCTTCTGACTACTACCCCCATCATTCCTGCTTTTACTAGCTTCTTCCGGCGTTGAGGCTGGAGCTGGGACAGGGTGAACAGTCTACCCACCCCTAGCAGGAAACCTAGCCCACGCAGGGGCATCAGTAGATCTAACAATCTTCTCATTACACTTAGCAGGTGTTTCGTCAATCCTTGGGGCAATCAACTTTATCACCACAATTATTAATATGAAACCCCCAGCCATCTCCCAGTATCAAACACCTCTATTCGTCTGATCCGTACTTGTAACTGCCGTTCTTCTTCTCCTGTCACTGCCTGTTTTAGCTGCTGGAATTACAATACTCCTAACGGATCGAAACCTTAACACCACATTCTTTGACCCGGCAGGAGGAGGAGACCCCATCCTTTACCAACACCTATTCTGATTCTTCGGCCACCCGGAAGTTTACATCCTCATCCTCCCAGGATTTGGAATTATCTCCCATGTTGTAGCCTACTACTCAGGTAAAAAGGAACCATTTGGTTACATGGGAATAGTTTGAGCCATAATGGCCATCGGCCTTCTAGGGTTTATCGTATGAGCTCATCACATATTCACCGTTGGAATAGACGTTGACACCCGCGCATACTTTACGTCTGCAACAATAATCATTGCAATCCCAACAGGTGTAAAAGTGTTTAGCTGACTAGCCACGCTCCACGGAGGATCAATCAAATGAGAAACACCTATACTATGAGCCCTGGGGTTCATTTTCCTGTTCACAGTAGGTGGACTCACAGGAATCGTCTTATCTAATTCATCACTTGATATTGTTCTCCATGACACCTATTATGTAGTCGCACATTTCCACTATGTACTATCCATGGGTGCTGTATTCGCCATTATAGCAGCCTTTGTGCACTGATTCCCACTACTAACCGGGTACACCCTTCATAGCACCTGAACAAAAATCCACTTCACAGTTATATTTATTGGAGTTAACCTTACATTCTTCCCACAACACTTCCTAGGCCTAGCGGGCATGCCACGACGATATTCTGACTACCCAGATGCCTACGCCCTATGAAACACAATCTCATCTATCGGGTCGCTAATCTCATTAGTAGCAGTAATTATGTTCTTATTCATTCTATGAGAAGCCTTCACCGCTAAACGAGAAGTTCTATCTGTAGAACTAACAACAACAAATGTAGAATGACTCCATGGCTGCCCCCCTCCCTACCACACATACGAAGAACCAGCATTCGTTCAAGTTCAATCAAAC

>A._parallens_4

GTGGCAATCACGCGCTGATTCTTCTCTACCAACCACAAAGACATTGGTACCCTCTATCTCGTATTTGGTGCCTGAGCCGGAATAGTAGGAACTGCCCTAAGCCTTCTAATTCGGGCCGAGCTAAGTCAACCCGGATCACTTCTCGGTGATGATCAAATTTACAATGTTATCGTAACTGCTCACGCATTTGTTATAATCTTCTTTATAGTAATACCCATCCTTATTGGGGGATTTGGAAATTGACTCGTGCCACTAATAATCGGAGCCCCCGACATAGCATTTCCACGAATAAATAACATAAGCTTCTGACTACTACCCCCATCATTCCTGCTTTTACTAGCTTCTTCTGGCGTTGAAGCTGGAGCTGGGACAGGATGAACAGTCTACCCACCCCTAGCAGGAAACCTAGCCCACGCAGGGGCATCAGTAGATCTAACAATCTTCTCATTACACTTAGCAGGTGTTTCGTCAATCCTTGGGGCAATCAACTTTATCACCACAATTATTAATATGAAACCCCCAGCCATCTCCCAGTATCAAACACCTCTATTCGTCTGATCCGTACTTGTAACTGCTGTTCTTCTTCTCCTGTCACTGCCTGTTTTAGCTGCTGGAATTACAATACTCCTAACGGATCGAAACCTTAACACCACATTCTTTGACCCGGCAGGAGGAGGAGACCCCATCCTTTACCAACACCTATTCTGATTCTTCGGCCACCCGGAAGTTTACATCCTCATCCTCCCAGGATTTGGAATTATCTCCCATGTTGTAGCCTACTACTCAGGTAAAAAGGAGCCATTTGGTTACATGGGAATAGTTTGAGCCATAATGGCCATCGGCCTTCTAGGGTTTATCGTATGGGCTCATCACATATTCACCGTTGGAATAGACGTTGACACCCGCGCATACTTTACGTCTGCAACAATAATCATTGCAATCCCAACAGGTGTAAAAGTGTTTAGCTGACTAGCCACGCTCCACGGAGGATCAATCAAATGAGAAACACCTATACTATGAGCCCTGGGGTTCATTTTCCTGTTCACAGTAGGTGGGCTCACAGGAATCGTCTTATCTAATTCATCACTTGATATTGTTCTCCATGACACCTATTATGTAGTCGCACATTTCCACTATGTACTATCCATGGGTGCTGTATTCGCCATTATAGCAGCCTTTGTGCACTGATTCCCACTACTAACCGGGTACACCCTTCATAGCACCTGAACAAAAATCCACTTCACAGTTATATTTATTGGAGTTAACCTTACATTCTTCCCACAACACTTCCTAGGCCTAGCGGGCATGCCACGACGATATTCTGACTACCCAGATGCCTACGCCCTATGAAACACAATCTCATCTATCGGATCGCTAATCTCATTGGTAGCAGTAATTATGTTCTTATTTATTCTATGAGAAGCCTTCACCGCTAAACGGGAAGTTCTATCTGTAGAACTAACAACAACAAATGTAGAATGACTCCATGGCTGCCCCCCTCCCTACCACACATACGAAGAACCAGCATTCGTTCAAGTTCAATCAAAC

>A._spinifer

GTGGCAATCACACGCTGATTCTTCTCTACCAATCACAAAGACATTGGCACCCTTTATCTTGTATTCGGTGCCTGAGCCGGAATAGTAGGAACTGGCCTAAGCCTCCTGATTCGGGCCGAGCTTAGTCAGCCTGGATCACTTCTTGGTGATGACCAAATTTATAATGTTATCGTAACTGCTCACGCATTCGTTATAATCTTCTTTATAGTAATACCCATCCTCATTGGGGGATTTGGTAATTGACTCGTACCACTAATAATTGGAGCCCCAGACATAGCGTTTCCACGAATAAATAATATAAGCTTCTGACTACTGCCCCCATCATTCCTGCTTTTATTAGCTTCTTCTGGCGTTGAGGCTGGAGCTGGGACAGGGTGAACAGTCTACCCTCCCCTTGCAGGAAACCTAGCTCACGCAGGGGCATCAGTAGACCTAACAATCTTCTCATTACACTTAGCAGGTGTTTCATCAATCCTTGGAGCAATCAACTTCATCACCACAATTATTAACATGAAACCCCCAGCCATCTCCCAATATCAAACACCCCTGTTCGTTTGATCCGTACTTGTAACTGCCGTTCTTCTTCTCCTATCACTGCCTGTTCTAGCTGCTGGAATTACAATGCTCCTAACAGATCGAAATCTCAACACCACATTCTTTGACCCAGCAGGAGGAGGAGACCCAATCCTCTACCAACACTTATTCTGATTCTTCGGCCACCCAGAAGTCTACATCCTCATTCTCCCAGGGTTCGGAATTATTTCTCACGTTGTAGCCTACTACTCCGGCAAAAAAGAACCGTTCGGCTACATAGGAATAGTCTGAGCTATGATGGCTATCGGCCTCCTGGGGTTTATCGTATGAGCTCACCACATATTCACTGTTGGAATAGACGTAGATACCCGCGCATACTTTACATCTGCAACAATAATTATTGCAATCCCAACAGGTGTAAAAGTGTTTAGCTGACTGGCCACGCTTCACGGAGGATCAATCAAATGAGAAACACCTATACTATGAGCCCTAGGGTTCATTTTCCTGTTTACAGTCGGTGGACTCACAGGAATTGTCCTATCCAACTCATCACTTGATATTGTTCTTCACGACACTTATTATGTAGTCGCACACTTCCACTATGTACTATCTATGGGGGCTGTGTTTGCCATCATAGCAGCCTTTGTACACTGATTCCCACTACTGACCGGATATACCCTTCACAGCGCCTGAACAAAAATCCATTTCACAGTCATATTCATCGGAGTTAACCTCACATTCTTCCCACAACACTTCCTTGGCCTAGCAGGTATGCCACGACGATATTCTGACTACCCGGATGCCTACGCCCTATGAAACACAGTCTCATCTATTGGATCACTAATCTCACTAGTAGCAGTAGTTATATTCCTATTTATCCTATGAGAAGCCTTCACCGCCAAACGAGAAGTGTTATCTGTAGAACTAACAATAACAAATGTAGAATGACTTCACGGCTGCCCCCCTCCCTATCACACATACGAGGAACCAGCATTCGTTCAAGTTCAATTAAAT

>A._stenotaeniatus

GTGGCAATCACACGCTGATTCTTCTCTACCAATCACAAAGACATTGGCACCCTTTATCTTGTATTCGGTGCCTGAGCCGGAATAGTAGGAACTGCCCTAAGCCTCCTGATTCGGGCCGAGCTTAGTCAGCCTGGATCACTTCTTGGTGATGACCAAATTTATAATGTTATCGTAACTGCTCACGCATTCGTTATAATCTTCTTTATAGTAATACCCATCCTCATTGGGGGATTTGGTAATTGACTCGTACCACTAATAATTGGAGCCCCAGACATAGCGTTTCCACGAATAAACAATATAAGCTTCTGACTACTGCCCCCATCATTCCTGCTTTTATTAGCTTCTTCTGGCGTTGAGGCTGGAGCTGGGACAGGGTGAACAGTCTACCCTCCCCTTGCAGGAAACCTAGCTCACGCAGGAGCATCAGTAGACCTAACAATCTTCTCATTACACTTAGCAGGTGTTTCATCAATCCTTGGAGCAATCAACTTCATCACCACAATTATTAACATGAAACCCCCAGCCATCTCCCAATATCAAACACCCCTGTTCGTTTGATCCGTACTTGTAACTGCCGTTCTTCTTCTCCTATCACTGCCTGTTCTAGCTGCTGGAATTACAATGCTCCTAACAGATCGAAATCTCAACACCACATTCTTTGACCCAGCAGGAGGAGGAGACCCAATCCTCTACCAACACTTATTCTGATTCTTCGGCCACCCTGAAGTCTACATCCTCATTCTCCCAGGGTTCGGAATTATTTCTCACGTTGTAGCCTACTACTCCGGCAAAAAAGAACCGTTCGGCTACATAGGAATAGTCTGAGCTATGATGGCTATCGGCCTCCTGGGGTTTATCGTATGAGCTCACCACATATTCACTGTTGGAATAGACGTAGATACCCGCGCATACTTTACATCTGCAACAATAATTATTGCAATCCCAACTGGTGTAAAAGTGTTCAGCTGACTGGCCACACTTCACGGAGGATCAATCAAATGAGAAACACCTATACTATGAGCCCTAGGGTTCATTTTCCTGTTTACAGTCGGTGGGCTCACAGGAATTGTCCTATCCAACTCATCACTTGATATTGTTCTCCACGACACTTATTATGTAGTCGCACACTTCCACTATGTACTATCTATAGGGGCTGTGTTTGCCATCATAGCAGCCTTTGTACACTGATTCCCACTACTAACCGGATATACCCTTCACAGCGCCTGAACAAAAATCCATTTCACAGTCATATTCATCGGAGTTAACCTCACATTCTTCCCACAACACTTCCTTGGCCTAGCAGGTATGCCACGACGATATTCTGACTACCCGGATGCCTACGCCCTATGAAACACAGTCTCATCTATTGGATCACTAATCTCACTAGTAGCAGTAGTTATATTCCTATTTATCCTATGAGAAGCCTTCACCGCCAAACGAGAAGTGTTATCTGTAGAACTAACAATAACAAATGTAGAATGACTTCACGGCTGCCCCCCTCCCTATCACACATACGAGGAACCAGCATTCGTTCAAGTTCAATTAAAT

>A._wenchowensis_1

GTGGCAATCACGCGCTGATTCTTCTCTACTAACCACAAAGACATTGGTACCCTTTATCTCGTATTTGGTGCCTGAGCCGGAATAGTGGGAACTGCCTTAAGCCTCCTAATTCGGGCCGAGCTTAGTCAACCCGGATCACTTCTAGGTGATGATCAAATTTACAATGTTATCGTAACTGCTCACGCATTTGTTATAATCTTCTTTATAGTAATACCCATCCTCATCGGAGGATTTGGAAATTGACTCGTACCACTAATAATCGGAGCCCCGGACATAGCATTCCCGCGAATAAATAATATAAGCTTCTGACTACTACCCCCATCATTCCTACTTTTACTAGCTTCTTCTGGTGTTGAAGCTGGGGCCGGAACAGGATGAACAGTCTACCCGCCCTTAGCAGGAAACCTGGCCCATGCAGGAGCATCAGTAGATCTGACAATCTTCTCACTACACTTAGCAGGTGTTTCATCAATCCTCGGGGCAATCAACTTCATCACCACAATTATTAACATGAAACCCCCAGCCATCTCCCAATATCAAACACCTCTGTTCGTCTGATCCGTACTTGTAACTGCCGTTCTTCTTCTCCTATCATTACCTGTTTTAGCCGCCGGAATTACAATACTCCTAACAGATCGAAACCTCAACACCACATTCTTTGACCCAGCAGGGGGAGGGGACCCAATCCTCTACCAACACCTGTTCTGATTCTTTGGCCACCCAGAAGTCTACATCCTTATCCTCCCAGGATTCGGAATCATCTCTCATGTTGTAGCCTACTACTCCGGTAAAAAGGAACCATTTGGTTACATGGGAATAGTTTGAGCCATAATAGCTATCGGTCTTCTAGGGTTTATCGTATGAGCCCACCATATATTCACCGTTGGAATAGACGTTGATACCCGCGCATACTTTACATCTGCAACAATAATCATCGCAATCCCAACAGGTGTAAAAGTGTTTAGCTGACTAGCCACACTCCACGGAGGATCAATCAAATGGGAAACACCTATACTATGAGCCCTAGGATTCATTTTCCTATTCACAGTGGGGGGACTCACAGGAATTGTCCTATCTAATTCATCACTTGATATTGTTCTTCATGACACTTACTATGTAGTCGCACACTTCCACTACGTGTTATCTATGGGTGCTGTATTTGCCATTATAGCAGCCTTTGTACACTGATTCCCATTACTAACCGGGTATACCCTTCACAGCACCTGAACAAAAATCCACTTCACAGTCATATTTGCTGGAGTTAACCTTACATTCTTCCCACAACACTTCCTAGGCTTAGCGGGTATGCCACGACGATATTCTGACTACCCAGATGCCTACGCCCTATGAAACACAATTTCATCTATTGGATCACTAATCTCATTAGTAGCAGTAATTATGTTCTTATTTATTCTATGAGAAGCCTTCACCGCTAAACGAGAAGTACTATTTGTTGAACTAACAACAACAAATGTAGAGTGACTTCACGGCTGCCCCCCTCCCTATCACACATACGAGGAACCAGCATTCGTTCAAGTTCAATCAAAC

>A._wenchowensis_2

GTGGCAATCACGCGCTGATTCTTCTCTACTAACCACAAAGACATTGGTACCCTTTATCTCGTATTTGGTGCCTGAGCCGGAATAGTGGGAACTGCCCTAAGCCTCCTAATTCGGGCCGAGCTTAGTCAACCCGGATCACTTCTCGGTGATGATCAAATTTACAACGTTATCGTAACTGCTCACGCATTTGTTATAATCTTCTTTATAGTAATACCCATCCTCATTGGGGGATTTGGAAATTGACTCGTACCACTAATGATTGGAGCCCCGGACATAGCATTCCCGCGAATAAATAACATAAGCTTCTGACTACTGCCCCCGTCATTCCTGCTTTTACTAGCCTCTTCTGGCGTTGAAGCCGGGGCTGGAACAGGGTGAACAGTCTACCCGCCCCTAGCAGGAAACCTGGCTCATGCAGGAGCATCAGTAGATCTGACAATCTTCTCACTACATTTAGCAGGTGTTTCATCAATCCTCGGGGCAATCAACTTCATCACCACAATTATTAATATGAAACCCCCGGCCATCTCCCAATATCAAACACCTCTGTTCGTCTGATCCGTACTTGTAACTGCCGTTCTTCTCCTCCTATCATTACCAGTTTTAGCTGCCGGAATTACAATACTCCTAACAGATCGAAACCTCAACACCACATTCTTTGACCCAGCAGGAGGGGGAGACCCAATCCTCTACCAACATCTGTTCTGATTCTTTGGCCACCCAGAAGTTTACATCCTTATCCTCCCAGGATTTGGAATCATCTCTCATGTTGTAGCCTACTACTCCGGTAAAAAAGAACCATTTGGTTACATGGGAATAGTTTGAGCCATAATGGCTATCGGTCTTCTAGGGTTTATCGTATGAGCTCACCACATATTCACCGTTGGAATAGACGTTGATACCCGCGCATACTTTACATCTGCAACAATAATCATTGCAATCCCGACAGGTGTAAAAGTATTTAGCTGACTAGCCACACTCCACGGGGGATCAATCAAATGAGAAACACCCATACTGTGAGCCCTAGGATTCATTTTCCTGTTTACAGTGGGGGGACTCACAGGAATTGTCCTATCTAATTCATCACTTGATATTGTTCTTCATGACACTTATTATGTAGTCGCACATTTCCACTATGTATTATCTATGGGTGCTGTATTTGCCATTATAGCAGCCTTTGTACACTGATTCCCATTACTAACCGGATATACCCTTCACAGCACCTGAACAAAAATCCACTTCACAGTTATATTTGCTGGGGTAAACCTCACATTCTTCCCACAACACTTCCTAGGCTTAGCGGGCATGCCACGACGATATTCTGATTACCCGGATGCCTACGCCCTATGAAACACAATTTCATCTATCGGGTCACTAATCTCATTAGTAGCAGTAATTATGTTCTTATTTATTCTATGAGAAGCCTTCACCGCTAAACGAGAAGTACTATCTGTTGAACTAACAACCACAAATGTAGAGTGACTTCACGGCTGCCCCCCTCCCTACCACACATACGAGGAACCAGCATTCGTTCAAGTTCAATCAAAC

>A._wuyiensis

GTGGCAATCACACGCTGATTCTTCTCTACCAACCACAAAGACATTGGCACCCTTTATCTCGTATTTGGTGCCTGAGCCGGAATAGTAGGAACTGCCCTAAGCCTTCTGATTCGAGCCGAGCTTAGTCAGCCTGGATCGCTTCTTGGTGATGACCAAATTTATAATGTTATCGTAACTGCTCACGCATTTGTTATAATCTTCTTTATAGTAATACCCATCCTTATTGGAGGATTTGGAAATTGACTCGTGCCACTAATAATTGGAGCCCCAGACATAGCATTTCCACGAATAAATAACATAAGCTTTTGACTACTGCCCCCATCATTCCTGCTTTTATTAGCTTCTTCTGGTGTTGAAGCTGGAGCCGGAACAGGGTGAACAGTCTATCCACCCCTTGCAGGAAACCTGGCTCACGCAGGGGCATCAGTAGACCTAACAATCTTCTCGTTACACTTAGCAGGTGTCTCATCAATCCTTGGGGCAATCAACTTCATCACCACAATTATTAACATGAAACCCCCAGCTATTTCCCAATATCAAACGCCCCTGTTTGTCTGATCCGTACTTGTAACTGCCGTTCTTCTTCTCCTTTCACTGCCCGTCTTAGCTGCCGGTATTACAATACTCCTAACAGATCGAAATCTCAACACCACATTCTTTGATCCCGCAGGAGGAGGAGACCCAATCCTTTACCAACACTTATTCTGATTCTTCGGCCACCCAGAAGTTTATATCCTCATCCTCCCAGGATTCGGAATTATTTCTCATGTTGTAGCCTACTACTCCGGTAAAAAAGAGCCGTTTGGCTACATAGGAATAGTTTGAGCCATGATGGCCATCGGCCTTCTAGGATTTATCGTATGAGCTCACCACATATTCACCGTTGGAATAGACGTAGATACTCGCGCATACTTTACATCTGCAACAATAATCATTGCAATCCCAACAGGTGTAAAAGTGTTTAGCTGACTGGCCACGCTTCACGGAGGATCAATCAAATGAGAAACACCTATACTATGAGCCCTAGGGTTCATTTTCTTATTTACAGTCGGTGGGCTTACAGGAATCGTCCTATCCAACTCGTCACTCGACATTGTTCTTCACGACACTTATTATGTAGTTGCACATTTCCATTACGTACTATCTATAGGCGCTGTATTTGCCATCATAGCAGCCTTCGTACACTGATTCCCGCTACTAACCGGATATACCCTCCACAGTACCTGAACAAAAATCCATTTTACAGTCATGTTTATTGGAGTCAACCTCACATTCTTCCCACAACACTTCCTTGGTTTAGCAGGTATGCCACGGCGATATTCTGACTACCCAGATGCCTACGCCCTGTGAAACACAGTCTCATCTTTTGGGTCACTAATCTCATTAGTAGCAGTAGTTATATTCTTATTCATCCTATGAGAAGCCTTCACCGCCAAACGAGAAGTATTATCTGTCGAACTAACAATAACAAATGTAGAATGACTTCACGGCTGCCCCCCTCCCTATCACACATACGAGGAACCAGCATTTGTACAAGTTCAATCAAAT

>A._yunnanensis_1

GTGGCAATCACGCGCTGATTCTTCTCTACTAACCACAAAGACATTGGTACCCTTTATCTCGTATTTGGTGCCTGAGCCGGAATAGTGGGAACCGCCCTAAGCCTTCTAATTCGGGCTGAACTAAGTCAACCCGGGTCGCTTCTAGGTGATGACCAAATTTACAATGTTATCGTTACTGCCCACGCCTTCGTTATAATTTTCTTTATAGTAATGCCCATCCTTATTGGAGGATTTGGAAACTGACTCGTACCACTAATAATTGGAGCCCCGGACATGGCATTCCCACGAATAAATAACATAAGCTTCTGATTACTACCCCCATCATTCCTACTGCTACTAGCTTCTTCTGGTGTTGAAGCTGGGGCCGGAACAGGATGAACAGTTTACCCGCCCCTTGCAGGAAATCTGGCCCACGCAGGAGCATCAGTAGACCTAACAATTTTCTCCCTCCACTTAGCAGGTGTTTCATCAATCCTTGGGGCAATCAACTTCATCACCACAATCATTAACATGAAACCCCCAGCCATCTCTCAATACCAAACACCTCTGTTCGTCTGATCCGTGCTTGTAACCGCTGTACTTCTTCTCCTGTCACTACCTGTCCTAGCCGCTGGGATCACAATGCTCCTCACAGATCGAAACCTCAACACTACATTCTTTGACCCAGCAGGGGGAGGAGACCCAATCCTTTACCAACACCTATTCTGATTCTTCGGTCACCCAGAAGTCTATATCCTCATCCTTCCAGGGTTTGGAATTATTTCTCATGTCGTAGCCTATTATTCAGGTAAAAAAGAACCATTTGGCTACATAGGAATAGTTTGGGCCATAATAGCCATTGGCCTTCTGGGGTTTATCGTATGGGCCCACCACATATTCACCGTTGGAATAGACGTAGACACCCGTGCATACTTTACATCTGCAACAATAATCATCGCAATCCCAACAGGTGTAAAAGTATTTAGCTGACTTGCCACACTCCACGGAGGGTCAATCAAATGAGAAACACCCATACTATGAGCCCTAGGGTTCATTTTCCTATTTACAGTGGGCGGGCTCACAGGAATTGTCCTGTCTAACTCATCACTTGACATTGTTCTTCATGACACTTATTACGTAGTCGCACATTTCCACTACGTATTATCTATGGGCGCTGTATTCGCTATTATAGCAGCCTTCGTTCACTGATTCCCTCTATTAACTGGATACACCCTTCATAGCGCCTGAACAAAAATCCACTTCGCAGTCATATTTATTGGAGTTAACCTTACGTTCTTCCCACAACACTTCCTAGGACTGGCGGGCATGCCACGACGATACTCTGACTACCCAGATGCCTATGCCTTATGAAACACAATATCATCTATCGGATCACTAATTTCATTAGTAGCGGTAATTATGTTCTTATTTATCCTGTGAGAAGCCTTCACCGCTAAGCGAGAAGTACTATCTGTAGAACTAACAACAACAAACGTAGAATGACTCCACGGCTGCCCCCCTCCTTACCACACATACGAGGAACCAGCGTTTGTACAAGTTCAATCGAAC

>A._yunnanensis_2

GTGGCAATCACGCGCTGATTCTTCTCTACTAACCACAAAGACATTGGTACCCTTTATCTCGTATTTGGTGCCTGAGCCGGAATAGTGGGAACCGCCCTAAGCCTTCTAATTCGGGCTGAACTAAGTCAACCCGGGTCGCTTCTAGGTGATGACCAAATTTACAATGTTATCGTTACTGCCCACGCCTTCGTTATAATTTTCTTTATAGTAATGCCCATCCTTATTGGAGGATTTGGAAACTGACTCGTACCACTAATAATTGGAGCCCCGGACATGGCATTCCCACGAATAAATAACATAAGCTTCTGATTACTACCCCCATCATTCCTACTGCTACTAGCTTCTTCTGGTGTTGAAGCTGGGGCCGGAACAGGGTGAACAGTTTACCCGCCCCTTTCAGGAAATCTGGCCCACGCAGGAGCATCAGTAGACCTAACAATTTTCTCCCTCCACTTAGCAGGTGTCTCATCAATCCTTGGGGCAATCAACTTCATCACCACAATTATCAACATGAAACCCCCAGCCATCTCTCAATACCAAACACCTCTGTTCGTCTGATCCGTGCTTGTAACCGCTGTGCTTCTTCTCCTGTCACTACCTGTCCTAGCCGCTGGGATCACAATACTCCTCACAGATCGAAACCTCAACACTACATTCTTTGACCCGGCAGGGGGAGGAGACCCAATCCTTTACCAACACCTATTCTGATTCTTCGGCCACCCAGAAGTCTATATCCTCATCCTTCCAGGGTTTGGAATTATTTCTCATGTCGTAGCCTATTATTCAGGCAAAAAAGAACCATTTGGCTACATAGGAATAGTTTGGGCCATAATAGCCATTGGCCTTCTGGGGTTTATCGTGTGAGCCCACCACATATTCACCGTTGGAATAGACGTAGACACCCGTGCATACTTTACATCTGCAACAATAATCATCGCAATCCCAACAGGTGTAAAAGTATTTAGCTGACTGGCCACACTCCACGGAGGGTCAATCAAATGAGAAACACCTATACTATGAGCCCTAGGGTTCATTTTCCTATTTACAGTGGGCGGGCTCACAGGAATTGTCCTGTCCAACTCATCACTTGACATTGTTCTTCACGACACTTATTATGTAGTCGCACATTTCCACTACGTGTTATCTATGGGTGCTGTATTCGCTATTATAGCAGCCTTTGTTCACTGATTCCCTCTATTAACTGGATACACCCTCCATAGCGCCTGAACAAAAATCCACTTCGCAGTCATATTTATTGGAGTTAACCTTACGTTCTTCCCACAACACTTCCTAGGACTGGCGGGCATGCCACGACGATACTCTGACTACCCAGATGCCTATGCCTTATGAAACACAATATCATCTATCGGATCACTAATTTCATTAGTGGCGGTAATTATGTTCTTATTTATCCTGTGAGAAGCCTTCACCGCTAAACGAGAAGTACTATCTGTAGAACTAACAACAACAAACGTAGAATGACTCCACGGCTGCCCCCCTCCTTACCACACATACGAGGAACCAGCATTTGTACAAGTTCAATCGAAC

>Onychostoma_barbatulum

GTGGCAATCACACGCTGATTCTTCTCTACCAACCACAAAGACATTGGCACCCTTTATCTAGTATTTGGTGCCTGAGCCGGAATAGTGGGAACTGCCCTAAGCCTCCTAATTCGGGCTGAACTGAGCCAACCCGGGTCGCTTCTGGGTGATGACCAAATTTACAACGTTATCGTAACTGCTCACGCCTTTGTTATAATCTTCTTTATAGTAATGCCCATCCTTATTGGAGGTTTCGGAAACTGGCTCGTACCACTAATAATTGGGGCCCCCGATATGGCATTCCCACGAATGAACAACATAAGCTTCTGATTATTACCCCCATCATTCCTGCTGCTCCTGGCTTCTTCCGGTGTAGAGGCGGGAGCTGGGACAGGATGAACAGTTTACCCGCCCCTTGCGGGAAACCTGGCCCACGCAGGGGCATCCGTAGACTTAACAATCTTTTCGCTACATTTAGCAGGTGTTTCATCAATCCTGGGGGCAATCAACTTCATCACTACAATTATTAATATGAAACCCCCAGCTATTTCCCAATACCAAACACCCCTATTCGTATGATCCGTGCTTGTTACTGCCGTCCTTCTTCTCCTATCGCTACCTGTCTTAGCTGCTGGGATTACAATACTCCTAACAGATCGAAACCTCAACACCACATTCTTTGACCCGGCAGGGGGAGGGGACCCAATCCTCTACCAACACCTATTCTGATTCTTCGGTCACCCAGAAGTTTACATTCTCATCCTCCCGGGATTTGGAATTATTTCCCATGTTGTAGCCTACTATTCCGGTAAAAAAGAACCATTTGGTTACATAGGGATGGTTTGGGCTATAATAGCCATTGGACTTCTAGGGTTCATCGTATGGGCCCACCACATATTTACCGTTGGAATAGATGTAGATACTCGCGCATACTTCACATCCGCAACAATAATCATCGCAATCCCAACAGGCGTAAAAGTATTTAGCTGATTAGCCACACTCCACGGAGGATCAATCAAATGAGAAACACCAATACTATGAGCCCTTGGATTCATCTTCCTCTTTACAGTAGGCGGACTTACGGGAATTGTCTTATCTAATTCATCCCTTGATATTGTTTTACATGATACTTATTATGTAGTTGCACACTTCCACTACGTCCTATCCATAGGTGCCGTGTTTGCTATTATAGCAGCCTTTGTACACTGATTCCCCCTACTAACCGGGTATACCCTTCACAGCACCTGAACAAAAATCCATTTCGCAATTATATTTATTGGGGTAAATCTTACATTCTTCCCGCAACACTTCCTGGGCCTAGCGGGCATACCACGACGATACTCTGATTACCCAGATGCCTATGCCTTATGAAACACAATTTCATCTATTGGATCACTAATTTCACTAGTAGCAGTAATTATATTCTTATTTATCCTATGAGAAGCCTTCACCGCTAAACGAGAAGTACTATCTGTTGAATTAACAACAACAAACGTAGAATGACTTCACGGCTGCCCCCCTCCTTACCACACATACGAAGAACCAGCATTTGTTCAAGTTCAATCCAAC

>Onychostoma_meridionale

GTGGCAATCACGCGCTGATTCTTCTCTACCAACCACAAAGACATTGGTACCCTTTATCTCGTATTTGGTGCCTGAGCCGGAATAGTAGGAACTGCCCTAAGCCTCCTAATTCGGGCTGAGCTAAGTCAACCCGGATCGCTTCTGGGCGATGACCAAATTTATAACGTTATCGTAACTGCCCACGCCTTTGTTATAATCTTCTTTATAGTAATGCCCATCCTTATTGGAGGGTTCGGAAACTGACTCGTGCCCTTAATAATCGGAGCCCCCGACATGGCGTTCCCACGAATAAATAACATAAGCTTCTGATTACTACCCCCATCATTCCTGCTACTCCTGGCTTCCTCTGGTGTCGAAGCAGGAGCTGGAACAGGATGAACAGTCTACCCACCCCTTGCAGGAAACCTGGCCCACGCAGGAGCATCAGTAGACCTAACAATTTTCTCACTCCATTTAGCAGGTGTCTCATCAATCCTGGGGGCAATCAACTTTATCACTACGATTGTTAACATGAAACCCCCAGCCATCTCCCAGTATCAAACACCCCTATTCGTCTGATCCGTGCTTGTAACTGCTGTCCTCCTTCTCCTGTCACTACCTGTTTTAGCTGCTGGGATCACAATACTCTTAACAGACCGAAACCTTAATACCACATTCTTTGACCCAGCAGGGGGAGGAGACCCGATCCTCTACCAACACCTGTTTTGATTCTTCGGCCACCCAGAAGTCTACATCCTCATCCTCCCAGGGTTCGGAATTATTTCCCATGTCGTAGCCTACTATTCTGGTAAAAAAGAACCATTTGGTTACATAGGAATGGTCTGAGCCATAATAGCCATCGGCCTTCTAGGGTTCATCGTGTGGGCCCATCACATATTTACCGTCGGAATAGATGTAGACACCCGCGCATACTTTACATCTGCAACAATAATCATCGCAATCCCAACGGGTGTAAAAGTGTTTAGCTGACTAGCTACACTCCACGGGGGGTCAATCAAATGAGAAACACCCATACTATGAGCCCTAGGATTCATCTTCCTATTTACAGTGGGCGGACTCACGGGAATTGTCTTATCTAACTCATCACTCGACATCGTCTTACATGACACCTACTACGTAGTCGCACATTTCCACTACGTTCTATCCATGGGTGCCGTATTTGCCATTATAGCAGCCTTCGTACACTGATTTCCTTTATTAACCGGGTACACCCTTCACAGCACCTGAACAAAAATCCATTTCACAGTTATATTTATTGGGGTCAACCTTACATTCTTCCCACAACATTTCCTAGGTCTAGCAGGCATGCCACGACGATATTCTGACTACCCGGACGCCTATGCCCTATGAAACACAATCTCATCTATTGGATCACTAATCTCACTAGTAGCAGTAATTATGTTCTTATTTATTCTGTGAGAAGCCTTCACCGCCAAACGAGAGGTGTTATCTGTAGAACTAACAACAACAAACGTAGAATGACTCCACGGCTGCCCCCCTCCCTACCACACATACGAGGAGCCAGCATTTGTCCAAGTTCAATCAAAC

>Onychostoma_gerlachi

GTGGCAATCACGCGCTGATTCTTCTCTACCAACCACAAAGACATTGGTACCCTTTATCTCGTATTTGGTGCCTGAGCCGGAATAGTGGGAACTGCCCTAAGCCTCCTAATTCGGGCTGAACTGAGCCAACCCGGGTCGCTTCTGGGCGATGACCAAATTTATAACGTTATCGTAACTGCCCACGCCTTCGTCATAATTTTCTTTATAGTAATGCCCATCCTTATTGGGGGGTTCGGAAATTGACTTGTACCTCTAATAATCGGAGCCCCCGACATGGCATTCCCACGAATAAATAACATAAGCTTCTGATTACTACCTCCATCATTTCTGCTACTCCTAGCTTCTTCTGGTGTTGAAGCAGGGGCCGGGACAGGATGAACCGTTTATCCACCCCTTGCAGGAAACCTGGCCCACGCAGGGGCATCAGTAGACCTGACAATTTTCTCACTTCATTTAGCAGGTGTCTCATCAATCCTAGGGGCAATCAACTTCATCACTACGATTGTTAACATAAAACCCCCAGCCATCTCCCAATATCAAACACCCCTATTCGTCTGATCCGTGCTTGTAACTGCTGTCCTCCTTCTCCTGTCACTACCTGTTTTAGCTGCCGGAATCACAATACTATTAACAGACCGAAACCTCAATACCACATTCTTTGACCCGGCAGGGGGAGGAGACCCAATCCTCTACCAACACCTGTTCTGATTCTTCGGCCACCCAGAAGTCTACATCCTCATCCTCCCGGGGTTCGGAATTATTTCCCATGTCGTAGCCTACTATTCTGGTAAAAAAGAACCATTTGGCTACATGGGGATGGTCTGAGCCATAATAGCCATCGGCCTTCTGGGATTCATCGTATGAGCCCACCACATATTCACCGTCGGAATAGACGTAGACACCCGCGCATACTTTACATCTGCAACAATAATCATCGCAATCCCAACAGGTGTAAAAGTGTTTAGCTGACTTGCCACGCTCCACGGAGGGTCAATCAAATGAGAAACACCCATACTATGAGCCCTGGGGTTCATCTTCCTATTCACAGTTGGCGGACTCACGGGAATTGTTCTATCTAACTCATCACTCGACATCGTCTTACATGACACCTACTACGTAGTTGCTCACTTCCACTACGTGCTTTCTATGGGTGCCGTATTTGCTATTATAGCAGCCTTCGTACATTGATTTCCTTTATTAACCGGGTACACCCTTCACAGCACCTGAACAAAAATCCATTTTGCAGTTATATTTATTGGGGTTAACCTTACATTCTTCCCACAACACTTCCTAGGCCTAGCAGGCATGCCACGACGATATTCTGACTACCCGGATGCCTATGCTCTATGAAACACAATTTCATCTATCGGATCACTAATCTCACTAGTAGCAGTAATTATGTTCCTATTTATCCTGTGAGAAGCCTTCACCGCCAAACGAGAAGTACTATCTGTAGAATTAACAACAACAAACGTAGAATGACTCCACGGCTGCCCCCCTCCCTACCACACATACGAGGAACCAGCATTTGTCCAAGTTCAATCAAAC

>Spinibarbus_denticulatus

GTGGCAATCACGCGCTGATTCTTCTCTACTAATCACAAAGACATTGGCACCCTTTATCTTGTATTTGGTGCCTGAGCCGGAATAGTGGGAACCGCCTTAAGCCTCCTCATCCGGGCTGAACTAAGCCAAACCGGGTCGCTTCTAGGTGATGACCAAATTTATAACGTTATCGTTACTGCCCACGCCTTCGTAATAATTTTCTTTATAGTAATGCCTATTCTCATTGGAGGATTTGGAAATTGACTTGTACCCCTAATAATTGGAGCCCCAGACATAGCATTCCCACGAATAAATAATATAAGCTTCTGACTATTACCTCCATCATTCCTGCTACTATTAGCTTCTTCTGGAGTTGAAGCCGGGGCCGGAACAGGATGAACAGTTTATCCCCCCCTTGCAGGAAATCTAGCCCACGCAGGAGCATCAGTAGACCTAACAATTTTCTCCCTCCACTTGGCAGGTGTTTCATCAATCCTAGGGGCAATCAATTTTATTACCACAACTATCAATATAAAACCTCCAGCTATCTCCCAGTATCAAACACCTCTATTTGTCTGATCTGTACTTGTAACCGCCGTACTACTCCTTCTATCACTACCTGTTTTAGCCGCCGGAATTACAATACTACTAACAGACCGAAATCTTAACACTACATTCTTTGATCCCGCAGGAGGGGGGGACCCAATTCTTTACCAACACTTATTCTGATTCTTTGGCCACCCAGAAGTTTACATTCTCATTCTCCCAGGATTTGGGATCATTTCTCACGTTGTAGCCTACTATTCAGGTAAAAAAGAACCATTTGGTTATATAGGAATAGTTTGAGCTATAATGGCTATTGGCCTCCTGGGGTTCATCGTATGGGCCCACCATATATTCACCGTTGGAATAGACGTAGACACCCGCGCATATTTTACATCTGCGACAATAATTATCGCAATTCCAACAGGTGTAAAAGTATTTAGCTGACTAGCTACACTTCACGGAGGGTCAATTAAATGAGAAACACCCATACTATGGGCTCTCGGGTTTATTTTCCTGTTTACAGTAGGTGGGCTCACAGGAATTGTCCTGTCCAACTCATCACTTGATATTGTCCTTCATGACACTTATTATGTAGTCGCACACTTCCACTACGTGCTATCAATAGGTGCTGTATTTGCTATTATAGCAGCATTTGTACACTGATTCCCCCTACTAACCGGATACACCCTTCACAGCGCTTGAACAAAAATTCACTTCGGGGTTATATTCATTGGAGTTAACCTTACGTTCTTCCCACAACACTTCCTAGGCTTAGCAGGCATGCCACGGCGATATTCTGACTACCCAGATGCCTATGCCCTATGAAACACAGTATCATCTATCGGGTCACTAATTTCTTTAGTGGCGGTAATTATATTCCTATTTATTCTATGAGAAGCCTTCGCCGCTAAACGAGAAGTACTATCTGTAGAATTAACAATAACAAATGTAGAATGACTTCATGGCTGCCCTCCTCCCTACCACACATACGAAGAGCCAGCATTTGTTCAAATTCAATCAAAC

>Spinibarbus_hollandi

GTGGCAATCACGCGCTGATTCTTCTCTACTAATCACAAAGACATTGGCACCCTTTATCTCGTATTTGGTGCCTGAGCCGGAATAGTGGGAACCGCCTTAAGCCTCCTCATCCGGGCTGAACTAAGCCAACCCGGATCGCTTCTAGGTGATGACCAAATTTATAATGTTATCGTTACAGCCCACGCCTTCGTAATAATTTTCTTTATAGTAATACCTATTCTCATTGGGGGATTTGGAAATTGACTTGTGCCCCTAATAATTGGAGCCCCAGACATAGCATTCCCACGAATAAATAATATAAGTTTCTGACTATTACCTCCATCATTCCTACTATTATTAGCTTCTTCTGGTGTTGAAGCCGGGGCCGGAACAGGATGAACAGTTTATCCCCCTCTCGCAGGGAATCTAGCCCACGCAGGAGCATCAGTGGACCTAACAATTTTCTCACTTCACTTAGCACGTGTTTCATCAATCCTAGGGGCAATCAATTTTATTACCACAACTATTAATATAAAACCTCCAGCTATTTCCCAGTATCAAACGCCTCTATTTGTCTGATCCGTGCTTGTAACCGCCGTACTACTTCTTCTATCATTACCTGTTTTAGCTGCCGGAATTACAATACTCCTAACAGACCGAAATCTTAACACTACATTCTTTGATCCTGCAGGAGGAGGGGACCCAATCCTTTACCAACACTTATTCTGATTCTTTGGCCACCCAGAAGTCTACATTCTTATTCTCCCAGGATTTGGAATCATTTCTCACGTTGTAGCCTACTATTCAGGTAAAAAAGAACCATTTGGTTACATAGGAATAGTTTGAGCTATAATGGCTATCGGCCTCCTGGGATTTATCGTATGAGCCCACCATATATTCACGGTTGGGATAGACGTAGACACTCGCGCATATTTTACATCCGCAACAATAATTATCGCAATTCCAACAGGTGTGAAAGTATTTAGCTGATTAGCTACACTCCACGGAGGGTCAATTAAATGAGAAACACCCATACTATGGGCCCTCGGGTTTATTTTCCTATTTACAGTAGGTGGGCTCACAGGAATCGTCCTGTCCAACTCATCACTTGATATTGTCCTTCATGACACTTATTATGTAGTCGCACACTTCCACTACGTACTATCAATAGGTGCTGTGTTTGCTATTATAGCAGCATTTGTACACTGATTTCCCCTACTAACAGGATACACCCTTCACAGCGCCTGAACAAAAATTCACTTCGGGGTTATATTTATTGGAGTTAACCTTACGTTCTTCCCACAACACTTCCTGGGCTTAGCAGGCATGCCACGGCGATATTCTGACTACCCAGATGCCTACGCCCTATGAAACACAGTATCATCTATCGGGTCACTAATTTCTTTAGTGGCGGTAATTATATTCCTATTTATCCTATGAGAAGCCTTCGCCGCTAAACGAGAAGTACTATCTGTAGAATTAACAATAACAAATGTAGAATGACTTCATGGCTGCCCTCCTCCCTACCACACATACGAAGAACCAGCATTCGTTCAAATTCAATTAAAC

>Spinibarbus_sinensis

GTGGCAATCACGCGCTGATTCTTCTCTACTAACCACAAAGACATTGGTACCCTTTATCTTGTATTTGGTGCCTGAGCCGGAATAGTGGGAACTGCCTTAAGCCTCCTTATTCGGGCTGAACTAAGTCAACCCGGGTCGCTTCTAGGTGATGACCAAATTTATAATGTTATCGTTACTGCCCACGCCTTCGTAATAATCTTCTTTATAGTAATACCTATTCTCATTGGAGGATTTGGAAACTGACTTGTACCATTAATAATCGGAGCCCCAGACATGGCATTCCCACGAATAAATAATATGAGCTTCTGGTTACTACCCCCATCATTCCTGTTATTACTAGCTTCCTCTGGCGTTGAGGCCGGAGCCGGGACAGGGTGAACTGTTTACCCACCTCTTGCAGGAAATCTAGCCCACGCAGGAGCATCAGTAGACCTAACAATTTTCTCACTTCACTTAGCAGGTGTTTCATCAATCCTGGGGGCCATCAATTTCATTACCACAACCATTAACATGAAACCCCCAGCCATCTCCCAATATCAAACACCTCTGTTTGTCTGATCCGTACTTGTAACCGCTGTACTACTTCTCCTATCACTACCTGTCTTAGCTGCCGGAATTACAATACTCCTAACAGATCGAAACCTTAACACCACATTCTTTGATCCAGCAGGAGGAGGAGACCCAATCCTTTATCAACACTTATTCTGGTTCTTTGGCCACCCAGAAGTTTATATTCTTATCCTTCCGGGATTTGGAATTATTTCTCATGTTGTAGCCTATTATTCAGGTAAAAAAGAACCATTTGGCTACATGGGAATGGTTTGAGCTATAATGGCTATTGGCCTTCTAGGGTTTATCGTATGAGCCCACCATATATTCACTGTTGGAATAGACGTAGATACTCGCGCATACTTTACATCTGCAACAATAATTATTGCAATTCCAACAGGTGTAAAAGTATTCAGTTGACTAGCCACACTCCACGGAGGATCAATTAAATGAGAAACACCCATATTATGAGCGCTCGGGTTCATTTTCCTATTTACAGTAGGCGGACTTACAGGAATTGTCCTATCTAACTCATCACTTGATATTGTTCTTCACGACACCTATTACGTAGTCGCACATTTCCACTACGTGTTATCAATGGGTGCTGTATTTGCTATTATGGCAGCTTTTGTACACTGATTCCCCCTACTAACCGGATATACCCTCCACAGCGCCTGAACAAAAATCCACTTTGGGGTTATATTTATTGGGGTTAACCTTACATTCTTCCCACAACACTTCCTAGGCTTAGCAGGCATGCCACGACGATACTCTGACTACCCAGATGCCTACGCCCTATGAAATACAGTGTCATCCATTGGATCACTAATTTCTTTAGTGGCGGTAATCATATTCCTATTTATTCTATGAGAAGCCTTCGCCGCTAAACGAGAAGTACTATCCGTAGAACTAACAATAACAAACGTAGAATGACTTCACGGTTGCCCTCCTCCTTACCACACATACGAGGAACCGGCATTTGTTCAAATTCAATCAAAC

**COX2**

>A._barbodon

ATGGCACATCCAACACAACTAGGATTCCAAGACGCAGCATCACCAGTCATAGAAGAGCTTCTTCACTTCCACGATCACGCACTAATAATTGTATTCCTAATTAGCGCCCTAGTACTATATATTATTATTGCAATAGTATCCACCAAACTCACTAATAAGTTTATTTTAGACTCCCAAGAAATCGAAATCGTATGAACTATTTTACCCGCCGTAATTTTAGTAATAATTGCTTTACCCTCCCTACGCATTCTTTACCTTATAGACGAAATCAACGACCCCCACCTAACAATTAAAGCAATAGGACATCAATGATACTGAAGCTACGAATACACTGACTATGAAAACCTAAACTTCGACGCCTATATAGTACCAACCCAAGACCTTGCCCCAGGACAATTCCGACTTCTAGAAACAGACCACCGAATAGTTATCCCAGTAGAATCCCCAATCCGAATTCTGGTGCCTGCCGAAGACGTGTTACACTCTTGAGCTGTTCCATCTATGGGTGTAAAAATAGATGCCGTCCCAGGACGACTTAACCAAACTGCTCTCCTCGCCTCACGCCCAGGGGTGTACTATGGACAATGCTCAGAAATTTGCGGGGCCAACCACAGCTTTATACCAATTGTAATTGAAACAGTACCCCTACCACACTTCGAAACTTGGTCCGCACACCAACTAGACTTCGCC

>A._beijiangensis_1

ATGGCCCATCCAACACAACTAGGATTCCAAGATGCAGCATCACCAGTCATAGAAGAACTTCTTCACTTCCACGATCACGCACTAATAATTGTATTCTTAATTAGTGCCCTAGTACTATATATTATTATTGCAATAGTCTCTACTAAACTTACCAATAAATTTATTTTAGATTCTCAAGAAATTGAAATTGTATGAACTATTTTACCCGCTGTAGTTTTAGTAATAATTGCTCTACCCTCCTTACGCATTCTATACCTTATAGACGAAATCGGCGACCCCCACCTAACAATTAAAGCAATAGGACACCAATGATACTGAAGCTACGAATATACAGACTATGAAGACCTAAACTTTGATGCTTACATAGTACCAACCCAAGACCTCACCCCAGGACAATTCCGACTCCTAGAAACAGACCACCGAGTAGTTATTCCAGTGGAATCCCCCATCCGCATTTTAGTATCTGCCGAAGACGTACTACACTCTTGAGCCGTCCCCTCCATGGGCGTAAAAATAGACGCAGTCCCAGGGCGACTTAACCAAACTGCCATCCTCGCTTCACGCCCAGGAGCATACTACGGACAATGCTCAGAAATTTGCGGAGCCAACCACAGCTTTATACCAATTGTAATTGAAGCAGTACCCCTACCACAATTTGAAACTTGATCCGCATACCAACTAGATCTCGCC

>A._beijiangensis_2

ATGGCCCATCCAACACAACTAGGATTCCAAGACGCAGCATCACCAGTCATAGAAGAACTTCTTCACTTCCACGATCACGCATTAATAATTGTATTCTTAATTAGTGCCCTAGTGCTATATATTATTATTGCAATAGTCTCTACCAAACTTACCAATAAGTTTATTTTAGATTCTCAAGAAATTGAAATTGTATGAACTATTTTACCCGCTGTAATTTTAGTAATAATTGCCCTACCCTCCTTACGCATTCTATACCTTATAGACGAAATCGGCGACCCTCACCTAACAATTAAAGCAATAGGACACCAATGATACTGAAGCTACGAATATACAGACTATGAAGACCTAAACTTTGATGCTTATATAGTACCAACCCAGGACCTCACCCCAGGACAATTCCGACTCCTAGAAACAGACCACCGAGTAGTTATTCCAGTGGAATCCCCCATCCGCATTCTAGTATCTGCCGAAGACGTACTACACTCTTGAGCCGTCCCCTCCATGGGCGTAAAAATAGACGCAGTCCCAGGGCGACTTAACCAAACTGCCATCCTCGCTTCACGCCCAGGAGCATACTACGGACAATGCTCAGAAATTTGCGGAGCCAACCACAGCTTTATACCAATTGTAATTGAAGCAGTACCCCTACCACAATTTGAAACTTGATCCGCATACCAACTAGACCTCGCC

>A._fasciatus

ATGGCACATCCAGCCCAACTAGGATTCCAAGACGCAGCATCACCAGTCATAGAAGAACTTCTTCACTTCCACGACCACGCACTAATAATCGTATTCCTAATTAGTGCTCTAGTATTATATATTATTATTGCAATAGTATCCACCAAACTTACTAATAAATTTATCTTAGACTCTCAGGAAATCGAAATTGTGTGAACTATTTTACCTGCCGTAATTTTAGTGTTAATTGCCCTGCCCTCTCTACGCATTCTATACCTCATAGACGAAATTAATGACCCCCACCTAACAATTAAAGCAATAGGACATCAATGATATTGAAGTTACGAGTATACGGACTATGAAAACCTAAATTTTGATGCCTACATAGTACCAACTCAAGACCTCACCCCAGGACAATTTCGACTCCTAGAAACAGACCACCGAATAGTCATCCCAGTAGAATCCCCAATCCGCATTCTAGTCTCTGCTGAAGACGTATTACACTCTTGAGCTATCCCTTCCATGGGCGTAAAAATAGATGCAGTCCCAGGACGACTTAATCAAACTGCCCTCCTTGCTTCACACCCAGGAGTGTACTACGGACAATGCTCAGAAATTTGCGGAGCCAACCACAGCTTCATACCAATTGTAGTTGAAGCAGTACCCCTACCACACTTCGAAACTTGGTCCGCATGTCAACTAGACCTTGCC

>A._hemispinus

ATGGCACACCCAGCCCAACTAGGATTCCAAGACGCAGCATCACCAGTCATAGAAGAACTTCTCCACTTCCACGATCACGCACTAATAATTGTATTCTTAATTAGTGCCCTAGTACTATACATTATTGTTGCAATAGTATCCACCAAACTTACCAATAAACTTATCTTAGACTCTCAAGAAATCGAAATTGTATGAACTATTTTACCCGCTGTAATTTTAGTATTAATTGCCCTACCCTCCCTACGCATTCTATACCTTATAGACGAAATCAATGACCCTCACCTAACAATTAAGGCAATGGGACACCAATGATACTGAAGCTACGAATATACGGACTATGAAAACCTAAATTTTGATGCCTACATAGTTCCTACCCAAGACCTCACCCCAGGACAATTTCGACTCCTAGAAACAGACCACCGAATAGTCATCCCAGTAGAATCTCCAATCCGCATTCTAGTTTCTGCCGAAGACGTGCTACACTCTTGAGCTGTTCCCTCCATGGGCGTGAAAATAGATGCAGTCCCAGGACGACTTAATCAAACCGCCCTCCTTGCTTCACACCCAGGAGTATACTACGGGCAATGCTCAGAAATTTGCGGAGCTAACCACAGCTTTATACCAATTGTAATTGAAGCAGTACCCCTACCACACTTCGAAACTTGGTCCGCATGTCAACTAGACCTTGCT

>A._iridescens

ATGGCACATCCAACACAACTAGGATTCCAAGACGCAGCATCACCAGTCATAGAAGAGCTTCTTCACTTCCACGATCACGCACTAATAATTGTATTTCTAATTAGCGCCCTAGTATTATATATTATTATTGCAATAGTATCCACCAAACTCACTAATAAGTTTATTTTAGACTCCCAAGAAATCGAAATCGTATGAACTATTTTACCCGCCGTAATTTTAGTAATAATTGCCCTACCTTCCCTACGCATTCTTTACCTTATAGACGAAATCAACGACCCCCACCTAACAATTAAAGCAATAGGACATCAATGATACTGAAGCTACGAATACACTGACTATGAAAACCTAAACTTCGACGCCTATATAGTACCAACCCAAGACCTTACCCCAGGGCAATTCCGACTTCTAGAAACAGATCACCGAATAGTTATTCCAGTAGAATCCCCAATCCGAATTCTGGTGTCTGCCGAAGACGTGTTACACTCTTGAGCTGTTCCATCTATGGGCGTAAAAATAGATGCTGTCCCAGGACGACTTAATCAAACTGCTCTCCTCGCCTCACGCCCAGGAGTGTACTATGGACAATGCTCAGAAATTTGCGGGGCCAACCACAGCTTTATACCAATTGTAATTGAAACAGTGCCCCTACCACACTTCGAAACTTGGTCCGCACACCAACTAGACTTCGCC

>A._jishouensis

ATGGCACATCCAACCCAATTAGGATTCCAAGACGCAGCATCACCAGTAATAGAAGAACTTCTTCATTTCCACGATCACGCACTAATAATTGTATTCTTAATTAGTGCTCTAGTACTATATATTATTATTGCAATGGTATCCACTAAACTTACTAATAAGCTTATCCTAGACTCTCAAGAAATCGAAATTGTGTGAACTATTTTACCCGCTGTAATTTTAGTATTAATTGCCCTACCCTCCCTACGTATTCTTTATCTTATAGACGAAATCAGTGACCCTCACCTAACAATTAAAGCAATAGGACACCAATGATACTGAAGCTACGAATATACGGACTATGAAAACCTAAATTTTGATGCCTACATAGTTCCAACCCAAGACCTTACCCCAGGACAATTTCGACTCCTAGAAACAGACCACCGGATAGTTATCCCAGTAGAATCTCCAATCCGCATTCTAGTTTCTGCCGAAGACGTACTACACTCTTGAGCTGTTCCCTCCATGGGCGTAAAAATAGATGCAGTCCCAGGACGACTTAATCAAACTGCCCTCCTTGCTTCACATCCAGGAGTATACTACGGACAATGCTCAGAAATTTGCGGAGCCAACCACAGCTTTATACCAATTGTAATTGAAGCAGTACCCCTACCACACTTCGAAACTTGGTCCGCATGTCAACTAGACCTTGCT

>A._kreyenbergii_1

ATGGCACACCCAACACAGCTGGGATTTCAAGACGCGGCGTCACCTGTCATAGAAGAACTTCTTCATTTCCACGACCACGCACTAATAATCGTATTCTTAATTAGTGCTCTAGTATTATATATTATTATTGCAATAGTATCCACCAAACTTACTAATAAATTTATTTTAGACTCTCAAGAAATCGAAATTGTGTGAACTATTCTACCTGCTGTAATTTTAGTGTTAATTGCCTTACCCTCTTTACGCATTCTGTACCTTATAGACGAAATCAATGACCCTCACTTAACAATTAAAGCAATAGGACACCAATGATATTGAAGCTACGAATATACGGACTATGAAAACCTAAATTTTGATGCCTACATAGTACCAACTCAAGACCTTACCCCAGGACAATTTCGGCTCCTAGAAACAGACCACCGAATAGTCATCCCAGTAGAGTCTCCGATCCGCATTCTAGTTTCTGCTGAAGACGTACTACACTCTTGAGCTATCCCCTCCATGGGCGTAAAAATAGATGCAGTCCCAGGACGACTTAATCAAACTGCCCTCCTTGCTTCTCGCCCAGGAGTATATTACGGACAATGCTCGGAAATTTGCGGAGCCAACCACAGCTTTATACCAATTGTAGTTGAAGCAGTACCTCTACCACACTTTGAAACTTGGTCCGCATGTCAACTAGACCTTGCC

>A._kreyenbergii_2

ATGGCACACCCAGCCCAACTAGGATTCCAAGACGCAGCATCACCAGTCATAGAAGAACTTCTTCATTTCCACGACCACGCACTAATAATCGTATTCTTAATTAGTGCTCTAGTATTATATATTATTATTGCAATAGTATCCACCAAACTTACTAATAAATTTATTTTAGACTCTCAAGAAATCGAAATTGTGTGAACTATTCTACCTGCTGTAATTTTAGTATTAATTGCCCTACCCTCCTTACGCATTCTATACCTTATAGACGAAATCAATGACCCTCACTTAACAATTAAAGCAATAGGACACCAATGATATTGAAGCTACGAATATACGGACTATGAAAACCTAAATTTTGATGCCTACATAGTACCGACTCAAGACCTTACCCCAGGACAATTTCGACTCCTAGAAACAGACCACCGAATAGTCATCCCAGTAGAATCTCCAATCCGCATTCTAGTTTCTGCTGAAGACGTACTACACTCTTGAGCTATCCCCTCCATGGGCGTAAAAATAGATGCGGTCCCAGGACGACTTAATCAAACTGCCCTCCTTGCTTCCCACCCAGGAGTATATTATGGACAATGCTCGGAAATTTGCGGAGCCAACCACAGCTTTATACCAATTGTAGTTGAAGCAGTACCCCTACCACACTTCGAAACTTGGTCCGCATGTCAACTAGACCTTGCC

>A._longipinnis

ATGGCACATCCAACACAACTAGGATTCCAAGACGCAGCATCACCAGTCATAGAAGAGCTTCTTCACTTCCACGATCACGCACTAATAATTGTATTTCTAATTAGCGCCCTAGTATTATATATTATTATTGCAATAGTATCCACCAAACTCACTAATAAGTTTATTTTAGACTCCCAAGAAATCGAAATCGTATGAACTATTTTACCCGCCGTAATTTTAGTAATAATTGCCCTACCTTCCCTACGCATTCTTTACCTTATAGACGAAATCAACGACCCCCACCTAACAATTAAAGCAATAGGACATCAATGATACTGAAGCTACGAATACACTGACTATGAAAACCTAAACTTCGACGCCTATATAGTACCAACCCAAGACCTTACCCCAGGGCAATTCCGACTTCTAGAAACAGATCACCGAATAGTTATTCCAGTAGAATCCCCAATCCGAATTCTGGTGTCTGCCGAAGACGTGTTACACTCTTGAGCTGTTCCATCTATGGGCGTAAAAATAGATGCTGTCCCAGGACGACTTAATCAAACTGCTCTCCTCGCCTCACGCCCAGGAGTGTACTATGGACAATGCTCAGAAATTTGCGGGGCCAACCACAGCTTTATACCAATTGTAATTGAAACAGTACCCCTACCACACTTCGAAACTTGGTCCGCACACCAACTAGACTTCGCC

>A._monticola_1

ATGGCACATCCAGCACAACTAGGATTCCAAGACGCAGCATCACCAGTCATAGAAGAGCTTCTTCACTTCCACGACCACGCACTAATAATTGTATTCCTAATTAGCGCCCTAGTATTATATATTATCATTGCAATGGTATCTACTAAACTCACTAATAAATTTATTCTAGACTCCCAAGAAATCGAAATCGTATGAACCATCTTACCCGCTGTAATTTTAGTCATAATTGCCCTACCCTCCCTACGCATTCTATATCTCATAGACGAAATCAACGACCCCCACCTAACAATTAAAGCAATAGGACATCAATGATACTGAAGCTACGAATATACAGACTACGAAGACCTAGCCTTCGATGCCTATATAGTACCAACCCAAGATCTTACCCCAGGACAATTTCGACTCCTAGAGACAGACCACCGAATAGTTATCCCAGTACAATCCCCCATTCGTATTCTAGTATCTGCCGAAGATGTACTACACTCTTGAGCTGTTCCATCTATGGGTGTAAAAATGGATGCAGTCCCAGGACGACTTAACCAAACTGCTCTCCTTGCCTCACGCCCAGGGGTGTACTATGGACAATGCTCAGAAATCTGCGGGGCTAACCATAGCTTTATACCAATTGTGATCGAAACAGTACCACTACCAAACTTCATGCTCTGATCTACATACCCTGCAACCTCTGCC

>A._monticola_2

ATGGCACATCCAGCACAACTAGGATTCCAAGACGCAGCATCACCAGTCATAGAAGAGCTTCTTCACTTCCACGACCACGCACTAATAATTGTATTCCTAATTAGCGCCCTAGTATTATATATTATCATTGCAATGGTATCTACTAAACTCACTAATAAATTTATTCTAGACTCCCAAGAAATCGAAATCGTATGAACCATCTTACCCGCTGTAATTTTAGTCATAATTGCCCTACCCTCCCTACGCATTCTATATCTCATAGACGAAATCAACGACCCCCACCTAACAATTAAAGCAATAGGACATCAATGATACTGAAGCTACGAATATACAGACTACGAAGACCTAGCCTTCGATGCCTATATAGTACCAACCCAAGATCTTACCCCAGGACAATTTCGACTCCTAGAGACAGACCACCGAATAGTTATCCCAGTACAATCCCCCATTCGTATTCTAGTATCTGCCGAAGATGTACTACACTCTTGAGCTGTTCCATCTATGGGTGTAAAAATGGATGCAGTCCCAGGACGACTTAACCAAACTGCTCTCCTTGCCTCACGCCCAGGGGTGTACTATGGACAATGCTCAGAAATCTGCGGGGCTAACCATAGCTTTATACCAATTGTGATCGAAACAGTACCACTACCAAACTTCATGCTCTGATCTACATACCCTGCAACCTCTGCC

>A._paradoxus_1

ATGGCCCATCCAACACAACTAGGATTCCAAGACGCAGCATCACCAGTCATAGAAGAACTTCTTCACTTCCACGACCACGCACTAATAATTGTATTCTTAATTAGTGCCCTAGTACTATATATTATTATTGCAATGGTATCCACCAAGCTTACTAATAAATTTATTTTAGACTCTCAAGAAATTGAAATTGTATGAACTATTTTACCCGCCGTAATCTTAGTAATAATCGCCCTGCCCTCCCTACGCATTCTATACCTTATAGACGAAATCAGCGACCCCCACCTAACAATTAAAGCAATAGGACACCAATGATACTGAAGCTACGAATATACAGACTACGAAGACCTAAACTTTGATGCTTACATAGTGCCAACCCAAGACCTCACCCCAGGACAATTTCGGCTCCTGGAAACAGACCACCGAGTAGTTATTCCAGTGGAATCCCCAATTCGTATCCTGGTATCTGCCGAAGACGTACTACACTCTTGAGCCGTCCCCTCCATAGGCGTAAAAATAGATGCAGTTCCAGGACGACTTAACCAGACTGCCATCCTCGCTTCGCGCCCAGGAACATACTATGGACAATGCTCAGAAATTTGCGGGGCCAACCACAGCTTCATACCAATTGTAATTGAAGCCGTACCCCTACCACAGTTTGAAATTTGATCCGCATACCAATTAGACCTCGCC

>A._paradoxus_2

ATGGCACATCCAACCCAATTAGGATTCCAAGACGCAGCATCACCAGTAATAGAAGAACTTCTTCATTTCCACGATCACGCACTAATAATTGTATTCTTAATTAGTGCTCTAGTACTATATATTATTATTGCAATGGTATCCACTAAACTTACTAATAAGCTTATCCTAGACTCTCAAGAAATCGAAATTGTGTGAACTATTTTACCCGCTGTAATTTTAGTATTAATTGCCCTACCCTCCCTACGTATTCTTTATCTTATAGACGAAATCAGTGACCCTCACCTAACAATTAAAGCAATAGGACACCAATGATACTGAAGCTACGAATATACGGACTATGAAAACCTAAATTTTGATGCCTACATAGTTCCAACCCAAGACCTTACCCCAGGACAATTTCGACTCCTAGAAACAGACCACCGGATAGTTATCCCAGTAGAATCTCCAATCCGCATTCTAGTTTCTGCCGAAGACGTACTACACTCTTGAGCTGTTCCCTCCATGGGCGTAAAAATAGATGCAGTCCCAGGACGACTTAATCAAACTGCCCTCCTTGCTTCACATCCAGGAGTATACTACGGACAATGCTCAGAAATTTGCGGAGCCAACCACAGCTTTATACCAATTGTAATTGAAGCAGTACCCCTACCACACTTCGAAACTTGGTCCGCATGTCAACTAGACCTTGCT

>A._parallens_1

ATGGCACACCCAGCCCAACTAGGATTCCAAGACGCAGCATCACCAGTCATAGAAGAACTTCTCCACTTCCACGATCACGCACTAATAATTGTATTCTTAATTAGTGCCCTAGTACTATACATTATTGTTGCAATAGTATCCACCAAACTTACCAATAAACTTATCTTAGACTCTCAAGAAATCGAAATTGTGTGAACTATTTTACCCGCTGTAATTTTAGTATTAATTGCCCTACCCTCCCTACGCATTCTATACCTTATAGACGAAATCAATGACCCTCACCTAACAATTAAAGCAATGGGACACCAATGATACTGAAGCTACGAATATACGGACTATGAAAACCTAAATTTTGATGCCTACATAGTTCCTACCCAAGACCTCACCCCAGGACAATTTCGACTCCTAGAAACAGACCACCGAATAGTCATCCCAGTAGAATCTCCAATCCGCATTCTAGTCTCTGCCGAAGACGTGCTACACTCTTGAGCTGTTCCCTCCATGGGCGTGAAGATAGATGCAGTCCCAGGACGACTTAATCAAACCGCCCTCCTTGCTTCACACCCAGGAGTATACTACGGGCAATGCTCAGAAATTTGCGGAGCCAACCACAGCTTTATACCAATTGTAATTGAAGCGGTACCCCTACCACACTTCGAAACTTGGTCCGCATGTCAACTAGACCTTGCT

>A._parallens_2

ATGGCACACCCAGCCCAACTAGGATTCCAAGACGCAGCATCACCAGTTATAGAAGAACTTCTCCACTTCCACGATCACGCACTAATAATTGTATTCTTAATTAGTGCCCTAGTACTATACATTATTATTGCAATAGTATCCACCAAACTTACCAATAAACTTATCTTAGACTCTCAAGAAATCGAAATTGTGTGAACTATTTTACCCGCTGTAATTTTAGTATTAATTGCCCTACCCTCCCTACGCATTCTATACCTTATAGACGAAATCAATGACCCTCACCTAACAATTAAAGCAATAGGACACCAATGATACTGAAGCTACGAATATACGGACTATGAAAACCTAAATTTTGATGCCTACATAGTTCCTACCCAAGACCTCACCCCAGGACAATTTCGACTCCTAGAAACAGACCACCGAATAGTCATCCCAGTAGAATCTCCAATCCGCATTCTAGTTTCTGCCGAAGACGTGCTACACTCTTGAGCTGTTCCCTCCATGGGCGTGAAAATAGATGCGGTCCCAGGACGACTTAATCAAACCGCCCTCCTTGCTTCACACCCGGGAGTATACTACGGGCAATGCTCAGAAATTTGCGGAGCCAACCACAGCTTTATACCAATTGTAATTGAAGCAGTACCCCTACCACACTTCGAAACTTGGTCCGCATGTCAACTGGATCTTGCT

>A._parallens_3

ATGGCACACCCAGCCCAACTAGGATTCCAAGACGCAGCATCACCAGTCATAGAAGAACTTCTCCACTTCCACGATCACGCACTAATAATTGTATTCTTAATTAGTGCCCTAGTACTATACATTATTATTGCAATAGTATCCACCAAACTTACCAATAAACTTATCTTAGACTCTCAAGAAATCGAAATTGTGTGAACTATTTTACCCGCTGTAATTTTAGTATTAATTGCCCTGCCCTCCCTACGCATTCTATACCTTATAGACGAAATCAATGACCCTCACCTAACAATTAAAGCAATAGGACACCAATGATACTGAAGCTACGAATATACGGACTATGAAAACCTAAATTTTGATGCCTACATAGTTCCTACCCAAGACCTCACCCCAGGACAATTTCGACTCCTAGAAACAGACCACCGAATAGTCATCCCAGTAGAATCTCCAATCCGCATTCTAGTCTCTGCCGAAGACGTGCTACACTCTTGAGCTGTTCCCTCCATGGGCGTGAAGATAGATGCAGTCCCAGGACGACTTAATCAAACCGCCCTCCTTGCTTCACACCCAGGAGTATACTACGGGCAATGCTCAGAAATTTGCGGAGCCAACCACAGCTTTATACCAATTGTAATTGAAGCAGTACCCCTACCACACTTCGAAACTTGGTCCGCATGTCAACTAGACCTTGCT

>A._parallens_4

ATGGCACACCCAGCCCAACTAGGATTCCAAGACGCAGCATCACCAGTCATAGAAGAACTTCTCCACTTCCACGATCACGCACTAATAATTGTATTCTTAATTAGTGCCCTAGTACTATACATTATTATTGCAATAGTATCCACCAAACTTACCAATAAACTTATCTTAGACTCTCAAGAAATCGAAATTGTGTGAACTATTTTACCCGCTGTAATTTTAGTATTAATTGCCCTGCCCTCCCTACGCATTCTATACCTTATAGACGAAATCAATGACCCTCACCTAACAATTAAAGCAATAGGACACCAATGATACTGAAGCTACGAATATACGGACTATGAAAACCTAAATTTTGATGCCTACATAGTTCCTACCCAAGACCTCACCCCAGGACAATTTCGACTCCTAGAAACAGACCACCGAATAGTCATCCCAGTAGAATCTCCAATCCGCATTCTAGTCTCTGCCGAAGACGTGCTACACTCTTGAGCTGTTCCCTCCATGGGCGTGAAGATAGATGCAGTCCCAGGACGACTTAATCAAACCGCCCTCCTTGCTTCACACCCAGGAGTATACTACGGGCAATGCTCAGAAATTTGCGGAGCCAACCACAGCTTTATACCAATTGTAATTGAAGCAGTACCCCTACCACACTTCGAAACTTGGTCCGCATGTCAACTAGACCTTGCT

>A._spinifer

ATGGCCCATCCAACACAACTAGGATTCCAAGACGCAGCATCACCAGTCATAGAAGAACTTCTTCACTTCCACGATCACGCACTAATAATTGTATTCTTAATTAGTGCCCTAGTACTATATATTATTATTGCAATAGTCTCCACCAAACTTACCAATAAGTTTATTTTAGACTCCCAAGAAATTGAAATTGTATGAACTATTTTACCTGCTGTAATTTTAGTAATAATTGCCCTACCCTCCTTACGCATTCTATACCTTATAGACGAAATCAGCGACCCTCACCTAACAATTAAAGCAATAGGACACCAATGATACTGAAGCTACGAATATACAGACTATGAAGACCTAAACTTTGATGCTTATATAGTACCAACCCAAGACCTCACCCCAGGACAATTCCGACTCCTAGAAACAGACCACCGAGTAGTTATTCCAGTGGAATCCCCCATTCGCATTCTAGTATCTGCCGAAGACGTACTACACTCTTGAGCCGTCCCCTCCATGGGCGTAAAAATAGACGCAGTCCCAGGGCGACTTAACCAAACTGCCATCCTCGCTTCACGCCCAGGAGCATACTATGGGCAATGCTCAGAAATTTGCGGAGCCAACCACAGCTTTATACCAATTGTAATTGAAGCAGTACCCCTACCACAATTTGAAACTTGATCCGCATACCAACTAGACCTCGCC

>A._stenotaeniatus

ATGGCCCATCCAACACAACTAGGATTCCAAGACGCAGCATCACCAGTCATAGAAGAACTTCTTCACTTCCACGATCACGCACTAATAATTGTATTCTTAATTAGTGCCCTAGTACTATATATTATTATTGCAATAGTCTCCACCAAACTTACCAATAAGTTTATTTTAGACTCCCAAGAAATTGAAATTGTATGAACTATTTTACCTGCTGTAATTTTAGTAATAATTGCCCTACCCTCCTTACGCATTCTTTACCTTATAGACGAAATCAGCGACCCCCACCTAACAATTAAAGCAATAGGACACCAATGATACTGAAGCTACGAATATACAGACTATGAAGACCTAAACTTTGATGCTTATATAATACCAACCCAAGACCTCACCCCAGGACAATTCCGACTCCTAGAAACAGACCACCGAGTAGTTATTCCAGTGGAATCCCCCATTCGCATTCTAGTATCTGCCGAAGACGTACTACACTCTTGAGCCGTCCCCTCCATGGGCGTAAAAATAGACGCAGTCCCAGGGCGACTTAACCAAACTGCCATCTTCACTTCACGCCCAGGGGCATACTATGGGCAATGCTCAGAAATTTGCGGAGCCAACCACAGCTTTATACCAATTGTAATTGAAGCAGTACCCCTACCACAATTTGAAACTTGATCCGCATACCAACTAGACCTCGCC

>A._wenchowensis_1

ATGGCACATCCAGCCCAACTAGGATTCCAAGACGCAGCATCACCAGTCATAGAAGAACTTCTTCACTTCCACGACCACGCACTAATAATCGTATTCTTAATTAGTGCTCTAGTATTATATATTATTATTGCAATAGTATCCACCAAACTTACTAATAAGTTTATTTTAGACTCTCAAGAAATCGAAATTGTGTGAACTATTTTACCTGCCGTAATTTTAGTGTTAATTGCCCTACCCTCTCTACGCATCCTATACCTTATAGACGAAATCAATGACCCCCACCTAACAATTAAAGCAATAGGACACCAATGATACTGAAGTTACGAATACACAGACTATGAAAACCTAAATTTTGATGCCTACATAGTACCAACTCAAGACCTCACCCCAGGACAATTTCGACTTCTAGAAACAGACCACCGAATAGTCATCCCAGTAGAATCTCCAATCCGCATTCTAGTTTCTGCCGAAGACGTACTACACTCTTGAGCTATCCCCTCCATGGGCGTAAAAATAGATGCAGTCCCTGGACGACTTAATCAAACTGCCCTCCTTGCTTCACACCCAGGAGTATACTACGGACAATGCTCAGAAATTTGCGGAGCCAACCACAGCTTCATACCAATTGTAGTTGAAGCAGTACCCCTACCACACTTCGAAACTTGGTCCGCATGTCAACTAGACCTTGCC

>A._wenchowensis_2

ATGGCACATCCAGCCCAACTAGGATTCCAAGACGCAGCATCACCAGTCATAGAAGAACTTCTCCACTTCCACGACCACGCACTAATAATCGTATTCTTAATTAGTGCTCTAGTGTTATATATTATTATTGCAATAGTATCCACCAAACTTACTAATAAATTTATCTTAGACTCTCAGGAAATCGAAATTGTGTGAACCATTTTACCTGCCGTAATTTTAGTGTTAATTGCCCTGCCCTCTCTACGCATTCTATACCTTATAGACGAAATCAATGACCCCCACCTAACAATTAAAGCAATGGGACACCAATGATACTGAAGCTACGAGTATACGGACTATGAAAACCTAAATTTTGATGCCTACATAGTACCAACTCAAGACCTCACCCCAGGACAATTTCGACTCCTAGAAACAGACCACCGAATAGTCATCCCAGTAGAATCTCCAATCCGCATTCTAGTTTCTGCTGAAGACGTACTACACTCTTGAGCTATCCCTTCCATAGGCGTAAAAATAGATGCGGTCCCAGGACGACTTAATCAAACTGCCCTCCTTGCTTCACACCCAGGAGTATACTACGGACAATGCTCAGAAATTTGCGGAGCCAATCACAGCTTTATACCAATTGTAGTTGAAGCAGTACCCCTACCACACTTCGAAACTTGGTCCGCATGTCAACTAGACCTTGCC

>A._wuyiensis

ATGGCCCATCCAACACAACTAGGATTCCAAGACGCAGCATCACCAGTCATAGAAGAACTTCTTCACTTCCACGACCACGCACTAATAATTGTATTCTTAATTAGTGCCCTAGTACTATATATTATTATTGCAATGGTATCCACCAAGCTTACTAATAAATTTATTTTAGACTCTCAAGAAATTGAAATTGTATGAACTATTTTACCCGCTGTAATCTTAGTAATAATCGCCCTGCCCTCCCTACGCATTCTATACCTTATAGACGAAATCAGCGACCCTCACCTAACAATTAAAGCAATAGGACACCAATGATACTGAAGCTACGAATATACAGACTACGAAGACCTAAACTTTGATGCTTACATAGTGCCAACCCAAGACCTCACCCCAGGACAATTTCGGCTCCTGGAAACAGACCACCGAGTAGTTATTCCAGTGGAATCCCCAATTCGTATCCTGGTATCTGCCGAAGACGTACTACACTCTTGAGCCGTCCCCTCCATAGGCGTAAAAATAGATGCAGTTCCAGGACGACTTAACCAAACTGCCATCCTCGCTTCGCGCCCAGGAACATACTATGGACAATGCTCAGAAATTTGCGGGGCCAACCACAGCTTCATACCAATTGTAATTGAAGCCGTACCCCTACCACAGTTTGAAATTTGATCCGCATACCAATTAGACCTCGCC

>A._yunnanensis_1

ATGGCACATCCAACACAACTAGGATTCCAAGACGCAGCATCACCAGTCATAGAAGAACTTCTTCACTTCCACGACCACGCACTAATAATTGTATTCCTAATCAGCGCCCTAGTATTATATATTATTATTGCAATGGTATCTACCAAACTCACCAATAAATTTATTTTAGACTCCCAAGAAATCGAAATCGTATGAACTATTTTACCAGCTGTAATTTTAGTCATAATTGCCCTGCCCTCCCTACGCATTCTATATCTTATAGACGAAATCAATGACCCTCACCTAACAATTAAAGCAATAGGACATCAATGATACTGAAGCTACGAATATACAGATTACGAAGACCTAGCCTTCGATGCCTATATAGTACCAACCCAAGATCTTGCCCCAGGACAATTTCGACTCCTAGAAACAGACCACCGAATAGTTATTCCAGTACAATCCCCAATTCGTATCCTAGTATCTGCCGAAGATGTACTACACTCTTGAGCTGTTCCATCTATGGGCGTAAAAATAGATGCGGTCCCAGGACGACTTAACCAAACCGCCCTCCTCTCATCACGCCCAGGGGTGTACTACGGACAATGCTCAGAAATCTGCGGGGCCAACCACAGCTTTATACCAATTGTAATTGAAACAGTACCTCTACTAAACTTCATACTTTGATCTACATACCCTGTAGTCTCTGCC

>A._yunnanensis_2

ATGGCACATCCAACACAACTAGGATTCCAAGACGCAGCATCACCAGTCATAGAAGAACTTCTTCACTTCCACGACCACGCACTAATAATTGTATTCCTAATCAGCGCCCTAGTATTATATATTATTATTGCAATGGTATCTACCAAACTCACCAATAAATTTATTTTAGACTCCCAAGAAATCGAAATCGTATGAACTATTTTACCCGCTGTAATTTTAGTTATAATTGCCCTACCCTCCCTACGCATTCTATATCTTATAGACGAAATCAATGACCCTCACCTAACAATTAAAGCAATAGGACATCAATGATACTGAAGCTACGAGTATACAGATTACGAAGACCTAGCCTTCGATGCTTATATAGTACCAACCCAAGATCTTGCCCCAGGACAATTTCGACTCCTAGAAACAGACCACCGAATAGTCATCCCAGTACAATCCCCAATTCGTATCCTAGTATCTGCCGAAGATGTGCTACACTCTTGAGCTGTTCCATCTATGGGCGTAAAAATAGATGCGGTCCCAGGACGACTCAACCAAACCGCCCTCCTCTCATCACGCCCAGGGGTGTACTACGGACAATGCTCAGAAATCTGCGGGGCCAACCACAGCTTTATACCAATTGTAATTGAAACAGTACCTCTACTAAACTTCATACTTTGATCTACATACCCCCTAGTCTCTGCC

>Onychostoma_barbatulum

ATGGCACATCCAGCACAACTAGGATTCCAAGACGCAGCATCACCAGTCATAGAAGAACTTCTTCACTTCCACGATCACGCACTAATAATTGTGTTCCTAATTAGCACCCTGGTATTATATATTATTATTGCAATAGTATCTACTAAACTCACTAATAAACTTATCTTAGACTCTCAAGAAATCGAAATCGTATGAACTATTTTACCCGCTGTAATTTTAGTAATAATTGCCCTACCCTCCCTACGCATTCTATATCTTATAGACGAAATCAATGACCCCCACCTAACAATCAAAGCAATGGGACACCAATGATACTGAAGCTATGAATATACAGATTATGAAAACCTAAGCTTTGACGCCTACATGATATCAACCCAAGACCTCTCCCCAGGACAATTTCGACTCCTAGAAACAGACCATCGAACAGTTATTCCAGTAGAATCCCCAATTCGTATCTTAGTATCTGCCGAAGACGTACTACACTCTTGAGCTGTTCCATCCATGGGTGTAAAAATAGATGCTGTTCCAGGGCGACTAAACCAAACCACCCTCCTCGCCTCACGCCCAGGGGTGTACTACGGACAATGCTCAGAAATTTGTGGAGCCAACCACAGCTTTATGCCAATTGTAATTGAAGCAGTACCTCTTCCACACTTTGAAACTTGATCCGCACACCAGCTAGACTTCGCC

>Onychostoma_meridionale

ATGGCACATCCGACACAACTAGGATTCCAAGACGCAGCATCACCAGTCATAGAAGAACTTCTTCACTTCCACGACCACGCATTAATAATTGTATTCCTAATTAGCGCCCTAGTACTATATATTATTATTGCTATAGTGTCTACTAAACTCACTAACAAATTTATCTTAGACTCTCAAGAAATCGAAATCGTATGAACTATTTTACCCGCTGTAATTTTAGTATTAATCGCCCTGCCCTCCCTACGAATCTTATATCTTATAGACGAAATTAACGACCCCCACTTAACAATTAAAGCAATAGGACATCAATGATACTGAAGCTATGAGTATACAGACTACGAAAACCTAAACTTCGACGCTTACATGGTGCCGACCCAAGACCTCACCCCTGGCCAATTTCGACTTCTAGAAACAGACCACCGAATAGTCATCCCGGTAGAATCTCCAATTCGTATTCTAGTGTCTGCCGAAGACGTGCTGCACTCCTGAGCCGTCCCTTCCATGGGCGTAAAAATGGACGCAGTTCCAGGACGACTCAACCAAACCGCCCTCCTCGCCTCACGACCAGGGGTATACTACGGGCAATGCTCAGAAATTTGTGGAGCCAACCACAGCTTTATACCAATTGTAATCGAAACTGTACCACTGCCATATTTCGAAACTTGATCCGCACATCAACTAGACTTCGCC

>Onychostoma_gerlachi

ATGGCACATCCGACACAACTAGGATTCCAAGACGCAGCATCACCAGTCATAGAAGAACTTCTTCACTTCCACGACCACGCATTAATAATTGTATTCCTAATTAGCGCCCTAGTACTATATATTATTATTGCGATAGTGTCTACTAAACTCACTAACAAGTTTATCTTAGATTCTCAAGAAATCGAAATCGTATGAACTATTTTACCCGCTGTAATTTTAGTGTTAATCGCCCTACCTTCCCTACGAATCTTATATCTTATAGACGAAATTAATGACCCCCACTTAACAATTAAAGCGATAGGACATCAATGATACTGAAGCTATGAATACACAGACTATGAAAACCTAAACTTTGACGCCTACATGGTGCCAACCCAAGACCTCACCCCCGGCCAATTTCGGCTTCTAGAAACAGACCACCGGATAGTCATCCCGGTAGAATCCCCAATTCGTATTCTAGTGTCTGCCGAAGACGTGCTACACTCCTGAGCCGTTCCATCCATAGGCGTAAAAATGGACGCAGTTCCGGGGCGACTCAACCAAACCGCCCTCCTCGCCTCACGTCCAGGAGTATACTACGGACAATGCTCAGAGATTTGTGGGGCCAACCACAGCTTTATACCAATTGTAATCGAAACTGTACCACTCCCACACTTCGAAACTTGGTCCGCACATCAACTAGACTTCGCC

>Spinibarbus_denticulatus

ATGGCACATCCAACACAACTAGGATTCCAAGACGCGGCATCACCCGTTATAGAAGAACTTCTTCATTTTCATGACCACGCACTAATAATTGTATTTCTAATCAGCACCTTAGTATTATATATTATTATCGCAATGGTATCCACCAAACTTACTAATAAATATATTTTAGACTCCCAAGAAATCGAAATTGTATGAACTATCTTACCGGCCGTTATTTTAGTATTAATCGCCCTGCCCTCCCTTCGTATTCTATACCTTATAGATGAAATTAATGACCCCCACCTAACAATTAAAGCAATAGGTCATCAATGGTACTGAAGCTACGAATATACAGATTATGAAAACCTGGGCTTTGACTCCTACATGGTCCCAACCCAAGACCTTACCCCAGGACAATTCCGACTTCTAGAAACAGACCACCGAATAGTTATTCCAATAGAATCCCCAATCCGTGTTCTAGTATCTGCTGAAGACGTCCTACACTCTTGAGCTGTCCCATCCCTGGGCGTAAAAATGGACGCAGTCCCAGGACGACTTAACCAAACCGCCTTCATTGCCTCGCGCCCAGGAGTATTTTACGGACAATGCTCTGAAATCTGCGGAGCTAATCACAGCTTTATACCAATTGTAGTTGAAGCAGTACCGCTAGAATACTTCGAAAACTGATCCTCATTAATACTAGAAGACGCC

>Spinibarbus_hollandi

ATGGCACATCCAACACAACTAGGATTCCAAGATGCAGCATCACCCGTTATAGAAGAACTTCTTCATTTTCATGACCACGCACTAATAATTGTATTTCTAATCAGCACCTTAGTACTATATATCATTATCGCAATGGTATCTACCAAACTTACTAATAAATATATTTTAGATTCCCAAGAAATCGAAATTGTATGAACCATCTTACCAGCCGTTATTTTAGTATTAATTGCCCTACCCTCCCTTCGTATCCTGTACCTTATAGATGAAATTAATGACCCCCACCTAACAATTAAAGCAATAGGTCATCAATGATACTGAAGCTACGAATATACAGATTATGAAAATCTGGGCTTTGACTCCTACATGACCCCGACCCAAGACCTTACCCCAGGACAATTCCGACTTTTAGAAACAGACCACCGAATAGTTATTCCAATAGAATCCCCAATCCGTGTCCTAGTATCTGCTGAAGACGTCCTACACTCTTGAGCTGTCCCATCCCTGGGCGTAAAAATGGACGCAGTCCCAGGACGGCTTAACCAAACTGCCTTCATTGCCTCGCGCCCAGGAGTATTTTATGGACAATGCTCTGAAATCTGCGGAGCTAATCACAGCTTTATACCAATTGTAGTTGAAGCAGTACCGCTAGAATACTTCGAAAACTGATCCTCATTAATACTAGAAGACGCC

>Spinibarbus_sinensis

ATGGCACATCCAACACAACTAGGATTCCAAGACGCGGCATCACCCGTTATAGAAGAACTTCTTCACTTTCATGACCACGCACTAATAATTGTATTTCTAATTAGCACCTTGGTGTTATACATTATTATTGCAATGGTATCTACCAAACTTACTAATAAATATATTTTAGACTCTCAAGAAATCGAAATTGTATGAACTATCCTACCGGCTGTTATTTTAGTATTAATTGCCCTCCCCTCCCTACGTATTTTATATCTTATAGACGAAATTAATGACCCCCACCTGACAATTAAAGCAATAGGACATCAGTGATACTGAAGCTACGAGTACACAGATTATGAAAATCTAGGCTTTGACTCCTATATGGTACCAACCCAAGACCTCGCCCCAGGACAATTCCGGCTGCTAGAAACAGACCATCGAATGGTTATCCCAATAGAATCTCCAGTCCGTGTCCTAGTATCTGCTGAAGACGTACTACACTCTTGAGCTGTCCCATCTCTAGGCGTAAAAATGGATGCGGTCCCAGGACGACTTAACCAAACCGCCTTCATCGCCTCACGTCCAGGAGTATTTTATGGACAATGCTCTGAAATCTGTGGAGCCAACCACAGCTTTATACCAATTGTAGTTGAGGCAGTACCACTGGAACACTTCGAAAACTGATCCTCATTAATACTAGAAGACGCC

**COX3**

>A._barbodon

ATGGCCCACCAAGCACATGCCTACCATATAGTTGACCCAAGCCCATGACCACTGACCGGGGCTATTGCTGCCCTACTAATAACATCAGGCCTAGCAATCTGATTCCACTTCCACTCAACAACACTAATAACCTTAGGAATAATTCTTCTCCTTCTTACTATATATCAATGATGACGAGATATTATCCGGGAAGGAACCTTCCAAGGACACCACACACCCCCAGTACAAAAAGGACTGCGATATGGAATAATCCTATTTATTACCTCTGAAGTATTCTTTTTCCTCGGGTTCTTCTGAGCTTTTTACCACTCAAGTCTGGCACCAACACCTGAGCTGGGAGGATGCTGGCCTCCCACAGGAATCATCCCACTAGACCCCTTTGAAGTACCACTCCTTAACACAGCCGTACTATTAGCATCAGGGGTCACAGTAACATGAGCCCACCATAGTATTATGGAAGGGGGACGAAAACAAGCTATCCAATCTCTAACATTAACCATCCTACTAGGACTTTACTTTACCGCACTCCAAGCCATAGAGTACTACGAAGCACCCTTCACAATCGCAGACGGAGTCTACGGCTCAACATTCTTTGTGGCTACAGGATTCCACGGACTACACGTTATTATCGGATCAACTTTTCTAGCAGTATGCCTACTACGCCAAGCCCAATATCACTTCACATCTGAACATCACTTTGGTTTCGAAGCCGCTGCTTGATACTGACACTTCGTCGACGTAGTATGACTATTCCTCTACGTATCTATCTACTGATGAGGCTCA

>A._beijiangensis_1

ATGGCCCATCAAGCACACGCCTACCACATAGTTGACCCAAGCCCATGACCACTAACCGGGGCTATTGCTGCCTTACTAATAACATCAGGCCTGGCAACCTGATTCCACTTCCACTCAACAACACCAATAACTTTAGGTATAATTCTTCTACTTCTTACCATATACCAATGATGACGTGACATCATCCGGGAGGGAACCTTCCAAGGCCACCACACACCCCCAGTACAAAAAGGACTACGATATGGAATAATCTTATTTATTACCTCTGAGGTATTCTTTTTCCTTGGGTTCTTTTGAGCTTTTTATCACTCCAGCCTGGCACCAACACCTGAACTAGGAGGATGCTGACCCCCCACAGGAATCCTCCCACTAGACCCCTTCGAAGTGCCACTTCTTAACACAGCCGTACTATTAGCCTCAGGGGTTACAGTAACATGAGCCCACCACAGTATCATGGAGGGAGAACGAGAACAAGCCCTCCAATCTTTAACACTAACTATCCTACTAGGACTTTATTTTACCTCACTCCAAGCCATCGAATACTACGAAGCACCCTTCACAATTGCAGACGGAGTCTACGGCTCAACATTTTTCGTAGCCACAGGGTTCCACGGACTACATGTTATTATTGGATCAACTTTCCTAGCAGTATGCCTTCTACGCCAAGCCCTGTACCACTTTACATCCGAACACCACTTTGGCTTCGAGGCCGCTGCCTGATACTGACACTTCGTCGACGTAGTATGACTATTCCTCTACGTATCCATCTACTGATGAGGCTCA

>A._beijiangensis_2

ATGGCCCATCAAGCACACGCCTACCACATAGTTGACCCAAGCCCATGGCCACTAACCGGGGCTATTGCTGCCTTACTAATAACATCAGGCCTGGTAACCTGATTCCACTTCCACTCAACAACACCAATAACTTTAGGTATAATTCTTCTACTTCTTACCATATACCAATGATGACGTGATATTATCCGGGAGGGAACCTTCCAAGGCCACCACACACCCCCAGTACAAAAAGGACTACGATATGGAATAATCTTATTTATTACCTCTGAGGTATTCTTTTTCCTTGGGTTCTTTTGAGCTTTTTATCACTCCAGCCTGGCACCAACACCCGAGCTGGGAGGATGCTGACCCCCCACAGGAATCCTCCCACTAGACCCCTTCGAAGTGCCACTTCTTAACACAGCCGTACTATTAGCCTCAGGGGTTACAGTAACATGAGCCCACCATAGTATCATGGAAGGAGAACGAGAACAAGCCCTCCAATCTTTAACACTAACTATCCTACTAGGACTTTATTTTACCGCACTCCAAGCCGTCGAATATTACGAAGCACCATTCACAATTGCAGACGGAGTCTACGGCTCAACATTTTTCGTAGCCACAGGGTTCCACGGACTACATGTTATTATTGGATCAACTTTCCTAGCAGTATGCCTTCTACGCCAAGCCCTGTACCACTTTACATCCGAACACCACTTTGGCTTCGAGGCCGCTGCCTGATACTGACACTTCGTCGACGTAGTATGACTATTCCTCTACGTATCCATCTACTGATGAGGCTCA

>A._fasciatus

ATGGCCCATCAAGCACACGCCTACCATATAGTTGACCCAAGCCCATGACCATTAACCGGAGCTATTGCTGCGCTATTAATAACATCAGGCCTAGCAATCTGATTCCATTTTCACTCAACAACACTAATAACTTTAGGAATAATTCTTCTACTCCTTACCATGTACCAGTGATGACGTGACATCATTCGAGAAGGGACCTTCCAAGGCCACCACACGCCCCCAGTACAAAAAGGATTACGATATGGAATAATTTTATTTATCACCTCTGAAGTATTCTTTTTCCTTGGATTCTTTTGAGCTTTTTATCACGCCAGCCTGGCACCAACACCTGAATTAGGAGCATGCTGACCCCCCTCAGGAATTATCCCACTAGACCCCTTTGAAGTACCACTCCTTAACACAGCCGTTCTATTAGCCTCGGGGGTTACAGTAACATGAGCCCATCATAGTATTATAGAAGGAGAACGAAAGCAAGCCATTCAATCTTTAGCACTAACCATCCTACTAGGACTTTATTTTACAGCACTCCAAGCCATGGAGTACTACGAAGCACCATTCACAATCGCAGATGGGGTCTACGGCTCAACATTTTTCGTAGCTACAGGATTCCACGGACTACATGTTATTATTGGGTCAACTTTCCTAGCAGTATGCCTTCTACGCCAAGCCCAATATCACTTTACGTCCGAACACCACTTTGGTTTTGAAGCCGCTGCCTGATACTGACACTTTGTCGACGTAGTATGACTATTCCTTTACGTATCCATCTACTGATGAGGCTCA

>A._hemispinus

ATGGCCCATCAAGCACACGCCTACCATATAGTTGACCCAAGCCCATGACCACTAACCGGAGCTATTGCTGCCCTACTAATAACATCAGGCCTAGCAATCTGATTCCATTTTCACTCAACAACACTAATATCTTTAGGAATAATTCTTCTACTCCTCACCATATACCAATGATGACGTGATATCATCCGAGAAGGGACCTTCCAAGGCCACCACACACCCCCAGTACAAAAAGGATTACGATACGGAATAATCTTATTTATCACCTCTGAAGTATTCTTTTTCCTCGGGTTCTTTTGAGCCTTTTATCACGCTAGCCTGGCACCAACACCCGAACTAGGAGCATGCTGACCCCCCTCAGGAATCATCCCACTAGACCCCTTTGAAGTACCACTCCTTAACACAGCCGTACTATTAGCCTCCGGGGTCACAGTAACATGAGCCCACCATAGTATTATAGAAGGAGAACGAAAACAAGCCATCCAATCTTTAGCACTAACCATTCTGCTAGGACTCTATTTTACCGCACTCCAAGCCATGGAGTATTACGAAGCACCATTCACAATTGCAGACGGAGTCTACGGCTCAACATTCTTCGTAGCTACAGGATTCCACGGACTACATGTTATTATTGGATCGACCTTCCTAGCAGTCTGCCTTTTACGCCAAGCCCAATATCACTTTACGTCCGAACACCACTTTGGTTTTGAGGCCGCTGCCTGATACTGACACTTTGTTGACGTAGTATGACTATTCCTTTACGTATCCATCTACTGATGAGGCTCA

>A._iridescens

ATGGCCCACCAAGCACATGCCTACCACATAGTTGACCCAAGCCCATGACCACTGACCGGGGCTATTGCTGCCCTACTAATAACATCAGGCCTAGCAATCTGATTCCACTTCCACTCAACAACACTAATAACCTTAGGAATAATTCTTCTACTTCTTACTATATATCAATGATGACGAGACATTATCCGGGAGGGAACCTTCCAAGGACACCACACACCCCCAGTACAAAAAGGACTACGATATGGAATAATCCTATTTATTACCTCTGAAGTATTCTTTTTCCTCGGGTTCTTCTGAGCTTTTTACCACTCAAGTCTGGCACCAACACCTGAACTGGGGGGATGCTGACCTCCCACAGGAATCATCCCACTAGACCCCTTTGAAGTACCACTCCTTAACACAGCTGTACTATTAGCATCAGGGGTCACAGTAACATGAGCCCACCACAGTATTATAGAAGGGGGACGAAAACAAGCTATCCAATCTCTAACATTAACCATCCTACTAGGACTTTATTTTACCGCACTCCAAGCCATAGAGTACTACGAAGCACCCTTCACAATCGCAGACGGAGTCTACGGCTCAACATTCTTTGTGGCTACAGGATTCCACGGACTACACGTTATTATTGGATCAACTTTCCTAGCAGTGTGCCTACTGCGCCAAGCCCAATACCACTTCACATCTGAACACCACTTTGGTTTCGAAGCCGCTGCTTGATACTGACATTTCGTCGACGTAGTATGACTATTCCTCTACGTATCTATCTACTGATGAGGCTCA

>A._jishouensis

ATGGCCCATCAAGCACACGCCTACCATATAGTTGACCCAAGCCCATGACCACTAACCGGAGCTATTGCTGCCCTATTAATAACATCAGGCCTAGCAATCTGGTTCCATTTTCACTCAACAACACTAATGACTCTAGGAATAATTCTTCTGCTCCTTACCATATACCAATGATGACGTGACATCATCCGAGAAGGGACCTTCCAAGGCCACCACACACCCCCAGTACAAAAAGGATTACGATACGGAATAATCTTATTTATCACTTCTGAAGTATTCTTTTTCCTTGGGTTCTTTTGAGCTTTTTATCACGCCAGCCTAGCACCAACACCCGAATTAGGAGCATACTGACCTCCCTCAGGAATTATCCCACTAGACCCCTTTGAAGTACCACTCCTTAACACAGCCGTACTATTAGCCTCAGGAGTTACAGTAACATGAGCCCACCATAGTATTATAGAAGGAGAACGAAAACAGGCCATCCAATCTTTAGCACTAACTATTCTGCTAGGACTTTATTTTACCGCACTCCAAGCTATTGAGTATTACGAAGCACCTTTCACAATCGCAGACGGAGTCTACGGCTCAACATTCTTCGTAGCCACAGGATTCCACGGGCTACATGTTATTATTGGATCGACTTTCCTAGCAGTATGCCTTCTACGCCAAGCCCAATATCACTTTACGTCTGAACACCACTTTGGTTTTGAGGCCGCTGCCTGATACTGACACTTTGTCGACGTAGTATGACTATTCCTTTACGTATCCATCTACTGATGAGGCTCA

>A._kreyenbergii_1

ATGGCCCATCAAGCACACGCCTACCATATAGTTGACCCAAGCCCATGACCATTAACCGGGGCTATTGCTGCCCTATTAATAACATCAGGCCTAGCAATCTGATTCCACTTTCACTCAACAACACTAATAACTTTAGGAATAACTCTTCTACTCCTTACTATGTACCAATGATGACGTGACATCATCCGAGAAGGGACCTTCCAAGGCCACCACACGCCCCCAGTACAAAAAGGATTACGATACGGAATAATTTTATTCATCACCTCTGAAGTGTTCTTTTTCCTTGGGTTCTTTTGAGCTTTCTATCACGCCAGCCTAGCACCAACACCTGAATTAGGAGCATGCTGACCCCCCTCAGGAATCATCCCACTGGACCCCTTTGAAGTACCGCTCCTCAACACAGCCGTTCTATTAGCCTCAGGGGTTACAGTAACATGGGCCCACCATAGTATTATAGAAGGAGAACGAAAGCAAGCCATCCAATCTTTAGCACTAACCATCCTACTAGGACTTTATTTTACCGCACTTCAAGCCATGGAGTACTACGAAGCACCATTCACAATCGCAGACGGAGTCTACGGCTCAACATTCTTTGTAGCTACAGGATTCCACGGACTACATGTTATTATTGGATCAACTTTCCTAGCGGTATGCCTTCTACGTCAAGCCCAATATCACTTTACGTCCGAACACCACTTTGGTTTCGAGGCCGCTGCCTGATACTGGCACTTTGTCGACGTAGTATGACTATTCCTTTACGTATCCATCTACTGATGAGGCTCA

>A._kreyenbergii_2

ATGGCCCATCAAGCACACGCCTACCATATAGTTGACCCAAGCCCATGACCATTAACCGGGGCTATTGCTGCCCTATTAATAACATCAGGCCTAGCAATCTGATTCCACTTTCACTCAACAACACTAATAACCTTAGGAATAATTCTTCTACTCCTTACTATGTACCAATGATGACGTGACATCATCCGAGAAGGGACCTTCCAAGGCCACCACACGCCCCCAGTACAAAAAGGATTACGGTACGGAATAATCTTATTTATCACCTCTGAAGTGTTCTTTTTCCTTGGGTTCTTTTGAGCTTTTTATCACGCCAGCCTGGCACCAACGCCTGAATTAGGAGCATGCTGACCCCCCTCAGGAATCATCCCACTAGACCCCTTTGAAGTACCACTCCTTAACACAGCCGTTCTATTAGCCTCAGGAGTTACAGTAACATGGGCCCACCATAGTATTATAGAAGGGGAACGAAAACAAGCCATCCAATCTTTAGCACTAACCATCCTACTAGGACTTTATTTTACCGCGCTCCAAGCCATGGAGTACTACGAAGCACCATTCACAATCGCAGACGGAGTCTACGGCTCAACATTCTTTGTAGCTACAGGATTCCACGGACTACATGTTATTATTGGGTCAACTTTCCTAGCGGTATGCCTTCTACGCCAAGCCCAATATCACTTTACGTCCGAACACCACTTTGGTTTCGAGGCCGCTGCCTGATACTGACACTTTGTCGACGTAGTATGACTATTCCTTTACGTATCCATCTACTGATGAGGCTCA

>A._longipinnis

ATGGCCCACCAAGCACATGCCTACCACATAGTTGACCCAAGCCCATGACCACTGACCGGGGCTATTGCTGCCCTACTAATAACATCAGGCCTAGCAATCTGATTCCACTTCCACTCAACAACACTAATAACCTTAGGAATAATTCTTCTACTTCTTACTATATATCAATGATGACGAGACATTATCCGGGAAGGAACCTTCCAAGGACACCACACACCCCCAGTACAAAAAGGACTACGATATGGAATAATCCTATTTATTACCTCTGAAGTATTCTTTTTCCTCGGGTTCTTCTGAGCTTTTTACCACTCAAGTCTGGCACCAACACCTGAACTGGGAGGATGCTGACCTCCCACAGGAATCATCCCACTAGACCCCTTTGAAGTACCACTCCTTAACACAGCTGTACTATTAGCATCAGGGGTCACAGTAACATGAGCCCACCATAGTATTATAGAAGGGGGACGAAAACAAGCTATCCAATCTCTAACATTAACCATCCTACTAGGACTTTATTTTACCGCACTCCAAGCCATAGAGTACTACGAAGCACCCTTCACAATCGCAGACGGAGTCTACGGCTCAACATTCTTTGTGGCTACAGGATTCCACGGACTACACGTTATTATTGGATCAACTTTCCTAGCAGTGTGCCTACTGCGCCAAGCCCAATATCACTTCACATCTGAACACCACTTTGGTTTCGAAGCCGCTGCTTGATACTGACATTTCGTCGACGTAGTATGACTATTCCTCTACGTATCTATCTACTGATGAGGCTCA

>A._monticola_1

ATGGCCCACCAAGCACATGCCTACCACATAGTCGACCCAAGCCCATGACCCCTAACCGGAGCTATCGCTGCCCTACTAACAACATCCGGCTTAGCAATCTGATTTCACTTCCACTCAACAACACTAATAACCTTAGGAATAATCCTCCTACTTCTCACCATATACCAATGATGACGCGACATTATTCGAGAGGGAACCTTCCAAGGCCATCATACGCCCCCCGTGCAAAAAGGCCTACGGTACGGAATAATTCTATTTATCACCTCTGAAGTATTCTTTTTCCTCGGGTTCTTCTGGGCCTTTTATCACTCAAGCCTAGCACCAACACCTGAACTAGGGGGATGCTGACCCCCCACAGGAATCATCCCACTAGACCCCTTTGAAGTGCCCCTCCTCAACACAGCTGTACTACTGGCATCAGGGGTTACAGTAACATGAGCTCACCACAGCATTATAGAAGGAAAACGAAAACAAGCTATTCAATCTCTAGCATTAACAATTCTACTGGGACTTTACTTCACCGCACTTCAAGCCATAGAATACTACGAAGCACCATTTACAATCGCAGATGGAGTCTACGGCTCAACATTCTTCGTAGCCACAGGATTCCACGGACTACATGTCATTATTGGATCAACCTTCCTGGCAGTCTGTCTTCTACGCCAAGCCCAATATCACTTTACATCTGAACATCACTTTGGCTTTGAAGCCGCTGCCTGATACTGACACTTTGTAGACGTAGTATGACTATTCCTCTACGTATCTATCTACTGATGAGGCTCA

>A._monticola_2

ATGGCCCACCAAGCACATGCCTACCACATAGTCGACCCAAGCCCATGACCCCTAACCGGAGCTATCGCTGCCCTACTAACAACATCCGGCTTAGCAATCTGATTTCACTTCCACTCAACAACACTAATAACCTTAGGAATAATCCTCCTACTTCTCACCATATACCAATGATGACGCGACATTATTCGAGAGGGAACCTTCCAAGGCCATCATACGCCCCCCGTGCAAAAAGGCCTACGGTACGGAATAATTCTATTTATCACCTCTGAAGTATTCTTTTTCCTCGGGTTCTTCTGGGCCTTTTATCACTCAAGCCTAGCACCAACACCTGAACTAGGGGGATGCTGACCCCCCACAGGAATCATCCCACTAGACCCCTTTGAAGTGCCCCTCCTCAACACAGCTGTACTACTGGCATCAGGGGTTACAGTAACATGAGCTCACCACAGCATTATAGAAGGAAAACGAAAACAAGCTATTCAATCTCTAGCATTAACAATTCTACTGGGACTTTACTTCACCGCACTTCAAGCCATAGAATACTACGAAGCACCATTTACAATCGCAGATGGAGTCTACGGCTCAACATTCTTCGTAGCCACAGGATTCCACGGACTACATGTCATTATTGGATCAACCTTCCTGGCAGTCTGTCTTCTACGCCAAGCCCAATATCACTTTACATCTGAACATCACTTTGGCTTTGAAGCCGCTGCCTGATACTGACACTTTGTAGACGTAGTATGACTATTCCTCTACGTATCTATCTACTGATGAGGCTCA

>A._paradoxus_1

ATGGCCCATCAAGCACACGCCTACCACATAGTCGACCCAAGCCCATGACCACTAACCGGGGCTATTGCTGCCTTACTAATAACATCAGGCCTGGCAACCTGATTCCACTTCCACTCAACAACACCAATAACTTTAGGCATAATTCTTCTACTTCTTACCATGTACCAATGATGACGTGATATTATCCGGGAAGGAACCTTCCAAGGCCACCACACGCCCCCAGTACAAAAAGGACTACGATATGGAATAATCTTATTTATTACCTCTGAAGTATTCTTTTTCCTCGGGTTCTTTTGAGCTTTTTATCACTCTAGCCTAGCACCGACACCCGAATTAGGAGGGTGCTGGCCCCCCGCAGGAATCGTCCCACTAGACCCCTTTGAAGTACCACTCCTTAACACAGCCGTTCTACTAGCCTCGGGGGTCACAGTAACATGAGCCCACCATAGTATCATGGAAGGACAACGAGAACAAGCCCTTCAATCTTTAGTACTAACTATCCTACTAGGACTTTATTTTACCACACTCCAAGCCATCGAATACTATGAAGCACCATTCACAATTGCAGACGGAGTCTACGGCTCAACATTTTTCGTGGCCACAGGGTTCCACGGACTACATGTTATTATTGGGTCAACTTTCCTAGCAGTATGCCTTCTGCGCCAAGCCCTATACCACTTTACGTCCGAACACCACTTTGGCTTTGAGGCCGCTGCCTGATACTGACACTTCGTCGACGTAGTATGACTATTCCTCTACGTATCCATCTACTGATGAGGCTCA

>A._paradoxus_2

ATGGCCCATCAAGCACACGCCTACCATATAGTTGACCCAAGCCCATGACCACTAACCGGAGCTATTGCTGCCCTATTAATAACATCAGGCCTAGCAATCTGGTTCCATTTTCACTCAACAACACTAATGACTCTAGGAATAATTCTTCTGCTCCTTACCATATACCAATGATGACGTGACATCATCCGAGAAGGGACCTTCCAAGGCCACCACACACCCCCAGTACAAAAAGGATTACGATACGGAATAATCTTATTTATCACTTCTGAAGTATTCTTTTTCCTTGGGTTCTTTTGAGCTTTTTATCACGCCAGCCTAGCACCAACACCCGAATTAGGAGCATACTGACCTCCCTCAGGAATTATTCCACTAGACCCCTTTGAAGTACCACTCCTTAACACAGCCGTACTATTAGCCTCAGGAGTTACAGTAACATGAGCCCACCATAGTATCATAGAAGGAGAACGAAAACAGGCCATCCAATCTTTAGCACTAACTATTCTGCTAGGACTTTATTTTACCGCACTCCAAGCTATTGAGTATTACGAAGCACCTTTCACAATCGCAGACGGAGTCTACGGCTCAACATTCTTCGTAGCCACAGGATTCCACGGGCTACATGTTATTATTGGATCGACTTTCCTAGCAGTATGCCTTCTACGCCAAGCCCAATATCACTTTACGTCTGAACACCACTTTGGTTTTGAGGCCGCTGCCTGATACTGACACTTTGTCGACGTAGTATGACTATTCCTTTACGTATCCATCTACTGATGAGGCTCA

>A._parallens_1

ATGGCCCATCAAGCACACGCCTACCATATAGTTGACCCAAGCCCATGACCACTAACCGGGGCTATTGCTGCCCTATTAATAACATCAGGCCTAGCAATCTGATTCCATTTTCACTCAACAACATTAATATCTTTAGGAATAATTCTTCTACTCCTCACCATATACCAATGATGACGTGACATCATCCGAGAAGGGACCTTCCAAGGCCACCACACACCCCCAGTACAAAAAGGATTACGATACGGAATAATCTTATTTATCACCTCTGAAGTATTCTTTTTCCTCGGGTTCTTTTGAGCCTTTTATCACGCCAGCCTGGCACCAACACCCGAACTAGGAGCATGCTGACCCCCCTCAGGAATCATCCCACTAGACCCCTTTGAAGTGCCACTCCTTAACACAGCCGTACTATTAGCCTCCGGGGTCACAGTAACATGAGCCCACCACAGTATTATAGAAGGAGAACGAAAACAAGCCATCCAATCTTTAGCACTAACCATTCTACTAGGACTCTATTTTACCGCACTCCAAGCCATGGAGTATTACGAAGCACCATTCACAATCGCAGACGGAGTCTACGGCTCAACATTCTTCGTGGCTACAGGATTCCACGGACTACATGTTATTATTGGGTCGACCTTCCTAGCAGTCTGCCTCCTACGCCAAGCCCAATATCACTTTACGTCCGAACACCACTTTGGTTTTGAGGCCGCTGCCTGATACTGACACTTTGTTGACGTAGTATGACTATTCCTTTACGTATCCATCTACTGATGAGGCTCA

>A._parallens_2

ATGGCCCATCAAGCACACGCCTACCATATAGTTGACCCAAGCCCATGACCACTAACCGGGGCTATTGCTGCCCTATTAATAACATCAGGCCTAGCAATCTGATTCCATTTTCACTCAACAACACTAATATCTTTAGGAATAATTCTTCTACTCCTCACCATATACCAATGATGACGTGATATCATCCGAGAAGGGACCTTCCAAGGCCACCACACACCCCCAGTACAAAAAGGGTTACGATACGGAATAATCTTATTTATCACCTCTGAAGTATTCTTTTTCCTCGGGTTCTTTTGAGCCTTTTATCACGCCAGCCTAGCACCAACACCCGAACTGGGAGCATGCTGACCCCCCTCAGGAATCATCCCACTAGACCCCTTTGAAGTACCACTCCTTAACACAGCCGTACTATTAGCCTCCGGGGTCACAGTAACATGAGCCCACCACAGTATTATAGAAGGAGAACGAAAACAAGCCATCCAATCTTTGGCACTAACCATTCTACTAGGACTCTATTTTACCGCACTCCAAGCCATGGAATATTACGAAGCACCATTCACAATCGCAGACGGAGTCTACGGCTCAACATTCTTCGTAGCTACAGGATTCCACGGACTACATGTTATTATTGGATCGACCTTCCTAGCAGTCTGCCTTTTACGCCAAGCCCAATATCACTTTACGTCCGAACACCACTTTGGTTTTGAGGCCGCTGCCTGATACTGACACTTTGTTGACGTAGTATGACTATTCCTTTACGTATCCATCTACTGATGAGGCTCA

>A._parallens_3

ATGGCCCATCAAGCACACGCCTACCATATAGTTGACCCAAGCCCATGACCACTAACCGGAGCTATTGCTGCCCTATTAATAACATCAGGCCTAGCAATCTGATTCCATTTTCACTCAACAACACTAATATCTTTAGGAATAATTCTTCTACTCCTCACCATATACCAATGATGACGTGACATCATCCGAGAAGGGACCTTCCAAGGCCACCACACACCCCCAGTACAAAAAGGATTACGATACGGAATAATCTTATTTATCACCTCTGAAGTATTCTTTTTCCTCGGGTTCTTTTGAGCCTTTTATCACGCCAGCCTGGCACCAACACCCGAACTAGGAGCATGCTGACCCCCCTCAGGAATCATCCCACTAGACCCCTTTGAAGTGCCACTCCTTAACACAGCCGTACTATTAGCCTCCGGGGTCACAGTAACATGAGCCCACCACAGTATTATAGAAGGAGAACGAAAACAAGCCATCCAATCTTTAGCACTAACCATTCTACTAGGACTCTATTTTACCGCACTCCAAGCCATGGAATATTACGAAGCACCATTCACAATCGCAGACGGAGTCTACGGCTCAACATTCTTCGTGGCTACAGGATTCCACGGACTACATGTTATTATTGGATCGTCCTTCCTAGCAGTCTGCCTCTTACGCCAAGCCCAATATCACTTTACGTCCGAACACCACTTTGGTTTTGAGGCCGCTGCCTGATACTGACACTTTGTTGACGTAGTATGACTATTCCTTTACGTATCCATCTACTGATGAGGCTCA

>A._parallens_4

ATGGCCCATCAAGCACACGCCTACCATATAGTTGACCCAAGCCCATGACCACTAACCGGAGCTATTGCTGCCCTATTAATAACATCAGGTCTAGCAATCTGATTCCATTTTCACTCAACAACACTAATATCTTTAGGAATAATTCTTCTACTCCTCACCATATACCAATGATGACGTGACATCATCCGAGAAGGGACCTTCCAGGGCCACCACACACCCCCAGTACAAAAAGGATTACGATACGGAATAATCTTATTTATCACCTCTGAAGTATTCTTTTTCCTCGGGTTCTTTTGAGCCTTTTATCACGCCAGCCTGGCACCAACACCCGAACTAGGAGCATGCTGACCCCCCTCAGGAATCATCCCACTAGACCCCTTTGAAGTGCCACTCCTTAACACAGCCGTACTATTAGCCTCCGGGGTCACAGTAACATGAGCCCACCACAGTATTATAGAAGGAGAACGAAAACAAGCCATCCAATCTTTAGCACTAACCATTCTACTAGGACTCTATTTTACCGCACTCCAAGCCATGGAATATTACGAAGCACCATTCACAATCGCAGACGGAGTCTACGGCTCAACATTCTTCGTGGCTACAGGATTCCACGGACTTCATGTTATTATTGGATCGACCTTCCTAGCAGTCTGCCTTTTACGCCAAGCCCAATATCACTTTACGTCCGAACACCACTTTGGTTTTGAAGCCGCTGCCTGATACTGACACTTTGTTGACGTAGTATGACTATTCCTTTACGTATCCATCTACTGATGAGGCTCA

>A._spinifer

ATGGCCCATCAAGCACACGCCTACCACATAGTTGACCCAAGCCCATGACCACTAACCGGGGCTATTGCTGCCTTACTAATAACATCAGGCCTGGCAACCTGATTCCACTTCCACTCAACAACACCAATAACTTTAGGTATAATTCTTCTACTTCTTACCATATACCAGTGATGACGTGACATCATCCGGGAGGGAACCTTCCAAGGCCACCACACACCCCCAGTACAAAAAGGACTACGATATGGGATAATCTTATTTATTACCTCTGAAGTATTCTTTTTCCTTGGGTTCTTTTGAGCTTTTTATCACTCCAGCCTGGCACCAACACCCGAACTAGGAGGATGCTGACCCCCCACAGGAATCCTCCCACTAGACCCCTTCGAAGTGCCACTTCTTAACACAGCCGTACTATTAGCCTCAGGGGTTACAGTAACATGAGCCCACCATAGTATCATGGAGGGAGAACGAGAACAAGCCCTCCAATCTTTAACACTAACCATCCTACTGGGACTTTATTTTACCGCACTCCAAGCCATCGAATACTACGAAGCACCATTCACAATTGCAGACGGAGTCTACGGCTCAACATTTTTCGTAGCCACAGGGTTCCACGGACTACATGTTATTATCGGATCAACTTTCCTAGCAGTATGCCTTCTACGCCAAGCCCTGTACCACTTTACATCCGAACACCATTTTGGCTTCGAGGCCGCTGCCTGATACTGACACTTCGTCGACGTAGTATGACTATTCCTCTACGTATCCATCTACTGATGAGGCTCA

>A._stenotaeniatus

ATGGCCCATCAAGCACACGCCTACCACATAGTTGACCCAAGCCCATGACCACTAACCGGGGCTATTGCTGCCTTACTAATAACATCAGGCCTGGCAACCTGATTCCACTTCCACTCAACAACACCAATAACTTTAGGTATAATTCTTCTACTTCTTACCATATACCAATGATGACGTGACATCATCCGGGAGGGAACCTTCCAAGGCCACCACACACCCCCAGTACAAAAAGGACTACGATATGGAATAATCTTATTTATTACCTCTGAGGTATTCTTTTTTCTTGGGTTCTTTTGAGCTTTTTATCACTCCAGCCTGGCACCAACACCCGAACTAGGAGGATGCTGACCCCCCACAGGAATCCTCCCACTAGACCCCTTCGAAGTACCACTTCTTAACACAGCCGTACTATTAGCCTCAGGGGTTACGGTAACATGAGCCCACCATAGTATCATGGAGGGAGAACGAGAACAAGCCCTCCAATCTTTAACACTAACCATCCTACTGGGACTTTATTTTACCGCACTCCAAGCCATTGAGTACTACGAAGCACCATTCACAATTGCAGACGGAGTCTACGGCTCAACATTTTTCGTAGCCACAGGGTTCCACGGACTACATGTTATTATCGGATCAACTTTCCTAGCAGTATGCCTTCTACGCCAAGCCCTCTACCACTTTACATCCGAACACCATTTTGGCTTCGAGGCCGCTGCCTGATACTGACACTTCGTCGACGTAGTATGACTATTCCTCTACGTATCCATCTACTGATGAGGCTCA

>A._wenchowensis_1

ATGGCCCATCAAGCACACGCCTACCACATAGTTGACCCAAGCCCATGACCATTAACCGGGGCTATTGCTGCCCTACTAATAACATCAGGCCTAGCAATCTGATTCCATTTTCACTCAACAACACTAATAACTTTAGGGATAATTCTTCTACTCCTCACCATATACCAATGATGACGTGACATCATCCGAGAAGGAACCTTCCAAGGCCATCACACACCCCCAGTACAAAAAGGATTACGATATGGAATAATTTTATTTATCACCTCTGAAGTATTCTTTTTCCTTGGGTTCTTTTGAGCTTTTTATCACGCCAGCCTGGCACCAACACCTGAATTAGGAGCATGCTGACCCCCCTCAGGAATCGTTCCACTAGACCCCTTTGAAGTACCACTCCTTAACACAGCCGTTCTATTAGCCTCAGGGGTTACAGTTACATGAGCCCACCATAGTATTATAGAAGGAGAACGAAAGCAAGCCATCCAATCTTTAGCACTAACCATTCTACTAGGACTTTATTTTACCGCACTCCAAGCCATGGAGTATTACGAAGCACCATTCACAATCGCAGACGGAGTCTACGGCTCAACATTTTTCGTGGCTACAGGATTCCACGGACTACATGTTATTATCGGATCAACTTTCCTAGCGGTATGTCTTCTACGCCAAGCCCAATATCACTTTACGTCCGAACACCACTTTGGTTTCGAAGCCGCTGCCTGATACTGACACTTTGTCGACGTAGTATGACTATTCCTTTACGTATCCATCTACTGATGAGGCTCA

>A._wenchowensis_2

ATGGCCCATCAAGCACACGCCTACCATATAGTTGACCCAAGCCCATGACCATTAACCGGAGCTATTGCTGCCCTATTAATAACATCAGGCCTAGCAATCTGATTCCATTTTCACTCAACAACACTAATAACTTTAGGAATAATTCTTCTACTCCTTACCATGTACCAATGATGACGTGACATCATCCGAGAAGGGACCTTCCAAGGCCACCACACGCCCCCAGTACAAAAAGGATTACGATATGGAATAATTTTATTTATCACCTCTGAAGTATTCTTTTTCCTTGGATTCTTTTGAGCTTTTTATCACGCCAGCCTGGCACCAACACCTGAATTAGGAGCATGCTGACCCCCCTCAGGAATCATCCCACTAGACCCCTTTGAAGTGCCACTCCTCAACACAGCCGTTCTATTAGCCTCGGGGGTTACAGTAACATGAGCCCACCATAGTATCATGGAAGGAGAACGAAAGCAAGCCATTCAATCTTTAGCACTAACCATCCTTCTAGGACTTTATTTTACCGCACTCCAAGCCATGGAGTACTACGAAGCACCATTCACAATCGCAGACGGAGTCTACGGCTCAACATTTTTCGTAGCTACAGGGTTCCACGGACTACATGTTATTATTGGGTCAACTTTCCTAGCGGTATGCCTTCTACGCCAAGCCCAATATCACTTTACGTCCGAACACCACTTTGGTTTTGAAGCCGCTGCCTGATACTGACACTTTGTCGACGTAGTATGACTATTCCTTTACGTATCCATCTACTGATGAGGCTCA

>A._wuyiensis

ATGGCCCATCAAGCACACGCCTACCACATAGTCGACCCAAGCCCATGACCACTAACCGGGGCTATTGCTGCCCTACTAATAACATCAGGCCTGGCAACCTGATTCCACTTCCACTCAACAACACCAATAACTTTAGGCATAATTCTTCTACTTCTTACCATGTACCAATGATGACGTGATATTATTCGGGAAGGAACCTTCCAAGGCCACCACACGCCCCCAGTACAAAAAGGACTACGATATGGAATAATCTTATTTATTACCTCTGAAGTATTCTTTTTCCTCGGGTTCTTTTGAGCTTTTTATCACTCTAGCCTAGCACCAACACCCGAATTAGGAGGGTGCTGACCCCCCGCAGGAATCGTCCCACTAGACCCCTTTGAAGTACCACTCCTTAACACAGCCGTTCTACTAGCCTCAGGGGTCACAGTAACATGGGCCCACCATAGTATCATGGAAGGACAACGAGAACAAGCCCTTCAATCTTTAGTACTAACTATCCTACTAGGACTTTATTTTACTACACTCCAAGCCATCGAATACTATGAAGCCCCATTCACAATTGCAGACGGAGTCTACGGCTCAACATTTTTCGTGGCCACAGGATTCCACGGACTACATGTTATTATTGGGTCAACTTTCCTAGCAGTATGCCTTCTGCGCCAAGCCCTATACCACTTTACGTCCGAACACCACTTTGGCTTTGAGGCCGCTGCCTGATACTGACACTTCGTCGACGTAGTATGACTATTCCTCTACGTATCCATCTACTGATGAGGCTCA

>A._yunnanensis_1

ATGGCCCACCAAGCACATGCCTACCACATAGTTGACCCAAGCCCATGACCACTGACCGGAGCTATTGCTGCCCTACTAATAACATCAGGCTTAGCAATCTGATTTCACTTCCACTCAACAACATTAATAACTTTAGGAATAATTCTCCTACTTCTCACCATATACCAATGATGACGTGATATTATTCGAGAAGGGACCTTTCAAGGCCATCACACTCCCCCGGTACAAAAAGGCCTGCGATACGGAATAATTCTATTTATCACCTCTGAAGTATTCTTTTTCCTCGGGTTCTTCTGAGCCTTTTACCACTCAAGCCTAGCACCAACACCTGAGCTAGGAGGATGCTGACCCCCCACAGGAATCGTGCCACTAGACCCCTTCGAAGTGCCCCTCCTCAACACAGCCGTACTATTAGCATCAGGGGTTACAGTAACATGAGCCCATCACAGCATCATGGAAGGAAAACGAAAACAAGCTATCCAATCTCTAGCACTAACAATCCTTCTAGGACTTTACTTCACCGCACTTCAAGCCATAGAATACTACGAAGCGCCATTTACAATCGCAGACGGAGTCTATGGCTCAACATTCTTCGTAGCTACAGGATTCCACGGACTACATGTCATTATTGGATCAACCTTCCTGGCAGTATGTCTTCTGCGCCAAGCCCAATACCACTTCACATCTGAACACCACTTTGGTTTTGAAGCCGCTGCCTGATACTGACACTTTGTCGACGTAGTATGGCTATTCCTCTACGTGTCCATCTACTGATGAGGCTCA

>A._yunnanensis_2

ATGGCCCACCAAGCACATGCCTATCACATAGTTGACCCAAGCCCATGACCACTGACCGGAGCTATTGCTGCCCTACTAATAACATCAGGCTTAGCAATCTGATTTCACTTCCACTCAACAACATTAATAACTTTAGGAATAATTCTCCTACTTCTCACCATATACCAATGATGACGTGATATTATTCGAGAAGGGACCTTTCAAGGCCATCACACTCCCCCAGTACAAAAAGGCCTGCGATACGGAATAATTCTATTTATTACCTCTGAAGTATTCTTTTTCCTCGGGTTCTTCTGAGCCTTTTACCACTCAAGCCTAGCACCAACACCTGAGCTAGGAGGATGCTGACCCCCCACAGGAATCGTGCCACTAGACCCCTTCGAAGTACCCCTCCTCAACACAGCCGTACTATTAGCATCAGGAGTTACAGTAACATGAGCCCATCACAGCATCATGGAGGGAAAACGAAAACAAGCTATCCAATCTCTAGCACTAACAATCCTTCTAGGACTTTACTTCACCGCACTTCAAGCCATAGAATACTACGAAGCGCCATTTACAATCGCAGACGGAGTCTATGGCTCAACATTCTTCGTAGCTACAGGATTCCACGGACTACATGTCATTATTGGATCAACCTTCCTGGCAGTATGCCTTCTGCGCCAAGCCCAGTACCACTTCACATCTGAACACCACTTTGGTTTTGAAGCCGCTGCCTGATACTGACACTTTGTCGACGTAGTATGACTATTCCTCTACGTGTCCATCTACTGATGAGGCTCA

>Onychostoma_barbatulum

ATGGCCCACCAAGCACATGCCTACCACATAGTTGACCCAAGCCCATGACCACTGACAGGAGCTATTGCTGCCCTATTAACAACATCAGGATTAGCAATCTGATTTCACTTCCACTCAATAACACTAATAACTTTAGGAATAATTCTTCTACTTCTTACTATATACCAATGATGACGAGACATCATCCGAGAAGGGACCTTTCAAGGCCACCACACACCCCCAGTACAAAAAGGGTTACGGTACGGAATAATTCTATTTATCACCTCTGAAGTATTCTTTTTTCTAGGATTCTTCTGAGCCTTTTACCACTCAAGCCTAGCACCAACACCTGAATTAGGCGGGTGCTGACCTCCTACAGGAATTATTCCACTAGACCCCTTTGAAGTACCACTCCTTAATACAGCCGTACTATTAGCATCAGGGGTCACAGTAACATGAGCCCACCACAGCATTATGGAAGGAAAGCGAAAACAAGCCATCCAATCTTTAATGTTAACTATTCTACTAGGGCTTTATTTCACCGCACTCCAAGCCATAGAATACTACGAAGCACCATTTACAATCGCAGATGGAGTCTACGGCTCAACATTCTTCGTAGCCACAGGGTTCCATGGACTACATGTTATTATTGGGTCAACCTTCCTGGCAGTATGCCTTCTCCGCCAAACCCAGTACCACTTTACATCTGAACACCACTTTGGTTTTGAAGCCGCTGCTTGATATTGACACTTTGTCGACGTAGTCTGATTATTCCTCTACGTATCTATTTACTGATGAGGCTCA

>Onychostoma_meridionale

ATGGCCCATCAAGCACACGCCTATCACATAGTCGATCCAAGCCCATGACCACTGACCGGAGCTATTGCTGCCTTACTGATAACATCAGGCCTAGCAATCTGATTTCACTTCCACTCAACCACACTAATAACTTTAGGAATAATTCTCCTACTTCTTACCATATATCAATGATGACGAGACATCATCCGAGAAGGGACCTTCCAAGGCCACCACACACCCCCAGTTCAAAAAGGACTACGATATGGAATAATCCTATTTATCACCTCCGAAGTATTCTTCTTCCTTGGGTTCTTCTGAGCTTTTTATCACTCAAGCCTGGCACCCACACCTGAGCTAGGAGGATGTTGACCCCCCACAGGAATCGTCCCACTAGACCCCTTTGAAGTACCACTCCTTAACACAGCCGTTTTATTGGCCTCGGGTGTTACAGTAACATGGGCCCACCACAGCATCATAGAGGGAAAACGAAAACAAGCCATCCAATCTCTAGCCCTAACCATCCTACTAGGACTTTATTTCACCGCACTCCAAGCCATTGAGTACTATGAAGCACCATTCACGATTGCAGACGGAGTCTACGGCTCAACATTCTTCGTTGCCACAGGATTCCATGGACTACACGTTATTATTGGATCAACCTTCCTGGCAGTCTGTCTTCTACGCCAAGCCCAATACCACTTTACGTCCGAACACCACTTTGGCTTTGAAGCCGCTGCCTGATACTGACACTTTGTTGACGTAGTATGACTATTCCTCTACGTATCAATCTACTGATGAGGCTCA

>Onychostoma_gerlachi

ATGGCCCATCAAGCACACGCCTACCACATAGTTGATCCAAGCCCATGACCACTGACCGGAGCTATTGCCGCCTTACTGATAACATCAGGCCTAGCAATCTGATTTCACTTCCACTCAACCACACTAATATCTTTAGGAATAATTCTCCTACTTCTTACCATGTATCAATGATGACGAGACATCATTCGAGAAGGGACCTTCCAAGGACACCATACACCCCCAGTACAAAAAGGGCTACGATACGGAATAATCCTGTTCATCACCTCCGAAGTATTCTTCTTCCTTGGGTTCTTCTGAGCCTTTTATCACGCAAGCCTGGCACCCACACCCGAATTAGGAGGATGCTGACCCCCCACAGGAATCATCCCACTAGACCCCTTTGAAGTACCACTCCTCAACACAGCCGTACTACTGGCCTCAGGTGTTACAGTGACATGAGCCCACCACAGCATCATAGAAGGAAAACGAAAACAAACCATCCAATCTCTAGCCCTAACCATCCTACTAGGACTTTACTTCACTGCACTCCAAGCCATAGAATACTACGAAGCACCATTCACAATTGCAGACGGAGTCTACGGCTCAACATTCTTCGTGGCCACAGGATTCCACGGACTACACGTTATCATCGGATCAACCTTCCTAGCAGTATGTCTTCTCCGCCAAGCCCAATACCACTTTACGTCCGAACACCACTTTGGTTTCGAAGCCGCTGCCTGATACTGACACTTCGTTGACGTAGTATGACTATTCCTCTACGTATCAATCTATTGATGAGGCTCA

>Spinibarbus_denticulatus

ATGGCCCACCAAGCACATGCACTTCATATAGTAGATCCAAGCCCATGACCACTAACCGGAGCTATCGCTGCCCTATTAATAACATCCGGCTTAGCAATCTGATTCCACTTCCACTCAACAACACTTATAACTTTAGGAATAATTCTCCTACTTCTTACCATGTACCAATGATGACGTGATATTATCCGAGAGGGGACCTTTCAAGGCCACCACACACCCCCAGTACAAAAAGGATTACGCTACGGAATAATTCTATTTATTACCTCCGAAGTATTCTTTTTCCTCGGATTCTTCTGAGCTTTCTACCACTCAAGTTTAGCACCGACACCAGAGCTAGGAGGATGCTGACCCCCTACAGGAATCACCCCACTAGACCCCTTCGAAGTGCCACTCCTTAATACAGCCGTATTACTAGCATCAGGGGTTACAGTAACATGAGCTCACCACAGTATTATAGAAGGCGAACGAAAACAAGCTATTCAATCTTTAACTTTAACCATTTTACTAGGACTTTATTTTACCGCACTTCAAGCCATAGAATACTACGAAGCGCCATTTACAATCGCAGACGGAGTCTACGGCTCAACATTCTTCGTAGCCACAGGATTTCATGGACTACACGTTATTATTGGATCAACTTTCTTAGCAGTATGTCTTCTACGCCAAATCCAATACCACTTTACATCCGAACACCACTTTGGCTTCGAAGCCGCTGCCTGATACTGACACTTTGTTGACGTAGTATGACTATTCCTCTACGTGTCTATCTATTGATGAGGCTCA

>Spinibarbus_hollandi

ATGGCCCACCAAGCACATGCCTATCACATAGTAGATCCAAGCCCATGACCACTAACCGGAGCTATCGCTGCCCTACTAATAACATCCGGCCTAGCAATCTGATTTCACTTCCACTCAACAACACTTATAACCTTAGGAATAATTCTCCTACTTCTTACCATATATCAATGATGACGTGATATTATCCGAGAAGGAACCTTTCAAGGCCACCACACACCCCCAGTACAAAAAGGATTACGATATGGAATAATCCTGTTTATTACTTCCGAAGTATTCTTTTTCCTCGGATTCTTCTGAGCCTTTTATCACTCAAGTTTAGCGCCAACACCAGAGCTAGGAGGATGCTGACCCCCCACAGGAATCACCCCACTAGACCCCTTCGAAGTGCCACTCCTTAACACAGCTGTATTACTAGCATCAGGGGTTACAGTAACATGAGCTCACCACAGTATCATAGAAGGCGAACGAAAACAAGCTATTCAATCCTTAATTTTAACCATTTTACTAGGACTTTATTTTACCGCGCTTCAAGCCATAGAATACTACGAAGCGCCATTTACAATCGCAGACGGGGTGTACGGCTCAACATTCTTTGTAGCCACAGGGTTCCATGGACTACATGTTATTATTGGATCAACTTTCTTAGCAGTATGTCTTCTACGCCAAATCCAATACCATTTCACATCCGAACACCACTTTGGTTTTGAAGCCGCTGCCTGATACTGACACTTTGTTGACGTAGTATGACTATTCCTTTACGTATCTATCTATTGATGAGGCTCA

>Spinibarbus_sinensis

ATGGCCCACCAAGCACATGCCTATCATATAGTTGATCCAAGCCCATGACCACTAACCGGAGCTATCGCTGCCCTACTAATAACATCCGGCTTAGCAATCTGATTCCACTTCCACTCAACAACACTAATAACTTTAGGATTAATTCTCCTACTTCTTACTATATATCAATGATGACGTGACATTATCCGAGAGGGGACCTTCCAAGGTCACCACACGCCTCCAGTCCAAAAAGGACTGCGATACGGAATAATCCTGTTCATTACCTCCGAGGTATTCTTCTTCCTTGGGTTCTTCTGAGCCTTTTACCACTCAAGCCTGGCACCCACACCAGAACTAGGAGGATGCTGACCCCCTACAGGAATCACTCCGCTAGACCCCTTTGAAGTACCACTCCTCAACACAGCCGTACTACTAGCGTCAGGGGTTACAGTAACATGAGCTCATCACAGCATTATGGAAGGAGAACGAAAACAAGCTATTCAATCTCTAGCATTAACCATTTTACTAGGACTTTATTTCACCGCACTCCAAGCCATAGAATATTACGAAGCACCATTCACAATCGCAGACGGAGTCTACGGCTCAACATTCTTTGTAGCCACAGGGTTCCATGGACTACACGTTATTATTGGATCAACTTTCCTAGCAGTATGTCTTCTACGCCAAATCCAATACCACTTTACCTCCGAACACCACTTTGGTTTTGAAGCCGCTGCCTGATACTGACACTTTGTTGACGTAGTATGACTATTCCTCTACGTATCCATCTATTGATGAGGCTCA

**CYTB**

>A._barbodon

ATGGCAAGCCTACGAAAAACACACCCCTTAATTAAAATTGCTAACGACGCACTAGTTGACCTACCAGCACCATCCAACATTTCAGCATGATGAAACTTCGGATCCCTCCTAGGACTATGTTTAGCTACTCAAATTTTAACTGGCCTATTCCTGGCCATACACTACACCTCAGATATCTCAACCGCATTTTCATCAGTAACCCATATTTGCCGGGACGTAAATTACGGCTGACTAATCCGTAACATCCACGCCAACGGAGCATCATTCTTTTTCATCTGCATCTACATACACATCGCCCGAGGCCTCTATTACGGATCCTACCTCTACAAAGAAACTTGAAACATTGGCGTAATCCTCCTCCTGCTAGTTATAATAACAGCTTTCGTCGGCTATGTCCTCCCATGGGGCCAAATATCCTTCTGAGGCGCCACAGTTATTACAAACCTCCTATCTGCTGTACCGTATATAGGAGATATACTAGTCCAATGAATCTGAGGCGGATTCTCAGTTGATAACGCAACACTAACACGATTCTTCGCATTCCACTTCCTACTACCATTTATTATTGCCGCCGTAACCATTCTTCACCTTCTATTCCTCCACGAAACAGGATCAAACAACCCAATCGGGCTAAATTCAGACGCAGACAAAATCTCCTTCCACCCATACTTTTCATACAAAGACTTACTCGGGTTTGTACTAATACTTCTGGCCCTTATACTACTAGCTCTATTCTCCCCAAACTTACTAGGAGACCCAGAAAACTTTACCCCCGCTAACCCCTTAGTTACACCTCCACACATTAAACCAGAATGATACTTCCTATTCGCTTACGCCATCCTACGATCAATCCCAAACAAACTCGGAGGTGTCCTTGCATTACTATTCTCTATCTTAGTACTAATAGTAGTACCACTTTTACATACCTCCAAGCAACGGGGACTAACATTCCGCCCCCTCACTCAATTCCTATTCTGAACCCTAGTGGCAGATATAATTATCTTAACATGAATTGGGGGTATACCAGTAGAACACCCATTTATTATCATTGGACAAATCGCATCCGTCTTATACTTCGCACTGTTCCTAGTCTTTATCCCACTAGCAGGATGACTAGAAAACAAAGCACTAGAATGAGCT

>A._beijiangensis_1

ATGGCAAGCCTACGAAAAACACACCCTCTAATTAAAATTGCTAACGACGCACTAGTCGACCTACCAGCACCATCTAACATCTCAGTATGATGAAATTTTGGCTCCCTCCTAGGACTATGCTTAATTACTCAAATCCTGACTGGCCTATTCCTAGCCATACACTACACCTCAGACATTTCAACCGCATTCTCGTCAGTAGCACACATCTGCCGAGACGTAAATTACGGGTGACTAATCCGCAACATCCACGCCAACGGTGCATCATTCTTCTTCATCTGCATCTACCTCCATATTGCCCGAGGCCTCTATTACGGGTCATACCTCTATAAAGAAACCTGAAACATCGGCGTAGTCCTCCTCCTGCTAGTTATAATAACAGCCTTTGTCGGCTATGTCCTCCCATGAGGCCAAATATCCTTCTGAGGTGCTACAGTTATCACTAACCTCCTATCTGCCGTACCATATGTAGGGGACATTCTAGTTCAATGAATCTGAGGCGGATTCTCAGTAGACAACGCAACACTAACACGATTCTTCGCATTCCATTTCCTACTGCCATTCATCATTGCTGCTGTAACTATCCTCCACCTCCTCTTCCTTCATGAAACAGGATCAAACAATCCAATTGGACTAAACTCGGACGCGGACAAAATCCCCTTCCACCCATACTTTACATATAAAGACCTACTTGGATTTGTACTTATGCTCCTAGCCCTAGCACTACTAGCGTTATTCTCCCCCAACCTTCTAGGAGACCCAGATAACTTCACCCCCGCCAACCCCCTAATTACTCCCCCACATATTAAACCAGAGTGATATTTCCTATTTGCCTACGCCATCCTACGATCAATCCCAAATAAACTAGGAGGAGTTCTTGCATTACTATTTTCTATTCTAGTACTTATAGTAGTGCCACTTCTACACACCTCGAAACAACGAGGACTTATATTCCGCCCCCTCACCCAATTCCTATTCTGAACCCTGGCAGCAGACGTAATTATCCTTACATGAATTGGAGGCATACCAGTAGAACACCCATTTATTATTATTGGACAAATCGCATCTATCCTATACTTCGCACTATTCCTAATCTTATTCCCACTAGCAGGATGGTTAGAAAACAAAGCACTAGAATGAGCT

>A._beijiangensis_2

ATGGCAAGCCTACGAAAAACGCACCCTCTAATTAAAATTGCTAACGACGCACTAGTCGACCTACCAGCACCATCTAACATCTCAGTATGATGAAATTTTGGTTCCCTCCTAGGACTATGCTTAATTGCTCAAATCCTGACCGGCCTATTCCTAGCCATACACTACACCTCAGACATTTCAACCGCATTCTCGTCGGTGGCACACATCTGTCGAGACGTAAATTACGGGTGACTAATCCGCAACATCCACGCCAATGGTGCATCGTTCTTCTTCATCTGCATCTACCTCCACATTGCCCGAGGCCTCTATTACGGATCGTACCTCTATAAAGAAACCTGAAACATCGGCGTAGTCCTCCTCCTACTAGTTATGATAACAGCCTTTGTCGGCTATGTCCTCCCATGAGGCCAAATATCCTTCTGAGGTGCTACAGTTATCACTAATCTCCTATCTGCCGTACCATATGTAGGGGACATTCTAGTTCAATGAATCTGAGGCGGATTCTCAGTAGACAACGCAACACTAACACGATTCTTCGCATTCCATTTCCTACTCCCATTCATCATTGCTGCTGTAACTATCCTCCACCTCCTCTTCCTTCATGAAACAGGATCAAACAATCCAATTGGACTAAACTCGGACGCAGACAAAATCCCCTTCCACCCATACTTTACATACAAAGACCTACTTGGATTCGTACTAATACTCCTAGCCCTAGCACTACTAGCGTTATTCTCCCCCAACCTTCTAGGAGACCCAGAAAACTTCACCCCCGCCAACCCCCTAGTTACTCCCCCACACATCAAACCAGAGTGATATTTCCTATTTGCCTACGCCATCCTGCGATCAATCCCAAATAAACTAGGAGGCGTTCTTGCATTACTATTCTCTATTCTAGTACTTATAGTAGTGCCACTTCTACACACCTCGAAACAACGAGGACTTGCATTCCGTCCCCTCACCCAGTTTCTATTCTGAACCCTGGCAGCAGACGTAATTATCCTTACATGAATTGGGGGCATACCAGTAGAACACCCATTTATCATTATTGGACAAATCGCATCTATCCTATACTTCGCACTATTCCTAATCTTATTCCCACTAGCAGGATGGTTAGAGAACAAAGCACTAGAATGAGCT

>A._fasciatus

ATGGCAAGCCTACGAAAAACGCACCCATTAATTAAAATTGCTAACGACGCGCTAGTCGACCTACCAGCGCCATCTAATATCTCAGTGTGATGGAACTTTGGCTCCCTCCTAGGGCTATGTTTGATTACCCAAATCCTAACCGGCCTATTCCTAGCCATACACTACACCTCAGACATTTCAACCGCATTTTCATCAGTAGCCCACATTTGTCGAGATGTAAACTACGGATGACTAATCCGCAATATCCACGCCAATGGAGCATCATTCTTCTTCATCTGCATCTACATACACATTGCCCGAGGCCTCTATTACGGGTCATACCTCTACAAGGAAACCTGAAACATCGGCGTGGTCCTTCTTCTGCTAGTCATGATGACAGCCTTTGTAGGCTACGTACTCCCATGGGGCCAAATATCCTTCTGAGGTGCCACAGTTATCACAAATCTCCTATCCGCCGTACCATATATAGGAGACATGTTGGCTCAATGGATCTGAGGTGGCTTCTCAGTAGACAACGCAACATTAACACGATTCTTCGCATTCCACTTCCTATTCCCATTTATTATTGCCGCCGCAACCATCCTTCACCTGCTCTTCCTCCATGAAACAGGATCAAATAACCCAATTGGACTAAACTCAGATGCAGACAAAATCTCCTTCCACCCGTACTTTACATATAAAGACTTACTTGGATTCGTACTTATACTCTTAGCCCTTACACTACTAGCATTATTTTCCCCAAACCTGCTAGGAGACCCAGAAAACTTTACCCCCGCCAACCCCCTAGTAACTCCTCCACACATCAAACCAGAATGATACTTTCTATTTGCCTACGCCATCCTCCGATCGATCCCAAATAAACTAGGAGGCGTTCTTGCATTACTATTCTCTATCCTAGTACTAATAGTAGTACCACTTCTACACACCTCAAAACAGCGAGGACTAACATTTCGCCCCCTTACCCAACTTCTATTTTGGACCCTGGTAGCAGACATAATCATCCTTACATGAATTGGAGGCATGCCAGTAGAACACCCCTTCATCATTATCGGACAAATCGCATCCGTTTTATACTTCGCACTGTTCTTAATCTTCTTCCCACTAGCAGGATGATTAGAAAACAAGGCATTAGAATGAGCT

>A._hemispinus

ATGGCAAGCCTACGAAAAACACACCCCCTGATTAAAATTGCTAACGACGCATTAGTTGACCTACCAGCGCCGTCTAACATCTCAGTATGATGAAACTTTGGTTCCCTCCTAGGACTATGTCTAATTACCCAAATTCTGACCGGCCTATTCCTAGCCATACACTACACCTCAGACATTTCGACCGCATTTTCATCAGTAGCCCACATTTGTCGAGACGTAAACTACGGATGGCTAATCCGTAACATCCACGCCAATGGGGCATCATTCTTCTTCATCTGCATCTACATACACATCGCCCGAGGCCTCTATTACGGGTCGTACCTCTACAAAGAAACCTGAAACATCGGCGTAGTCCTTCTTCTACTAGTCATGATGACAGCCTTCGTCGGCTACGTACTCCCATGAGGCCAAATATCCTTCTGAGGCGCCACAGTCATCACAAATCTCCTATCTGCCGTACCATACATAGGAGACATACTAGTTCAATGAATTTGAGGCGGATTCTCAGTAGACAACGCAACACTAACACGATTCTTCGCATTCCACTTCCTACTCCCATTTATTATTGCCGCCGCAACTATCCTTCACCTTCTTTTCCTCCACGAAACAGGATCAAACAACCCAATTGGATTAAATTCAGACGCGGACAAAATCTCTTTCCACCCATACTTCACATATAAAGACTTGCTTGGATTCGTGCTCATACTCCTAGCCCTCACTCTACTGGCACTATTTTCCCCAAACCTTCTAGGAGACCCAGAAAACTTTACCCCCGCCAACCCCTTAGTTACTCCCCCACACATCAAACCAGAGTGATACTTTCTATTTGCCTACGCCATCCTTCGATCGATCCCAAACAAACTAGGAGGCGTTCTTGCATTACTATTTTCTATCCTAGTACTAATAGTAGTACCACTTCTACACACCTCGAAACAACGAGGACTAACATTCCGCCCCCTCACCCAACTTCTATTTTGAACCCTAGTAGCAGATATAATTATCCTTACATGAATTGGAGGCATGCCAGTAGAACATCCGTTCATTATCATCGGACAAATCGCATCCGTTCTATACTTCGCATTATTCCTAATCTTCTTCCCGCTAGCAGGATGATTAGAAAACAAAGCACTAGAATGAGCT

>A._iridescens

ATGGCAAGCCTACGAAAAACACACCCCTTAATTAAAATTGCTAACGACGCACTAGTTGACCTACCAGCACCATCCAACATCTCAGCATGATGAAACTTCGGATCCCTTCTAGGACTATGTTTAGCTACTCAAATTTTAACTGGCCTATTCCTGGCCATACACTACACCTCAGATATCTCAACCGCATTTTCATCAGTAACCCATATTTGCCGAGACGTAAATTACGGCTGACTAATCCGTAATATCCACGCCAACGGAGCATCATTCTTTTTCATCTGCATCTACATACACATCGCCCGAGGCCTCTATTACGGATCTTACCTCTACAAAGAAACTTGAAACATTGGCGTAGTCCTCCTCCTACTAGTTATAATAACAGCTTTCGTCGGCTATGTCCTCCCATGAGGCCAAATATCCTTCTGAGGCGCCACAGTTATTACAAACCTCCTATCTGCTGTACCATATATGGGAGATATACTAGTCCAATGAATCTGAGGCGGATTCTCAGTTGATAACGCAACACTAACACGATTCTTCGCATTCCACTTCCTACTACCATTTATTATTGCCGCCGTAACCATTCTTCACCTTCTTTTCCTCCACGAAACAGGATCAAACAACCCAATTGGACTAAATTCAGACGCAGACAAAATCTCCTTCCACCCATACTTTACATACAAAGACTTACTCGGATTTGTACTAATACTTCTGGCTCTTATACTACTAGCTCTATTCTCCCCAAACTTATTAGGAGACCCAGAAAACTTTACCCCCGCCAACCCCTTAGTTACACCTCCACACATTAAACCAGAGTGATACTTCCTATTCGCTTACGCCATCCTACGATCAATCCCAAACAAACTCGGAGGTGTCCTTGCATTACTATTCTCTATTTTAGTACTAATAGTAGTACCACTTTTACATACCTCTAAGCAACGGGGACTAACATTCCGCCCCCTCACTCAATTCCTATTCTGAACCCTGGTGGCAGATATAATTATCCTCACATGAATTGGGGGCATACCAGTAGAACACCCATTTATTATCATTGGACAAATCGCATCCATCTTATACTTCGCACTGTTCCTAGTCTTTATCCCACTAGCAGGATGACTAGAAAACAAAGCACTAGAATGAGCT

>A._jishouensis

ATGGCAAGCCTACGAAAAACACACCCCCTAATTAAAGTTGCTAACGATGCATTAGTCGACCTACCAGCACCATCTAACATTTCACTGTGATGAAACTTTGGTTCCCTCCTAGGACTATGTTTAATTACCCAAATTCTAACCGGCCTATTCTTAGCCATACACTACACCTCAGACATTTCAACCGCATTTTCATCAGTAGCCCACATTTGTCGAGACGTAAACTACGGATGGCTAATCCGCAACATCCACGCCAATGGGGCATCATTCTTCTTCATCTGCATCTACATGCACATCGCCCGAGGCCTTTATTATGGGTCATACCTCTACAAGGAAACCTGAAACATCGGCGTGGTCCTTCTTCTACTAGTTATGATAACAGCCTTCGTTGGCTATGTGCTCCCATGAGGCCAAATGTCCTTCTGAGGCGCCACAGTTATCACAAATCTTCTATCTGCTGTACCATACATAGGGGACATGCTAGTTCAATGAATCTGAGGCGGATTCTCGGTAGACAACGCGACACTAACACGGTTCTTCGCATTCCACTTCCTACTACCATTCATCATTGCCGCCGCAACTATCCTTCACCTTCTTTTCCTCCATGAAACAGGATCAAATAACCCAATTGGATTAAACTCAGACGCAGACAAAATCTCTTTCCACCCATACTTCACATATAAAGACTTACTTGGGTTTATACTCATGCTCCTAGCGCTTATAATACTAGCATTGTTTTCCCCAAACCTACTAGGAGACCCAGAAAACTTTACCCCCGCTAACCCCTTAGTTACTCCTCCACACATTAAACCAGAATGGTACTTTCTGTTTGCCTACGCTATCCTTCGATCAATCCCAAATAAACTAGGAGGTGTTCTTGCATTACTATTCTCTATCTTAGTACTAATAGTAGTACCACTTCTACACACCTCAAAACAACGAGGACTAACATTCCGCCCCCTCACCCAACTTCTATTTTGAACCCTAGTAGCAGACATAATTATCCTTACATGAATTGGAGGCATACCAGTAGAACACCCATTCATCATTATTGGACAAATCGCATCCGTTCTATACTTCGCACTATTCCTAATCTTCTTCCCACTAGCGGGCTGATTAGAAAATAAAGCATTAGAATGAGCT

>A._kreyenbergii_1

ATGGCAAGCCTACGAAAAACACACCCCTTAATTAAAATTGCTAACGACGCACTAGTTGACCTACCAGCACCATCCAATATCTCAGTGTGATGGAACTTTGGCTCCCTTCTGGGACTATGTTTAATTACCCAAATCCTAACCGGCCTATTTCTAGCTATGCACTACACCTCAGACATTTCAACCGCATTTTCATCAGTGGCCCACATTTGTCGAGACGTAAACTACGGGTGGCTAATCCGCAACATCCACGCCAATGGGGCATCATTCTTCTTCATCTGTATCTACATACACATTGCCCGAGGCCTTTATTACGGGTCATACCTCTACAAAGAAACCTGAAACATCGGCGTAGTCCTTCTTCTTCTAGTTATAATAACAGCCTTTGTGGGCTATGTCCTCCCATGAGGCCAAATATCCTTCTGAGGTGCCACAGTTATCACAAATCTCCTATCCGCCGTACCATATATGGGAGACATGTTAGTTCAATGAATCTGAGGAGGATTCTCAGTAGACAACGCAACATTAACACGATTCTTCGCATTCCACTTCCTATTCCCATTTATCATTGCCGCCGCAACTATTCTTCACCTCCTTTTCCTCCATGAAACAGGATCAAACAACCCAATCGGACTAAACTCAGATGCAGACAAAATCTCCTTCCACCCGTACTTTACATATAAAGACTTACTTGGATTTGTGCTCATACTCTTAGCCCTAACACTACTAGCGTTATTTTCTCCTAGCCTACTAGGAGACCCAGAAAACTTTACCCCCGCTAACCCCTTAGTAACTCCCCCACACATCAAACCAGAGTGGTACTTTCTATTTGCCTACGCCATCCTACGATCAATTCCCAATAAACTAGGAGGTGTTCTTGCATTACTATTCTCTATTCTAGTACTAATAGTAGTACCACTCCTACACACCTCAAAACAACGAGGACTAACATTCCGCCCCCTTACTCAACTTCTATTTTGAACCCTGGTAGCAGACATAATTATCCTTACCTGAATCGGAGGCATGCCAGTAGAACATCCCTACATCATTATCGGACAAATCGCATCCGTTCTATACTTCGCACTATTCCTAATCCTATTCCCACTAGCGGGATGATTAGAAAACAAGGCATTAGAATGAGCT

>A._kreyenbergii_2

ATGGCAAGCCTACGAAAAACACACCCCTTAATTAAAATTGCTAACGACGCACTAGTCGACCTACCAGCACCATCTAATATCTCAGTGTGATGAAACTTTGGCTCCCTTCTGGGACTATGTTTAATTACCCAAATCCTAACCGGCCTATTTCTAGCTATGCACTACACCTCAGACATTTCAACCGCATTTTCATCAGTGGCCCACATTTGTCGAGACGTAAACTACGGATGACTAATCCGCAACATCCACGCCAATGGGGCATCGTTCTTCTTCATCTGCATCTACATGCACATTGCCCGAGGCCTCTATTACGGATCATACCTCTACAAAGAAACCTGAAACATCGGCGTAGTCCTTCTTCTACTAGTCATGATAACAGCCTTTGTGGGCTATGTACTCCCATGAGGCCAAATATCCTTCTGAGGTGCCACAGTTATCACAAATCTCCTATCCGCCGTACCATATATGGGAGACATATTAGTTCAATGAATCTGAGGAGGATTCTCAGTAGACAACGCGACATTAACACGATTCTTCGCATTCCACTTCCTATTCCCATTTATCATTGCTGCCGCAACTATCCTTCACCTCCTCTTCCTCCATGAAACAGGATCAAACAACCCAATCGGGCTAAACTCAGATGCAGACAAAATCTCCTTCCACCCGTACTTTACATATAAAGACCTACTTGGATTTGTACTCATGCTCTTAGCCCTCACACTACTAGCGTTATTTTCCCCTAGCCTACTGGGAGACCCAGAAAACTTTACCCCCGCCAACCCTTTAGTAACTCCCCCGCACATCAAACCAGAGTGGTACTTTCTATTTGCCTACGCCATCCTCCGATCAATCCCTAATAAACTAGGAGGTGTTCTTGCACTACTATTCTCTATCCTAGTACTAATAGTGGTACCACTTCTACACACCTCAAAACAACGAGGACTAACATTCCGCCCCCTTACCCAACTTCTATTTTGAACCCTGGTAGCAGACATAATTATCCTTACATGAATCGGAGGCATGCCAGTAGAACACCCCTTCATCATTATCGGACAAATCGCATCCGTTCTATACTTCGCACTATTCCTAATCTTCTTCCCACTAGCGGGATGATTAGAAAACAAGGCATTAGAATGAGCT

>A._longipinnis

ATGGCAAGCCTACGAAAAACACACCCCTTAATTAAAATTGCTAACGACGCACTAATTGACCTACCAGCACCATCCAACATTTCAGCATGATGAAACTTCGGATCCCTTCTAGGACTATGTTTAGCTACTCAAATTTTAACTGGCCTATTCCTGGCCATACACTACACCTCAGATATCTCAACCGCATTTTCATCAGTAACCCATATTTGCCGAGACGTAAATTACGGCTGACTAATCCGTAATATCCACGCCAACGGAGCATCATTCTTTTTCATCTGCATCTACATACACATCGCCCGAGGCCTCTATTACGGATCTTACCTCTACAAAGAAACTTGAAACATTGGCGTAGTCCTCCTCCTACTAGTTATAATAACAGCTTTCGTCGGCTATGTCCTCCCATGAGGCCAAATATCCTTCTGAGGCGCCACAGTTATTACAAACCTCCTATCTGCTGTACCATACATGGGAGATATACTAGTCCAATGAATCTGAGGCGGATTCTCAGTTGATAACGCAACACTAACACGATTCTTCGCATTCCACTTCCTACTACCATTTATTATTGCCGCCGTAACCATTCTTCACCTTCTTTTCCTCCACGAAACAGGATCAAACAACCCAATTGGGCTAAATTCAGACGCAGACAAAATCTCCTTCCACCCATACTTTACATACAAAGACTTACTCGGATTTGTATTAATACTTCTGGCTCTTATACTACTAGCTCTATTCTCCCCAAACTTATTAGGAGACCCAGAAAACTTTACCCCCGCCAACCCCTTAGTTACACCTCCACACATTAAACCAGAGTGATACTTCCTATTCGCTTACGCCATCCTACGATCAATCCCAAACAAACTCGGAGGTGTCCTTGCATTACTATTCTCTATTTTAGTACTAATAGTAGTACCACTTTTACATACCTCTAAGCAACGGGGACTAACATTCCGCCCCCTCACTCAACTCCTATTCTGAGCCCTGGTGGCAGATATAATTATCCTCACATGAATTGGGGGCATACCAGTAGAACACCCATTTATTATCATTGGACAAATTGCATCCGTCTTATACTTCGCACTGTTCCTAGTCTTTATCCCACTAGCAGGATGACTAGAAAACAAAGCACTAGAATGAGCT

>A._monticola_1

ATGGCAAGCCTACGAAAAACACACCCCCTAATTAAAATCGCTAACGACGCACTAGTCGACCTACCAGCACCGTCCAACATCTCAGCATGATGAAATTTTGGATCCCTCCTAGGACTATGTCTAGCCACTCAAATCCTAACCGGCCTATTCCTAGCCATACACTACACCTCAGATATTTCAACCGCATTTTCATCAGTAACCCATATCTGCCGAGACGTAAATTACGGCTGACTAATCCGCAACATTCACGCCAACGGAGCATCATTCTTCTTCATCTGCATCTATATACATATTGCCCGAGGCCTCTATTACGGATCCTACCTCTATAAAGAAACCTGAAACATTGGCGTAGTCCTCCTCCTCCTAGTCATAATAACAGCCTTCGTTGGCTACGTCCTCCCATGAGGCCAAATATCCTTCTGAGGTGCCACAGTCATTACAAACCTTCTATCCGCCGTACCATATATAGGAGACATGCTAGTTCAATGAATCTGAGGCGGATTCTCAGTAGACAACGCAACACTAACACGATTCTTTGCATTCCACTTCCTGCTTCCGTTTATCATCGCCGCTGCAACCATCCTCCACCTTCTATTCCTTCACGAAACAGGATCAAACAACCCAATCGGACTAAACTCAGACGCGGACAAAATCTCCTTTCACCCATACTTCACATATAAAGACCTACTAGGATTTGTACTTATACTCCTAGCTCTCATACTGCTAGCACTATTCTCCCCCAACCTACTAGGCGACCCAGAAAACTTCACCCCCGCTAACCCCTTAGTTACTCCCCCACATATCAAGCCAGAATGATACTTTCTGTTCGCCTATGCCATCTTACGATCCATCCCAAACAAACTTGGAGGTGTTCTTGCATTACTATTTTCCATCCTGGTCTTAATAGTAGTCCCACTCCTTCACACCTCAAAACAACGAGGATTAACATTCCGCCCAATTACCCAATTCCTGTTCTGAGCCCTAGTGGCAGATATAATTATCTTAACATGAATCGGAGGAATGCCAGTAGAACACCCATTTATTATCATTGGACAAATCGCATCCGTCTTATACTTTGCTTTATTCCTCATCTTTATTCCACTAGCAGGATGGTTGGAAAATAAAGCACTAGAATGAGCT

>A._monticola_2

ATGGCAAGCCTACGAAAAACACACCCCCTAATTAAAATCGCTAACGACGCACTAGTCGACCTACCAGCACCGTCCAACATCTCAGCATGATGAAATTTTGGATCCCTCCTAGGACTATGTCTAGCCACTCAAATCCTAACCGGCCTATTCCTAGCCATACACTACACCTCAGATATTTCAACCGCATTTTCATCAGTAACCCATATCTGCCGAGACGTAAATTACGGCTGACTAATCCGCAACATTCACGCCAACGGAGCATCATTCTTCTTCATCTGCATCTATATACATATTGCCCGAGGCCTCTATTACGGATCCTACCTCTATAAAGAAACCTGAAACATTGGCGTAGTCCTCCTCCTCCTAGTCATAATAACAGCCTTCGTTGGCTACGTCCTCCCATGAGGCCAAATATCCTTCTGAGGTGCCACAGTCATTACAAACCTTCTATCCGCCGTACCATATATAGGAGACATGCTAGTTCAATGAATCTGAGGCGGATTCTCAGTAGACAACGCAACACTAACACGATTCTTTGCATTCCACTTCCTGCTTCCGTTTATCATCGCCGCTGCAACCATCCTCCACCTTCTATTCCTTCACGAAACAGGATCAAACAACCCAATCGGACTAAACTCAGACGCGGACAAAATCTCCTTTCACCCATACTTCACATATAAAGACCTACTAGGATTTGTACTTATACTCCTAGCTCTCATACTGCTAGCACTATTCTCCCCCAACCTACTAGGCGACCCAGAAAACTTCACCCCCGCTAACCCCTTAGTTACTCCCCCACATATCAAGCCAGAATGATACTTTCTGTTCGCCTATGCCATCTTACGATCCATCCCAAACAAACTTGGAGGTGTTCTTGCATTACTATTTTCCATCCTGGTCTTAATAGTAGTCCCACTCCTTCACACCTCAAAACAACGAGGATTAACATTCCGCCCAATTACCCAATTCCTGTTCTGAGCCCTAGTGGCAGATATAATTATCTTAACATGAATCGGAGGAATGCCAGTAGAACACCCATTTATTATCATTGGACAAATCGCATCCGTCTTATACTTTGCTTTATTCCTCATCTTTATTCCACTAGCAGGATGGTTGGAAAATAAAGCACTAGAATGAGCT

>A._paradoxus_1

ATGGCAAGCCTACGAAAAACGCACCCTCTAATTAAAATTGCTAACGACGCATTAGTCGACCTACCCGCACCATCTAACATCTCAGTATGATGAAACTTTGGCTCCCTTCTAGGGCTCTGCTTAATTACCCAGATCCTAACCGGCCTATTCCTAGCCATGCATTACACCTCAGACATTTCAACCGCATTCTCGTCAGTAGCACACATTTGTCGAGACGTAAACTATGGATGACTAATCCGCAGCCTCCACGCCAATGGCGCATCATTCTTCTTCATCTGTATCTACCTCCATATTGCCCGAGGCCTCTATTACGGATCATATCTTTACAAAGAAACCTGAAACATCGGCGTAGTCCTTCTCCTATTAGTTATGATAACAGCCTTTGTCGGCTATGTCCTCCCATGGGGTCAAATATCCTTCTGAGGTGCCACTGTCATCACAAACCTCCTATCTGCCGTGCCATATGTAGGAGACATCCTAGTTCAATGAATTTGAGGCGGATTCTCAGTAGACAACGCAACGCTAACACGATTCTTTGCATTCCATTTCCTACTGCCATTCATCATCGCTGCTATAACCATCCTCCACCTCCTCTTCCTTCATGAAACAGGGTCAAACAATCCAATCGGACTAAACTCGGACGCAGACAAAATCCCCTTCCACCCATACTTTACATACAAAGACCTGCTTGGGTTCGTACTGATACTCCTAGCCCTAGCACTACTAGCATTATTTTCCCCCAACCTTTTAGGAGACCCAGACAACTTTACCCCTGCCAACCCCCTAGTTACTCCCCCACACATCAAACCAGAATGATATTTCCTATTTGCCTACGCCATCCTACGATCAATCCCAAATAAACTAGGAGGTGTTCTTGCACTACTATTTTCTATCCTGGTACTTATAGTAGTGCCACTTCTACACACCTCAAAACAACGAGGACTAACATTCCGCCCCCTAACCCAATTCCTATTCTGAACCCTAGCAGCAGACGTCATGATCCTTACATGAATTGGAGGTATACCAGTAGAACACCCATTCATTGTCATTGGACAAATCGCATCTGTCCTATACTTTGCACTATTCCTAATCTTATTCCCACTAGCAGGATGGTTAGAAAACAAAGCACTAGAATGAACT

>A._paradoxus_2

ATGGCAAGCCTACGAAAAACACACCCCCTAATTAAAGTTGCTAACGATGCATTAGTCGACCTACCAGCACCATCTAACATTTCACTGTGATGAAACTTTGGTTCCCTCCTAGGACTATGTTTAATTACCCAAATTCTAACCGGCCTATTCTTAGCCATACACTACACCTCAGACATTTCAACCGCATTTTCATCAGTAGCCCACATTTGTCGAGACGTAAACTACGGATGGCTAATCCGCAACATCCACGCCAATGGGGCATCATTCTTCTTCATCTGCATCTACATGCACATCGCCCGAGGCCTTTATTATGGGTCATACCTCTACAAGGAAACCTGAAACATCGGCGTAGTCCTTCTTCTACTAGTTATGATAACAGCCTTCGTTGGCTATGTGCTCCCATGAGGCCAAATGTCCTTCTGAGGCGCCACAGTTATCACAAATCTTCTATCTGCTGTACCATACATAGGGGACATGCTAGTTCAATGAATCTGAGGCGGATTCTCGGTAGACAACGCGACACTAACACGGTTCTTCGCATTCCACTTCCTACTACCATTCATCATTGCCGCCGCAACTATCCTTCACCTTCTTTTCCTCCATGAAACAGGATCAAATAACCCAATTGGATTAAACTCAGACGCAGACAAAATCTCTTTCCACCCATACTTCACATATAAAGACTTACTTGGGTTTATACTCATGCTCCTAGCGCTTATAATACTAGCATTGTTTTCCCCAAACCTACTAGGAGACCCAGAAAACTTTACCCCCGCTAACCCCTTAGTTACTCCTCCACACATAAAACCAGAATGGTACTTTCTGTTTGCCTACGCTATCCTTCGATCAATCCCAAATAAACTAGGAGGTGTTCTTGCATTACTATTCTCTATTTTAGTACTAATAGTAGTACCACTTCTACACACCTCAAAACAACGAGGACTAACATTCCGCCCCCTCACCCAACTTCTATTTTGAACCCTAGTAGCAGACATAATTATCCTTACATGAATTGGAGGCATACCAGTAGAACACCCATTCATCATTATTGGACAAATCGCATCCGTTCTATACTTCGCACTATTCCTAATCTTCTTCCCACTAGCGGGCTGATTAGAAAATAAAGCATTAGAATGAGCT

>A._parallens_1

ATGGCAAGCCTACGAAAAACGCACCCCCTGATTAAAATTGCTAACGACGCATTAGTCGACCTACCAGCGCCGTCTAACATCTCAGTATGATGGAACTTTGGTTCCCTCCTAGGACTATGTCTAATTACCCAAATTCTAACCGGCCTATTCCTAGCCATACACTACACCTCAGACATTTCGACCGCATTTTCATCAGTAGCCCACATTTGTCGGGACGTAAACTACGGATGGCTAATCCGTAACATCCACGCCAATGGGGCATCATTCTTCTTCATCTGCATCTACATACACATCGCCCGGGGCCTCTATTACGGGTCATACCTCTACAAAGAAACCTGAAACATCGGCGTAGTCCTTCTTCTACTAGTTATGATGACAGCCTTCGTCGGCTACGTACTCCCATGAGGCCAAATATCCTTCTGAGGCGCCACAGTCATCACAAATCTCCTATCTGCCGTACCGTATATAGGAGACATACTAGTTCAATGAATTTGAGGCGGATTCTCAGTAGACAGCGCAACACTAACACGATTCTTCGCATTCCACTTCCTTCTCCCATTTATCATTGCCGCCGCAACTATCCTTCACCTTCTTTTCCTCCACGAAACAGGATCAAACAACCCAATTGGATTAAATTCAGACGCAGACAAAATCTCTTTCCATCCGTACTTCACATATAAAGACTTGCTTGGATTCGTACTCATACTCCTAGCCCTCACTCTACTAGCACTATTTTCCCCAAACCTTCTAGGAGACCCAGAAAACTTTACCCCCGCCAACCCCTTAGTTACTCCCCCACACATCAAACCAGAGTGGTACTTTCTGTTTGCCTACGCCATCCTTCGATCAATCCCAAATAAACTAGGAGGCGTTCTTGCATTACTATTTTCTATCCTAGTACTAATAGTAGTACCACTTCTACATACCTCGAAACAACGAGGACTAACATTCCGCCCCCTCACCCAACTTCTATTTTGAACCCTAGTAGCAGATATAATTATCCTCACATGAATTGGAGGCATGCCAGTGGAACACCCATTCATTATCATTGGACAAATCGCATCCGTTCTATACTTCGCATTATTCCTAATCTTCTTCCCGCTAGCAGGGTGATTAGAAAATAAAGCACTAGAATGAGCT

>A._parallens_2

ATGGCAAGCCTACGAAAAACACACCCCCTGATTAAAATTGCTAACGACGCATTAGTCGACCTACCAGCGCCATCTAACATCTCAGTGTGATGGAACTTTGGTTCCCTCCTAGGACTATGTCTAATTACCCAAGTTCTGACCGGCCTATTCCTAGCCATACACTACACCTCAGACATTTCGACCGCATTTTCATCAGTAGCCCACATTTGTCGAGACGTAAACTACGGATGGCTAATCCGTAACATTCACGCCAACGGGGCATCATTCTTCTTCATCTGCATCTACATACACATCGCCCGAGGCCTCTATTACGGGTCATACCTCTACAAAGAAACCTGAAACATCGGCGTAGTCCTTCTTCTACTAGTCATGATGACAGCCTTCGTCGGCTACGTACTCCCATGAGGCCAAATATCCTTCTGAGGCGCCACAGTCATCACAAATCTCCTATCTGCCGTACCATATATAGGAGACATATTGGTTCAATGAATTTGAGGCGGATTCTCAGTAGACAACGCAACACTAACACGATTCTTCGCATTCCACTTCCTGCTCCCATTTATCATTGCCGCCGCAACTATCCTTCACCTTCTTTTCCTCCATGAAACAGGATCAAACAACCCAATTGGATTAAATTCAGACGCAGACAAGATCTCTTTCCACCCATACTTCACATATAAAGACTTGCTTGGGTTCATACTCATACTCCTAGCCCTCACTCTACTAGCACTATTTTCCCCGAACCTTCTAGGAGACCCAGAAAACTTTACCCCCGCCAACCCCTTGGTTACTCCTCCACACATCAAACCAGAGTGGTACTTTCTGTTTGCCTACGCCATCCTTCGATCGATCCCAAATAAACTAGGAGGTGTTCTTGCATTACTATTTTCTATCCTGGTACTAATAGTAGTACCACTTCTGCACACCTCGAAGCAGCGAGGACTAACATTCCGCCCCCTCACCCAACTTCTATTTTGAACCCTAGTAGCGGACATAATCATCCTTACATGAATTGGAGGCATGCCAGTAGAACACCCATTCATTATTATTGGACAAATCGCATCCGTTCTATACTTCGCATTATTCCTAATCTTCTTCCCCCTAGCAGGATGATTAGAAAACAAAGCACTAGAATGAGCT

>A._parallens_3

ATGGCAAGCCTACGAAAAACGCACCCCCTAATTAAAATTGCTAACGACGCATTAGTCGACCTACCAGCGCCATCTAACATCTCAGTATGATGGAACTTTGGTTCCCTCCTAGGACTATGTCTAATTACCCAAATTCTAACCGGCCTATTCCTAGCCATACACTACACCTCAGACATTTCGACCGCATTTTCGTCAGTAGCCCACATTTGTCGAGACGTAAACTACGGATGACTAATCCGTAGCATCCACGCCAATGGGGCATCATTCTTCTTCATCTGCATCTACATACACATCGCCCGAGGCCTCTATTACGGGTCATACCTCTACAAAGAAACCTGAAACATCGGCGTAGTCCTCCTTCTACTAGTTATGATGACAGCCTTCGTCGGCTACGTTCTCCCATGAGGCCAAATATCCTTCTGAGGCGCCACAGTCATCACAAATCTCCTATCTGCCGTACCATATATAGGAGACATACTGGTTCAATGAATTTGAGGCGGATTCTCAGTAGACAACGCAACACTAACACGATTCTTCGCATTCCACTTCCTTCTCCCATTTATTATTGCCGCCGCAACTATCCTTCACCTTCTTTTCCTCCACGAAACAGGATCAAACAACCCAATTGGATTAAATTCAGACGCAGACAAAATCTCTTTCCACCCGTATTTCACATATAAAGACTTGCTTGGATTCGTACTCATACTCCTAGCCCTCACTCTACTAGCACTATTTTCCCCAAACCTTCTAGGAGACCCAGAAAACTTTACCCCCGCCAACCCCTTAGTTACTCCTCCACACATCAAACCAGAGTGGTACTTTCTGTTTGCCTACGCCATCCTTCGATCAATCCCAAATAAACTAGGAGGCGTTCTTGCATTACTATTTTCTATCCTGGTACTAATAGTAGTACCACTTCTACACACCTCGAAACAACGAGGACTAACATTCCGCCCCCTCACCCAACTTCTATTTTGAACCCTAGTTGCAGATATAATTATCCTTACATGAATTGGAGGCATGCCAGTAGAACATCCATTCATTATCATTGGACAAATCGCATCCGTTCTATACTTCGCATTATTCCTAATCTTCTTCCCACTAGCAGGGTGATTAGAAAACAAAGCACTAGAATGAGCT

>A._parallens_4

ATGGCAAGCCTACGAAAAACGCACCCCCTGATTAAAATTGCTAACGACGCATTAGTCGACCTACCAGCGCCATCTAACATCTCAGTATGATGGAACTTTGGTTCCCTCCTAGGACTATGTCTAATTACCCAAATTCTAACCGGCCTTTTCCTAGCCATACACTACACCTCAGACATTTCGACCGCATTTTCGTCAGTAGCCCACATTTGTCGAGACGTAAACTACGGATGGCTAATCCGTAACATCCACGCCAATGGGGCATCATTCTTCTTCATCTGCATCTACATACACATCGCCCGAGGCCTCTATTACGGGTCATACCTCTACAAAGAAACCTGAAACATCGGCGTAGTCCTCCTTCTACTAGTTATGATGACAGCCTTCGTCGGCTACGTTCTCCCATGAGGCCAAATATCCTTCTGAGGCGCCACAGTCATCACAAATCTCCTATCTGCCGTACCATATATAGGAGACATACTGGTTCAATGAATTTGAGGCGGGTTCTCAGTAGACAACGCAACACTAACACGATTCTTCGCATTCCACTTCCTTCTCCCATTTATTATTGCCGCCGCAACTATCCTTCACCTTCTTTTCCTCCACGAAACAGGATCAAACAACCCAATTGGATTAAATTCAGACGCAGACAAAATCTCTTTCCACCCGTACTTCACATATAAAGACTTGCTTGGATTCGTACTCATACTCCTAGCCCTCACTCTACTAGCACTATTTTCCCCAAACCTTCTAGGAGACCCAGAAAACTTTACCCCCGCCAACCCCTTAGTTACTCCTCCCCACATCAAACCAGAGTGGTACTTTCTGTTTGCCTACGCCATCCTTCGGTCAATCCCAAATAAACTAGGAGGCGTTCTTGCATTACTATTTTCTATCCTGGTACTAATAGTAGTGCCCCTTCTACACACCTCGAAACAACGAGGATTAACATTCCGCCCCCTCACCCAACTTCTATTTTGAACCCTAGTAGCAGATATAATTATCCTTACATGAATTGGAGGCATGCCAGTAGAACATCCGTTCATTATCATTGGACAAATCGCATCCGTTCTATACTTCGCATTATTCCTAATCTTCTTCCCACTAGCAGGGTGATTAGAAAACAAAGCACTAGAATGAGCT

>A._spinifer

ATGGCAAGCCTACGAAAAACGCACCCTCTAATTAAAATTGCTAACGACGCACTAGTCGACCTACCAGCACCATCTAACATCTCAGTATGGTGAAATTTTGGCTCCCTTCTAGGACTATGCTTAATTACTCAAATCCTGACTGGCCTGTTCCTAGCCATACACTACACCTCAGACATTTCAACCGCATTCTCGTCAGTGGCACACATCTGTCGAGACGTAAATTACGGATGACTAATCCGCAACATCCACGCCAACGGTGCATCATTCTTCTTCATCTGCATCTACCTCCACATTGCCCGAGGCCTCTATTACGGATCATACCTCTACAAAGAAACCTGAAACATCGGCGTAGTCCTCCTCCTGCTAGTTATGATAACAGCCTTTGTTGGCTATGTCCTCCCATGAGGCCAAATATCCTTCTGAGGTGCTACAGTTATCACCAACCTCCTATCTGCCGTACCATATGTAGGGGACATTCTAGTTCAATGAATCTGAGGCGGATTCTCAGTAGACAACGCAACACTAACACGATTCTTCGCATTCCACTTCCTACTGCCATTCATCATTGCTGCTGTAACTATTCTCCACCTCCTTTTCCTTCATGAAACAGGATCAAACAATCCAATTGGACTAAACTCAGACGCAGACAAAATCCCCTTCCACCCATACTTTACATATAAAGACTTACTTGGGTTCGTACTAATACTCCTAGCCCTAGCACTACTAGCGTTATTTTCCCCCAACCTTCTAGGAGACCCAGAAAACTTCACCCCCGCCAACCCCCTAGTTACTCCCCCACACATCAAACCAGAGTGATATTTCCTATTTGCCTACGCCATCCTACGATCAATCCCAAATAAACTAGGAGGCGTTCTTGCATTACTATTTTCTATTCTAGTACTTATAGTAGTGCCACTCCTACACACCTCAAAACAACGAGGACTTACATTCCGCCCCCTCACCCAATTCCTATTCTGAACCCTAGCAGCAGACGTAATAATCCTTACATGAATTGGAGGCATACCAGTAGAACACCCATTTATTATTATTGGACAAATCGCATCTATTCTATACTTCGCACTATTCCTAATCTTATTCCCACTAGCAGGATGGTTAGAAAACAAAGCACTAGAATGAGCT

>A._stenotaeniatus

ATGGCAAGCCTACGAAAAACACACCCTCTAATTAAAATTGCTAACGACGCACTAGTCGACCTACCAGCGCCATCTAACATCTCAGTATGGTGAAATTTTGGCTCCCTTCTAGGACTATGCTTAATTACTCAAATCCTGACTGGCCTGTTCCTAGCCATACACTACACCTCAGACATTTCAACCGCATTCTCGTCAGTGGCACACATCTGTCGAGACGTAAATTACGGATGACTAATCCGCAACATCCACGCCAACGGTGCATCATTCTTCTTCATCTGCATCTACCTCCACATTGCCCGAGGCCTCTATTACGGATCGTACCTCTACAAAGAAACCTGAAACATCGGCGTAGTCCTCCTCCTACTAGTTATGATAACAGCCTTTGTTGGCTATGTCCTCCCATGAGGCCAAATGTCCTTCTGAGGTGCTACAGTTATCACCAACCTCCTATCTGCCGTACCATATGTAGGAGACATTCTAGTTCAATGAATCTGAGGCGGATTCTCAGTAGACAACGCAACACTAACACGATTCTTCGCATTCCATTTCCTACTGCCATTCATTATTGCTGCTGTGACTATTCTCCACCTCCTCTTCCTTCACGAAACAGGATCAAACAATCCAATTGGACTAAACTCGGACGCAGACAAAATCCCCTTCCACCCATACTTCACATATAAAGACTTACTTGGGTTCGTACTAATACTCCTAGCCCTAGCACTACTAGCGTTATTTTCCCCCAACCTTCTAGGAGACCCAGAAAACTTCACCCCCGCCAACCCCCTAGTTACTCCCCCACACATCAAACCAGAGTGATATTTCCTATTTGCCTACGCCATCCTACGATCAATCCCAAATAAACTAGGAGGCGTTCTTGCATTACTATTTTCTATTCTAGTACTTATAGTAGTGCCACTCCTACACACCTCGAAACAACGAGGACTTATATTCCGCCCCCTCACCCAATTCCTATTCTGAACCCTAGCAGCAGACGTAATAATCCTTACATGAATTGGGGGCATACCAGTAGAACACCCATTTATTATTATTGGACAAATCGCATCTATCCTATACTTCGCACTATTCCTAATCTTATTCCCACTAGCAGGATGGTTAGAAAACAAAGCACTAGAATGAGCT

>A._wenchowensis_1

ATGGCAAGCCTACGAAAAACGCACCCATTAATTAAAATCGCTAACGACGCACTAGTCGACCTACCAGCACCATCCAATATCTCAGTATGATGGAACTTTGGCTCCCTCCTGGGACTATGTTTAATTACCCAAATCCTAACCGGCCTATTCCTAGCCATACACTACACCTCAGACATTTCAACCGCATTTTCATCAGTAGCCCACATTTGCCGAGATGTAAACTACGGATGGCTAATCCGCAACATCCACGCCAATGGGGCATCATTCTTCTTCATCTGCATTTACATACACATTGCCCGAGGCCTTTATTACGGGTCTTACCTCTATAAAGAAACCTGAAACATCGGCGTAGTCCTTCTTCTACTAGTCATAATGACAGCCTTTGTAGGCTACGTACTCCCATGGGGCCAAATATCTTTTTGAGGTGCCACAGTTATCACGAATCTCCTGTCCGCCGTACCATATATAGGAGACATATTAGTTCAGTGAATCTGAGGGGGATTCTCAGTAGACAACGCAACATTAACACGATTCTTCGCATTCCACTTCCTATTCCCATTTATCATTGCCGCCGCAACCATCCTTCACCTCCTCTTCCTCCATGAAACAGGGTCAAACAACCCAATTGGACTAAACTCAGATGCAGACAAAATCTCCTTCCACCCGTACTTTACATATAAAGACTTACTTGGATTCGTACTTATACTCTTAGCCCTTACACTACTAGCGTTATTCTCCCCAAACCTGCTAGGAGACCCAGAAAACTTTACCCCCGCTAACCCCCTAGTGACTCCCCCACACATCAAACCAGAATGATATTTTCTGTTTGCCTACGCCATCCTTCGATCAATCCCAAATAAACTAGGAGGTGTTCTTGCATTACTATTCTCTATCCTAGTACTAATAGTAGTACCACTTCTACACACCTCAAAACAACGAGGACTGACATTTCGCCCCCTCACCCAACTTCTATTTTGAACCCTAGTAGCAGACATAATCATCCTTACATGAATTGGGGGCATGCCAGTAGAACACCCCTTCATCATTATCGGACAAATCGCATCCGTTCTATACTTCGCACTATTCCTAATCTTCTTCCCACTAGCAGGATGATTAGAAAACAAGGCATTAGAATGAGCT

>A._wenchowensis_2

ATGGCAAGCCTACGAAAAACGCACCCATTAATTAAAATTGCTAACGACGCACTAGTCGACCTACCAGCACCATCTAATATCTCAGTGTGATGGAACTTTGGCTCCCTTCTGGGACTATGTTTAATTACCCAAATCCTAACCGGCCTATTTCTAGCCATACACTACACCTCAGACATTTCAACCGCATTTTCATCAGTGGCCCACATTTGCCGAGATGTAAACTACGGGTGGCTAATCCGCAACATCCACGCCAACGGAGCATCATTCTTCTTCATCTGCATCTACATACACATTGCCCGAGGCCTCTATTACGGGTCATACCTCTACAAAGAAACCTGAAACATCGGCGTGGTCCTTCTTCTACTAGTCATGATGACGGCCTTTGTAGGCTACGTACTCCCATGAGGCCAAATATCCTTCTGAGGTGCCACAGTCATCACAAATCTCCTATCCGCCGTACCATATATAGGAGACATGTTGGTTCAGTGAATCTGAGGGGGATTCTCAGTAGACAACGCAACATTAACACGATTCTTCGCATTCCACTTCCTATTCCCATTTATCATTGCCGCCGCAACCATCCTTCACCTCCTCTTCCTCCATGAAACAGGATCAAACAACCCAATTGGACTAAACTCAGATGCAGACAAAATCTCCTTCCACCCATACTTTACATATAAAGACTTACTCGGATTCGTACTTATACTCTTAGCCCTTACACTACTAGCGTTATTTTCCCCAAACCTCCTAGGAGACCCAGAAAACTTTACCCCCGCTAACCCCCTAGTAACTCCCCCACACATCAAACCAGAATGATATTTTCTGTTTGCCTACGCCATCCTCCGATCAATCCCAAATAAACTAGGAGGTGTTCTTGCACTACTATTCTCCATTCTAGTACTAATAGTAGTACCACTTCTACACACCTCAAAACAACGAGGACTAACATTTCGCCCCCTTACCCAACTTCTATTTTGAACCCTCGTAGCAGACATAATTATCCTCACGTGAATTGGAGGCATGCCAGTAGAACACCCCTTCATCATTATTGGACAAATCGCATCCGTTCTATACTTCGCACTGTTCTTAATCTTCTTCCCACTAGCGGGATGATTAGAAAACAAGGCATTAGAATGAGCT

>A._wuyiensis

ATGGCAAGCCTACGAAAAACGCACCCTCTAATTAAAATTGCTAACGACGCATTAGTCGACCTACCCGCACCATCTAACATCTCAGTATGATGAAACTTTGGCTCCCTTCTAGGGCTCTGCTTAATTACCCAAATCCTAACCGGCCTATTCCTAGCCATACATTACACCTCAGACATTTCAACCGCATTCTCGTCAGTAGCACACATTTGCCGAGACGTAAACTATGGGTGACTAATCCGCAGCCTCCACGCCAATGGCGCATCATTCTTCTTCATCTGTATTTACCTCCATATTGCTCGAGGCCTCTATTACGGGTCATACCTCTACAAAGAAACCTGAAACATCGGCGTAGTCCTTCTCCTATTAGTTATGATAACAGCCTTTGTCGGCTATGTCCTCCCGTGGGGTCAAATATCCTTCTGAGGGGCCACTGTCATCACAAACCTCCTATCTGCCGTGCCATATGTAGGAGACATCCTAGTTCAATGAATTTGAGGCGGATTCTCAGTAGACAACGCAACACTAACACGATTCTTTGCATTCCATTTCCTACTGCCGTTCATCATCGCTGCTATAACTATCCTCCACCTCCTCTTCCTTCATGAAACAGGGTCAAACAATCCAATCGGACTAAACTCGGACGCAGACAAAATCCCCTTCCACCCATACTTTACATACAAAGACCTACTTGGGTTCGTACTGATGCTCCTAGCCCTAGCACTACTAGCACTATTTTCCCCCAACCTTTTAGGAGACCCAGACAACTTTACCCCTGCCAACCCCCTAGTTACTCCCCCACACATCAAACCAGAATGATATTTCCTATTTGCCTACGCCATCCTACGATCAATCCCAAATAAACTAGGAGGTGTTCTTGCATTACTATTTTCTATCCTGGTACTTATAGTAGTGCCACTTCTACACACTTCAAAACAACGAGGACTAACATTCCGCCCCCTAACCCAATTCCTATTCTGAACCCTAGCAGCAGACGTCATGATCCTTACATGAATTGGAGGTATACCAGTAGAACACCCATTCATTGTCATTGGACAAATCGCATCTGTCCTATACTTTGCACTATTCCTAATCTTATTCCCACTAGCAGGATGGTTAGAAAACAAAGCACTAGAATGAACT

>A._yunnanensis_1

ATGGCAAGCCTACGAAAAACGCACCCCCTAATTAAAATCGCTAACGACGCACTAGTCGACCTACCAGCACCATCCAACATCTCAGCATGATGAAATTTTGGATCCCTCCTAGGGCTATGTTTGGCCACTCAAATCCTAACCGGCCTATTCCTAGCCATACACTACACCTCAGATATTTCAACCGCATTTTCATCAGTAGCCCATATCTGCCGGGACGTAAACTACGGCTGACTAATCCGCAACATTCACGCTAACGGAGCATCATTCTTCTTCATCTGCATCTACATACACATCGCCCGAGGCCTTTACTATGGATCCTACCTTTACAAAGAAACCTGAAACATCGGCGTGATCCTCCTCCTCTTGGTCATAATAACGGCCTTTGTTGGCTACGTCCTTCCATGAGGCCAAATGTCTTTCTGAGGCGCCACAGTCATTACAAACCTCCTGTCTGCCGTACCATACATAGGAGACATACTAGTTCAATGAATCTGAGGCGGATTTTCAGTAGATAACGCAACACTAACACGATTCTTCGCATTCCACTTCCTACTGCCATTCGTTGTCGCTGCCGCAACCATCCTCCACCTACTATTCCTTCACGAAACAGGGTCAAACAACCCAATCGGACTAAACTCAGACGCGGACAAAATCTCCTTCCACCCATACTTTACATATAAAGACCTGCTCGGATTCGTACTCATACTTCTGGCTCTTATGCTACTAGCATTATTTTCCCCTAATCTACTAGGAGACCCAGAGAACTTCACCCCCGCTAATCCACTTGTCACTCCCCCACACATTAAACCAGAATGATACTTTCTGTTCGCTTATGCAATTTTACGATCAATTCCAAACAAACTTGGGGGTGTCCTTGCATTACTATTTTCTATCCTAGTATTAATAGTGGTCCCACTCCTGCACACCTCAAAACAACGAGGACTAACATTCCGCCCAATTACCCAATTCCTATTCTGAACCCTGGTAGCAGACATAATTATTTTAACATGAATCGGAGGCATACCAGTAGAACACCCATTTATCATTATTGGACAAATCGCATCCGTTTTATATTTCGCACTATTCCTTGTCTTTATCCCACTAGCAGGATGGTTAGAAAATAAAGCACTAGAATGAGCC

>A._yunnanensis_2

ATGGCAAGCCTACGAAAAACGCACCCCCTAATTAAAATCGCTAACGACGCACTAGTCGACCTACCAGCACCATCCAACATCTCAGCATGATGAAATTTTGGATCTCTCCTAGGGCTATGTTTGGCCACTCAAATCCTAACCGGCCTATTCCTAGCCATACACTACACCTCAGATATTTCAACCGCATTTTCATCAGTAGCTCATATCTGCCGGGACGTAAACTACGGCTGACTAATCCGCAACATTCACGCTAACGGAGCATCATTCTTCTTCATCTGCATCTACATGCACATCGCCCGAGGCCTTTATTATGGGTCCTACCTTTATAAAGAAACCTGAAACATCGGCGTGGTCCTCCTCCTCTTGGTCATAATAACAGCCTTTGTCGGCTACGTCCTTCCATGAGGCCAAATGTCTTTCTGAGGCGCCACAGTCATCACAAACCTCCTGTCCGCCGTACCATACATAGGGGACATACTAGTTCAATGAATCTGAGGCGGATTTTCAGTAGATAACGCAACACTAACACGATTCTTCGCATTCCACTTCCTACTGCCATTCGTTGTCGCTGCCGCAACCATCCTCCACCTCCTATTCCTTCACGAAACAGGGTCAAACAACCCAATTGGACTAAACTCAGACGCGGACAAAATCTCCTTCCACCCATACTTTACATATAAAGACCTGCTCGGATTCGTACTTATACTTCTGGCTCTTATACTACTAGCATTATTTTCCCCTAATCTACTAGGAGACCCAGAGAACTTCACCCCCGCTAATCCACTAGTCACTCCTCCACACATTAAACCAGAATGATACTTTCTATTCGCTTATGCAATTTTACGATCAATTCCAAACAAACTTGGGGGTGTCCTTGCATTACTATTCTCTATCCTAGTATTAATAGTGGTCCCACTCCTACACACCTCAAAACAACGAGGACTAACATTCCGCCCAATTACCCAATTCCTATTCTGAACCCTGGTGGCAGATATAATTATTTTAACATGAATCGGAGGCATACCAGTAGAACACCCATTTATCATCATTGGACAAATCGCATCCGTTTTATATTTCGCACTATTCCTTGTCTTCATTCCACTAGCAGGATGGTTAGAAAATAAAGCACTAGAATGAGCC

>Onychostoma_barbatulum

ATGGCAAGCCTACGAAAAACACACCCCTTAATTAAAATCGCCAACGACGCACTAATTGACCTACCAGCACCATCTAATATCTCAGCATGATGAAACTTTGGATCCCTCCTAGGACTATGTTTAGCTACTCAAATCTTAACCGGCCTATTCCTAGCTATACACTACACCTCGGACATTTCAACCGCATTTTCATCAGTAGTCCACATCTGCCGAGACGTAAACTACGGATGACTAATCCGCAACATCCACGCTAATGGAGCATCGTTCTTCTTCATCTGCATCTACATACACATCGCCCGAGGCCTCTATTACGGATCTTACCTTTATAAAGAAACCTGAAACATCGGCGTAATTCTCCTCCTACTAGTCATAATGACAGCCTTTGTCGGCTATGTCCTCCCATGAGGCCAAATATCCTTCTGGGGCGCCACAGTTATCACAAATCTCCTATCTGCCGTACCATATATAGGAGATATACTAGTTCAATGAATCTGAGGCGGATTCTCAGTTGATAATGCAACACTAACACGATTCTTCGCATTCCACTTCCTATTCCCATTTGTTATTGCCGGAGCAACCATCCTACACCTTCTATTTCTTCATGAAACAGGATCAAACAACCCAGTTGGATTAAACTCAGATGCAGACAAAATCTCTTTCCACCCATACTTTACATACAAAGATTTACTAGGATTCGTACTTATACTCCTGGCTTTAATACTACTAGCGCTATTTTCCCCTAACCTCCTAGGAGACCCAGAAAACTTTACCCCCGCCAACCCACTAGTTACCCCCCCACATATCAAACCAGAATGATACTTCTTATTCGCCTACGCCATCCTGCGATCAATCCCAAATAAACTTGGGGGCGTCCTTGCACTACTGTTCTCCATCTTAGTACTTATAATGGTGCCCCTACTACATACATCAAAACAACGAGGACTAACATTTCGCCCACTCACTCAGCTCCTATTTTGAACCCTGGTAGCAGACATAATTATCCTGACATGAATTGGAGGCATACCAGTAGAACACCCATTCATCATTATCGGACAAATTGCATCTATCTTATACTTTGCACTATTTCTAATCTTTATACCACTAGCAGGGTGATTAGAAAACAAAGCACTAGAATGAGCT

>Onychostoma_meridionale

ATGGCAAGCCTACGAAAAACACACCCCCTAATTAAAATTGCTAACGACGCGCTAGTTGATCTACCAGCACCATCCAACATTTCAGCATGATGAAACTTCGGATCCCTCCTGGGACTATGCCTGGCCACCCAAATCCTGACCGGCCTATTCCTAGCCATGCACTACACCTCAGATATTTCAACCGCATTTTCATCAGTAACCCATATCTGTCGAGACGTAAACTACGGCTGATTAATCCGCAACATCCATGCTAATGGCGCATCATTCTTCTTCATCTGCATCTACATACACATCGCCCGAGGCCTATACTACGGATCCTACCTCTACAAAGAAACCTGAAACATCGGCGTAATTCTGCTCCTACTAGTTATAATAACAGCCTTTGTAGGCTATGTCCTCCCATGAGGCCAAATATCCTTCTGAGGCGCTACAGTTATTACAAATCTACTATCTGCCGTACCATATATGGGAGACATATTAGTTCAATGAATCTGAGGCGGATTTTCAGTAGACAACGCGACATTAACACGATTCTTCGCATTCCACTTCCTGCTTCCATTTGTTATTGCCGCTGCAACCATCCTCCACCTCCTGTTCCTCCACGAAACAGGATCAAACAACCCGATTGGCTTAAACTCAGACGCAGACAAAATCTCTTTTCACCCATACTTTACCTACAAAGACCTACTCGGATTCGTAATTATGCTTCTAGCCCTCATACTACTAGCGTTATTTTCCCCCAACCTTCTAGGAGACCCAGAAAACTTTACCCCCGCCAACCCATTGGTCACCCCTCCACACATCAAACCAGAATGATATTTCCTGTTTGCCTATGCCATCCTACGATCCATCCCTAACAAGCTCGGGGGAGTCCTCGCATTACTATTTTCTATTCTAGTACTAATAGTCGTGCCACTTCTCCATACTTCAAAACAACGAGGACTCACATTCCGCCCCCTCACCCAATTCTTATTCTGAACCCTAGTGGCAGACATAATAATCCTAACATGAATTGGAGGCATGCCGGTAGAACACCCATTTATTATCATTGGCCAAGTCGCATCTGTCTTGTACTTTGCCCTATTCCTAATCTTTATTCCACTAGCAGGATGGCTAGAAAATAAAGCACTAGAATGAGCT

>Onychostoma_gerlachi

ATGGCAAGCCTACGAAAGACACACCCCCTAATTAAGATTGCTAACGACGCACTAGTTGATCTACCAGCACCATCTAACATCTCAGTATGATGAAACTTCGGGTCCCTCCTAGGACTATGCCTAATCACTCAGATTCTAACCGGCCTATTCCTAGCCATACACTACACTTCGGATATTTCAACCGCATTTTCATCAGTAGTCCACATCTGCCGAGACGTAAACTACGGCTGATTAATCCGCAACATCCATGCCAATGGCGCATCATTCTTCTTCATCTGCATCTACATACACATCGCCCGAGGCCTATACTACGGATCCTACCTCTACAAAGAAACCTGAAATATCGGCGTAGTTCTACTCCTACTAGTTATAATAACAGCCTTCGTGGGCTATGTTCTACCATGAGGACAAATATCCTTTTGAGGCGCCACAGTCATTACAAATCTACTATCTGCCGTACCATATATGGGAGATATACTAGTACAATGAATCTGAGGCGGATTTTCAGTAGACAACGCAACACTAACACGATTCTTCGCGTTCCACTTCCTATTCCCATTCGTCATTGCCGCCGCAACCATCCTTCACCTCCTATTCCTCCACGAAACAGGATCAAACAACCCAATTGGCTTAAATTCAGACGCAGACAAAATCTCTTTCCACCCGTACTTTACCTATAAAGACCTACTTGGATTCGTAATTATGCTTTTAGCCCTGATACTTCTAGCATTATTTTCCCCCAACCTCTTAGGGGACCCAGAAAACTTTACCCCCGCCAATCCACTAGTTACTCCTCCACACATCAAGCCAGAATGATATTTCCTATTTGCCTACGCCATCCTGCGATCCATCCCTAACAAGCTCGGAGGAGTCCTTGCATTACTATTCTCTATTCTAGTACTAATAGTGGTGCCACTTCTACACACTTCAAAACAACGAGGACTAACATTCCGCCCACTCACCCAATTCTTATTCTGAACCCTAGTAGCAGACATAATAATTCTAACATGAATTGGGGGCATACCAGTAGAACACCCGTTCATTATTATTGGCCAAGTCGCATCTGTCTTGTACTTTGCCCTATTCCTAGTCTTTATTCCACTAGCAGGATGGTTAGAAAATAAAGCACTAGAATGAGCT

>Spinibarbus_denticulatus

ATGGCAAGCCTACGAAAAACACACCCTCTAATTAAAATCGCTAATGACGCGCTAGTTGATTTACCCGCACCATCCAACATCTCAGTATGATGAAACTTCGGGTCCCTCCTAGGATTATGTCTAGCTACTCAAATCCTAACCGGCCTATTCCTAGCTATACACTACACCTCAGATATCTCAACCGCATTCTCATCAGTAACCCACATCTGCCGAGACGTAAACTACGGTTGACTAATCCGCAATGTACACGCCAACGGAGCATCATTCTTCTTCATCTGCATCTACATGCACATTGCCCGAGGCCTATATTACGGGTCGTATCTTTATAAAGAAACCTGAAACATCGGTGTAATTCTCCTGCTGCTAGTCATAATGACAGCCTTCGTCGGATATGTCCTCCCATGAGGTCAAATATCCTTTTGAGGCGCTACAGTAATTACAAACCTTCTATCCGCCGTACCATACATAGGAGATATGCTAGTTCAATGAATCTGAGGTGGATTTTCAGTAGACAACGCAACACTAACGCGATTCTTCGCATTCCACTTCCTTCTACCATTCATTATTGCTGCCATAACCATCCTACATCTCCTCTTCCTCCACGAGACAGGATCAAATAATCCAGTTGGTTTAAATTCAGACGCGGACAAAATCTCTTTCCACCCATACTTCACATACAAAGACCTACTTGGATTCGTAATTATACTTCTAGCTCTCACTCTACTAGCATTATTTTCCCCCAACCTATTAGGAGACCCAGAAAACTTCACCCCCGCTAATCCCCTAGTCACCCCTCCACATATTAAACCAGAATGATACTTCCTATTTGCCTATGCCATTCTACGATCAATCCCAAATAAACTTGGGGGTGTCCTTGCATTACTGTTCTCCATCCTAGTACTAATAATAGTACCACTACTACACACCTCAAAACAACGAGGACTAACATTCCGTCCAATCACCCAATTTCTATTTTGAACCTTAGTAGCAGACATAGTCATCTTAACATGAATTGGAGGTATACCAGTAGAACATCCATTTATTATTATTGGGCAAATTGCATCCGTCTTATACTTTGCATTATTCCTAATTTTTATTCCCCTAGCAGGATGATTGGAAAACAAAACACTAGAATGAGCT

>Spinibarbus_hollandi

ATGGCAAACCTACGAAAGACACACCCCCTAATTAAAATTGCTAACGACGCACTAGTTGACTTACCCGCACCATCTAACATCTCAGCATGATGAAACTTTGGATCCCTCCTAGGGCTATGCCTAGCTACTCAAATCCTGACTGGTCTATTTCTAGCTATACACTACACCTCAGATATTTCAACCGCATTCTCATCAGTAACCCACATCTGCCGAGACGTAAACTACGGTTGATTAATCCGCAATGTACACGCCAACGGAGCATCATTCTTCTTTATCTGCATCTATATGCACATTGCCCGAAGCCTATACTACGGGTCATATCTTTATAAAGAAACCTGAAATATTGGCGTAATTCTCCTACTACTAGTTATAATGACAGCCTTTGTCGGATATGTCCTCCCATGAGGCCAAATATCCTTTTGAGGCGCTACAGTAATTACAAACCTTCTATCCGCCACACCATATATAGGAGATATACTAGTTCAATGAATCTGAGGTGGATTCTCAGTAGATAACGCAACACTAACGCGATTCTTCGCATTCCACTTCCTACTACCATTCATTATTGCTGCCGCAACCATTCTGCATCTCCTTTTCCTCCACGAAACAGGGTCAAATAACCCAGCTGGTTTAAATTCAGACGCGGACAAGATCTCTTTCCACCCATACTTCACATACAAAGACCTACTTGGATTCGTAATTATACTCCTACTCCTCACTCTATTAGCACTATTTTCCCCCAGCCTGTTAGGAGACCCAGAAAACTTCACCCCCGCCAACCCCTTAGTCACTCCTCCACATATTAAACCAGAATGATACTTCCTATTTGCATACGCCATTTTACGATCAATCCCAAATAAACTTGGAGGTGTTCTTGCATTATTATTCTCGATTTTAGTACTAATAATAGTACCACTACTACACACCTCAAAACATCGAGGACTAACATTCCGCCCAATCACCCAATTCCTATTTTGAGCCTTAGTAGCAAACATAGCTATTCTAACATGAATTGGGGGTATACCAGTAGAACACCCATTTATCATTATTGGGCAAACTGCATCCGTCTTATACTTCTCATTATTCCTGATCTTTATCCCACTAGCAGGTTGACTGGAAAATAAAACGTTAGAGCAAACC

>Spinibarbus_sinensis

ATGGCAAGCCTACGAAAAACACACCCCCTTATTAAAATCGCTAATGACGCACTAGTCGACCTACCTGCACCATCCAATATTTCAGTATGATGAAACTTTGGATCTCTTCTAGGACTATGCCTAGCTACTCAAATCCTAACTGGCCTATTCTTAGCCATACACTATACCTCAGATATCTCAACCGCATTCTCATCAGTAGTCCACATCTGCCGTGACGTTAACTACGGCTGATTAATCCGCAACGTACACGCTAATGGAGCATCATTCTTCTTCATCTGCATCTATATACATATTGCCCGAGGTCTATACTATGGGTCTTACCTTTACAAAGAAACCTGGAACATCGGCGTAGTTCTCCTGCTACTCGTTATAATAACAGCCTTTGTCGGCTATGTTCTTCCGTGAGGACAAATATCCTTCTGAGGTGCCACAGTAATCACAAATCTCCTATCCGCCGTACCATATATGGGAGACATACTAGTTCAATGAATCTGGGGTGGCTTTTCAGTAGACAACGCAACACTAACACGATTCTTTGCATTTCACTTCCTCCTGCCATTTGTTATTGCTGCCATAACCATCCTACACCTTCTATTTCTCCACGAAACAGGGTCAAATAACCCAATCGGACTAAACTCAGACGCAGACAAAATCTCCTTCCATCCATACTTCACATACAAAGACCTACTTGGATTCGTAGTCATACTTCTAGCTCTTACACTACTGGCACTATTCTCCCCAAACCTACTAGGAGACCCAGAAAACTTCACCCCCGCTAACCCCCTAGTTACTCCTCCACATATTAAGCCAGAATGATACTTCTTATTCGCCTACGCCATCCTACGATCAATCCCAAATAAACTTGGAGGTGTCCTTGCACTACTGTTCTCTATTCTAGTATTAATAGTAGTACCACTACTACATACCTCAAAACAACGAGGACTAACATTCCGCCCAATCACCCAATTCCTATTCTGAACCTTAGTAGCAGATATAGTAATCTTAACATGAATTGGAGGTATACCAGTAGAGCACCCGTTCATCATCATTGGACAAATCGCATCCGTCCTATACTTCGCACTATTCCTCATTTTTATCCCACTAGCAGGATGGTTAGAAAATAAAGCACTAGAATGAGCT

**ND1**

>A._barbodon

ATGCTAAACATCCTGATTACCCACTTAATTAACCCTTTAGCCTACATCGTACCAGTACTCCTAGCGGTAGTTTTCTTAACACTAGTCGAACGAAAAGTACTAGGATATATGCAACTGCGAAAAGGACCAAACGTGGTAGGACCCCACGGACTTCTACAACCCATTATTGATGGGGTAAAGCTCTTCATTAAAGAACCCGTCCGCCCATCCACATCATCCCCATTCCTATTTTTAGCCGCCCCCATGCTCGCACTAACTCTGGCCATAATCCTATGAGCACCAATCCCTATACCCCACCCAATAATTGACCTCAACTTAGGAATCCTATTTATCCTGGCCCTGTCAAGCCTCACAGTCTACTCAATCCTTGGATCAGGCTGAGCATCAAATTCAAAATACGCATTAATTGGTGCCCTACGGGCTGTGGCCCAAACAATTTCCTATGAAGTAAGCTTAGGACTAATTCTCCTCTCCGTGATCATCTTCTCAGGAGGGTACACCCTACAAACATTTAATATCACTCAAGAAAGCATTTGACTACTCATTCCTGCCTGACCCCTAGCCGCAATATGATATATCTCAACACTAGCCGAAACCAACCGAGCACCCTTCGACCTAACAGAAGGAGAATCAGAACTAGTATCCGGCTTCAACGTAGAATATGCAGGAGGACCATTTGCACTCTTCTTCCTAGCCGAATATGCTAACATCCTTCTAATAAACACCCTCTCAGCCGTACTGTTCCTAGGAGCCTCACACATCCCCAACATCCCTGAACTCACAACAATTAATCTCATAACTAAAGCTGCCCTATTATCCGCCGTATTCCTATGAGTACGAGCCTCCTACCCACGATTTCGATATGATCAACTAATACACCTAGTATGAAAAAACTTCCTTCCTCTCACACTCGCCTTTGTGCTATGACACGCCGCCCTGCCAATCGCCCTAGCAGGACTCCCCCCACAACTA

>A._beijiangensis_1

ATGCTAAACATCCTGATTACCCATTTAATTAACCCATTAGCCTACATCGTGCCAGTACTTCTAGCAGTAGCCTTCCTAACACTAGTTGAACGAAAAGTACTAGGTTATATACAACTGCGAAAAGGGCCAAATGTAGTAGGACCCTGCGGACTCCTCCAACCCATCGTTGATGGAGTAAAACTATTTATCAAAGAACCCATCCGCCCATCCACAGCATCCCCCCTCCTATTTTTAACCGCCCCCATGCTCGCACTGACCCTAGCCATAGTCTTATGAGCACCAATTCCTATGCCCTACCCAGTAATTGACCTCAACCTAGGAATTCTATTTATCCTTGCCCTATCAAGCCTTACAGTATACTCAATCTTAGGATCAGGCTGAGCATCAAACTCAAAATACGCACTAATCGGTGCCCTACGAGCCGTGGCCCAAACAATTTCTTATGAGGTAAGCCTAGGACTAATTCTTCTCTCCGTAATTATCTTTTCAGGAGGGTACACCCTACAGACATTCAACACCACCCAAGAAAGCATTTGACTGCTCATCCCTGCCTGACCCCTGGCCGCAATATGATATATCTCAACACTAGCTGAAACAAACCGAGCACCATTTGACCTAACAGAAGGAGAATCAGAACTAGTCTCTGGTTTCAACGTAGAATATGCAGGAGGACCCTTTGCACTCTTCTTCCTAGCCGAATACGCCAATATCCTTTTAATAAACACCCTCTCAGCCGTACTATTCCTAGGAGCCTCACACATCCCCTACATCCCCGAACTTACAACAATCAACCTCATAATCAAGGCTGCACTGCTATCAGCCGTTTTCCTATGAGTACGAGCCTCATACCCCCGATTCCGATATGACCAACTAATACACCTAGTGTGAAAGAACTTTCTTCCCCTGACACTCGCCTTCGTCCTATGACACACCGCCCTACCAATTGCCCTGGCAGGACTCCCCCCACAACTA

>A._beijiangensis_2

ATGCTAAACATCCTAATTACCCATTTAATCAACCCATTAGCCTACATCGTACCAGTACTTCTAGCAGTAGCCTTCCTAACACTAATTGAACGAAAAGTACTAGGTTATATACAACTGCGAAAAGGGCCAAATGTAGTAGGACCCTACGGACTCCTCCAACCCATCGCTGATGGAGTAAAACTATTTATCAAAGAACCCATCCGCCCATCCACAGCATCCCCCCTCCTATTTTTAGCCGCCCCCATGCTCGCGCTGACCCTGGCCATAGTCTTATGAGCACCAATTCCTATGCCCTACCCAGTAATTGACCTCAATCTAGGAATTCTATTCATCCTTGCCCTATCAAGCCTTACAGTATACTCAATCTTAGGATCAGGCTGAGCATCAAACTCAAAATACGCACTAATCGGTGCCCTACGAGCCGTAGCCCAAACAATTTCTTATGAAGTAAGCTTAGGACTAATTCTTCTCTCCGTAATTATCTTTTCAGGAGGGTACACCCTACAGACATTCAACACCACCCAAGAAAGCATTTGGCTGCTCATCCCTGCCTGACCCCTGGCCGCAATATGATACATCTCAACACTAGCCGAAACAAACCGAGCACCATTTGACCTAACAGAAGGAGAATCAGAACTAGTCTCTGGTTTCAACGTAGAATATGCAGGAGGACCCTTTGCACTCTTCTTCCTAGCCGAATACGCCAATATCCTTTTAATAAACACCCTCTCAGCCGTACTATTCCTAGGAGCCTCACACATCCCCAACATCCCCGAACTTACAACAATCAACCTCATAATCAAGGCTGCACTGCTATCAGCCGTTTTCCTATGAGTACGAGCCTCGTATCCCCGATTCCGATATGACCAACTAATACACTTAGTGTGAAAGAGCTTTCTTCCCCTCACACTAGCCTTCGTCCTATGACACACCGCCCTACCAATTGCCCTGGCAGGACTCCCCCCACAACTA

>A._fasciatus

ATGCTAAACACCCTAATCACCCACTTAATTAACCCACTAGCCTACATCGTACCAGTGCTGCTAGCAGTGGTCTTCCTAACACTGGTTGAACGAAAAGTATTAGGCTATATACAGCTACGAAAAGGGCCAAATGTAGTAGGACCTTATGGACTCCTTCAACCCATTGTTGACGGGGTAAAACTATTTACCAAAGAACCCGTCCGCCCATCCACAGCATCTCCACTTTTATTCCTAGCTGCCCCCGTGCTCGCACTGACTCTGGCCATAATCCTATGAGCACCAATCCCCATGCCCTACCCAATAATTGACCTCAACCTAGGAATCCTATTTATCCTCGCCCTCTCAAGCCTTGCAGTATACTCAATTCTAGGATCAGGCTGAGCATCAAATTCAAAGTACGCACTAATCGGGGCCCTACGGGCCGTGGCCCAAACAATCTCTTATGAAGTGAGCTTAGGACTAATCCTTCTCTCTGTAATTATCTTTTCAGGGGGGTACACCCTACAAACATTCAATACCACCCAAGAAAGCATTTGACTACTCATCCCCGCATGGCCCCTAGCCGCAATATGATACATCTCAACATTAGCTGAAACAAACCGGGCACCATTCGACTTAACAGAGGGGGAGTCAGAACTAGTATCTGGTTTTAACGTAGAATATGCAGGGGGACCCTTTGCGCTTTTCTTCCTGGCTGAGTACGCCAACATCCTTTTAATAAATACCCTATCGGCCGTATTATTTTTAGGAGCCTCACATATCCCCAGCATCCCCGAACTCACAACAGTCAATCTTATGATTAAAGCTGCACTACTATCAGCTGTATTCCTATGAGTGCGAGCCTCATATCCCCGATTCCGATACGACCAACTCATACACCTAGTCTGAAAAAACTTTCTTCCTCTCACACTCGCCTTCGTTCTATGACACACCGCCCTGCCAATCGCCCTGGCAGGGCTCCCCCCACAACTA

>A._hemispinus

ATGCTAAACATCCTTATCACCCACTTAATCAACCCCTTAGCCTACATCGTACCAGTACTCCTAGCAGTGGCCTTCCTAACACTAGTTGAACGAAAAGTACTAGGCTATATGCAACTACGAAAAGGACCAAACGTAGTAGGACCTTATGGACTCCTTCAACCCATTATAGACGGGGTAAAACTATTTACCAAAGAACCCGTCCGCCCATCTACAGCATCCCCACTTTTATTTTTGGCCGCCCCAGTGCTCGCACTAACTCTGGCCATAATCCTATGAGCACCAATTCCCATGCCCTACCCAATAATTGACCTTAACCTAGGAATCCTGTTCATCCTTGCCCTATCAAGCCTTGCAGTATACTCAATCCTAGGATCAGGCTGAGCATCAAATTCAAAATATGCACTAATCGGAGCCCTACGGGCCGTAGCCCAAACAATTTCCTATGAAGTAAGCTTAGGACTAATCCTTCTCTCTGTAATTATCTTTTCAGGGGGATACACCCTGCAAACATTTAATACCACCCAAGAAAGCGTTTGACTACTCATCCCTGCCTGACCCCTAGCCGCAATATGATATATCTCAACACTAGCTGAAACAAACCGGGCACCCTTCGACCTAACAGAGGGGGAATCAGAACTAGTATCTGGCTTCAACGTAGAATATGCAGGAGGACCCTTCGCACTCTTCTTCTTGGCTGAGTACGCCAACATTCTTTTAATAAATACCCTATCAGCCGTACTATTTCTAGGGGCCTCGCACATCCCCAGCATCCCCGAACTTACAACAATTAACCTCATAATTAAAGCTGCACTACTATCAGCTGTATTCCTATGGGTACGAGCCTCATACCCCCGGTTCCGATACGACCAACTCATACACCTAGTCTGGAAAAACTTCCTTCCCCTCACACTCGCCTTCGTACTTTGACACACCGCCCTACCAATTGCCCTGGCAGGGCTCCCCCCACAACTA

>A._iridescens

ATGCTAAACATCCTAATTACCCACTTAATTAACCCCTTAGCCTACATCGTACCAGTACTCCTAGCGGTAGCCTTCTTAACACTAATTGAACGAAAAGTACTAGGATACATGCAACTGCGAAAGGGACCAAACGTGGTAGGACCTTACGGACTTCTACAACCCATTGTTGATGGGGTAAAGCTATTCATTAAAGAACCCGTCCGCCCATCCACATCATCCCCGTTCCTATTTTTAGCCACCCCCATGCTCGCACTAACTCTGGCCATAATCCTATGAGCACCAATCCCTATACCCCACCCAATAATTGACCTCAACCTAGGAATCCTATTTATCCTGGCCCTATCAAGCCTTGCAGTCTACTCAATCCTTGGATCAGGCTGAGCATCAAATTCAAAATACGCACTAATTGGTGCCCTACGGGCTGTGGCCCAAACAATTTCCTATGAAGTAAGCTTAGGACTAATTCTCCTCTCCGTGATCATCTTCTCAGGAGGGTACACCCTACAAACATTCAACACCACTCAAGAAAGCATTTGACTACTCATTCCTGCCTGACCCCTAGCCGCAATATGATATATCTCAACACTAGCCGAAACTAACCGAGCACCCTTCGACCTAACAGAGGGAGAGTCAGAACTAGTATCCGGCTTCAACGTAGAATATGCAGGAGGACCATTTACACTCTTCTTCCTGGCCGAATATGCTAACATCCTTCTAATAAATACCCTCTCAGCCGTACTATTCCTAGGAGCCTCACACATCCCTAACATCCCTGAACTCACAACAATTAATCTCATAACTAAAGCTGCCCTATTATCCGCCGTATTCCTATGAGTACGAGCCTCCTACCCACGATTTCGATACGACCAACTAATACACCTAGTATGAAAAAACTTCCTTCCCCTCACACTCGCCTTTGTGTTATGACACGCCGCCCTGCCAATCGCCCTAGCAGGACTCCCCCCACAACTA

>A._jishouensis

ATGCTAAACATCCTAATCACCCACTTAATTAACCCATTAGCCTACATCGTACCAGTATTGCTAGCAGTGGCCTTCCTGACACTAGTCGAGCGAAAAGTACTAGGCTATATGCAACTACGAAAAGGGCCAAATGTAGTAGGACCTTACGGACTCCTTCAACCCATTGTCGACGGGGTAAAACTATTTACCAAGGAACCCATCCGCCCATCCACAGCATCCCCACTTTTGTTCTTAGCTGCCCCCGTGCTCGCACTAACTCTAGCCATGATCTTATGAGCACCCATTCCCATGCCCTATCCAATAATTGACCTCAACCTAGGAATCCTATTTATCCTTGCCCTATCAAGCCTTGCGGTATACTCAATCTTGGGGTCGGGCTGAGCATCAAATTCAAAATACGCACTAATTGGAGCCCTACGGGCCGTGGCCCAAACAATTTCCTATGAAGTAAGCTTAGGATTAATCCTTCTCTCTGTAATTATCTTTTCAGGAGGGTACACCCTACAAACATTTAACACCACCCAGGAAAACATTTGACTACTCATCCCTACCTGACCCCTAGCCGCAATATGATATATCTCAACACTAGCTGAAACAAACCGGGCACCCTTCGACCTAACAGAGGGGGAATCAGAGCTAGTATCTGGTTTCAACGTAGAATATGCAGGGGGACCCTTTGCACTCTTCTTCTTGGCTGAGTACGCCAACATCCTTTTAATAAATACCCTATCAGCCGTGCTATTTCTAGGAGCCTCACACATCCCCAGCATCCCCGAACTTACAACAATTAACCTCATGATTAAAGCTGCACTGCTATCAGCTGTATTCCTATGAGTACGAGCCTCATACCCTCGATTCCGATATGATCAACTCATACACCTAGTCTGAAAGAACTTTCTTCCCCTCACACTCGCCTTTGTACTATGACACACCGCCCTACCAATTGCCCTAGCAGGACTCCCCCCACAGCTA

>A._kreyenbergii_1

ATGCTAAACATCCTAATTACCCACTTAATTAACCCACTAGCCTACATCGTACCAGTACTGCTAGCAGTGGCCTTCCTTACACTAGTTGAACGGAAAGTACTAGGCTATATGCAACTACGAAAGGGGCCGAATGTGGTAGGACCTTATGGACTCCTTCAGCCCATTGTCGATGGAGTAAAACTGTTTACCAAAGAGCCCGTACGCCCATCCACAGCATCCCCGCTCCTATTCCTAGCCGCCCCCGTGCTCGCGCTGACTCTAGCCATGATTCTATGAGCACCAATTCCCATACCCTACCCAATAATTGACCTCAACCTAGGAATCCTATTTATCCTTGCCCTGTCAAGCCTTGCAGTATACTCAATCCTGGGCTCGGGCTGAGCATCAAATTCAAAATACGCACTAATTGGGGCCCTCCGAGCCGTGGCCCAAACAATTTCCTATGAAGTAAGCTTAGGACTAATCCTTCTCTCTGTAATTATATTTTCGGGAGGTTACACCCTGCAAACATTCAATACCACCCAAGAAAGCATTTGACTACTCATCCCTGCCTGACCCCTGGCCGCAATATGATATATCTCAACACTAGCTGAAACAAACCGGGCACCCTTCGACCTAACAGAAGGAGAGTCAGAACTAGTATCTGGTTTTAATGTAGAGTATGCAGGAGGTCCATTTGCACTTTTTTTCCTAGCTGAATACGCTAACATCCTTTTAATAAATACCCTATCAGCCGTACTATTTCTAGGAGCCTCACACATCCCCAGCATCCCCGAACTTACAACAATTAATCTTATGATTAAAGCTGCACTACTATCAGCTGTATTCCTATGGGTACGAGCCTCATATCCCCGATTCCGATACGATCAACTTATACACCTGGTCTGAAAAAACTTTCTTCCTCTCACACTCGCCTTCGTACTATGACACACCGCCCTACCAATTGCCCTAGCAGGACTCCCCCCACAACTA

>A._kreyenbergii_2

ATGCTAAACATCCTAATCACCCACTTAATTAACCCACTGGCCTACATCGTACCAGTGCTGCTAGCAGTGGCCTTCCTTACACTGGTTGAACGAAAAGTACTAGGCTATATGCAGCTACGAAAAGGGCCAAATGTAGTAGGACCTTATGGACTCCTTCAGCCCATTGTTGATGGGGTAAAACTATTTACCAAAGAGCCCGTACGCCCATCCACAGCATCCCCGCTCCTATTCCTGGCCGCCCCCGTGCTCGCACTGACTCTAGCCATAATTCTATGAGCACCTATTCCCATACCCTACCCGATAATTGACCTCAACCTAGGAATCCTATTTATCCTTGCCCTATCAAGCCTTGCAGTATACTCAATCCTGGGGTCGGGCTGAGCATCAAATTCAAAATACGCATTAATTGGGGCCCTACGAGCCGTGGCCCAAACAATTTCTTATGAAGTAAGCCTAGGACTAATCCTTCTCTCTGTAATTATCTTTTCGGGAGGATATACCCTGCAAACATTCAATACTACCCAAGAAAGCATTTGACTACTCATCCCTGCCTGACCCCTAGCCGCAATATGATACATCTCAACACTAGCTGAAACAAACCGAGCACCCTTCGACCTAACAGAAGGGGAGTCAGAACTAGTATCTGGTTTCAACGTAGAATATGCAGGAGGGCCCTTTGCACTTTTCTTCCTGGCTGAATACGCTAACATCCTTTTAATAAATACCCTGTCAGCCGTACTATTTCTAGGAGCCTCACACATCCCCAGCATCCCCGAACTTACAACAATTAATCTTATGGTCAAGGCTGCACTACTATCAGCTGTATTTCTATGAGTACGAGCCTCATACCCCCGATTCCGATACGATCAACTTATACACCTGGTCTGAAAAAACTTTCTTCCTCTCACACTCGCCTTCGTACTATGACACACCGCCCTACCAATCGCCCTAGCAGGACTCCCCCCACAACTA

>A._longipinnis

ATGCTAAACATCCTAATTACCCACTTAATTAACCCCTTAGCCTACATCGTACCAGTACTCCTAGCGGTAGCCTTCTTAACACTAATTGAACGAAAAGTACTAGGATACATGCAACTGCGAAAGGGACCAAACGTGGTAGGACCCTACGGACTTCTACAACCCATTGCTGACGGGGTAAAGCTCTTCATTAAAGAACCCGTCCGCCCATCCACATCATCCCCGTTCCTATTTTTAGCCACCCCCATGCTCGCACTAACTCTGGCCATAATCCTATGAGCACCAATCCCTATACCCCACCCAATAATTGACCTCAACCTAGGAATCCTATTTATCCTGGCCCTATCAAGCCTTGCAGTCTACTCAATCCTTGGATCAGGCTGAGCATCAAACTCAAAATACGCCCCAATGGGGGCCTTAGGGGCTGTGGCCCAAACAATTTCCTATGAAGTAAGCTTAGGACTAATCCTCCTCTCCGTGATCATCTTCTCAGGAGGGTACACCCTACAAACATTCAACACCACTCAAGAAAGCATTTGACTACTCATTCCTGCCTGACCCCTAGCCGCAATATGATATATCTCAACACTGGCCGAAACTAACCGAGCACCCTTCGACCTAACAGAGGGAGAGTCAGAACTAGTATCCGGCTTCAACGTAGAATATGCAGGAGGACCATTTGCACTCTTCTTCCTGGCCGAATATGCTAACATCCTTCTAATAAATACCCTCTCAGCCGTACTATTCCTAGGAGCCTCACACATCCCTAACATCCCTGAACTCACAACAATTAATCTCATAACTAAAGCTGCCCTATTATCCGCCGTATTCCTATGAGTACGAGCCTCCTACCCACGATTTCGATACGACCAACTAATACACCTAGTATGAAAAAACTTCCTTCCCCTCACACTCGCCTTTGTGTTATGACACGCCGCCCTGCCAATCGCCCTAGCAGGACTCCCCCCACAACTA

>A._monticola_1

ATGCTAAACACCTTAATTACTCACCTAATTAACCCCTTGGCTTACATCGTACCTGTACTCCTAGCAGTAGTCTTCCTAACATTAATTGAACGAAAAGTACTAGGATATATACAACTACGGAAAGGACCAAATGTGGTAGGACCCTACGGACTTCTACAACCCATTGCTGATGGAGTAAAACTCTTCACCAAAGAACCCGTCCGCCCATCCACATCATCCCCATTCCTATTTTTAGCCGCCCCAATACTTGCACTAACCCTAGCCATGATCCTATGAACACCAATACCCATACCCCACCCAATAATTGACCTCAACCTAGGAATTCTATTTATTCTGGCCCTATCAAGCCTTGCAGTATATTCAATTCTTGGATCAGGCTGAGCATCAAATTCAAAGTACGCACTAATTGGGGCCCTACGAGCTGTAGCCCAAACAATTTCCTACGAAGTCAGCCTAGGGCTAATCCTCCTTTCCGTAATTATTTTTTCAGGAGGCTACACCCTACAAACGTTCAACACCACCCAAGAAAGCATCTGACTACTCATCCCCGCCTGACCTTTAGCCGCAATATGATACATTTCAACACTAGCCGAAACAAACCGAGCACCATTCGACCTGACAGAAGGAGAATCTGAACTAGTATCTGGTTTCAACGTAGAATATGCAGGAGGACCCTTCGCACTCTTTTTCCTGGCCGAGTACGCCAACATCCTTTTAATAAATACCCTCTCAGCCGTACTTTTCCTAGGGGCCTCACACATCCCTAGCATCCCCGAACTCACAACAATCAACTTAATGACTAAAGCTGCACTCCTGTCCACTGTTTTCCTCTGGGTACGAGCCTCATACCCGCGATTCCGGTACGACCAACTCATGCACCTAGTATGAAAAAACTTCCTTCCCCTCACACTCGCCTTCGTACTCTGACACACCGCCCTACCAATCGCCCTGGCAGGACTCCCCCCACAACTA

>A._monticola_2

ATGCTAAACACCTTAATTACTCACCTAATTAACCCCTTGGCTTACATCGTACCTGTACTCCTAGCAGTAGTCTTCCTAACATTAATTGAACGAAAAGTACTAGGATATATACAACTACGGAAAGGACCAAATGTGGTAGGACCCTACGGACTTCTACAACCCATTGCTGATGGAGTAAAACTCTTCACCAAAGAACCCGTCCGCCCATCCACATCATCCCCATTCCTATTTTTAGCCGCCCCAATACTTGCACTAACCCTAGCCATGATCCTATGAACACCAATACCCATACCCCACCCAATAATTGACCTCAACCTAGGAATTCTATTTATTCTGGCCCTATCAAGCCTTGCAGTATATTCAATTCTTGGATCAGGCTGAGCATCAAATTCAAAGTACGCACTAATTGGGGCCCTACGAGCTGTAGCCCAAACAATTTCCTACGAAGTCAGCCTAGGGCTAATCCTCCTTTCCGTAATTATTTTTTCAGGAGGCTACACCCTACAAACGTTCAACACCACCCAAGAAAGCATCTGACTACTCATCCCCGCCTGACCTTTAGCCGCAATATGATACATTTCAACACTAGCCGAAACAAACCGAGCACCATTCGACCTGACAGAAGGAGAATCTGAACTAGTATCTGGTTTCAACGTAGAATATGCAGGAGGACCCTTCGCACTCTTTTTCCTGGCCGAGTACGCCAACATCCTTTTAATAAATACCCTCTCAGCCGTACTTTTCCTAGGGGCCTCACACATCCCTAGCATCCCCGAACTCACAACAATCAACTTAATGACTAAAGCTGCACTCCTGTCCACTGTTTTCCTCTGGGTACGAGCCTCATACCCGCGATTCCGGTACGACCAACTCATGCACCTAGTATGAAAAAACTTCCTTCCCCTCACACTCGCCTTCGTACTCTGACACACCGCCCTACCAATCGCCCTGGCAGGACTCCCCCCACAACTA

>A._paradoxus_1

ATGCTAAACATCCTAATTACCCACCTAATTAACCCATTAGCCTACATCGTACCAGTACTTCTAGCAGTGGCCTTCCTAACACTAATCGAACGAAAAGTTCTAGGCTACATACAACTACGAAAAGGGCCAAATGTAGTAGGACCCTATGGACTTCTTCAACCTATCGTTGACGGAGTAAAACTATTCGTTAAAGAGCCCATCCGCCCATCCACAGCATCCCCATTTTTATTTTTAGCCGCCCCCGTGCTCGCGTTAACCCTAGCTATAATCCTATGAGCACCAATTCCTATGCCCTACCCAATGACTGACCTCAACCTAGGAATTCTATTTATCCTTGCCCTATCAAGCCTTGCAGTATACTCAATTTTAGGGTCAGGCTGAGCATCAAATTCAAAATACGCACTAATTGGTGCCCTACGGGCCGTGGCCCAAACAATTTCCTATGAGGTAAGCCTAGGACTAATTCTTCTCTCCGTAATTATCTTTTCAGGAGGATACACCCTGCAAACATTCAACACCACTCAAGAAAGCATTTGACTACTTATCCCTGCCTGACCCCTGGCCGCAATATGGTATATCTCAACACTAGCTGAAACAAACCGAGCACCATTCGACCTAACAGAAGGAGAATCAGAGCTAGTATCTGGTTTCAACGTGGAATATGCAGGAGGACCCTTCGCACTCTTCTTCTTAGCCGAGTACGCCAACATCCTTCTAATAAATACCCTCTCAGCCGTATTATTCCTAGGAGCCTCGCACATCCCCAACATCCCCGAACTTACAACAATTAACCTTATAATTAAAGCTGCACTACTATCAGCCGTCTTCCTATGAGTACGGGCCTCATATCCCCGATTTCGATACGACCAACTAATGCATCTAGTATGGAAAAACTTCCTTCCCCTCACACTCGCCTTTGTACTGTGACACACCGCCCTACCGATTGCCCTGGCAGGACTCCCCCCACAACTG

>A._paradoxus_2

ATGCTAAACATCCTAATCACCCACTTAATTAACCCATTAGCCTACATCGTACCAGTATTGCTAGCAGTGGCCTTCCTGACACTAGTCGAACGAAAAGTACTAGGCTATATGCAACTACGAAAAGGGCCAAATGTAGTAGGACCTTACGGACTCCTTCAACCCATTGTCGACGGGGTAAAACTATTTACCAAGGAACCCATCCGCCCATCCACAGCATCCCCACTTTTGTTCTTAGCTGCCCCCGTGTTCGCACTAACTCTAGCCATGATCTTATGAGCACCCATTCCCATGCCCTATCCAATAATTGACCTCAACCTAGGAATCCTATTTATCCTTGCCCTATCAAGCCTTGCGGTATACTCAATCTTAGGGTCGGGCTGAGCATCAAATTCAAAATACGCACTAATTGGAGCCCTACGGGCCGTGGCCCAAACAATTTCCTATGAAGTAAGCTTAGGATTAATCCTTCTCTCTGTAATTATCTTTTCAGGAGGGTACACCCTACAAACATTTAACACCACCCAGGAAAACATTTGACTACTCATCCCTACCTGACCCCTAGCCGCAATATGATATATCTCAACACTAGCTGAAACAAACCGGGCACCCTTCGACCTAACAGAGGGGGAATCAGAGCTAGTATCTGGTTTCAACGTAGAATATGCAGGGGGACCCTTTGCACTCTTCTTCTTGGCTGAGTACGCCAACATCCTTTTAATAAATACCCTATCAGCCGTGCTATTTCTAGGAGCCTCACACATCCCCAGCGTCCCCGAACTTACAACAATTAACCTCATGATTAAAGCTGCACTGCTATCAGCTGTATTCCTATGAGTACGAGCCTCATACCCTCGATTCCGATATGATCAACTCATACACCTAGTCTGAAAGAACTTTCTTCCCCTCACACTCGCCTTCGTACTATGACACACCGCCCTGCCAATTGCCCTAGCAGGACTCCCCCCACAGCTA

>A._parallens_1

ATGCTAAACATCCTTATCACCCACTTAATCAACCCCCTAGCCTACATCGTACCAGTACTCCTGGCAGTGGCCTTCCTAACACTAGTTGAACGAAAAGTACTAGGCTATATACAACTACGAAAAGGACCAAACGTGGTGGGACCTTACGGACTCCTTCAACCCATTCTAGACGGGGTAAAACTATTTACCAAAGAACCCGTCCGCCCATCTACAGCATCCCCACTTTTATTTTTGGCCGCCCCTGTGCTCGCACTGACTCTGGCCATAATCCTATGAGCACCAATTCCCATGCCCTACCCAATAATTGACCTTAACCTAGGAATCCTGTTCATCCTTGCCCTATCAAGCCTTGCAGTATACTCAATCCTAGGATCAGGCTGGGCATCAAATTCAAAATATGCACTAATCGGAGCCTTGCGGGCTGTAGCCCAAACAATTTCTTATGAGGTAAGCCTAGGACTAATCCTTCTCTCTGTAATTATCTTTTCAGGGGGATACACCCTACAAACATTTAATACCACCCAAGAAAGCGTTTGACTACTCATCCCTGCCTGACCCCTAGCTGCAATATGATATATCTCAACACTAGCTGAAACAAACCGGGCACCCTTCGACCTAACAGAGGGAGAATCAGAACTAGTATCTGGTTTCAATGTAGAATATGCAGGAGGACCCTTTGCACTCTTCTTCTTGGCTGAATACGCCAACATTCTTTTAATAAATACCCTATCAGCCGTACTATTTCTAGGGGCCTCGCACATCCCCAGCATCCCCGAACTTACAACAATTAACCTTATAATTAAAGCTGCACTACTATCAGCTGTATTCCTATGAGTGCGAGCCTCATACCCCCGATTCCGATATGACCAACTCATACACCTAGTCTGAAAAAACTTTCTTCCCCTCACACTCGCCTTCGTACTTTGGCACACCGCCCTACCGATCGCCCTAGCAGGGCTCCCCCCACAAGTA

>A._parallens_2

ATGCTAAACATCCTTATCACCCACTTAATCAACCCCTTAGCCTACATTGTACCAGTACTCCTAGCAGTGGCCTTCCTAACACTAGTTGAACGAAAAGTACTAGGCTATATACAACTACGAAAAGGACCAAACGTGGTGGGACCTTACGGACTCCTTCAACCCATTATAGACGGAGTAAAACTATTTACCAAAGAACCTGTCCGCCCATCTACAGCATCCCCACTTTTATTTTTGGCCGCCCCAGTGCTCGCACTGACTCTGGCCATAATCCTATGAGCACCAATTCCCATGCCCTACCCAATGATTGACCTTAACCTAGGGATCCTGTTCATCCTTGCCCTGTCAAGCCTTGCAGTATACTCAATCCTGGGATCAGGCTGAGCATCAAATTCAAAATACGCACTAATCGGAGCCCTACGGGCCGTGGCCCAAACAATTTCCTATGAGGTAAGCTTGGGACTAATCCTTCTCTCTGTAATTATCTTTTCAGGAGGGTACACCCTACAAACATTTAACACCACCCAAGAAAGCGTTTGACTACTCATCCCTGCCTGACCCCTAGCTGCAATATGATATATCTCAACACTAGCTGAAACAAACCGGGCACCCTTCGACCTAACAGAGGGAGAATCAGAACTAGTATCTGGTTTCAACGTAGAATATGCAGGAGGACCCTTCGCACTCTTCTTCTTGGCTGAGTACGCCAACATTCTTTTAATAAATACCCTATCAGCCGTACTATTTCTAGGAGCCTCCCACATCCCCAGCATCCCCGAACTTACAACAATTAACCTTATAATTAAAGCTGCACTACTATCAGCTGTATTCCTATGAGTACGAGCCTCATACCCCCGATTCCGATACGACCAACTCATGCACCTAGTCTGAAAAAACTTTCTTCCCCTCACACTCGCCTTCGTACTTTGACACACCGCCCTGCCAATTGCCCTAGCAGGGCTCCCCCCACAACTA

>A._parallens_3

ATGCTAAACATCCTTATCACCCACTTAATCAACCCCTTAGCCTACATCGTACCAGTGCTCCTGGCAGTGGCCTTCCTAACACTAGTTGAACGAAAAGTACTAGGCTATATACAACTACGAAAAGGACCAAACGTAGTGGGACCTTACGGACTCCTTCAACCCATTGTAGACGGGGTAAAACTATTTACCAAAGAGCCCGTCCGCCCATCTACAGCATCCCCACTTTTATTTTTGGCCGCCCCAGTGCTCGCACTGACTCTGGCCATAATCCTGTGAGCACCAATTCCTATGCCCTACCCAATAATTGACCTTAACCTAGGAATCCTGTTTATCCTTGCCCTATCGAGCCTTGCAGTATACTCAATCCTGGGATCAGGCTGAGCATCAAATTCAAAATACGCACTAATCGGAGCCTTGCGGGCTGTGGCCCAAACAATTTCTTATGAAGTAAGCCTGGGACTAATCCTTCTCTCTGTAATTATCTTTTCAGGAGGGTACACCCTACAAACATTTAATACCACCCAAGAAAGCGTTTGACTACTCATCCCTGCCTGACCCCTAGCTGCAATATGGTATATCTCAACACTAGCTGAAACAAACCGGGCACCCTTCGACCTAACAGAGGGGGAATCAGAACTAATATCTGGTTTCAACGTAGAATATGCAGGAGGACCCTTCGCACTCTTCTTCTTGGCTGAGTACGCCAACATTCTTTTAATAAATACCCTATCAGCCGTACTATTTCTAGGGGCCCCGCACATCCCCAGCATCCCAGAACTTACAACAATTAACCTTATAATTAAAGCTGCACTACTATCAGCTGTGTTCCTATGAGTGCGAGCCTCATACCCCCGATTCCGATATGACCAACTCATACACCTAGTCTGAAAAAACTTTCTTCCCCTCACACTCGCCTTCGTACTTTGACACACCGCCCTGCCAATTGCCCTAGCAGGGCTCCCCCCACAAGTA

>A._parallens_4

ATGCTAAACATCCTTATCACCCACTTAATCAACCCCTTAGCCTACATCGTACCAGTACTCCTGGCAGTGGCCTTCCTAACACTAGTTGAACGAAAAGTACTAGGCTATATACAACTACGAAAAGGACCAAACGTAGTGGGACCTTACGGACTCCTTCAACCCATTGCAGACGGGGTAAAACTATTTACCAAAGAGCCCGTCCGCCCATCTACAGCATCCCCACTTTTATTTTTGGCCGCCCCAGTGCTCGCACTGACTCTGGCCATGATCCTATGAGCACCAATTCCTATGCCCTACCCAATAATTGACCTTAACCTAGGAATCCTGTTTATCCTTGCCCTATCGAGCCTTGCAGTATACTCAATCCTGGGATCAGGCTGAGCATCAAATTCAAAATACGCACTAATCGGAGCCTTGCGGGCTGTAGCCCAAACAATTTCTTATGAAGTAAGCCTAGGACTAATCCTTCTCTCTGTAATTATCTTTTCAGGAGGGTACACCCTACAAACATTTAATACCACCCAAGAAAGCGTTTGACTACTCATCCCTGCCTGACCCCTAGCTGCAATATGGTATATCTCAACACTAGCTGAAACAAACCGGGCACCCTTCGACCTAACAGAGGGGGAATCAGAACTAGTATCTGGTTTCAACGTAGAATATGCAGGAGGACCCTTCGCACTCTTCTTCTTGGCTGAGTACGCCAACATTCTTTTAATAAATACCCTATCAGCCGTACTATTTCTAGGGGCCTCGCACATCCCAAGCATCCCAGAGCTTACAACAATTAACCTTATAATTAAAGCTGCACTACTATCAGCTGTGTTCCTATGAGTGCGAGCCTCATACCCCCGATTCCGATATGACCAACTTATGCACCTAGTCTGAAAAAACTTTCTTCCCCTCACACTCGCCTTCGTACTTTGACACACCGCCCTACCAATTGCCCTAGCAGGGCTCCCCCCACAACTA

>A._spinifer

ATGCTAAACATCCTAATCACCCACTTAATCAACCCATTAGCCTACATCGTACCAGTACTTCTAGCAGTAGCCTTCCTAACACTAATTGAACGAAAAGTACTAGGTTATATACAACTGCGAAAGGGGCCAAATGTGGTAGGACCCTACGGACTCCTTCAACCCATCGCTGATGGAGTAAAACTATTTATCAAAGAACCCATCCGCCCATCCACAGCATCCCCCCTCCTATTTTTAGCTGCCCCCATGCTCGCACTGACCCTGGCCATAATCTTATGAGCACCAATTCCTATGCCCTACCCAGTAATTGACCTCAACCTAGGAATTCTATTTATCCTTGCCCTATCAAGCCTTACAGTATACTCAATCTTGGGGTCAGGCTGAGCATCAAACTCAAAATACGCACTAATCGGTGCCCTACGAGCCGTGGCCCAAACAATTTCTTATGAAGTAAGCCTAGGACTAATTCTTCTCTCCGTAATTATCTTTTCGGGAGGGTATACCCTACAGACATTCAACACCACCCAAGAAAGCATTTGACTGCTCATCCCTGCCTGGCCCCTAGCCGCAATATGATATATTTCAACACTAGCTGAAACAAACCGAGCACCATTTGACCTAACAGAAGGAGAATCAGAACTAGTGTCTGGTTTCAACGTAGAATATGCAGGAGGACCCTTTGTACTCTTCTTCCTAGCCGAATACGCCAACATCCTTTTAATAAATACCCTCTCAGCCGTACTATTCCTAGGAGCCTCACACATCCCCAACATCCCCGAACTTACAACAATCAACCTCATAATCAAGGCTGCACTGCTATCAGCCGTTTTCCTATGAGTACGAGCCTCGTACCCCCGATTCCGATACGACCAACTAATACACCTAGTATGAAAAAACTTCCTTCCCCTCACACTCGCCTTCGTCTTATGACACACCGCCCTACCAATTGCCCTGGCAGGACTCCCCCCACAACTA

>A._stenotaeniatus

ATGCTAAACATCCTAATTACCCATTTAATCAACCCATTAGCCTACATCGTACCAGTGCTTCTAGCAGTAGCCTTCCTAACACTAATCGAACGAAAAGTACTAGGTTATATACAACTGCGAAAGGGACCAAATGTGGTAGGACCCTACGGACTCCTTCAACCCATCGCTGATGGAGTAAAACTGTTTATCAAAGAACCCATCCGCCCATCCACAGCATCCCCCCTCCTATTTTTAGCCGCCCCAATGCTCGCACTGACCCTGGCCATGATCTTATGAGCACCAATTCCTATGCCCTACCCAGTAATTGACCTCAACCTAGGAATTCTATTTATCCTTGCCCTATCAAGCCTTACAGTATACTCAATCTTAGGGTCAGGCTGAGCATCAAACTCAAAATACGCACTAATCGGTGCCCTACGAGCCGTGGCCCAAACAATTTCTTATGAAGTAAGCCTAGGACTAATTCTTCTCTCCGTAATTATCTTTTCAGGAGGGTATACCCTACAGACATTCAACACCACCCAAGAAAGCATTTGACTGCTCATCCCTGCCTGACCCCTAGCCGCAATATGATATATTTCAACACTAGCTGAAACAAACCGAGCACCATTTGACCTAACAGAAGGAGAATCAGAACTAGTGTCTGGTTTCAACGTAGAATATGCAGGAGGACCCTTTGCACTCTTCTTCCTAGCCGAGTACGCCAACATCCTTTTAATAAATACCCTCTCAGCCGTACTATTCCTAGGAGCCTCACACATCCCCAACATCCCCGAACTTACAACAATCAACCTCATAATCAAGGCTGCACTGCTATCAGCCGTTTTCCTATGAGTACGAGCCTCATACCCCCGATTCCGATACGACCAACTAATACACCTAGTGTGAAAAAACTTCCTTCCCCTCACACTCGCCTTCGTCTTATGACACACCGCCCTACCAATTGCCCTGGCAGGACTCCCCCCACAACTA

>A._wenchowensis_1

ATGCTAAACATCCTAATCACCCACTTAATTAATCCACTAGCCTACATCGTACCAGTACTGCTAGCAGTGGCCTTTCTAACACTAGTTGAACGAAAAGTACTAGGCTATATGCAGCTACGAAAGGGACCAAATGTAGTAGGACCTTATGGACTCCTTCAACCCATTGTTGACGGAGTAAAACTATTTACCAAAGAACCCGTCCGCCCATCCACAGCATCCCCGCTTTTATTCCTAGCCGCCCCCGTGCTCGCACTGACTCTAGCCATAATCCTTTGAGCACCAATCCCCATACCCTACCCAATAACTGACCTCAACCTAGGAATCCTATTTATCCTTGCCCTGTCAAGCCTTGCAGTATACTCAATTCTAGGATCAGGCTGAGCATCAAATTCAAAATACGCGCTAATCGGGGCCCTGCGAGCCGTCGCCCAAACAATTTCTTATGAGGTAAGCTTAGGACTAATCCTTCTCTCTGTAATTATCTTCTCAGGGGGATACACCCTACAAACATTCAACACCACCCAAGAAAGCATTTGACTACTCATCCCTGCCTGACCCCTAGCCGCAATATGATACATCTCAACACTAGCCGAGACAAACCGGGCACCCTTCGACTTAACAGAAGGAGAATCAGAACTAGTATCTGGTTTTAACGTAGAATATGCAGGGGGACCCTTTGCGCTTTTCTTCCTGGCTGAATACGCCAACATCCTTTTAATAAATACCCTATCAGCCGTACTATTTCTGGGAGCCTCACACATCCCCAGCATCCCCGAACTTACAACGATTAATCTCATGATTAAGGCTGCACTACTGTCAGCTGTATTCCTATGAGTACGAGCCTCATACCCCCGATTCCGATACGACCAACTTATACACCTGGTCTGAAAAAACTTTCTTCCTCTTACACTCGCCTTCGTACTATGACACACCGCCTTGCCAATTGCCCTGGCAGGACTCCCCCCACAACTA

>A._wenchowensis_2

ATGCTAAACATCCTAATCACCCACTTAATTAACCCACTGGCCTACATCGTACCAGTACTGCTAGCAGTGGCCTTCCTAACACTAGTTGAACGAAAAGTATTAGGCTATATACAACTACGAAAAGGGCCAAATGTAGTAGGACCTTATGGTCTCCTTCAACCCATTGTTGACGGAGTAAAACTATTTACCAAAGAACCCGTCCGCCCATCCACAGCATCCCCACTCTTATTCCTAGCCGCCCCCGTGCTTGCACTGACCCTAGCCATAATCCTATGGGCACCAATTCCCATACCCTACCCAATAATTGACCTTAACCTGGGAATCCTATTTATCCTTGCCCTCTCAAGCCTTGCAGTATACTCAATTCTAGGATCGGGCTGAGCATCAAATTCAAAGTACGCGCTAATCGGGGCCCTACGAGCCGTGGCCCAAACAATTTCTTATGAAGTAAGCTTAGGACTAATCCTTCTCTCTGTAATTATCTTTTCAGGAGGGTACACCCTTCAAACATTCAATACCACCCAAGAAAGCATTTGACTACTCATTCCTGCATGACCCCTAGCCGCGATATGGTACATCTCAACACTAGCTGAAACAAACCGGGCACCATTTGACTTAACAGAGGGAGAATCAGAACTAGTATCTGGTTTTAACGTAGAATATGCAGGGGGGCCCTTTGCGCTTTTCTTCCTGGCCGAGTACGCCAACATCCTTTTAATAAATACCCTATCAGCCGTACTATTTCTGGGAGCCTCACACATCCCCAGCATCCCTGAACTTACAACAGTTAATCTCATGATTAAAGCTGCACTACTATCAGCTGTATTCCTATGAGTGCGAGCCTCGTATCCCCGATTCCGGTACGATCAACTTATGCACCTAGTCTGAAAAAACTTTCTTCCTCTTACACTCGCCTTCGTACTGTGACACACCGCCCTGCCAATCGCCCTGGCAGGACTCCCCCCACAACTA

>A._wuyiensis

ATGCTAAACATCCTAATTACCCACCTAATTAACCCATTAGCCTACATCGTACCAGTACTTCTAGCAGTGGCCTTCCTAACACTAGTCGAACGAAAAGTACTAGGTTATATACAACTACGAAAGGGGCCAAATGTAGTAGGACCCTATGGACTCCTTCAACCCATCCTTGACGGAGTAAAACTATTCACTAAAGAACCCATCCGCCCATCCACAGCATCCCCATTTTTATTTTTAGCCGCCCCCATCCTCGCACTAACCCTAGCTATAATCCTATGAGCACCAATTCCTATGCCCTACCCAATAACTGACCTCAACCTAGGAATTCTATTCATCCTTGCCCTATCAAGCCTTGCAGTATACTCAATTTTAGGGTCAGGCTGAGCATCAAATTCAAAATACGCACTAATTGGTGCCCTACGGGCCGTGGCCCAAACAATTTCCTATGAGGTAAGCCTAGGACTAATCCTTCTCTCCGTAATTATCTTTTCAGGAGGATACACCCTGCAAACATTCAACACCACTCAAGAAAGCATTTGACTACTTATCCCTGCCTGACCCCTGGCCGCAATATGATATATCTCAACACTAGCTGAAACAAACCGAGCACCATTCGACCTAACAGAAGGAGAATCAGAACTAGTATCTGGTTTCAACGTAGAATATGCAGGAGGACCCTTCGCACTCTTCTTCTTAGCCGAGTACGCCAACATCCTTCTAATAAATACCCTCTCAGCCGTACTATTCCTAGGAGCCTCACACATCCCCAACATCCCCGAACTTACAACAATTAACCTCATAATTAAAGCTGCACTACTATCAGCCGTCTTCCTATGAGTACGAGCCTCATATCCCCGATTTCGATATGACCAACTAATGCACCTGGTATGAAAAAACTTCCTTCCCCTCACACTCGCCTTTGTACTATGACACACCGCCCTACCGATTGCCCTGGCAGGACTCCCCCCACAACTG

>A._yunnanensis_1

ATGCTAAACACCTTAACCACCCACTTAATTAACCCCTTGGCCTACATCGTGCCAGTACTACTAGCAGTAGCCTTCCTAACACTAATTGAACGAAAAGTACTAGGGTATATACAACTACGAAAAGGACCAAATGTGGTGGGACCCTATGGACTCCTACAACCCATTGTCGACGGAGTAAAACTCTTTATTAAAGAGCCCGTACGCCCATCCACATCCTCCCCATTCCTATTTTTAGCCGCCCCAATACTTGCATTAACCCTAGCCATGATCCTATGAACACCAATACCTATACCCCACCCAATAGTTGACCTCAACCTAGGAATCCTGTTTATTCTGGCCCTATCAAGCCTCGCAGTATACTCAATCCTCGGGTCAGGCTGAGCATCGAATTCAAAATATGCACTAATTGGAGCCTTACGGGCCGTGGCTCAAACAATTTCCTACGAAGTCAGCCTCGGACTAATCCTCCTCTCTGTAATTATCTTTTCAGGGGGCTACACCCTGCAAACATTCAACACCACCCAAGAAAGCATTTGACTGCTAATCCCCGCCTGACCTTTAGCCGCAATATGATACATTTCAACACTGGCCGAAACAAACCGAGCACCATTTGACCTAACAGAAGGAGAATCCGAACTAGTATCTGGTTTTAACGTAGAATATGCAGGAGGGCCCTTCGCACTCTTCTTCCTAGCCGAGTACGCCAACATCCTTCTAATAAATACCCTTTCAGCCGTACTATTCCTAGGAGCCTCACACATCCCCAGCATCCCCGAACTCACAACAATAAACCTAATAACTAAAGCTGCACTCCTATCTGCCGTATTCCTCTGAGTGCGAGCCTCATACCCACGATTCCGATACGACCAACTTATACACCTAGTGTGAAAAAACTTCCTTCCCCTTACACTCGCCTTCGTACTATGACACGCCGCCCTACCAATCGCCCTAGCAGGGCTCCCCCCACAACTA

>A._yunnanensis_2

ATGCTAAACACCTTAACTACCCACCTAATTAACCCCTTGGCCTACATCGTGCCAGTACTACTAGCAGTAGCCTTCCTAACACTAATTGAACGAAAAGTACTAGGGTATATACAACTACGAAAAGGACCAAATGTGGTGGGACCCTATGGACTCCTACAACCCATTGTCGACGGAGTAAAACTCTTTATTAAAGAGCCCGTACGCCCATCCACATCCTCCCCATTTCTATTCTTAGCCGCCCCAATACTTGCATTAACCCTAGCCATAATCCTATGAACACCAATACCTATACCCCACCCAATAGTTGACCTCAACCTAGGAATCCTATTTATTCTGGCCCTATCAAGCCTCGCAGTATACTCGATCCTCGGATCAGGCTGAGCATCAAATTCAAAATATGCACTAATTGGGGCCTTACGAGCTGTGGCTCAAACAATTTCTTACGAAGTCAGCCTCGGACTAATCCTCCTCTCTGTAATTATCTTTTCAGGGGGCTACACCCTGCAAACATTCAACACCACCCAAGAAAGCATTTGACTGCTCATCCCTGCCTGACCTTTAGCCGCAATATGATACATTTCAACACTAGCCGAAACAAACCGAGCACCATTTGACCTAACAGAAGGAGAATCCGAGCTAGTATCTGGTTTCAATGTAGAATATGCAGGAGGACCCTTCGCACTCTTATTCCTAGCCGAGTACGCCAACATCCTTCTAATAAATACCCTTTCAGCCGTACTATTCCTAGGAGCCTCACACATCCCCAGCATCCCCGAACTAACAACAATCAACCTAATAACTAAAGCTGCACTCCTATCTGCCGTATTCCTCTGAGTGCGAGCCTCATACCCACGATTCCGATACGACCAACTTATACACCTAGTGTGAAAAAACTTCCTCCCCCTTACACTCGCCTTCGTACTATGACACGCCGCCCTACCAATCGCCCTAGCAGGGCTCCCCCCACAACTA

>Onychostoma_barbatulum

ATGCTAGACACCCTAATCACCCACCTAATCAATCCTTTAGCCTATATTGTACCAGTCCTCTTAGCAGTGGTCTTCCTTACACTAATTGAACGAAAAGTGCTAGGATATATACAACTACGAAAAGGACCAAATGTGGTAGGACCCTATGGACTCCTTCAACCTATTATTGACGGAGTAAAACTCTTTATCAAAGAACCCGTCCGCCCATCTACAGCATCCCCATTTTTATTTTTAGCCGCCCCCATATTAGCATTAACCCTAGCTATAATCTTATGGGCACCAATTCCCATACCCCACCCGATAGTTGACCTCAACCTAGGAATCCTCTTTATCCTGGCCCTATCAAGCCTAGCTGTATATTCAATTCTAGGATCAGGCTGAGCATCAAATTCAAAATATGCACTAATTGGAGCCCTGCGGGCCGTGGCCCAAACAATCTCCTACGAGGTGAGCCTGGGACTAATTCTCCTGTCCGTAATTATTTTCTCAGGCGGATATACCCTACAAACATTCAACACCACTCAAGAAAGCATTTGACTGCTCATCCCTGCTTGACCCCTAGCCGCAATATGATATATTTCAACACTAGCTGAAACAAACCGAGCGCCATTCGACCTAACGGAAGGAGAATCAGAGCTGGTATCCGGATTCAACGTAGAATATGCAGGGGGACCCTTCGCACTCTTCTTTTTAGCTGAATACGCCAACATCCTTTTAATAAACACCCTCTCAGCCGTACTATTCCTAGGAGCCTCACACATCCCCAGTATTCCAGAACTCACAACAATTAATCTGATAACCAAAGCCGCACTATTATCCACCATATTCCTATGGGTGCGAGCCTCATATCCACGATTCCGATACGACCAGTTAATACATCTGGTATGAAAAAACTTTCTCCCTCTCACACTCGCCTTCGTGCTATGACACGCCGCCCTGCCAATTGCCCTAGCAGGACTTCCTCCACAACTA

>Onychostoma_meridionale

ATGTTAAACACCTTAATTATCCAACTAATCAACCCCTTAGCCTACATCGTACCAGTCCTCCTAGCAGTTGCCTTCCTAACACTACTTGAACGGAAAGTCCTAGGATATATACAACTGCGGAAAGGACCAAATGTGGTGGGACCCTACGGACTTCTACAACCCATTGCCGACGGAGTAAAACTCTTTACTAAAGAACCCGTCCGCCCATCCACATCATCCCCATTTCTATTTTTAACCGCCCCCATGCTCGCACTAGCCCTAGCAATAATACTATGAACACCAATCCCTATACCCTACCCAGTAGTAGACCTAAACCTAGGAATCCTATTCATCCTAGCCCTATCGAGCCTCGCCGTATATTCAATTCTGGGATCAGGCTGAGCATCAAATTCAAAATATGCATTAATCGGAGCCCTGCGGGCCGTGGCCCAAACAATTTCATACGAAGTAAGCCTAGGACTAATCCTCCTCTCCGTAATTATTTTTTCAGGGGGGTACGCTCTACAAACATTCAACACCACCCAGGAAAGCATCTGACTACTTATCCCCGCCTGACCATTAGCCGCAATATGATATATCTCAACACTAGCCGAGACAAACCGAGCACCTTTTGACCTAACAGAGGGAGAATCAGAACTAGTATCCGGCTTCAACGTAGAATACGCGGGGGGACCCTTCGCACTCTTCTTTCTAGCCGAATACGCTAACATTCTCTTAATAAACACCCTCTCAGCTGTACTATTCCTAGGAGCCTCGCACATCCCCAGTATTCCCGAACTCACAACAATTAATATTATAACCAAAGCTGCCCTACTATCCACTATATTTTTGTGGGTACGAGCCTCATACCCCCGATTCCGATACGACCAACTAATGCACCTAGTATGAAAAAATTTCCTCCCCCTCACACTTGCCTTCGTTTTATGACACACCGCCCTACCAATTGCCCTGGCAGGACTCCCCCCACAACTA

>Onychostoma_gerlachi

ATGCTAAACACCTTAATTACCCAACTAATCAACCCTTTAGCCTACATCGTACCCGTCCTCCTAGCAGTTGCCTTCCTAACACTACTCGAACGAAAAGTCCTAGGATATATGCAACTGCGGAAGGGACCAAATGTGGTAGGACCCTACGGACTTCTTCAACCCATCGTCGACGGAGTAAAACTATTTACCAAAGAGCCCGTCCGCCCATCCACATCATCCCCGTTCTTATTCTTGGCCGCCCCCATGCTCGCACTGGCCCTAGCAATAATACTATGAACACCAATCCCTATGCCCCACCCAATAGTAGACCTGAACCTAGGAATTCTATTCATCCTAGCCCTCTCAAGCCTCGCCGTGTATTCAATCCTAGGATCAGGCTGAGCATCAAACTCAAAATATGCACTAATCGGAGCCCTACGGGCCGTAGCCCAAACAATTTCCTACGAAGTAAGCCTGGGGCTAATCCTCCTCTCCGTAATTATTTTTTCAGGAGGATATACCCTACAAACATTCAACACCACCCAGGAAAGCATTTGACTACTTATCCCCGCCTGACCCTTAGCCGCAATATGATATATTTCAACACTAGCCGAGACAAGCCGAGCACCTTTCGACCTAACAGAGGGAGAATCAGAACTAGTATCCGGTTTTAATGTAGAATACGCGGGGGGACCCTTCGCACTCTTATTCCTAGCCGAGTACGCTAACATTCTTTTAATAAATACCCTCTCAGCTGTGCTGTTCCTAGGAGCCTCGCACATCCCCAGCATTCCCGAGCTAACAACAATTAATATCATAACCAAAGCCGCCCTACTATCCACCATATTCCTATGAGTACGAGCCTCATATCCCCGATTTCGATACGACCAACTAATACACCTAGTATGAAAAAACTTCCTCCCCCTCACACTTGCCTTTGTACTATGGCACACCGCCCTGCCAATCGCCCTAGCAGGACTCCCCCCACAACTA

>Spinibarbus_denticulatus

ATGCTAAACACCCTAATTACCCACCTAATTAACCCCCTAGCCTACATCGTGCCGGTGCTCCTAGCAGTAGCCTTTCTAACACTAATTGAACGAAAAGTGTTAGGATATATACAACTGCGAAAAGGACCAAACGTAGTGGGGCCCTACGGGTTACTACAACCTATTGCCGACGGAGTAAAACTCTTCATCAAAGAACCCGTTCGCCCATCTACATCATCCCCATTTTTATTTCTAGCCGCCCCTATTCTTGCACTAACCCTGGCCATGACACTATGGGCACCCATACCCATGCCTTACCCAGTAGTTGACCTCAATCTGGGGGTCCTATTTATCCTAGCCTTATCAAGCCTTGCAGTATATTCCATTCTAGGATCAGGATGAGCATCAAATTCAAAATACGCGCTAATTGGGGCCCTACGAGCAGTAGCCCAAACAATTTCCTATGAAGTAAGCCTCGGACTAATTCTTCTATCTGTAATTATCTTCTCAGGAGGGTATACCCTACAAACATTCAACACAGCCCAAGAAAGTATCTGACTATTAGCCCCCGCTTGACCATTAGCCGCAATATGATATATCTCAACCCTAGCAGAAACAAACCGAGCACCATTTGACTTAACAGAAGGAGAGTCAGAACTAGTCTCTGGCTTCAACGTAGAGTATGCAGGAGGCCCCTTCGCCCTATTCTTCCTAGCCGAATACGCGAACATTTTATTAATAAATACTCTATCGGCCGTACTATTCCTAGGAACATCACATATTCACCACATCCCCGAACTAACAACAATCAGCCTCATGACCAAAGCCGCACTACTATCTATTGTGTTCTTATGAGTACGAGCCTCATATCCACGATTCCGGTATGACCAGCTCATACATCTCGTGTGAAAAAACTTCCTTCCCCTAACACTAGCCCTAGTACTATGACATATTGCCCTACCAATCGCACTAGCAGGCCTTCCCCCACAACTA

>Spinibarbus_hollandi

ATGCTAAACACCTTAATTACTCACCTAATTAACCCCCTAGCCTACATCGTACCAGTACTTCTAGCAGTAGTCTTTCTAACACTGATCGAACGAAAAGTGTTAGGATATATGCAACTACGAAAAGGACCAAACGTAGTGGGACCCTACGGGTTACTACAACCTATTGCCGATGGGGTAAAACTCTTCATCAAAGAACCCGTTCGCCCATCTACATCATCCCCATTTTTATTTCTAGCCACCCCAACACTTGCACTAACCCTAGCTATAACCCTGTGAGCACCAATACCTATACCCCACCCAGTAATTGACCTTAACCTGGGAATCTTATTTATTCTGGCCCTGTCAAGTCTCGCAGTATACTCAATTCTAGGATCCGGCTGAGCATCAAATTCAAAATATGCACTAGTTGGAGCATTGCGGGCTGTAGCCCAAACAATTTCCTATGAAGTTAGCCTAGGACTAATTCTTCTCTCCGTAATTATCTTTTCAGGGGGATATACTTTACAAACATTCAACATTACCCAAGAAAACATTTGATTACTTATCCCCGCCTGACCCTTAGCCGCAATGTGATATATCTCAACACTAGCCGAAACAAACCGAGCACCATTCGACCTAACAGAGGGGGAGTCCGAACTAGTATCTGGCTTTAATGTAGAGTATGCAGGAGGACCCTTCGCACTCTTTTTCCTAGCCGAATACGCCAATATCCTCCTAATAAATACCCTCTCAACCGTACTATTTCTAGGAGCCTCACATATCCCCAACGTCCCCGAACTCGCAACAATTAACCTCATAGTTAAAACTGCGCTCCTATCTATTCTATTTCTATGAGCACGGGCCTCATACCCACGATTCCGGTATGACCAACTGATACACCTGGCATGAAAAAACTTCCTCCCCCTCACACTTGCCTTTGTATTATGACACACTGCCCTACCAATCGCCCTAGCAGGACTCCCCCCACAACTA

>Spinibarbus_sinensis

ATGCTAAACATCCTAATTACCCACCTAATTAATCCCCTAGCCTACATCGTACCAGTACTTTTAGCAGTAGCATTCCTAACACTAATTGAACGAAAAGTGCTAGGGTATATACAACTACGAAAAGGGCCAAACGTAGTAGGACCCTACGGACTACTACAGCCCATCGCCGACGGATTAAAACTCTTCATTAAAGAGCCCGTTCGCCCATCTACATCATCCCCATTTCTATTTCTAGCCACTCCAATACTTGCACTAACCCTAGCCATAACCTTATGAGCACCAATCCCCATACCCCACCCAGTAACAGACCTCAACCTAGGAATTTTATTTATTCTAGCCCTATCAAGCCTCGCAGTATACTCAATTCTGGGATCAGGATGAGCATCAAATTCAAAATATGCATTAATTGGGGCCCTACGAGCCGTAGCCCAAACAATTTCCTACGAGGTCAGCCTAGGGTTAATTCTCCTCTCCATAGTCATTTTTTCAGGAGGGTATACTCTACAAACATTCAACAACACCCAAGAAAGCATCTGACTACTCGTCCCCGCTTGACCCCTGGCCGCAATATGATACATCTCAACACTAGCCGAAACAAACCGAGCACCATTCGACCTAACAGAGGGAGAATCAGAACTAGTATCTGGTTTTAATGTAGAATATGCAGGAGGACCCTTTGCACTCTTCTTCCTGGCCGAGTACGCCAACATCCTTCTAATAAACACCCTCTCGGCCGTGCTGTTCCTGGGAGCCTCACACATCCCCAACATCCCTGAACTCACAACAATCAACCTAATAACCAAAGCTGCATTCCTGTCTATTTTATTCCTATGAGTACGAGCCTCGTACCCACGATTCCGATATGACCAGCTAATGCACCTAGTATGAAAAAACTTCCTCCCCCTCACACTTGCCTTCGTACTATGACACACTGCCCTACCAATCGCCCTAGCAGGACTTCCCCCACAACTA

**ND2**

>A._barbodon

ATGAACCCATACGTACTTGCAATCCTACTATCCAGCCTAGGACTAGGAACTACCCTAACCTTTGCCAGCTCTCACTGACTCCTAGCCTGAATAGGCCTAGAAATTAATACGCTAGCAATTGCCCCACTAATAGCACAACACCATCACCCCCGTGCAGTCGAAGCAACCACAAAATACTTCTTAACCCAAGCCACCGCAGCAGCAATGATCCTATTTGCAAGCACAACAAACGCATGAATAACAGGAGAATGAGACATCAACAACCTATCAAACCCCCTCGCCAGCACAATATTTATAGCCGCCCTAGCACTTAAAATTGGACTTGCACCTATACATTTCTGAATACCAGAAGTTCTACAAGGACTAGACCTGCTAACAGGCCTAGTCTTGTCCACATGACAAAAACTCGCCCCATTTGCATTAATTATTCAAACGGCACAAGATATTGACCCGCTACTACTGACACTACTAGGGATTACATCCACACTAATCGGAGGATGAGGAGGACTAAACCAAACTCAACTACGAAAAATCCTAGCCTATTCCTCAATCGCCCACATAGGGTGAATAATTATCGTAATCCAATACGCCCCCCAACTTACTATACTTGCACTAGGAACATACATTATCATAACCTCCGCGACATTCCTGACCCTAAAAATATCTATAACAACCAAAATTAATACACTCGCAACAACCTGATCAAAAAACCCCATACTTGCATCAACAACTGCCCTAGTCCTACTATCATTAGGTGGCCTCCCTCCACTTACAGGATTCATACCAAAATGAATAATTCTGCAAGAACTAACAAAACAAGACCTACCCATCATCGCCACAACAATAGCCCTCGCCGCACTAATCAGCCTATACTTCTACCTACGACTATGCTACGCAATAACTCTGACTGTCTCCCCCAACACAACCAACGCAACCACCCCCTGACGAACCCAAACAACCCAAACCCCCTTACCACCTGCCCTATTCATCACAGCTACCCTGGGGCTATTACCAATAACCCCCACCATCCTAATACTAACCACC

>A._beijiangensis_1

ATCAGTCCGTTCGTCCTCATGATCCTACTGTCCAGCCTGGGACTAGGAACTACTCTAACTTTTGCCAGCTCCCACTGACTCCTAGCCTGAATGGGCCTAGAAATTAATACACTGGCAATCGCCCCACTAATAGCACAACACCATCATCCCCGTGCAGTTGAAGCGACCACAAAATACTTCTTAACACAAGCCACCGCAGCAGCAATAATCTTATTCGCAAGCACAACAAATGCATGAATAACAGGAGAATGAAGCATCAACAACCTATCAAACCCGCTCGCCTCCACAATATTTACGGCCGCTCTAGCACTCAAAATCGGACTTGCCCCTGTACACTTCTGAATACCAGAAGTCCTACAAGGACTAGACCTGTTAACAGGCCTAATCCTATCCACCTGACAAAAACTTGCCCCATTCGCATTAATTGTCCAAACAGCACACAACATTGACCCGCTACTATTAACACTACTAGGGATTACATCTACGCTAGTGGGAGGGTGAGGAGGACTGAACCAAACCCAACTACGAAAAATCTTAGCCTACTCCTCAATCGCCCACATAGGATGAATAATTATTGTGATCCAATACGCCCCACAACTCACTCTCCTTGCACTAGGAACATATATTATCATGACCTCCGCGACATTCATAACCCTAAAAATATCAATAACAACCAAAATTAATACACTCGCAACAACTTGATCGAAGAGCCCCACACTTGCATCAATAACTGCCCTAATCTTACTATCACTAGGCGGCCTCCCACCACTTACAGGATTTATACCAAAATGGATAATTTTACAAGAACTAACAAAACAAAACCTACCCACCATCGCTACAATAATGGCCCTCACCGCACTGATTAGCCTATATTTCTACCTGCGGCTATGTTACACAATAACACTAACCATCTCCCCCAGTACAAACAACTCAGTCACCCCCTGACGAACCAAAACAACCCAAACCACCCTACCACTCGCCCTACTCATCACAGCCGCCCTGGGACTCCTGCCGATGACCCCCACCATTATAATACTAACCACC

>A._beijiangensis_2

ATCAGTCCGTTCGTCCTCATAATCCTACTGTCCAGCCTAGGACTAGGAACTACTCTAACTTTTGCCAGCTCCCACTGACTCCTAGCCTGAATGGGCCTAGAAATTAATACACTGGCAATTGCCCCACTAATAGCACAACACCATCATCCCCGTGCAATTGAAGCGACCACAAAATACTTCTTAACACAAGCCACCGCAGCAGCAATAATCTTATTCGCAAGCACAACAAATGCATGAATAACAGGAGAATGAAGCATCAACAACCTATCAAACCCGCTCGCCTCCACAATATTTACAGCCGCTCTAGCACTCAAAATCGGACTTGCCCCTGTACACTTCTGAATACCAGAGGTCCTACAAGGACTAGACCTGCTAACAGGCCTAATCCTATCCACCTGACAAAAACTTGCCCCGTTCGCATTAATTGTTCAAACAGCACACAACATTGACCCGCTACTATTAACACTACTAGGAATTACGTCTACGCTAGTGGGAGGGTGAGGAGGACTGAACCAAACCCAACTACGGAAAATCTTAGCTTACTCCTCAATCGCCCACATAGGATGAATAATTATTGTAATCCAATACGCCCCGCAACTCACGCTCCTCGCACTAGGAACATATATTATCATGACCTCCGCGACATTCATAACCCTAAAAATATCAATAACAACCAAGATTAATACACTTGCAACAACTTGATCGAAAAACCCTACACTTGCATCGATGACTGCCCTAGTCTTACTATCGCTAGGCGGCCTCCCACCACTTACAGGATTTATACCAAAATGAATAATTTTACAAGAACTAACAAAACAAAACCTACCCACCATCGCTACAATAATGGCCCTCACCGCACTGATTAGCCTGTACTTCTACCTGCGGCTATGTTACACAATAACACTAACCATCTCCCCCAATACAAACAACTCAATCACCCCCTGACGAACCAAAACAACCCAAACCACCCTACCACTCGCCCTACTCATCACAGCCGCCCTGGGACTCCTGCCGATGACCCCCACCATTATAATACTAACCACC

>A._fasciatus

ATGAACCCATATGTACCTACAGTCCTACTATCCAGCCTGGGGTTAGGGACTACCTTAACCTTTGCCAGCTCACACTGACTTCTAGCCTGAATAGGTCTGGAAATTAATACACTAGCAATTGCCCCCTTAATAGCACAACACCACCACCCCCGTGCAGTTGAAGCAACCACAAAATACTTCTTAACCCAAGCCACCGCGGCAGCAATAATCTTATTCGCAAGCACAACAAACGCATGAATAACAGGAGAATGAAATATCAACAACCTCTCAAACCCTCTCGCTAGCACAATATTTACAGCCGCTCTGGCACTTAAAATCGGACTTGCACCCGTACACTTCTGGATGCCAGAAGTCCTACAAGGATTGGACCTGTTAACAGGGCTAATCTTATCCACCTGACAAAAACTTGCCCCGGTTGCACTAATTATCCAAACAGCACAAAATATCGACCCACTACTACTAACTCTTCTAGGAGTCACATCTACACTGGTGGGGGGATGAGGAGGACTAAACCAAACCCAGCTGCGAAAAATCCTAGCCTACTCCTCAATCGCCCACATAGGGTGAATAATTATTGTGATTCAGTACGCCCCACAACTCACCCTCCTTGCGCTAGGAACATACATTATCATAACCTCAGCAACATTCATAACCTTAAAAATATCAATAACAACCAAAGTCAATACACTTGCAACAACCTGATCGAAAAGCCCCGTGCTCGCATCTACAACCGCCCTGGTCCTACTTTCACTAGGCGGCCTCCCACCACTCACAGGGTTCATACCAAAATGAATAATTCTACAAGAGCTAACAAAACAAGACCTGCCCATCGTCGCCACAACAATAGCCTTAACCGCACTAATTAGCCTATACTTCTATTTACGACTGTGCTACGCAATGACATTAACTATCTCCCCCAACACAACCAACTCAATCACCCCCTGACGAACCAAAACAACCCAAACCACCCTGCCACTCGCCCTATTTATTACAGCCACCCTGGGACTACTACCCGTAACCCCCACCATTATAATGCTATCCACC

>A._hemispinus

ATGAACCCATACGTACTTGCAACCCTGCTATCCAGCCTGGGATTAGGGACCACCCTAACCTTTGCCAGCTCCCACTGACTTCTGGCCTGAATGGGCCTGGAGATTAATACACTAGCAATTGCCCCACTAATAGCACAACACCACCACCCCCGTGCAGTTGAAGCAACCACAAAATACTTCTTAACACAAGCCACCGCAGCAGCAATGATCTTGTTCGCAAGCACAACAAATGCATGAATAACAGGAGAATGAAGCATCAATAACCTGTCAAACCCCCTCGCCAGCACAATATTCACGGCCGCCCTGGCACTCAAAATTGGACTTGCACCCGTACACTTCTGAATACCAGAAGTACTACAAGGATTAGACCTGTTAACAGGCCTAATCTTATCTACCTGACAAAAACTTGCCCCATTCGCACTAATCATCCAAACAGCACAAAATATTGACCCCTTACTACTAACGCTACTAGGAGTCACATCCACACTAGTAGGGGGATGAGGGGGACTAAACCAAACCCAGCTACGAAAAATCCTGGCCTACTCCTCAATCGCCCACATGGGATGAATAATTATTGTGGTTCAGTACGCCCCGCAACTCACCCTTCTTGCACTAGGAACATACATCATCATAACATCCGCAGCATTTATAACCCTAAAAATATCAATAACAACCAAAATTGGAACACTCGCAACAACCTGATCAAAAAGCCCTGCACTCACATCAACAACCGCCCTGGTCTTATTATCATTAGGCGGCCTCCCACCCCTTACAGGATTTATACCAAAATGAATAATCCTACAAGAACTAACAAAACAAGACCTACCCATCATCGCCACAACAATGGCCCTGACCGCACTAATCAGCTTATACTTCTACCTACGACTATGCTACGCAATGACACTAACCATCTCCCCCAATACAACCAACTCAATTACCCCTTGACGAACCAAAACAACTCAAGCCGCCCTACCCCTCGCCCTATTCATCACAGCCACCCTGGGACTATTACCCATAACCCCCACCATTATAATACTAACTACC

>A._iridescens

ATGAACCCATACGTACTTGCAATCCTACTATCCAGCCTAGGACTAGGAACTACCCTAACCTTTGCCAGCTCTCACTGACTCCTAGCCTGAATAGGCCTAGAAATTAATACACTAGCAATTGCCCCACTAATAGCACAACACCATCACCCCCGTGCAGTCGAAGCAACCACAAAATACTTCTTAACCCAAGCCACCGCAGCAGCAATGATCCTATTTGCAAGCACAACAAACGCATGAATAACAGGAGAATGAGACATCAACAACCTATCAAACCCCCTCGCCAACACAATATTTATAGCCGCCCTGGCACTTAAAATTGGACTTGCACCTGTACATTTCTGAATACCAGAAGTTCTGCAAGGACTAGACCTGCTAACAGGCCTAATCTTATCCACATGACAAAAACTTGCCCCATTTGCACTAATTATTCAAACGGCACAAAATATTGACCCACTACTACTAACACTGCTAGGGATTACATCCACACTAATCGGGGGATGAGGAGGACTAAACCAAACTCAACTACGAAAAATCCTAGCCTATTCCTCAATTGCCCACATAGGATGAATGATTATCGTAATCCAATACGCCCCCCAACTTACTATACTTGCACTAGGAACATACATTATCATAACCTCCGCGACATTCCTAACCCTAAAAATATCTATAACAACCCAAATTAATACACTCGCAACAACCTGATCAAAAAACCCTATACTTGCATCAACAACTGCCCTAGTCCTACTATCACTAGGTGGCCTCCCTCCACTTACAGGTTTCATACCAAAATGAATAATTCTGCAAGAACTAACAAAACAAGACCTGCCCATCATCGCTACAACAATAGCCCTCACCGCACTAATCAGCCTATACTTCTACCTACGACTATGCTACGCAATAACTCTAACTATCTCCCCCAACACAACCAACGCAACCACCCCCTGACGAACCCAAACAACCCAAACCCCCCTACCACCTGCCCTATTCATCACAGCTACCCTGGGGCTATTACCAATAACCCCCACCATCCTAATACTAACCACC

>A._jishouensis

ATGAACCCATACGTACTTGCAACCCTACTATCCAGCCTAGGACTAGGCACCACCTTAGCCTTTGCCAGCTCCCACTGACTTCTGGCCTGAATGGGCCTGGAGATTAATACACTGGCAATTACCCCACTAATAGCACAACACCACCACCCCCGTGCGGTCGAAGCAACCACAAAATATTTCTTAACACAGGCCACCGCAGCAGCAATAATCTTATTCGCAAGCGCAACAAATGCATGAATAACAGGAGAATGAAGCATCAACAACCTATCAAACCCCCTCGCTAGCATGATATTTACAGCTGCCCTGGCACTCAAAATTGGACTAGCACCCGTACACTTCTGAATGCCAGAAGTCCTACAAGGATTAGACCTGTTAACAGGCCTAATCTTGTCCACCTGACAAAAACTTGCCCCATTCGCACTAATTATCCAAACAGCACAAAATATTGACCCGCTACTACTTACACTACTAGGAATTACATCCACACTAGTTGGAGGATGGGGGGGACTAAACCAAACCCAACTACGAAAGATCCTAGCCTACTCCTCAATCGCCCACATGGGATGAATGATTATTGTAATCCAGTACGCCCCACAACTCACCCTTCTTGCACTAGTAACATATATCATCATGACCTCCGCAGCATTTATGACCCTAAAAATATCAATAACAACCAAAATTAATACACTCGCAACAACCTGATCGAAAAGCCCCGCGCTCACGTCAACAACCGCCCTGGTCCTATTATCATTAGGCGGCCTCCCACCACTTACAGGGTTTATACCAAAATGAATAATTCTACAAGAACTGACAAAACAAGACCTACCCATCGTCGCCACAACAATAGCCCTGACCGCACTAATTAGCTTATACTTCTACTTACGACTATGTTACGCAATAACACTAACTATCTCTCCAAATACAACCAACTCAATCACCCCCTGACGGACCAAAACAACCCAAACCACCCTGCCACTCGCCCTATCCATCACGGCCACCCTAGGACTATTACCTATAACCCCCACCATTGTAATACTAACCACC

>A._kreyenbergii_1

ATGAACCCATATGTACTTGCAGTCCTACTATCTAGCCTAGGATTAGGAACCACCCTAACCTTTGCCAGCTCCCACTGACTTCTAGCCTGAATAGGCCTAGAAATTAATACACTAGCAATTGCCCCGTTAATAGCACAACACCACCATCCCCGTGCAGTTGAAGCAACCACAAAATACTTCTTAACGCAGGCCACCGCAGCAGCAATAATCTTATTTGCAAGCACAACAAATGCATGGATAACAGGAGAGTGAAGCATCAACAACCTATCAAACCCTCTCGCTAGCACAATATTTACAGCCGCCCTGGCACTCAAAATCGGACTTGCACCCGTGCACTTCTGAATGCCAGAAGTCCTACAAGGGTTAGACCTGTTAACAGGACTAATCTTATCCACCTGACAAAAACTTGCCCCATTTGCACTGATTATCCAAACAGCACAAAGTATTGACCCACTACTTCTAACACTTCTAGGGGTTACATCTACACTAGTAGGGGGATGAGGGGGACTAAACCAAACCCAGCTACGAAAAATCCTAGCTTACTCCTCAATCGCCCACATGGGATGAATGATTATTGTAATTCAGTACGCCCCGCAACTCACCCTTCTTGCGCTAGGAACATACATTGTCATGACCTCCGCAACGTTCATAACCTTAAAAATATCAATAACGACCAAAATCAACACACTTGCAACAACTTGATCAAAAAGCCCTGTACTCACATCTACAACCGCCCTAGTCCTACTATCATTAGGCGGCCTCCCACCACTCACAGGATTTATACCAAAATGAATAATTTTACAAGAACTAACAAAACAAGACCTACCCATCATCGCCACAACAATAGCCTTAACTGCACTAATTAGCCTATACTTCTATTTACGACTATGCTACGCAATAACATTAACTATCTCTCCTAATACAACCAACTCAATTACCCCCTGACGGACCAAAACAACCCAAACCACTCTACCCCTCGCCCTATTCATCACAGCCACCTTAGGATTATTACCCGTAACCCCCACCATTATGATGCTAACCACC

>A._kreyenbergii_2

ATGAACCCATATGTACTTGCAGTCCTACTATCTAGCCTAGGATTAGGAACCACCTTAACCTTTGCCAGCTCCCACTGACTTCTAGCCTGAATAGGCCTAGAAATTAACACACTAGCAATTGCCCCACTAATAGCACAACACCACCATCCCCGTGCAGTTGAAGCAACCACAAAGTACTTCTTAACACAAGCCACCGCAGCAGCAATAATCCTATTTGCAAGCACAACGAATGCATGAATAACAGGAGAATGAAGTATCAACAACCTATCAAACCCTCTCGCTAGCACAATATTTACAGCCGCCCTGGCGCTCAAAATCGGGCTTGCACCCGTGCACTTCTGAATGCCAGAAGTTCTACAAGGATTAGACCTGTTAACAGGACTAATCCTGTCCACCTGACAAAAACTTGCCCCATTTGCACTGATTATCCAAACAGCACAAAATATTGACCCACTACTACTAACACTTCTAGGAGTTACATCCACACTGGTGGGGGGATGGGGGGGACTAAACCAAACTCAACTACGAAAAATCCTAGCCTACTCCTCAATCGCCCACATGGGATGAATAATTATCGTAATTCAGTACGCCCCTCAACTCACCCTCCTTGCGCTAGGAACATACATTATCATGACCTCCGCAACATTCATAACCTTAAAGATATCAATAACAACCAAAATCAATACACTTGCAACAACTTGATCAAAAAGCCCTGTGCTTACATCTACAACCGCCCTAATCCTACTATCATTAGGTGGCCTCCCACCACTCACGGGATTTATACCAAAATGAATAATTCTACAAGAACTGACAAAACAAGACCTACCCATCATCGCCACAACAATAGCCTTAACTGCACTAATCAGCCTATACTTCTATTTGCGGCTATGCTACGCAATAACATTAACAATCTCCCCCAATACAACCAACTCAATCACCCCCTGACGAACCAAAACAACCCAAACCACCCTGCCACTCGCCCTATTCATCACGGCCACCTTAGGACTATTACCCGTAACCCCCACCATTATAATGCTGACCACC

>A._longipinnis

ATGAACCCATACGTACTTGCAATCCTACTATCCAGCCTAGGACTAGGAACTACCCTAACCTTTGCCAGCTCTCACTGACTCCTAGCCTGAATAGGCCTAGAAATTAATACACTAGCAATTGCCCCACTAATAGCACAACACCATCACCCCCGTGCAGTCGAAGCAACCACAAAATACTTCTTAACCCAAGCCACCGCAGCAGCAATGATCCTATTTGCAAGCACAACAAACGCATGAATAACAGGAGAATGAGACATCAACAACCTATCAAACCCCCTCGCCAACACAATATTTATAGCCGCCCTGGCACTTAAAATTGGACTTGCACCTGTACATTTCTGAATACCAGAAGTTCTGCAAGGACTAGACCTGCTAACAGGCCTAATCTTATCCACATGACAAAAACTTGCCCCATTTGCACTAATTATTCAAACGGCACAAAATATTGACCCACTACTACTAACACTGCTAGGGATTACATCCACACTAATCGGGGGATGAGGAGGACTAAACCAAACTCAACTACGAAAAATCCTAGCCTATTCCTCAATCGCCCACATAGGATGAATGATTATCGTAATCCAATACGCCCCCCAACTTACTATACTTGCACTAGGAACATACATTATTATAACCTCCGCGACATTCCTAACCCTAAAAATATCTATAACAACCAAAATTAATACACTCGCAACAACCTGATCAAAAAACCCTATACTTGCATCAACAACTGCCCTAGTCCTACTATCACTAGGTGGCCTCCCTCCACTTACAGGATTCATACCAAAATTAATAATTCTTTTGGAACTAACAAAACAAGACCTGCCCATCATGGCCACAACAATAGCCCTCTCGGTCGTGATCAGCCTATATTTCTACCTATGAGTATGTTACGCAATAACTCTAACTATCTCCCCCAACACAACCAACGCAACCACCCCCTGACGAACCCAAACAACCCAAGCCCCCCTACCACCTGCCCTATTCATCACAGCTACCCTGGGGCTATTACCAATAACCCCCACCATCCTAATACTAACCACC

>A._monticola_1

ATGAACCCATATGTACTTGCAATCCTTCTATCCAGCCTAGGATTAGGAACTACATTAACCTTTGCTAGCTCCCACTGACTCCTAGCCTGAATGGGCCTAGAAATTAATACACTAGCAATCGCCCCCCTAATAGCACAACACCATCACCCCCGTGCAGTTGAAGCAACTACAAAATACTTCTTAACCCAAGCCACCGCAGCAGCAATGATCCTTTTCGCAAGCACAACGAATGCCTGAATAACAGGAGAATGAAGCATCAACAACCTATCGAACCCCCTAGCCAACACGATATTCATAGCCGCCCTAGCACTTAAAATCGGACTCGCACCAGTACATTTCTGAATGCCAGAAGTCCTACAGGGACTAGACCTACTAACGGGCCTAATCCTATCCACCTGACAAAAACTCGCCCCATTCGCACTAATCATCCAAACAGCACAAAACATTGACCCACTACTCCTAACACTGTTAGGAGTAATATCCACATTAGTAGGTGGATGAGGAGGACTAAACCAAACCCAACTACGAAAAATCCTAGCCTACTCCTCGATCGCCCACATAGGATGAATAATTATTGTAATCCAATACGCCCCACAGCTTACCCTCCTCGCACTAGGAACATATATTATCATAACCTCCGCAACATTCTTAACCCTGAAAACATCATTAACAACCAACATTAGTACACTTGCAACAACCTGATCAAAAAACCCCGTGCTCGCATCAACAACTGCCTTAGTACTACTGTCACTTGGTGGCCTCCCACCACTCACAGGGTTTATACCAAAATGAATAATTCTACAAGAACTAACAAAACAAAACCTCCCCATCATCGCCACAACAATAGCCCTAACCGCCCTAATCAGCCTGTACTTCTACCTACGACTATGCTACGCAATAACACTAACCGTCTCCCCCAGCACAACCAACTCAACCACCCCCTGACGAACTCAATCAACCCAAACCTCCCTACCAACCGCCCTATTCATCACAGCCACCCTAGGACTTCTACCAATAACCCCAGCCATTTTAATAATAGCCACC

>A._monticola_2

ATGAACCCATATGTACTTGCAATCCTTCTATCCAGCCTAGGATTAGGAACTACATTAACCTTTGCTAGCTCCCACTGACTCCTAGCCTGAATGGGCCTAGAAATTAATACACTAGCAATCGCCCCCCTAATAGCACAACACCATCACCCCCGTGCAGTTGAAGCAACTACAAAATACTTCTTAACCCAAGCCACCGCAGCAGCAATGATCCTTTTCGCAAGCACAACGAATGCCTGAATAACAGGAGAATGAAGCATCAACAACCTATCGAACCCCCTAGCCAACACGATATTCATAGCCGCCCTAGCACTTAAAATCGGACTCGCACCAGTACATTTCTGAATGCCAGAAGTCCTACAGGGACTAGACCTACTAACGGGCCTAATCCTATCCACCTGACAAAAACTCGCCCCATTCGCACTAATCATCCAAACAGCACAAAACATTGACCCACTACTCCTAACACTGTTAGGAGTAATATCCACATTAGTAGGTGGATGAGGAGGACTAAACCAAACCCAACTACGAAAAATCCTAGCCTACTCCTCGATCGCCCACATAGGATGAATAATTATTGTAATCCAATACGCCCCACAGCTTACCCTCCTCGCACTAGGAACATATATTATCATAACCTCCGCAACATTCTTAACCCTGAAAACATCATTAACAACCAACATTAGTACACTTGCAACAACCTGATCAAAAAACCCCGTGCTCGCATCAACAACTGCCTTAGTACTACTGTCACTTGGTGGCCTCCCACCACTCACAGGGTTTATACCAAAATGAATAATTCTACAAGAACTAACAAAACAAAACCTCCCCATCATCGCCACAACAATAGCCCTAACCGCCCTAATCAGCCTGTACTTCTACCTACGACTATGCTACGCAATAACACTAACCGTCTCCCCCAGCACAACCAACTCAACCACCCCCTGACGAACTCAATCAACCCAAACCTCCCTACCAACCGCCCTATTCATCACAGCCACCCTAGGACTTCTACCAATAACCCCAGCCATTTTAATAATAGCCACC

>A._paradoxus_1

ATAAACCCATACGTTCTTATAGTTCTACTATCTAGCCTGGGACTAGGAACCACCCTAACCTTTGCCAGCTCCCACTGACTCCTAGCCTGAATAGGCCTGGAAATTAACACACTAGCAATTACACCACTAATAGCACAACACCACCACCCCCGTGCAATTGAAGCAACCACAAAGTACTTCTTAACACAAGCCACCGCAGCAGCAATAATCTTGTTCGCAAGCACAACAAATGCGTGAATAACAGGAGAATGGGACATCAATAACCTATCAAACCCACTCGCCACCACAATATTTACAGCCGCCCTAGCGCTCAAAATCGGACTTGCCCCTCTTCACTTCTGGATACCAGAAGTTCTACAGGGACTAGACCTGCTAACAGGCCTAATCCTATCCACCTGACAAAAACTCGCCCCATTCGCATTAATTGTCCAAACAGCACAGAGCATTGACCCACTACTACTGACACTACTAGGAATTACATCTACCCTTGTAGGGGGGTGAGGGGGACTAAACCAAACCCAACTACGAAAAATTTTAGCCTACTCCTCAATCGCCCACATAGGATGAATAATTATTGTAATCCAGTACGCCCCCCAACTTACTCTTCTTGCACTAGGAACATACATCATCATAACCTCCGCAACATTTATAGCCCTAAAAATATTAATAACAACCAAAATTAATACACTTGCAACGGCCTGATCAAAGAGCCCTACGCTTGCATCAATAACCGCCCTAATTTTACTATCATTAGGCGGCCTCCCACCACTCACGGGATTCATACCAAAATGAATAATTCTACAAGAACTAACGAAACAAGACCTGCCCATCATCGCTACAACAATAGCCCTAGCCGCCCTAATTAGTCTATACTTCTACCTGCGACTGTGCTACGCAATGGCATTAACCATCCCCCCAAGTACTAGTAATTCAATCACCCCCTGACGAACCAAAACAACCCAAACTATTTTACCACTCGCCCTGCTCATCACAGCCACTCTGGGACTACTACCAGTTACCCCCACCATTATGACATTCATCACC

>A._paradoxus_2

ATGAACCCATACGTACTTGCAACCCTACTATCCAGCCTAGGACTAGGCACCACCTTAGCCTTTGCCAGCTCCCACTGACTTCTGGCCTGAATGGGCCTGGAGATTAATACACTGGCAATTACCCCACTAATAGCACAACACCACCACCCCCGTGCGGTCGAAGCAACCACAAAATATTTCTTAACACAGGCCACCGCAGCAGCAATAATCTTATTCGCAAGCGCAACAAATGCATGAATAACAGGAGAATGAAGCATCAACAACCTATCAAACCCCCTCGCTAGCATGATATTTACAGCTGCCCTGGCACTCAAAATTGGACTAGCACCCGTACACTTCTGAATGCCAGAAGTCCTACAAGGATTAGACCTGTTAACAGGCCTAATCTTGTCCACCTGACAAAAACTTGCCCCATTCGCACTAATTATCCAAACAGCACAAAATATTGACCCGCTACTACTTACACTACTAGGAATTACATCCACACTAGTTGGAGGATGGGGGGGACTAAACCAAACCCAACTACGAAAGATCCTAGCCTACTCCTCAATCGCCCACATGGGATGAATGATTATTGTAATCCAGTACGCCCCACAACTCACCCTTCTTGCACTAGTAACATATATCATCATGACCTCCGCAGCATTTATGACCCTAAAAATATCAATAACAACCAAAATTAATACACTCGCAACAACCTGATCGAAAAGCCCCGCGCTCACGTCAACAACCGCCCTGGTCCTATTATCATTAGGCGGCCTCCCACCGCTTACAGGGTTTATACCAAAATGAATAATTCTACAAGAACTGACAAAACAAGACCTACCCATCGTCGCCACAACAATAGCCCTGACCGCACTAATTAGCTTATACTTCTACTTACGACTATGTTACGCAATAACACTAACTATCTCTCCAAATACAACCAACTCAATCACCCCCTGACGGACCAAAACAACCCAAACCACCCTGCCACTCGCCCTATCCATCACGGCCACCCTAGGACTATTACCTATAACCCCCACCATTGTAATACTAACCACC

>A._parallens_1

ATGAACCCATACGTACTTGCAACCCTACTATCCAGCCTAGGACTAGGGACCACCCTAACCTTTGCCAGCTCCCACTGACTTCTAGCCTGAATAGGCCTGGAGATTAATACGCTAGCAATTGCCCCACTAATAGCACAACACCACCACCCCCGTGCAGTTGAAGCAACCACAAAATACTTCTTAACACAAGCCACCGCAGCAGCAATGATCTTGTTCGCAAGCACAACAAATGCATGAATAACAGGAGAATGAAGCATCAATAACCTGTCAAACCCCCTCGCCAGCACAATATTCACAGCCGCCCTGGCACTCAAAATTGGACTTGCGCCCGTACACTTCTGAATACCAGAAGTTCTACAAGGATTAGACCTGTTAACAGGCCTAATCTTATCCACCTGACAAAAACTTGCCCCATTCGCACTAATTATCCAAACAGCACAAAATATTGACCCCTTACTACTAACACTACTAGGAGTCACGTCCACACTAGTAGGAGGATGGGGAGGACTAAACCAAACCCAGCTGCGAAAAATCCTAGCCTACTCCTCAATCGCCCACATGGGGTGAATAATTATTGTGGTTCAATACGCCCCACAGCTCACCCTTCTTGCACTAGGAACATACATCATCATAACATCCGCAGCATTTATAACCTTAAAAATGTCAATAACAACCAAAATTGGTACACTCGCAACAACCTGATCAAAAAGCCCTGCACTCACATCAACAACCGCCCTGGTCTTATTATCATTAGGCGGCCTCCCACCCCTTACAGGGTTTATACCAAAATGAATAATCCTACAAGAACTAACAAAACAGGACCTACCCATCATCGCCACAACAATGGCCCTGACCGCACTAATTAGCTTATACTTCTACCTGCGACTGTGCTACGCGATGACGCTAACTATCTCTCCCAATACAACCAACTCAATTACCCCTTGACGAACCAAAACAACTCAAGCCGCCCTGCCCCTCGCCCTATTCATCACAGCCACCCTAGGACTATTACCCGTAACCCCCACCATTATAATACTAACTACC

>A._parallens_2

ATGAACCCATACGTACTTGCAACCCTGCTATCCAGCCTAGGACTAGGAACCACCCTAACCTTTGCCAGCTCCCACTGACTTCTGGCCTGAATGGGCCTGGAGATTAATACACTAGCAATTGCCCCACTAATAGCACAACACCACCACCCCCGTGCAGTTGAAGCAACCACAAAATACTTCTTAACACAAGCCACCGCAGCAGCAATGATCTTGTTCGCAAGCACAACAAATGCATGAATAACAGGAGAATGAAGCATCAATAACCTATCAAACCCCCTCGCCAGCACAATATTCACAGCCGCCCTGGCACTCAAAATTGGACTTGCACCCGTACACTTCTGAATACCAGAAGTCCTACAAGGATTAGACCTGTTAACAGGCCTAATCTTATCTACCTGACAAAAACTTGCCCCATTCGCACTAATTATCCAGACAGCACAAAATATTGACCCCTTACTACTAACACTACTAGGAGTCACATCCACACTAGTAGGGGGATGGGGAGGACTAAACCAAACCCAGCTACGAAAAATCCTAGCCTACTCCTCAATCGCCCACATGGGGTGAATAATTATTGTAGTCCAATACGCCCCACAACTCACCCTTCTTGCACTAGGAACATACATCATCATAACGTCCGCAGCATTTATAACCCTAAAAATATCAATAACAACCAAAATTAGTACACTCGCAACGACCTGATCAAAAAGCCCTGCACTCACATCAACAACCGCCCTCGTCTTATTATCATTAGGCGGCCTCCCGCCCCTTACAGGGTTTATACCAAAATGAATAATCCTACAAGAACTAACAAAACAAGACCTACCCATCGTCGCCACAACAATGGCCCTCACCGCACTAATTAGCTTGTACTTCTACCTGCGACTATGCTACACGATGACACTAACTATCTCTCCCAATACAACCAACTCAATTACCCCTTGACGGACCAAAACAACTCAAGCTGCCCTGCCCCTCGCCCTATTTATCACAGCCACCCTAGGACTATTACCCATAACCCCCACCATTATAATACTAACTACC

>A._parallens_3

ATGAACCCATACGTACTTGCAACCCTACTATCCAGCCTAGGACTAGGGACCACCCTAACCTTTGCCAGCTCCCACTGACTTCTGGCCTGAATAGGCCTGGAGATTAATACACTAGCAATTGCCCCACTAATAGCGCAACACCACCACCCCCGTGCAGTTGAAGCAACCACAAAATACTTCTTAACACAAGCCACCGCAGCAGCAATGATCTTGTTCGCAAGCACAACAAATGCATGAATAACAGGAGAATGAAGCATCAATAACCTATCAAACCCCCTCGCCAGCACAATATTCACGGCCGCCCTAGCACTCAAAATTGGACTTGCACCCGTACACTTCTGAATACCAGAAGTCCTACAAGGATTAGACCTGTTAACAGGCCTAATCTTATCTACCTGACAAAAACTTGCCCCATTCGCACTAATCATCCAAACAGCACAAAATATTGACCCCCTACTACTAACACTACTAGGAGTCACATCCACACTAGTAGGAGGATGGGGGGGACTAAACCAAACCCAGCTACGAAAAATCCTAGCCTACTCTTCAATCGCCCACATGGGGTGGATAATTATTGTGGTTCAGTACGCCCCACAACTCACCCTTCTTGCACTAGGAACATACATCATCATAACATCCGCAGCATTTATAACCCTAAAAATATCAATAACAACCAAAATTGGTACACTCGCAACAACCTGATCAAAAAGCCCTGCACTCACATCAACAACCGCCCTGGTCTTATTATCATTAGGCGGCCTCCCACCCCTTACAGGGTTTATACCAAAATGAATAATCCTACAAGAACTAACAAAACAAGACCTACCCATCGTCGCCACAACAATGGCCCTAGCCGCACTAATTAGCTTATACTTCTACCTACGACTATGCTACGCGATGACACTAACTATCCCTCCCAATACAACCAACTCAATTACCCCTTGGCGAACCAAAACAACTCAAGCCGCCCTGCCCCTCGCCCTATTCATTACAGCCACCCTAGGACTATTACCCATAACCCCCACCATTATAATACTAACTACC

>A._parallens_4

ATGAACCCATATGTACTTGCAACCTTACTATCCAGCCTAGGACTAGGGACCACCCTAACCTTTGCCAGCTCCCACTGACTTCTGGCCTGAATGGGCCTGGAGATTAATACACTAGCAATTGCCCCACTAATAGCACAACACCACCACCCCCGTGCAGTTGAAGCAACCACAAAATACTTCTTAACACAAGCCACCGCAGCAGCAATGATCTTGTTCGCAAGCACAACAAATGCATGAGTAACAGGAGAATGAAGCATCAATAACCTGTCAAACCCCCTCGCCTGCACAATATTCACAGCCGCCCTAGCACTCAAAATTGGACTTGCACCCGTACACTTCTGAATACCAGAAGTCCTACAAGGATTAGACCTGTTAACAGGCCTAATCTTATCTACCTGACAAAAACTTGCCCCATTCGCACTAATTATCCAAACAGCACAAAATATTGACCCCTTACTACTAACACTACTAGGAGTCACATCCACACTAGTAGGAGGATGGGGGGGACTAAACCAAACCCAGCTACGAAAAATCCTAGCCTACTCTTCAATCGCCCACATAGGATGGATAATTATTGTGGTTCAGTACGCCCCACAGCTCACCCTTCTTGCACTAGGAACATACATCATCATAACATCCGCAGCATTTATAACCCTAAAAATATCAATAACAACCAAAATTGGTACACTCGCAACAACCTGATCAAAAAGCCCTGCGCTCACATCAACAACCGCCCTGGTCTTATTATCATTAGGCGGCCTCCCACCCCTTACAGGGTTTATACCAAAGTGAATAATCCTACAAGAACTAACAAAACAAGACCTACCCATTGTCGCCACAACAATGGCCCTGACCGCACTAATTAGCCTATACTTCTACCTACGACTATGCTACGCAATGACACTAACTATCTCTCCCAATACAACCAACTCAATTACCCCTTGGCGAACCAAAACAACTCAAGCCGCCCTGCCCCTCGCCCTATTCATTACAGCCACCCTAGGACTATTACCCATAACCCCCACCATTATAATACTAACTACC

>A._spinifer

ATCAGTCCATTTGTCCTCATAATCCTATTGTCCAGCCTGGGACTAGGAACTACTCTAACTTTTGCCAGCTCCCACTGACTCCTAGCCTGAATGGGCCTAGAAATTAATACACTGGCAATTGCCCCACTAATAGCACAACACCACCATCCCCGTGCAATTGAAGCGACTACAAAATACTTCTTAACACAAGCCACCGCAGCAGCAATAATCTTATTCGCAAGCACAACAAATGCATGGATAACAGGAGAATGAAGCATCAACAACCTGTCAAACCCACTCGCCTCCACAATATTTACAGCCGCTCTAGCACTCAAAATCGGACTTGCCCCTGTACACTTCTGAATACCAGAAGTCCTACAAGGACTAGACCTGCTAACAGGCCTAATCCTATCCACCTGACAAAAACTTGCCCCATTCGCATTAATTGTCCAAACAGCACACAACATTGACCCACTACTATTAACACTACTAGGAATTACATCCACACTAGTTGGGGGATGAGGAGGACTGAACCAAACCCAACTACGAAAAATCTTAGCTTACTCCTCAATCGCCCACATAGGATGAATAGTTATCGTGATCCAATATGCCCCACAACTCACTCTCCTCGCACTAGGAACATATATTATCATGACCTCCGCAACATTCATAACCCTAAAAATATCAATAACAACCAAGATTAATACACTTGCAACAACTTGATCGAAAAGCCCTACACTTGCGTCAATAACTGCCCTAGTCTTACTATCACTAGGCGGCCTCCCACCACTTACAGGATTTATACCAAAATGAATAATTTTACAAGAGCTAACAAAACAAGACCTACCTACCATCGCTACAATAATGGCCCTCACCGCACTAATTAGCCTGTACTTCTACCTACGGCTATGTTACACAATGACACTAACCATCTCCCCCAATACAAACAACTCAGTCACCCCCTGACGAACCAAAACAACCCAAACCACCCTACCACTCGCCCTACTCATCACAGCCGCCCTGGGACTCCTGCCGATGACCCCTACCATTATAATACTAACTACC

>A._stenotaeniatus

ATCAGTCCATTCGTCCTCATAATCCTATTGTCCAGCCTAGGACTAGGAACTACTCTAACTTTTGCCAGCTCCCACTGACTCCTAGCCTGAATGGGCCTAGAAATTAATACACTGGCAATTGCCCCACTAATAGCACAACACCACCATCCCCGTGCAATTGAAGCGACTACAAAATACTTCTTAACACAAGCCACCGCAGCAGCAATAATCTTATTCGCAAGCACAACAAATGCATGAATAACAGGAGAATGAAGCATCAACAACCTATCAAACCCACTCGCCTCCACAATATTTACAGCCGCTCTAGCACTCAAAATCGGACTTGCCCCTGTACACTTCTGAATACCAGAAGTCCTACAAGGACTAGACCTGCTAACAGGCCTAATCCTATCCACCTGACAAAAACTTGCCCCATTCGCATTAATTGTCCAAACAGCACACAACATTGACCCACTACTATTAACACTACTAGGAGTTACATCTACACTAGTCGGGGGATGAGGAGGACTGAACCAAACCCAACTACGAAAAATCTTAGCTTACTCCTCAATCGCCCATATAGGATGAATAGTTATCGTGATCCAATACGCCCCACAACTCACTCTCCTCGCACTAGGAACATATATTATCATGACCTCCGCGACATTCATAACCCTAAAAATATCAATAACAACCAAAATTAATACACTTGCAACAACTTGATCGAAAAGCCCTACACTTGCGTCAATAACTGCCCTAGTCTTACTATCACTAGGCGGCCTCCCACCACTCACAGGATTTATACCAAAATGAATAATTTTACAAGAGCTAACAAAACAAGACCTACCTACCATCGCTACAATAATGGCCCTCACCGCAGTGATTAGCCTGTACTTCTACCTACGGCTATGTTACACAATAACACTAACCATCTCCCCCAATACAAACAACTCAGTCACCCCCTGACGAACCAAAACAACCCAAACCACCCTACCACTCGCCCTACTCATCACAGCCGCCCTGGGACTCCTGCCGATGACCCCTACCATTATAATACTAACCACC

>A._wenchowensis_1

ATGAACCCATATGTACTTGCAGTCCTACTATCCAGCCTAGGGTTAGGAACCACCTTAACCTTTGCCAGCTCCCACTGACTTCTAGCCTGAATAGGTCTAGAGATTAACACACTAGCAATTGCCCCACTAATAGCACAACACCACCACCCCCGTGCAGTTGAAGCGACCACAAAATACTTCTTAACACAAGCCACCGCAGCAGCAATAATCCTATTCGCAAGCACAACAAATGCATGAATAACAGGAGAATGAAGCATCAACAACCTATCAAACCCTCTTGCCAGCACAATATTTACAGCCGCCCTAGCACTCAAAATCGGACTTGCACCCGTACACTTCTGAATACCAGAAGTCCTGCAAGGATTAGACCTGTTAACAGGACTAATCTTATCCACCTGACAAAAACTTGCCCCGTTTGCACTAATTATCCAAACAGCACAAAATATTGACCCACTACTACTAACACTTCTAGGAGTTACATCCACACTGGTAGGAGGATGGGGGGGATTGAACCAAACCCAGCTACGAAAAATCCTAGCCTACTCCTCAATCGCCCATATAGGATGAATAATTATTGTAATTCAATACGCCCCACAACTCACCCTTCTTGCACTAGGGACATACATTATCATAACCTCCGCAACATTCATAACCCTAAAAATATCAATAACAACCAAAATCAATACACTTGCAACAACCTGATCAAAAAGCCCTGTACTCGCATCTACAACCGCCCTGGTCCTACTATCATTAGGCGGCCTCCCACCACTCACAGGGTTTATACCAAAATGAATGATTCTACAAGAACTAACAAAACAAGACCTACCCATCATCGCCACAACAATAGCCTTAACCGCACTAATTAGCCTATACTTCTACTTACGACTATGCTACGCAATGACATTAACTATCTCTCCCAACACAACCAACTCAATCGCTCCCTGACGAGCCAAAACAACCCAAACCACCTTGCCACTCGCCCTATTTATCACGGCCACCCTAGGACTATTACCCGTAACCCCCACCATTATAATGCTATCCACC

>A._wenchowensis_2

ATGAACCCATATGTACTTACAGTCCTACTATCCAGCCTAGGGTTAGGAACCACCTTAACCTTCGCCAGCTCACACTGACTTCTAGCCTGAATGGGTCTAGAAATTAACACGCTAGCAATTGCCCCACTAATAGCACAACACCACCACCCCCGTGCAGTTGAAGCAACCACAAAATACTTCTTAACCCAAGCCACCGCAGCAGCAATAATCTTATTTGCAAGCACAACAAATGCATGAATAACAGGAGAATGAAGCATCAACAACCTCTCAAACCCTCTCGCTAGCACAATATTTACAGCCGCCCTAGCACTCAAGATCGGACTTGCACCCGTACACTTCTGAATACCAGAAGTCCTACAAGGACTGGACCTGTTGACAGGATTAATCTTGTCCACCTGACAAAAACTTGCCCCATTTGCACTAATTATCCAAACAGCACAAAACATTGACCCGCTACTACTAACTCTTCTAGGGGTTACATCTACACTAGTAGGGGGATGGGGAGGACTAAACCAAACCCAGCTACGAAAAATCCTGGCCTACTCCTCAATCGCCCACATAGGGTGAATAATTATTGTGATTCAATACGCCCCACAACTTACCCTTCTTGCACTAGGAACATACATTATCATGACCTCCGCAACATTCATAACCTTTAAAATATCAATGACAACCAAAATTAATACCCTTGCAACAACCTGATCAAAAAGCCCCGTGCTCGCATCTACAACCGCCCTGGTCCTGCTATCATTGGGCGGCCTCCCACCACTCACCGGGTTTATACCAAAATGAATAATTCTACAAGAACTAACAAAACAAGACCTACCCATCATCGCCACAACAATAGCCTTAACCGCACTAATTAGCCTGTACTTCTATTTACGACTGTGCTACGCAATGACATTAACTATCTCCCCCAATACAACCAACTCAATCGCCCCCTGACGGACCAAAACAACCCAAACCACCCTGCCGCTCGCCCTATTTATTACAGCCACCCTAGGACTACTGCCCGTAACCCCCACCATTATAATGCTATCCACC

>A._wuyiensis

ATAAACCCATACGTTCTTATAGTTCTATTATCTAGCCTGGGACTAGGAACCACCCTAACCTTTGCCAGCTCCCACTGACTCCTAGCCTGAATAGGCCTGGAAATTAATACACTAGCAATTACACCACTAATAGCACAACACCACCACCCCCGTGCAATTGAAGCAACCACAAAATACTTCTTAACACAAGCCACCGCGGCAGCAATAATCTTATTCGCAAGCACAACAAATGCGTGAATAACAGGAGAGTGGGACATCAATAACCTATCAAACCCACTCGCCACCACAATATTTACAGCCGCCCTAGCGCTCAAAATCGGACTTGCCCCTCTTCACTTCTGAATACCAGAAGTTCTACAAGGACTAGACCTGCTAACAGGCCTAATCCTATCCACCTGACAAAAACTCGCCCCATTCGCATTAATTGTCCAAACAGCACAAAGCATTGACCCACTACTATTGACACTACTAGGAATTACATCTACGCTAGTAGGAGGATGAGGGGGACTAAACCAAACCCAACTACGAAAAATTTTAGCCTACTCCTCAATCGCCCACATAGGATGAATGATTATTGTGATCCAGTACGCCCCCCAACTCACTCTTCTTGCACTAGGAACATACATCATCATAACCTCCGCAACATTCATAGCCCTAAAAATATTAATAACAACCAAAATCAATACACTTGCAACGGCCTGATCAAAGAGCCCTACGCTTGCATCATTAACCGCCCTAGTTTTACTATCATTAGGCGGCCTCCCACCACTAACGGGATTCATACCAAAATGAATAATCCTACAGGAACTAACAAAACAAGACCTGCCCATCATCGCTACAACAATAGCCCTAGCCGCCCTAATTAGTCTATACTTCTACCTACGACTGTGCTACGCAATGACACTAACCATCCCCCCAAGTACTAGTAATTCAATCACCCCCTGACGAACCAAAACAACCCAAACTATTCTACCACTCGCCCTACTCATCACAGCCACTCTGGGACTACTACCAGTTACCCCCACCATTATGACATTCATCACC

>A._yunnanensis_1

ATGAACCCATACGTACTCGCAATCCTACTATCCAGCCTAGGACTAGGAACTACACTAACCTTTGCTAGCTCCCACTGGCTCCTAGCCTGAATGGGCCTAGAGATTAACACACTAGCAATCGCCCCCTTAATAGCACAACACCACCACCCCCGTGCAGTTGAAGCGACTACAAAATACTTCTTAACCCAAGCCACCGCAGCAGCAATAATCTTGTTTGCAAGCACAACAAATGCATGAATAACGGGAGAGTGAAGCATCAACAACCTATCAAACCCCCTCGCCAACACAATATTCATGGCCGCCCTAGCGCTTAAAATCGGGCTTGCACCAGTGCACTTCTGAATACCAGAAGTCCTACAAGGACTAGACCTGCTGACAGGCCTAATTCTATCCACCTGACAAAAACTCGCCCCATTTGCACTAATTATCCAAACAGCACAAAATATTGACCCCCTATTACTAACACTATTAGGAATGGCATCCACACTAGTTGGAGGATGAGGAGGACTAAACCAAACCCAACTACGAAAAATCCTAGCTTACTCTTCAATTGCTCACATAGGATGAATGATTATTGTAATCCAATACGCCCCACAACTTACCCTCCTTGCACTAGGAACATATATTATCATAACCTCCGCAACATTCCTAACCCTAAAGATATCATTAACAACTAATATTAGCACGCTCGCAACAACCTGATCAAAAAACCCTGTACTTGCATCAACAACTGCCTTAGTCCTACTATCACTAGGCGGCCTTCCGCCACTTACAGGATTTACACCAAAATGAATAATCCTACAAGAATTAACAAAACAAGACCTCCCCATCATCGCCACAATAATAGCCCTAACCGCGCTAATCAGTCTATACTTTTATCTACGGCTATGCTACGCAATAACACTAACCGTCTCCCCTAACACAATCAACTCAACCACCCCCTGACGGACCCACTCAACCCAAACCTCCCTGCCAACTGCCCTATTTATCACAGCCACCTTAGGACTCCTACCAATAACCCCAGCTATCCTAATAATAACCACC

>A._yunnanensis_2

ATGAACCCATACGTACTCGCAATCCTACTATCCAGCCTAGGACTAGGGACTACACTAACCTTTGCTAGCTCCCACTGGCTCCTAGCCTGAATGGGCCTAGAGATTAATACACTAGCAATCGCCCCCCTAATAGCACAACACCACCACCCCCGTGCAGTTGAAGCGACTACAAAATACTTCTTAACCCAAGCCACCGCAGCAGCAATAATCTTGTTCGCAAGCACAACAAATGCATGAATAACGGGAGAGTGAAGCATCAACAACCTATCAAACCCCCTCGCCAACACAATATTCATAGCCGCCCTAGCGCTTAAAATCGGACTTGCACCAGTGCATTTCTGAATACCAGAAGTCCTACAAGGACTAGACCTGCTAACAGGCCTAATTCTATCCACCTGACAAAAACTCGCCCCCTTTGCCCTAATTATCCAAACAGCACAAAATATTGACCCCCTATTACTAACACTATTAGGAATGGCATCCACACTAGTAGGAGGATGAGGGGGACTAAACCAAACCCAACTACGAAAAATTCTAGCCTACTCTTCAATTGCTCACATAGGATGAATGATTATCGTAATCCAATACGCCCCACAACTTACCCTACTTGCACTAGGAATATACATTATCATAACCTCCGCAACATTCCTAACCCTAAAAATATCATTAACAACCAATATTAGCACCCTCGCAACAACCTGATCAAAAAACCCTGTACTTGCATCAACAACTGCCTTAGTCCTACTATCACTAGGCGGTCTTCCACCACTTACAGGATTTATACCAAAATGAATAATTCTACAAGAATTATCAAAACAAGACCTCCCCATCATCGCCACAATAATAGCCCTAACCGCACTAATCAGCCTATACTTTTATCTACGACTATGCTACGCAATAACACTAACCGTTTCCCCTAACACAATCAACTCAACCACCCCCTGACGGACCCACTCAACCCAAACCTCCCTGCCAACTGCCCTATTTATCACAGCCACCTTAGGACTCCTACCAATAACCCCAGCTATCCTAATAATAACCACC

>Onychostoma_barbatulum

ATGAACCCATATGTACTTATAATCCTACTATCCAGCCTAGGACTAGGAACCACCCTAACATTTGCCAGCTCCCACTGACTTTTAGCCTGAATAGGTCTAGAAATTAATACACTAGCAATCGCCCCACTGATAGCACAACACCACCACCCCCGTGCAGTCGAAGCAACTACAAAATATTTCTTAACCCAAGCCACCGCAGCAGCAATGATCCTATTCGCAAGCACAACAAATGCCTGAATAACAGGAGAATGAAGCATCAACAATTTATCAAACCCACTCGCCAACGCAATATTCATAGCCGCCTTAGCACTTAAAATCGGACTCGCACCAGTACATTTCTGAATACCAGAAGTTATACAAGGACTAGACCTGCTAACAGGCCTAATCCTATCCACCTGACAAAAACTTGCCCCATTTGCACTGATTATCCAAACGGCACAAAATATTGATCCACTACTACTAACACTACTAGGGGCCACATCCGCACTAGTCGGAGGGTGGGGGGGGCTAAACCAGACCCAACTACGAAAAATCTTAGCCTATTCCTCAATCGCCCACATAGGATGAATGATTATCGTAATTCAATATGCCCCACAACTCACCCTACTTGCACTAGGAACATACATCATCATGACCTCTGCAACATTCCTAACCCTGAAAATATCACTAACAACCAAAATTAATACACTCGCAACAACTTGGTCAAAAAACCCTGCGCTCGCATCAACAACCGCCTTAGTACTACTATCACTAGGGGGCCTCCCACCACTCACAGGATTTATACCAAAATGAATAATTTTACAAGAACTGACAAAACAAGACCTACCCATTATCGCCACAACAATAGCTTTAACTGCCCTAATCAGCCTGTACTTCTACCTACGACTATGTTATGCAATAACATTAACTGTTTCTCCTAACACAACCAACTCAACCACCCCATGACGAGCCCAAACAACCCAAACCTCTCTACCACTAGCCCTACTTATCACAGCCACCCTGGGGTTATTACCAATAACCCCAACCATCTTAATACTAGCCACC

>Onychostoma_meridionale

ATGAATCCATATGTACTCACAATCCTATTATCCAGCCTAGGACTAGGAACCACCCTAACCTTTGCCAGCTCCCACTGACTCCTAGCCTGAATGGGCTTAGAAATTAATACACTAGCAATCGCCCCCTTAATAGCACAACACCACCACCCCCGCGCAGTAGAGGCAACTACAAAATACTTTCTAACCCAAGCCACCGCAGCAGCAATAATCCTGTTTGCAAGCACAACAAACGCCTGAATAACTGGAGAATGAAGCATTAACCACCTATCAGACCCCCTTGCCAACACAACATTCGTAGCCGCTCTAGCACTCAAAATTGGACTCGCACCAATACACTTCTGAATGCCAGAAGTACTACAAGGGCTAGACCTATTAACAGGCCTAATCCTATCCACCTGGCAAAAACTCGCCCCATTCGCACTAATCATTCAAACAGCACAAAACATTAATCCACAATTACTAATACTGCTAGGAATCTCATCCACCCTAATTGGGGGATGAGGAGGACTAAACCAGACCCAACTACGAAAAATCCTGGCCTACTCCTCAATCGCCCACATAGGGTGAATAATTATTGTAATCCAATACGCCCCGCAACTCACACTACTTGCACTAATAACATATATTATTATAACCTCTGCGACATTCCTAACCCTAAAAATATCACTAACAACCAAAATTAACACACTCGCAACAACGTGGTCAAAAAACCCAATACTTGCATCAACAACTGCCTTAGTTTTACTATCACTAGGAGGCCTTCCGCCGCTCACAGGGTTTATACCAAAATGAATAATCCTACAAGAATTAGCGAAACAAGACCTACCCATTGTCGCCACAACAATAGCCCTAGCCGCACTAATTAGTCTATACTTCTACCTACGGCTATGCTACGCAATAACACTAACCATCTCCCCCAATACAAACAACTCAACCACCCCCTGACGAACTCAAACAACCCAAACCTCCCTACCACTCGCCCTATTCACCACAGCCACCCTGGGACTATTACCAATGACCCCCGCCATCCTAATACTAACCACC

>Onychostoma_gerlachi

ATGAACCCATATGTACTCACAATTCTACTATCCAGCCTAGGGCTAGGGACCACCCTAACCTTTGCCAGCTCCCACTGACTCCTAGCCTGAATGGGCCTAGAAATTAACACACTAGCAATCGCCCCACTAATAGCACAACACCACCACCCCCGTGCAGTAGAAGCAACTACAAAATACTTCTTAACCCAAGCCACCGCAGCAGCAATAATCCTATTCGCAAGCACAACAAACGCCTGAATAACTGGAGAATGAAGCATTAACCACTTATCAGACCCCCTCGCCAACACAACATTCATGGCCGCTCTAGCACTTAAAATCGGACTAGCACCAATACACTTCTGAATACCAGAAGTGCTACAAGGACTAGACCTACTAACAGGCCTAATCCTATCCACCTGACAAAAACTCGCCCCATTCGCACTAATCATTCAAACAGCACAAAACATTGACCCACAATTACTAATATTACTAGGAATCTCCTCCGCCCTAGTTGGAGGATGAGGAGGACTAAACCAAACCCAACTACGAAAAATCCTAGCCTACTCCTCAATCGCCCACATAGGATGAATGATTATCGTAATCCAATACGCCCCACAACTCACCCTACTTGCACTAATAACATATATTATTATAACCTCCGCAACATTCCTAACCCTAAAAATATCATTAACAACCAAAATTAACACACTCGCAACAACATGATCAAAAAACCCAATGCTCGCATCAACAACTGCCTTAGTCTTACTATCGCTAGGGGGCCTTCCACCACTCACAGGATTTATACCAAAATGAATAATTCTACAAGAACTAACAAAACAAGACCTGCCCATCATCGCCACAACAATAGCCCTGGCCGCACTAATCAGCCTATACTTCTACCTACGACTATGTTACGCAATAACACTAACCGTCTCCCCCAACACAAACAACTCAACCACCCCCTGACGAACACAAACAACCCAAACCTCCCTACCACTTACTCTATTTACCACAGCCACCCTGGGATTATTACCAATAACCCCCGCCATCCTAATACTAACCACC

>Spinibarbus_denticulatus

ATGAACCCATACGTACTTACAATCCTATTATCCAGCCTAGGGCTAGGAACCACCTTAACCTTCGCCAGCTCTCATTGACTCCTAGCTTGAATAGGCCTAGAAATCAATACCCTAGCAATCGCCCCCTTAATAGCACAACATCACCACCCTCGCGCAGTAGAAGCGACTACAAAATACTTCTTAACTCAAGCTACTGCAGCCGCAATAATCCTGTTCGCAAGTACAACAAACGCCTGAATAACAGGAGAATGAAGCATTAATGATTTGTCAGACCCTATCGCCAACACAATATTCATAACCGCTTTAGCACTTAAAATTGGACTCGCACCAATACACTTCTGAATGCCCGAAGTGTTACAGGGATTAGACCTACTAACAGGCCTAATTCTATCCACCTGACAAAAACTTGCCCCATTTGCACTAATTATCCAAACAATACAAAACATTGATCCATCACTATTAACACTTCTAGGAATTTTATCTACACTAGTGGGGGGATGAGGAGGGCTAAACCAAACCCAACTACGAAAAATCCTAGCCTACTCCTCAATTGCCCACATAGGGTGAATAGTTATTGTAATTCAATATGCCCCACAACTAACCCTAATTGCACTAGGAACATATATCATTATAACTTCCGCAGCATTCCTAACCCTAAAAATATCACTAACAACAAAAATTAGCACACTCACAACAACCTGGTCAAAGAGTCCCATGTTAGCGTCAACAACGGCCCTAGTCTTACTTTCACTAGGGGGCCTCCCGCCCCTCACAGGATTCATACCAAAATGAATAATTTTACAAGAGTTAACAAAACAGGATCTCCCCATCATTGCCACAACCATGGCCTTAGCTGCACTGATTAGTCTATACTTTTACCTACGATTATGCTATGCGATAACACTAACCATCTCCCCCAACATAATCAACTCAACCACCCCTTGACGAATTCAAACAACTCAAATCTCCCTGCCCCTAGCCCTATTTACTACAGCCGCCCTGGGATTACTACCAATAACCCCAACCATCCTAATACTAGCTACC

>Spinibarbus_hollandi

ATGAACCCCTACGTACTCACAATATTACTATCCAGCTTAGGGCTAGGAACCACCCTAACCTTCGCCAGCTCTCATTGACTCCTAGCTTGAATAGGCCTAGAAATCAACACCCTAGCAATCGCCCCTTTAATAACACAACATCACCACCCCCGCGCAGTAGAAGCAGCCACAAAATACTTCTTAACTCAAGCCACTGCAGCCGCAATAATCCTGTTCGCGAGTACAACAAACGCTTGAATAACAGGAGAATGATGCATTAATGATCTATCAAACCCTATTGCCAACACAATATTCATAACCGCTTTAGCACTTAAAATTGGACTCGCACCAATACACTTCTGAATACCCGAAGTATTACAAGGATTAGACCTGCTAACAGGCCTAATTCTATCCACCTGACAAAAACTTGCCCCATTTGCACTAATTATCCAAACGATACAAAACATTGACCCGTTACTACTAACACTTCTAGGGATCTTATCTACACTAGTAGGAGGATGGGGAGGACTAAACCAAACCCAACTACGAAAAATCCTAGCCTACTCCTCAATTGCCCACATAGGATGAATAATTATTGTAATCCAATACGCCCCCCAACTAACCTTGATTGCATTAGGAACATATATTATTATAACTTCCGCGGCATTCTTAACCCTAAAAATATCACTAGCAACAAAAATTAACACGCTCACAACAACCTGATCAAAAAGCCCCGTGTTAGCCTCAACAACCGCCCTAATTTTACTTTCACTAGGAGGCCTCCCACCCCTCACAGGGTTTATACCAAAATTAATAATTCTACAAGAATTAACAAAGCAAGACCTTCCCCTCATTGCCACAACCATGGCCTTAGCCGCACTAATTAGTCTATACTTTTACCTACGACTATGCTACGCAATAACATTAACCATCTCCCCTAACATAATCAACTCAACCACCCCCTGACGAATTCAAACAACCCAAACTTCCCTCCCCCTAGCCCTATTTATTATAGCCGCCCTAGGATTACTACCTATAACCCCGACCATCCTAATGCTAGCCACC

>Spinibarbus_sinensis

ATGAACCCATATGTACTTGCAATCTTATTATCTAGCCTGGGACTAGGAACCACCTTAACCTTCGCTAGCTCTCACTGACTCCTAGCTTGAATAGGCTTAGAAATTAATACACTAGCAATCGCCCCCCTAATAGCACAACACCACCACCCCCGCGCAGTAGAGGCAACTACAAAATACTTCTTAACCCAGGCCACCGCAGCAGCAATAATCCTGTTCGCAAGCACAACAAATGCCTGAATAACAGGAGAATGAAGCATCAACGATTTATCAAACCCTATCGCCAACACAATATTTATAGCCGCCCTAGCACTTAAAATTGGACTCGCACCAGTACACTTCTGAATACCAGAAGTCCTACAAGGATTAGACCTGCTAACAGGCCTTATCCTATCCACCTGACAAAAACTTGCCCCATTCGCACTAATTGTCCAAACAGCGCAAAACATCGACCCGCTACTGTTAACACTACTAGGGATTACATCCACATTAGTAGGAGGATGAGGAGGACTAAACCAAACCCAGCTACGAAAAATCCTAGCCTACTCCTCAATTGCCCACATAGGATGAATAATTATTGTAATCCAGTATGCCCCACAGCTTACCCTAATTGCACTAGGAACATATATTATCATGACCTCCGCAGCATTCCTGACCCTGAAAATATCACTAACAACAAAAATCAGCACACTCACAACAACCTGATCAAAGAACCCCGTACTGGCATCAACAACCGCCCTGGTCCTACTCTCACTAGGGGGCCTTCCACCACTCACAGGATTTATACCAAAATGAATAATTTTACAAGAACTAACAAAGCAAGACCTCCCCATTATCGCCACCGCCATAGCTCTAGCCGCACTAATCAGCCTATATTTCTACTTGCGATTATGCTACGCAATAACACTAACCGTCTCCCCAAACACAACCAACTCAACCACCCCCTGACGAACCCAAACAACCCAAACTTCCCTGCCCTTAGCCCTATCCATTACGGCCACCCTGGGACTATTACCAATAACCCCGACCATCCTAATACTAACCACC

**ND3**

>A._barbodon

ATGAATCTGATTATAGCCATTTTAATCACAGCAACAACTCTATCCTTAATTCTAGCAACTGTGTCTTTTTGACTACCACAAATAAACCCAGACGCAGAAAAACTTTCACCATATGAGTGCGGATTCGACCCACTAGGATCCGCCCGACTACCATTCTCACTACGCTTCTTCCTAGTAGCAATCCTATTCCTTCTCTTCGACCTGGAAATTGCTCTTCTCCTACCACTACCATGAGGAGATCAACTCCACAACCCAACAGGAACATTCTTCTGAGCTACAACAGTCTTAATTTTATTAACTCTAGGATTAATTTACGAATGAACCCAAGGTGGCTTAGAATGAGCAGAA

>A._beijiangensis_1

GTGAATCTAATTATAGCCATTTTAATCACCACAATAATTCTCTCCCTAATCCTAACAACTCTATCTTTTTGACTACCACAGATAAATCCAGACACAGAGAAACTTTCACCATACGAATGCGGATTCGACCCACTAGGGTCCGCCCGACTACCATTCTCCCTACGCTTCTTCCTAGTGGCAATCCTGTTCCTTCTCTTCGACCTGGAAATTGCCCTTCTCCTCCCCTTACCCTGAGGAGACCAGCTCCAAAACCCAACAGGAACATTTTTCTGAGCTACTATAATCTTAATTCTATTAACCCTAGGATTAGTCTATGAATGAACCCAAGGCGGCTTAGAATGAGCAGAA

>A._beijiangensis_2

GTGAATCTAATTATAGCCATTTTAATCACCACAATAATTCTCTCCCTAATCCTAACAACTCTATCTTTTTGACTACCACAGATAAATCCAGACGCAGAGAAACTCTCACCATACGAATGCGGATTCGACCCACTAGGATCCGCCCGACTACCATTCTCCCTACGCTTCTTCCTAGTAGCAATCCTGTTCCTTCTCTTCGACCTGGAAATTGCCCTTCTCCTCCCCTTACCCTGAGGAGACCAGCTCCACAGCCCAACAGGAACATTTTTCTGAGCTACTATAGTCTTAATTCTATTAACCCTAGGACTAGTCTATGAATGAACCCAAGGCGGCTTAGAATGAGCAGAA

>A._fasciatus

ATGAACCTAGTTATAACCATTCTAACCATCGCAATAATCTTATCCTTAGTTCTGGCAACTGTATCTTTTTGATTGCCGCAAATAAACCCAGATGCAGAAAAACTATCCCCATACGAGTGTGGATTCGACCCATTAGGATCCGCCCGACTACCATTCTCTTTACGCTTCTTCCTGGTAGCAATCCTATTCCTTCTCTTCGACCTAGAAATTGCCCTTCTCCTTCCCCTGCCCTGAGGAGACCAACTCCATAACCCAACAGGAACATTCTTCTGAGCTACAACAGTCTTAATCTTATTAACCCTTGGGCTAGTCTATGAATGAACCCAAGGCGGCTTAGAATGAGCAGAA

>A._hemispinus

ATGAACCTGGTTATAGCCATTCTAACCATTGCAATAATCTTATCCTTAATTTTAGCAACTGTATCTTTTTGATTACCACAAATAAACCCGGATGCAGAGAAATTATCCCCATACGAATGCGGATTTGACCCATTAGGGTCCGCCCGACTACCATTCTCCCTACGCTTTTTCCTAGTAGCAATCCTGTTCCTCCTCTTCGACCTAGAAATTGCTCTCCTCCTCCCCCTACCCTGAGGAGACCAACTCCATAACCCAACAGGAACATTCTTCTGAGCCACAACAGTCTTAATTTTACTAACCCTGGGATTAATTTATGAATGAACCCAAGGCGGCTTAGAATGAGCAGAA

>A._iridescens

ATGAATCTGATTATAGCCATTTTAATTACAGCAACAGCTCTATCCTTAATTCTAGCAACTGTATCTTTTTGACTACCACAAATAAACCCAGACGCAGAAAAACTTTCACCATATGAGTGCGGATTCGATCCACTAGGGTCCGCCCGGCTACCATTCTCATTACGCTTCTTCCTAGTAGCAATCCTATTCCTTCTCTTCGACCTGGAAATTGCTCTTCTCCTACCACTACCATGAGGAGATCAGCTCCACAACCCAACAGGAACATTCTTCTGAGCTACAACAGTCTTAATTTTATTAACTCTAGGATTAATTTACGAATGAACCCAAGGTGGCTTAGAGTGAGCAGAA

>A._jishouensis

GTGAACCTAGTTATAGCCATTCTAACCATCGCAATAATCTTATCCTTAATCCTAGCAACTCTATCTTTTTGATTACCACAAATAAACCCAGATGCAGAAAAACTGTCCCCATACGAATGCGGGTTTGACCCATTAGGGTCGGCCCGACTGCCATTCTCTCTACGCTTCTTCCTAGTAGCAATCCTGTTCCTTCTCTTCGACCTAGAAATTGCTCTCCTCCTCCCCCTGCCCTGAGGAGACCAACTCCATAACCCAACAGGAACATTCTTCTGGGCCACAACAGTCCTAATTTTACTAACCCTTGGATTAATTTATGAGTGAACCCAAGGCGGCTTAGAATGAGCAGAA

>A._kreyenbergii_1

ATGAACCTAGTCATAACTATTCTAACCATCGCAATAATCTTATCCTTAATCCTAGCAACTGTATCATTTTGATTACCACAAATAAACCCAGATGCAGAAAAATTGTCCCCATACGAATGCGGGTTTGACCCGTTAGGATCCGCCCGACTGCCGTTCTCTTTACGCTTCTTCCTAGTAGCAATCCTGTTCCTTCTCTTCGACCTAGAAATTGCCCTTCTCCTTCCCCTGCCCTGAGGAAACCAACTCCATAACCCAACAGGAACATTCTTCTGAGCTACAGCAGTCTTAATTCTATTAACCCTTGGACTAGTCTATGAATGAACTCAAGGCGGTTTAGAATGGGCAGAA

>A._kreyenbergii_2

ATGAACCTAGTTATAACTATTCTAACCATCGCAATAATCTTATCCTTAGTCCTAGCAACTGTATCATTTTGATTGCCACAAATAAATCCAGATGCAGAAAAACTATCCCCATACGAATGCGGATTTGACCCATTAGGTTCCGCCCGACTACCATTCTCTTTACGCTTCTTCCTAGTAGCAATCCTGTTCCTTCTCTTCGACCTAGAGATTGCTCTTCTCCTCCCCCTGCCCTGAGGGGACCAACTCCATAACCCAACAGGAACATTCTTCTGGGCTACAGCAGTCTTAATTCTATTAACCCTTGGACTAGTCTATGAGTGAACTCAAGGCGGTTTAGAATGGGCAGAA

>A._longipinnis

ATGAATCTGATTATAGCCATTTTAATTACAGCAACAGCTCTATCCTTAATTCTAGCAACTGTATCTTTTTGACTACCACAAATAAACCCAGACGCAGAAAAACTTTCACCATATGAGTGCGGATTCGATCCACTAGGGTCCGCCCGGCTACCATTCTCATTACGCTTCTTCCTAGTAGCAATCCTATTCCTTCTCTTCGACCTGGAAATTGCTCTTCTCCTACCACTACCATGAGGAGATCAGCTCCACAACCCAACAGGAACATTCTTCTGAGCTACAACAGTCTTAATTTTATTAACTCTAGGATTAATTTACGAATGAACCCAAGGTGGCTTAGAGTGAGCAGAA

>A._monticola_1

GTGAACCTAATTACAGCCATTTTAATTATTACAATAGCCCTGTCCCTAATTCTAGCAGTTGTTTCTTTTTGACTACCACAAATAAACCCAGACGCAGAAAAACTATCACCATACGAATGTGGATTTGACCCACTAGGATCCGCCCGACTACCATTTTCCTTACGTTTCTTCCTAGTGGCAATTCTATTCCTTCTCTTCGACCTAGAAATTGCTCTCCTCCTCCCACTACCTTGAGGTGACCAACTCCAAAATCCCACAGGAACATTTTTCTGAGCCACAACAGTCCTAATCCTATTAACCCTCGGATTAATTTATGAATGAACCCAAGGTGGCTTAGAATGAGCAGAA

>A._monticola_2

GTGAACCTAATTACAGCCATTTTAATTATTACAATAGCCCTGTCCCTAATTCTAGCAGTTGTTTCTTTTTGACTACCACAAATAAACCCAGACGCAGAAAAACTATCACCATACGAATGTGGATTTGACCCACTAGGATCCGCCCGACTACCATTTTCCTTACGTTTCTTCCTAGTGGCAATTCTATTCCTTCTCTTCGACCTAGAAATTGCTCTCCTCCTCCCACTACCTTGAGGTGACCAACTCCAAAATCCCACAGGAACATTTTTCTGAGCCACAACAGTCCTAATCCTATTAACCCTCGGATTAATTTATGAATGAACCCAAGGTGGCTTAGAATGAGCAGAA

>A._paradoxus_1

GTGAACCTTATTATAGCCATTTTAACCATCGCAATAGCCCTATCCCTAATCCTAGCAACTGTGTCTTTTTGACTACCACAAATAAATCCAGACGCAGAAAAACTATCACCATACGAGTGCGGATTCGACCCGCTAGGATCCGCCCGACTACCATTCTCCTTGCGATTCTTCCTAGTAGCAATCCTATTCCTTCTCTTCGACCTAGAAATTGCTCTCCTCCTTCCTTTACCCTGAGGAGACCAGCTCGACAGCCCAACAGGAACATTTTTCTGAGCTACTACAGTCTTAATTCTATTAACCCTTGGATTAATTTATGAATGAACCCAAGGCGGCTTAGAATGAGCAGAA

>A._paradoxus_2

GTGAACCTAGTTATAGCCATTCTAACCATCGCAATAATCTTATCCTTAATCCTAGCAACTCTATCTTTTTGATTACCACAAATAAACCCAGATGCAGAAAAACTATCCCCATACGAATGCGGGTTTGACCCATTAGGATCGGCCCGACTGCCATTCTCTCTACGCTTCTTCCTAGTAGCAATCCTATTCCTTCTCTTCGACCTAGAAATTGCTCTCCTCCTCCCCCTGCCCTGAGGAGACCAACTCCATAACCCAACAGGAACATTCTTCTGGGCCACAACAGTCCTAATTTTACTAACCCTTGGATTAATTTATGAGTGAACCCAAGGCGGCTTAGAATGAGCAGAA

>A._parallens_1

ATGAACCTAGTTATAGCCATTCTAACCATTGCAATAATCTTATCCTTAATCTTAGCAACTGTATCTTTTTGATTACCACAAATAAACCCGGATGCAGAAAAATTATCCCCATACGAATGCGGATTTGACCCATTAGGGTCCGCCCGGCTACCATTCTCCCTACGCTTTTTCCTAGTAGCAATCCTGTTCCTCCTCTTCGACCTAGAAATTGCTCTCCTCCTCCCCCTACCCTGAGGAGACCAACTCCATAACCCAACAGGAACATTCTTCTGAGCCACAACAGTCTTAATTTTACTAACCCTAGGGTTAATTTATGAATGAACCCAAGGCGGCTTAGAGTGAGCAGAA

>A._parallens_2

GTGAACCTAGTTATAGCCATTCTAACCATTGCAATAATCTTATCCTTAATCTTAGCAACTGTATCTTTTTGATTACCACAAATAAACCCGGATGCAGAAAAATTATCCCCATACGAATGCGGATTTGACCCATTAGGGTCCGCCCGGCTACCATTCTCCCTACGCTTTTTTCTAGTAGCAATCCTGTTCCTCCTCTTCGACCTAGAAATTGCTCTCCTCCTCCCCCTACCCTGAGGAGACCAACTCCATAACCCAACAGGAACATTCTTCTGAGCCACAACAGTCTTAATTTTACTAACCCTAGGATTAATTTATGAGTGAACCCAAGGCGGCTTAGAATGAGCAGAA

>A._parallens_3

ATGAACCTAGTCATAGCCATTCTAACCATTGCAATAATCTTATCCTTAATCTTAGCAACTGTATCTTTTTGATTACCACAAATAAACCCGGATGCAGAAAAATTATCCCCATACGAATGCGGATTTGACCCATTAGGGTCCGCCCGGCTACCATTCTCCCTACGCTTTTTCCTAGTAGCAATCCTGTTCCTCCTCTTCGACCTAGAAATTGCCCTCCTCCTCCCCCTACCCTGGGGAGACCAACTCCATAACCCAACAGGAACATTCTTCTGAGCCACAACAGTCTTAATTTTACTAACCCTGGGGCTAATTTATGAGTGAACCCAAGGCGGCTTAGAATGAGCAGAA

>A._parallens_4

ATGAACCTAGTTATAGCCATTCTAACCATTGCAATAATCTTATCCTTAATCTTAGCAACTGTATCTTTTTGATTACCACAAATAAACCCGGATGCAGAAAAATTATCCCCATACGAATGCGGATTTGACCCATTAGGGTCCGCCCGGCTACCATTCTCCCTACGCTTTTTCCTAGTAGCAATCCTGTTCCTCCTCTTCGACCTAGAAATTGCTCTCCTCCTCCCCCTACCCTGGGGAGACCAACTCCATAACCCAACAGGAACATTCTTCTGAGCCACAACAGTCTTAATTTTACTAACCCTGGGGCTAATTTATGAATGAACCCAAGGCGGCTTAGAATGAGCAGAA

>A._spinifer

GTGAATCTAATTATAGCCATTTTAACCACCACAGTAATTCTATCCCTAATCCTAACAACTCTATCTTTTTGATTACCACAGATAAACCCAGACGCAGAGAAACTTTCACCATACGAGTGCGGATTCGACCCACTAGGATCCGCCCGACTACCATTCTCCCTACGCTTTTTCCTAGTAGCAATCCTGTTCCTTCTCTTCGACCTGGAAATTGCTCTTCTCCTCCCCCTACCCTGAGGAGACCAGCTCCACAACCCAACAGGAACATTTTTCTGAGCTACTATAGTCCTAATTCTATTAACCCTAGGATTAGTCTATGAATGAGCCCAAGGCGGCTTAGAATGAGCAGAA

>A._stenotaeniatus

GTGAATCTAATTATAGCCATTTTAACCACCATAGTAATTCTCTCCCTAATCCTAACAACTCTATCTTTTTGATTACCACAGATAAACCCAGACGCAGAGAAACTTTCACCATACGAATGCGGATTCGACCCACTAGGATCCGCCCGACTACCATTCTCCCTACGCTTTTTCCTAGTAGCAATCCTGTTCCTTCTCTTCGACCTAGAAATTGCTCTTCTCCTCCCCTTACCCTGAGGAGACCAGCTCCACAACCCAACAGGAACATTTTTCTGAGCTACTACAGTCCTAATTCTATTAACCCTAGGATTAGTCTATGAATGAGCCCAAGGCGGCTTAGAATGAGCAGAA

>A._wenchowensis_1

ATGAACTTAGTTATAACCATTCTAACCATCGCAATAATCTTATCCTTAGTCCTAGCAACTGTATCTTTTTGACTACCGCAAATAAACCCAGATGCAGAAAAACTGTCCCCATACGAATGCGGGTTTGACCCATTAGGATCCGCCCGACTACCATTCTCTTTACGCTTCTTCCTAGTAGCAATCCTGTTCCTTCTCTTCGACCTAGAAATTGCCCTTCTCCTTCCCCTACCCTGAGGAGACCAACTCCATAATCCAACAGGAACATTCTTCTGAGCTACAACAGTCTTAATCTTATTAACCCTTGGGCTAGTCTATGAATGAACCCAAGGCGGCTTAGAATGAGCAGAG

>A._wenchowensis_2

ATGAACCTAGTTATAACCATTCTAACCATCGCAATAATCTTATCCCTAGTCCTAGCAACTGTATCTTTTTGATTACCACAAATAAACCCAGATGCAGAAAAACTATCCCCATACGAATGCGGGTTTGACCCATTGGGATCCGCCCGACTACCATTCTCTTTACGCTTCTTCCTGGTAGCAATCCTATTCCTTCTCTTCGACCTAGAAATTGCCCTTCTCCTTCCCCTACCCTGAGGAGACCAACTCCATAACCCAACAGGAACATTCTTCTGAGCTACAACAGTCTTAATCTTATTAACCCTTGGGCTAGTCTATGAATGAACCCAAGGCGGCTTAGAATGAGCAGAA

>A._wuyiensis

GTGAATCTAATTATAGCCATTTTAACCATCGCAATAGCCCTATCCCTAATCCTAGCAACTGTATCTTTTTGATTACCGCAAATAAATCCAGACGCAGAAAAACTATCACCATACGAGTGCGGATTCGACCCGCTAGGATCCGCCCGACTACCATTCTCCCTGCGATTCTTCCTAGTAGCAATCCTATTCCTTCTCTTTGACCTAGAGATTGCTCTCCTCCTTCCTTTACCCTGAGGAGACCAGCTCGACAGCCCAACAGGAACATTTTTCTGAGCTACTACAGTCTTAATTCTATTAACCCTTGGATTAATTTATGAATGAACCCAAGGCGGCTTAGAATGAGCAGAA

>A._yunnanensis_1

GTGAATCTAATTATAGCCATTTTAATTATTACAACAGCCCTGTCCCTAATTTTAGCAATTGTCTCTTTTTGACTACCACAAATAAACCCAGATGCAGAAAAACTATCACCATACGAATGTGGATTTGACCCACTAGGGTCCGCCCGGCTACCATTCTCTCTGCGCTTCTTCCTAGTGGCAATCTTATTTCTTCTCTTTGACCTAGAAATTGCTCTCCTACTCCCTCTACCCTGAGGTGATCAGCTCCAAAATCCCACAGGCACATTCTTCTGAGCTACAACAGTCCTAATCTTACTAACCCTGGGATTAATTTATGAATGAACTCAGGGCGGCCTAGAATGGGCAGAA

>A._yunnanensis_2

GTGAACCTAATTATAGCCATTTTAATTATTACAACAGCCCTGTCCCTAATTTTAGCAATTGTCTCTTTTTGACTACCACAAATAAATCCAGATGCAGAAAAACTATCACCATACGAATGTGGATTTGACCCACTAGGGTCCGCCCGACTACCATTCTCTCTGCGCTTCTTCCTAGTGGCAATCTTATTTCTTCTCTTCGACCTAGAAATTGCTCTCCTACTCCCTCTACCCTGAGGTGATCAGCTCCAAAATCCCACAGGCACATTCTTCTGAGCCACAACAGTCCTAATCTTACTAACCCTGGGATTAATTTATGAATGAACTCAGGGCGGCCTAGAATGGGCAGAA

>Onychostoma_barbatulum

ATGAATTTAATTATAACCATTTTAATTATTACAATAGCCCTCTCCTTAATTCTGGCAACTATCTCTTTTTGACTGCCACAGATAAATCCAGACACAGAAAAACTTTCACCATATGAATGTGGATTTGACCCACTAGGATCTGCCCGACTACCATTTTCCCTGCGCTTCTTCCTAGTAGCAATTCTATTCCTCCTTTTCGACCTAGAAATTGCTCTCCTCCTCCCACTACCCTGAGGAGACCAACTCCACAACCCAACAGGAACACTCTTCTGAGCTACCTCAGTCCTAATCTTACTAACCCTAGGATTAATTTATGAGTGAACTCAAGGCGGCTTAGAATGAGCAGAA

>Onychostoma_meridionale

GTGAACCTAATTACAACCATTTTAATTATTACCATAGCCCTCTCCTTAATTCTAGCGGCTGTATCTTTTTGACTACCGCAGATAAACCCAGACACAGAAAAACTATCACCATATGAATGCGGATTTGACCCACTGGGATCCGCTCGACTGCCATTCTCTTTACGCTTCTTTCTAGTAGCAATTCTATTCCTCCTCTTCGACCTAGAAATCGCCCTCCTCCTTCCACTGCCCTGAGGAGATCAACTCCACAACCCAACAGGAACATTCTTCTGAGCTACCACAGTCCTAATCTTATTAACACTAGGATTAATTTATGAGTGAACCCAAGGCGGCCTAGAATGGGCAGAA

>Onychostoma_gerlachi

GTGAACCTAATTATAGTCATTTTAATAATTACGATGGCCCTACTCTTAATTCTAGCAGCCGTATCTTTTTGATTACCGCAGATAAACCCAGACACAGAAAAACTATCCCCCTACGAATGCGGATTTGACCCACTAGGATCCGCTCGTCTACCATTCTCCTTGCGCTTCTTTCTAGTAGCAATCCTATTCCTCCTCTTCGACCTAGAGATCGCCCTCCTCCTCCCATTGCCCTGAGGAGACCAGCTCCACAACCCGACAGGAACATTCTTCTGAGCTACCACAGTCCTAATCTTATTAACCCTAGGACTAATTTATGAATGAACCCAAGGCGGCCTAGAATGGGCAGAA

>Spinibarbus_denticulatus

ATGAATATTATTATAACCATTTTAATTATTACAGTAGCCTTATCCCTAATTTTAGCTACTGTATCTTTTTGACTACCACAAATAAACCCAGACGCAGAGAAACTATCACCATACGAGTGCGGGTTTGACCCACTAGGATCCGCCCGACTACCATTCTCCTTACGCTTCTTCCTGGTAGCAATCCTATTTCTCCTGTTTGATCTAGAAATTGCCCTTCTTCTCCCACTACCTTGAGGAGACCAACTCTACAACCCCACCAAAACATTCTTCTGAGCCACAACCGTCCTAATTTTATTAACACTAGGACTAATCTATGAATGAACTCAAGGTGGCTTAGAATGAGCAGAA

>Spinibarbus_hollandi

ATGAATATTATTATAGCCATTTTAATCATTACAGTAACCTTATCCTTGATTTTAGCTACTGTATCCTTTTGATTACCACAAATAAACCCAGACGCAGAGAAACTATCACCATACGAGTGCGGATTTGACCCGCTAGGATCCGCCCGATTACCATTCTCCTTACGCTTCTTCCTGGTAGCAATCCTGTTCCTCCTATTTGATCTAGAAATTGCCCTTCTCCTTCCATTACCCTGAGGAGATCAACTCTATAACCCCGCCGGGACATTCTTCTGGGCTACAACAGTCCTAATTTTATTAACATTAGGACTAATCTATGAATGAACTCAAGGCGGCTTAGAATGGGCAGAA

>Spinibarbus_sinensis

ATGAATCTGATTATAACCATTTTAATTATCACAATAGCCCTGTCCTCAATCCTAGCAATTGTATCTTTTTGATTACCACAAATAAACCCCGATGCAGAAAAACTATCACCATACGAATGTGGCTTTGACCCACTAGGATCTGCCCGATTACCATTCTCCCTGCGCTTCTTCCTAGTAGCAATCCTATTTCTACTCTTCGACCTAGAAATTGCCCTCCTCCTCCCACTACCCTGGGGAGATCAACTCCACAACCCCACTGGAACATTCTTCTGAGCCACAACAGTCCTAATTTTATTAACCCTAGGACTAATCTATGAATGAACTCAAGGCGGTTTAGAATGAGCAGAA

**ND4**

>A._barbodon

ATGCTAAAAGTACTAATTCCCACAATTATGCTATTCCCAACAATTTGACTAACTTCCCCTAAATGGCTATGAACAACCACGACCACACACAGCCTCCTAATTGCTCTCACCAGCCTAACATGACTAAAATGAACATCCGAAACCGGATGAACCTCCTCCAACACATATATAGCCACAGACCCACTATCAACCCCCCTCCTAGTACTAACATGCTGACTACTCCCACTCATGATTCTAGCCAGCCAAAACCACATCAGCCCTGAACCGATTAGCCGACAACGCACGTACATCATACTCCTCGCCTCACTACAAACTTTTTTAATCTTAGCATTCAGTGCCACAGAAGTCATTATATTCTATATTATATTTGAAGCTACACTTATCCCAACCCTTATCATCATTACCCGGTGAGGAAACCAAACTGAACGGCTTAATGCAGGAACCTATTTTCTGTTTTATACCCTAGCAGGGTCACTCCCACTTCTAGTAGCCTTACTCCTTCTTCAACAATCCACTGGCACCCTATCCATGCTAGTGCTTCAATATTCACAACCCCTACAACTCAACCCCTGAGGCCACACAATTTGATGAGCTGGCTGCCTAGTCGCATTTTTAGTTAAAATACCCCTATATGGAGTCCACCTCTGACTACCAAAAGCACATGTAGAAGCTCCCGTAGCAGGATCTATAGTACTAGCAGCAGTTCTACTAAAACTCGGCGGGTACGGAATAATGCGCATAATAGTAATACTTGACCCCCTGTCAAAAGAGCTAGCCTACCCCTTCATTATCTTAGCCCTATGAGGCATTATTATAACCGGATCAATCTGCCTCCGACAAACAGACCTAAAATCACTTATTGCCTACTCATCCGTCGGCCACATGGGGCTAGTGGCAGGAGGAATTCTAATTCAAACTCCATGAGGCTTCTCAGGAGCAATCATTCTAATAATTGCCCACGGCCTAGCATCCTCAGCACTATTCTGCCTAGCCAACACAGCATATGAACGAACCCACAGCCGAACAATAGTCCTTGCCCGAGGGTTACAAGTAATTTTCCCGTTAACAGCAGTCTGATGATTCATCGCCAACCTGGCCAACCTAGCACTACCACCACTACCAAACCTCATGGGAGAACTTATAATCATCACAACATTGTTTAACTGATCTCCCTGAACAATCGTTCTTACCGGGCTGGGAACACTAATTACAGCCAGCTACTCCCTATATATATTCCTCATGTCACAACGAGGCCCAACACCAAACCACATCACAGGACTCCAACCATTCCACACCCGAGAACACTTACTAATAACCATACACTTAATCCCCATTATCCTACTAGTAACAAAGCCAGAACTCATATGAGGATGATGCTAC

>A._beijiangensis_1

ATGTTAAAAGTACTAATCCCCACAATCATATTATTTCCAACAATTTGACTAACTTCCCCTAAATGACTATGAACAACCACAACTGCACACAGTCTCCTAATCGCCCTTATTAGCTTAACCTGACTAAAATGAACATCCGAAACCGGATGGAACTCCTCTAACACATACCTAGCCACAGACCCATTATCAACCCCCCTCCTAGTGCTAACATGCTGATTACTCCCACTTATAATTTTAGCTAGTCAAAACCACATCAGCCCTGAACCAACCACCCGACAACGCACATATATTACTCTCCTTGCCTCGCTACAAACTTTTTTAATCATAGCATTCGGCGCTACAGAGGTCATTATATTTTATATTATATTTGAAGCTACACTTATCCCAACCCTCATTATTATTACCCGATGAGGAAATCAAACTGAACGACTCAATGCCGGAACCTATTTTCTATTCTATACCCTAGCAGGATCACTCCCACTTCTAGTTGCCCTACTCCTTCTCCAACAATCCACCGGCACCCTATCAATACTAGTGCTTCAATACTCACAACCCTTGCAGCTCAACTCTTGAGGCCACATAATCTGATGAGCTGGCTGTCTGATCGCATTCCTAGTTAAAATACCACTATATGGGGTTCACCTATGACTACCAAAGGCGCACGTAGAAGCCCCCGTAGCAGGATCCATAGTGCTAGCAGCAGTTTTATTAAAACTCGGCGGATACGGAATAATACGCATAATAGTAATGCTCGACCCACTGTCAAAAGAACTAGCCTACCCATTTATTATTTTAGCCCTATGAGGCATCATTATAACTGGATCAATCTGCCTTCGACAGACAGACTTAAAGTCATTAATTGCCTACTCATCCGTAGGCCATATAGGACTAGTAGCAGGAGGAATCCTAATTCAAACCCCATGAGGCTTCTCAGGAGCAATCATCCTAATAATTGCCCATGGATTAGCATCCTCAGCATTATTCTGTCTGGCCAACACAGCATATGAACGAACTCACAGCCGAACAATAGTCCTTGCCCGAGGGCTTCAAGTGATTTTTCCACTAACAGCAGTCTGATGATTCATCGCTAATCTGGCTAACCTGGCGCTACCACCACTACCAAACCTTATAGGAGAACTTATAATCATTACAACATTATTCAACTGATCCCCCTGAACAATTCTACTTACCGGACTAGGAACATTAATTACAGCCAGCTATTCCTTATACATATTCCTAATATCACAACGAGGCCCAGCACCAAACCATATTACAGGACTTCAACCATTCCACACCCGAGAACATCTACTAATAACCCTACACTTAATCCCCATTATTCTACTAGTAACAAAACCAGAACTTATATGAGGATGATGCTAC

>A._beijiangensis_2

ATGTTAAAAGTACTAATCCCCACAATCATATTATTCCCAACAATTTGACTAACTTCCCCTAAATGACTATGAACAACCACAACTGCACACAGTCTCCTAATCGCTCTTATTAGCCTAACCTGACTAAAATGAACATCCGAAACCGGATGGACCTCCTCTAACACATACCTAGCCACAGACCCGTTATCAACCCCCCTCCTAGTACTAACATGCTGATTACTCCCACTTATAATTTTAGCTAGTCAAAACCACATCAGCCCTGAACCAACCACCCGACAACGCACATATATTACCCTCCTTACCTCGCTACAAACTTTTTTGATCATAGCATTCGGCGCTACAGAGGTCATTATATTTTATATCATATTTGAAGCTACACTCATCCCAACCCTCATTATTATTACCCGATGAGGAAATCAAACTGAACGGCTCAATGCCGGAACCTATTTTCTATTCTATACCCTGGCAGGATCACTCCCGCTTCTAGTTGCCCTACTCCTTCTCCAACAATCCACCGGCACCTTATCAATACTAGTGCTTCAATACTCACAGCCCTTGCAGCTCAACTCTTGAGGCCACACAATCTGATGGGCTGGCTGTCTGATCGCATTCCTAGTTAAAATACCACTATATGGAGTTCACCTATGACTACCAAAGGCGCACGTAGAGGCCCCCGTAGCAGGATCCATAGTACTAGCAGCGGTTTTATTAAAACTCGGCGGATATGGAATGATACGCATAATAGTAATGCTCGACCCACTGTCAAAAGAACTAGCCTACCCATTTATTATTTTAGCCCTATGAGGCATCATTATAACTGGGTCAATCTGCCTCCGACAAACAGACTTAAAGTCATTAATTGCCTACTCATCCGTAGGCCATATAGGACTAGTAGCAGGAGGAATCCTAATTCAAACCCCATGAGGCTTCTCAGGAGCAATCATCCTGATAATTGCCCATGGACTAGCATCCTCAGCACTATTCTGTCTGGCCAACACAGCATATGAACGAACTCACAGCCGAACAATAGTCCTTGCCCGAGGACTTCAAGTGATTTTTCCACTAACAACAGTCTGATGACTCATCGCTAATCTGGCTAACCTGGCGCTACCACCACTACCAAACCTTATAGGAGAGCTTATAATCATTACAACATTATTCAACTGATCCCCCTGAACAATTCTACTCACCGGACTAGGAACATTAATTACAGCCAGCTATTCCTTATATATATTCCTAATATCACAACGAGGCCCAACACCAAACCATATTACAGGACTCCAACCATTCCACACCCGAGAGCACCTACTAATAACCCTACACTTAATCCCCATTATCCTACTAGTGACAAAACCAGAACTTATATGAGGATGATGCTAC

>A._fasciatus

ATGCTAAAAGTACTTGTCCCCACAATTATACTATTTCCAACAATTTGACTAACTTCCCCCAAATGACTATGAACAACTACAACCACACATAGCCTCCTAATTGCTCTTATTAGCCTAACTTGACTAAAATGAACATCCGAAACCGGATGGACCTCCTCCAATATATACCTAGCCACAGACCCCTTATCAACCCCCCTCCTAGTACTAACATGCTGATTACTCCCACTCATAATTCTAGCCAGCCAAAACCACATCAGCCCCGAACCAATTGCTCGACAGCGCACATACATTATGCTCCTCACCTCATTACAAACCTTTTTAATTATGGCATTCGGCGCTACAGAAGTCATTATATTTTACATCATATTCGAAGCCACACTTATTCCAACTCTTATTATTATTACCCGTTGGGGGAATCAGACTGAACGACTCAATGCGGGAACTTATTTTCTGTTCTACACCCTGGCAGGATCACTACCCCTCCTAGTTGCCCTGCTCCTTCTCCAACAATCCACTGGTACCCTGTCAATATTAGTGCTTCAATACTCACAACCCCTACAACTCAACTCCTGAGGCCACACAATCTGATGAGCTGGCTGCCTGATTGCATTTCTAGTCAAAATACCACTATACGGAGTTCATCTGTGGCTGCCAAAAGCGCACGTAGAAGCCCCCGTGGCAGGATCTATAGTCCTAGCAGCAGTTTTACTAAAACTTGGTGGATATGGAATAATACGAATGATAGTAATGCTTGACCCTTTATCAAAAGAGCTGGCCTATCCATTTATTATCCTAGCCCTATGAGGCATCATTATAACTGGATCAATCTGCCTCCGACAAACGGACCTAAAATCATTAATCGCCTATTCATCCGTGGGCCATATGGGATTAGTGGCAGGGGGAATCCTAATTCAAACCCCATGAGGCTTCTCAGGAGCAATCATTCTTATAATTGCCCACGGATTAGCATCTTCAGCATTATTCTGCTTAGCCAACACAGCGTATGAACGAACCCACAGTCGAACAATGGTACTTGCCCGAGGACTACAAGTGATCTTTCCATTAACAGCAGTCTGATGATTTATAGCTAATCTAGCCAACCTAGCACTACCACCACTACCAAACCTTATAGGGGAACTCATAATCATCACAACATTATTCAACTGATCCCCCTGGACAATCCTACTCACCGGGCTAGGGACACTAATTACAGCCAGCTACTCCCTATACATATTCCTTATATCACAACGAGGCCCAACACCAAACCACATCTCAGGACTCCAACCGTTCCACACCCGAGAACATTTACTGATAATCATACACTTAATCCCTATTATCCTACTAGTGACAAAACCAGAGCTTATGTGAGGATGATGCTAC

>A._hemispinus

ATGCTAAAGGTACTAATCCCTACAATTATACTATTCCCAACAATCTGACTAACTTCCCCTAAATGACTATGAACAACTACAACCGCACATAGCCTCCTAATTGCTCTTACCAGCTTAACTTGACTAAAGTGAACATCCGAAACCGGATGAGCCTCCTCCAATACATACCTAGCCACAGACCCCTTATCAACCCCCCTCCTAGTACTAACATGCTGATTACTCCCACTCATAATTCTAGCCAGCCAAAACCACATCAGCCCCGAACCGATTGCCCGACAACGCACATACATTATACTTCTCGCTTCATTACAAACCTTCTTAATCATAGCATTTGGCGCTACAGAAGTCATTATATTTTACATCATATTTGAAGCCACACTTATTCCGACTCTTATCATCATCACCCGATGGGGAAATCAAACTGAACGACTTAATGCAGGAACCTATTTTCTGTTCTACACCCTAGCGGGATCACTCCCGCTTCTCGTCGCCCTCCTCCTCCTCCAACAATCCACCGGCACCCTCTCAATATTAGTACTTCAATACTCACAACCCCTACAACTCAACACCTGAGGCCACATGGTCTGATGAGCCGGCTGCCTAATCGCATTCCTAGTCAAAATACCCCTATATGGAGTCCATCTATGACTACCAAAAGCGCACGTAGAAGCCCCCGTGGCAGGATCTATGGTACTAGCAGCAGTTTTACTAAAGCTCGGCGGATACGGAATAATACGCATAATAGTAATGCTCGACCCCTTATCAAAAGAACTAGCTTATCCATTTATTATCCTGGCCCTATGGGGCATCATTATAACTGGATCAATCTGCCTCCGACAAACAGACCTAAAGTCATTAATTGCCTACTCATCCGTAGGCCATATAGGATTGGTGGCAGGAGGAATCCTAGTTCAAACCCCATGGGGCTTCTCAGGAGCAATCATCCTTATAATTGCCCACGGATTAGCATCTTCAGCATTATTCTGTCTAGCTAACACAGCTTATGAACGAACCCATAGTCGAACAATAGTCCTTGCCCGAGGATTACAAGTGATCTTTCCACTAACAGCAGTCTGATGATTTACCGCTAATCTAGCCAACTTAGCACTACCACCACTACCAAACCTTATGGGGGAACTCATAATCATCACAACATTATTTAACTGATCCCCCTGAACAATCTTACTCACTGGACTAGGAACACTAATTACAGCCAGCTACTCTCTATATATGTTCCTCATATCGCAACGGGGCCCAACACCAAACCACATCACAGGACTTCAACCATTTCATACCCGAGAACATTTACTAATAACCCTGCACTTAATTCCTATCATCCTGCTAGTAACAAAACCAGAGCTTATATGAGGATGATGTTAC

>A._iridescens

ATGCTAAAAGTACTAATTCCCACAATTATGCTATTCCCAACAATTTGACTAACTTCCCCTAAATGGCTATGAACAACCACAACCGCACACAGCCTCCTAATTGCTCTCACCAGCCTAACATGACTAAAATGAACATCCGAAACCGGGTGAACCTCCTCCAACACATATATAGCCACAGACCCACTATCAACCCCTCTCCTAGTACTAACATGCTGACTACTCCCACTCATAATTCTAGCCAGCCAAAACCACATCAGCCCTGAACCTATTAGCCGACAACGCACGTACATCATACTCCTCACCTCACTACAGACTTTTTTAATCTTAGCATTCGGTGCCACAGAAGTCATTATATTCTATATTATATTTGAAGCTACACTTATCCCAACCCTTATTATCATTACCCGGTGAGGAAACCAAACTGAACGACTCAATGCAGGAACCTATTTTCTGTTTTACACCCTAGCAGGGTCACTCCCACTTCTAGTAGCCTTACTCCTTCTTCAACAATCCACTGGCACCCTATCCATGCTAGTGCTTCAATATTCACAGCCCCTACAACTCAACTCCTGAGGCCACACAATTTGATGAGCTGGCTGCCTAGTCGCATTTTTAGTTAAAATACCTCTATATGGAGTCCACCTATGATTACCAAAAGCACATGTAGAAGCCCCCGTAGCAGGATCTATAGTACTAGCAGCAGTTCTACTAAAACTCGGCGGGTACGGAATAATGCGCATAATAGTTATACTTGACCCCCTGTCAAAAGAGCTGGCCTACCCCTTCATTATCTTAGCCCTATGAGGCATTATTATAACCGGATCAATTTGCCTCCGACAAACAGACCTAAAATCACTTATTGCCTACTCATCCGTCGGCCACATGGGGCTAGTGGCAGGAGGAATTCTAATTCAAACTCCATGAGGCTTCTCAGGAGCAATCATTCTAATAATCGCCCACGGCCTAGCATCCTCAGCACTGTTCTGCCTAGCCAACACAGCATATGAACGAACCCACAGCCGAACAATAGTCCTTGCCCGAGGATTACAAGTAATTTTCCCGTTAACAGCAGTCTGATGATTCATCGCCAACCTGGCCAACCTAGCACTACCACCACTACCAAACCTCATGGGAGAACTTATAATCATCACAACATTATTTAACTGATCTCCCTGAACGATCATTCTTACCGGACTGGGAACACTAATTACAGCCAGCTACTCCCTATATATATTCCTCATATCACAACGAGGCCCAACACCAAACCACATCACAGGACTCCAACCATTCCACACCCGAGAACACTTACTAATAACCATACACTTAATCCCCATTATCCTACTAGTGACAAAGCCAGAACTTATATGAGGATGATGCTAC

>A._jishouensis

ATGCTAAAAGTACTTGTCCCCACAATTATACTATTTCCAACAATTTGACTAACTTCCCCCAAGTGACTATGGACAACTACAACTACACATAGCCTCCTAATCGCTCTTACTAGCCTAACTTGACTAAAATGAACATCCGAAACCGGATGAACCTCCTCCAATACATACCTAGCCACAGACCCCTTATCAACCCCCCTCCTAGTACTAACATGCTGATTACTCCCACTCATAATTCTAGCCAGCCAAAACCACATCAGCCCTGAACCAATTGCCCGACAACGCACGTACATTATGCTTCTCGCCTCATTACAAACCTTCTTAATCATAGCATTCGGCGCTACAGAAGTCATTATATTCTACATCATATTTGAAGCCACACTTATCCCAACTCTCATCATTATCACCCGGTGAGGAAATCAAACCGAACGGCTTAATGCGGGAACTTATTTTCTGTTCTATACCCTAGCAGGATCACTCCCACTTCTCGTTGCCCTTCTCCTCCTCCAACAATCCACCGGCACCCTATCAATATTAGTGCTTCAATACTCACAACCCCTACAACTCAACTCCTGAGGCCATATAATCTGATGAGCTGGCTGCCTGATCGCATTCCTAGTCAAAATACCACTATATGGGGTTCACCTATGATTACCAAAAGCGCACGTAGAAGCCCCCGTGGCAGGATCTATAGTACTAGCAGCAGTTTTACTAAAACTCGGCGGGTACGGAATAATACGCATGATAGTAATACTCGACCCCTTATCAAAAGAACTAGCTTATCCATTTATTATTCTAGCCCTATGAGGCATCATTATAACTGGATCAATCTGCCTCCGACAAACGGACCTAAAATCATTAATTGCTTACTCATCCGTAGGTCATATAGGATTGGTAGCAGGAGGAATCCTAATTCAAACCCCATGAGGCTTCTCAGGAGCAATCATCCTTATAATTGCCCACGGATTAGCATCCTCAGCATTATTCTGTTTGGCCAATACAGCATATGAACGAACCCACAGTCGAACAATGGTCCTCGCCCGAGGACTACAAATGATCTTTCCACTAACAGCAGTCTGATGATTCACCGCTAATCTAGCCAACCTAGCACTACCACCACTGCCAAACCTAATAGGAGAACTCATAATCATCACAACATTGTTCAACTGATCCCCTTGAACAATCTTACTCACCGGACTAGGGACACTAATTACAGCCAGCTATTCCTTATACATATTCCTTATATCACAACGAGGCCCAACACCAAACCATATCACAGGACTTCAACCATTCCACACTCGAGAACATTTACTAATAACCCTACACTTAATCCCTATCGTCCTGCTAGTTACAAAACCAGAGCTCATGTGAGGATGATGCTAC

>A._kreyenbergii_1

ATGCTAAAAGTACTTATCCCCACAATTATGCTATTTCCAACAATTTGACTAACTTCCCCTAAATGACTATGAACAGCTACAACCACACATAGCCTATTAATTGCTCTTACTAGCCTAACTTGACTAAAATGAACATCCGAAACCGGATGGGCCTCCTCCAATATATATCTAGCCACGGACCCCCTATCAACCCCCCTCCTGGTACTAACATGCTGATTACTCCCACTCATAATTCTAGCCAGCCAAAACCATATTAGCCCAGAACCAATTGCCCGACAACGCACATATATTATGCTCCTCGCCTCATTACAAACCTTTTTAATTATAGCATTCGGCGCTACAGAAATCATTATATTCTACATCATATTCGAAGCCACACTTATCCCAACTCTTATCATTATTACCCGTTGAGGGAATCAAACTGAACGACTCAATGCGGGAACTTATTTTCTGTTCTACACCCTGGCGGGGTCACTACCCCTCCTCGTTGCCCTACTCCTCCTCCAACAATCCACCGGCACCCTATCAATATTAGTGCTCCAATATTCACAACCTCTACAACTCAACTCCTGAAGCCACATAATCTGATGAGCTGGCTGCCTGATTGCGTTTCTAGTCAAAATACCGCTATACGGAGTTCATCTATGACTGCCCAAAGCGCACGTAGAAGCCCCCGTGGCAGGATCTATAGTCCTAGCAGCAGTCTTACTAAAACTTGGTGGATACGGAATAATACGAATAATAGTAATGCTTGACCCCCTATCAAAAGAACTAGCTTATCCATTTATTATTCTGGCCCTATGGGGCATCATTATAACCGGGTCAATCTGCCTTCGACAAACGGACCTGAAATCATTAATTGCCTACTCATCCGTGGGCCATATGGGATTGGTGGCAGGAGGAATCCTAATTCAAACCCCATGGGGCTTCTCGGGAGCAATCATCCTTATAATTGCCCACGGATTAGCATCTTCAACACTATTCTGTCTAGCCAACACAGCATATGAACGAACTCACAGTCGAACAATAGTACTCGCCCGAGGGTTGCAAGTGATCTTTCCACTGACAGCAGTCTGGTGATTCATAGCTAATCTAGCCAACCTAGCACTACCACCGCTGCCGAATCTTATAGGGGAACTCATAATCATCACAACATTATTCAACTGATCCCCTTGAACAATCCTACTCACCGGACTAGGGACACTAATTACAGCCAGCTACTCCCTATACATATTCCTTATATCACAACGAGGCCCAACACCAAACCACATCTCAGGGCTCCAACCGTTCCACACCCGAGAACATTTGCTGATAACCATACACTTAATTCCCATTATCCTGCTAGTAACAAAACCAGAGCTTATATGAGGATGATGCTAC

>A._kreyenbergii_2

ATGCTAAAAGTACTTATCCCCACAATTATGCTATTTCCAACAATTTGACTAACTTCCCCTAAATGACTATGAACAACTACAACCACACATAGCCTATTAATTGCTCTTACTAGCCTAACTTGACTAAAATGAACATCCGAAACCGGATGGGCCTCCTCCAATATATATCTAGCCACAGACCCCCTATCAACCCCCCTCCTAGTACTAACATGCTGATTACTCCCACTCATAATTCTAGCCAGCCAAAACCATATTAGCCCAGAACCAATTGCCCGACAACGCACATATATTATGCTCCTCGCCTCATTACAAACCTTTTTGATTATAGCATTCGGCGCTACAGAAATCATTATATTCTACATTATATTCGAAGCCACACTTATCCCAACTCTTATCATTATCACCCGTTGAGGAAATCAAACTGAACGACTCAATGCGGGAACTTATTTTCTGTTCTACACCCTGGCGGGGTCACTACCCCTCCTCGTTGCCCTACTCCTCCTCCAACAATCCACCGGCACCCTATCAATATTAGTGCTTCAATATTCACAACCTCTACAACTCAACTCCTGAGGCCACACAATCTGATGAGCCGGCTGCCTAATTGCATTTCTAGTCAAAATACCACTATATGGAGTTCATCTATGACTGCCTAAAGCACACGTAGAAGCCCCCGTGGCAGGATCTATAGTCCTAGCAGCGGTTTTACTAAAACTCGGTGGATACGGAATAATACGAATAATAGTAATGCTTGACCCCTTATCAAAAGAACTAGCTTATCCATTTATTATTCTAGCCCTGTGGGGCATCATTATAACTGGGTCGATCTGCCTTCGACAAACAGACCTAAAATCACTAATTGCCTACTCATCCGTGGGCCATATGGGGTTGGTGGCAGGAGGGATCCTAATTCAAACCCCATGGGGCTTCTCGGGGGCAATCATCCTTATAATTGCCCACGGATTAGCATCTTCAGCATTATTCTGTTTAGCCAACACAGCATATGAACGAACTCACAGTCGAACAATGGTACTTGCCCGAGGATTGCAAGTGATCTTTCCGCTAACAGCAGTCTGATGATTCATAGCTAATCTAGCCAACCTAGCACTACCACCACTACCAAATCTTATAGGGGAACTAATAATCATCACAACATTATTCAACTGATCCCCCTGAACAATCCTACTCACCGGACTAGGGACGCTAATTACAGCCAGCTACTCCCTATACATATTCCTTATATCACAACGAGGCCCAACACCAAACCACATCTCAGGACTCCAACCGTTCCACACCCGAGAACATTTGCTGATGACCATGCACTTAATTCCCATTATCCTACTAGTAACAAAACCAGAGCTTATGTGAGGATGGTGCTAC

>A._longipinnis

ATGCTAAAAGTACTAATTCCCACAATTATGCTATTCCCAACAATTTGACTAACTTCCCCTAAATGGCTATGAACAACCACAACCGCACACAGCCTCCTAATTGCTCTCACCAGCCTAACATGACTAAAATGAACATCCGAAACCGGGTGAACCTCCTCCAACACATATATAGCCACAGACCCACTATCAACCCCCCTCCTAGTACTAACATGCTGACTACTCCCACTCATAATTCTAGCCAGCCAAAACCACATCAGCCCTGAACCGATTAGCCGACAACGCACGTACATCATACTCCTCGCCTCACTACAGACTTTTTTAATCTTAGCATTCGGTGCCACAGAAATCATTATATTCTATATTATATTTGAAGCTACACTTATCCCAACCCTTATCATCATTACCCGGTGAGGAAACCAAACTGAACGGCTCAATGCAGGAACCTATTTTCTGTTTTACACCCTAGCAGGGTCACTCCCACTTCTAGTAGCCTTACTCCTTCTTCAACAATCCACTGGCACCCTATCCATGCTAGTGCTTCAATATTCACAACCCCTACAACTCAACTCCTGAGGCCACATAATTTGATGAGCTGGCTGCCTAGTCGCATTTTTAGTTAAAATACCCCTATACGGAGTCCACCTATGATTACCAAAAGCACATGTAGAAGCCCCCGTAGCAGGATCTATAGTACTAGCAGCAGTTCTACTAAAACTCGGCGGGTACGGAATAATGCGCATAATAGTTATACTTGACCCCCTGTCAAAAGAGCTGGCCTACCCCTTCATTATCTTAGCCCTATGAGGCATTATTATAACCGGATCAATCTGCCTCCGACAAACAGACCTAAAATCACTTATTGCCTACTCATCCGTCGGCCACATGGGGCTAGTGGCAGGAGGAATTCTAATTCAAACTCCATGAGGCTTCTCAGGAGCAATCATTCTAATAATCGCCCACGGCCTAGCATCCTCAGCACTGTTCTGCCTAGCCAACACAGCATATGAACGAACCCACAGCCGAACAATAGTCCTTGCCCGAGGATTACAAGTAATTTTCCCGTTAACAGCAGTCTGATGATTCATCGCCAACCTGGCCAACCTAGCACTACCACCACTACCAAACCTCATGGGAGAACTTATAATCATCACAACATTATTTAACTGATCTCCCTGAACGATCATTCTTACCGGACTGGGAACACTAATTACAGCCAGCTACTCCCTATATATATTCCTCATATCACAACGAGGCCCAACACCAAACCACATCACAGGACTCCAACCATTCCACACCCGAGAACACTTACTAATAACCATACACCTAATCCCCATTATCCTACTAGTGACAAAGCCAGAACTTATATGAGGATGATGCTAC

>A._monticola_1

ATGCTAAAAGTACTAATCCCAACAATTATGTTATTCCCAACAATCTGACTGACATCCCCCAAGTGGCTATGAACAACCACAACCGCACACAGCCTCTTAATTGCTTTTACCAGCCTAACATGACTAAAATGAACATCCGAAACCGGATGAACCTCCTCCAACACATACCTGGCCACAGACCCACTGTCAACCCCCCTCCTAGTACTAACATGCTGATTACTCCCACTTATAATCCTAGCTAGCCAGAACCATATTAACCCTGAACCAATTAGCCGACAACGCTCATACATTATACTCCTCACCTCACTACAAACTTTTTTAATTATAGCATTCGGCGCCACAGAAATTATCATATTCTACATCATATTCGAAGCTACACTTATTCCAACCCTCATTATCATTACCCGATGAGGAAATCAAACCGAACGCCTCAATGCAGGAACCTATTTCTTATTCTACACCCTGGCAGGATCCCTCCCACTTCTAGTTGCCCTACTCCTTCTTCAACAAACCACCGGTACACTCTCAATATTAGTACTCCAATACTCACAACCTATACAACTCAATTCCTGAGGCCATACGCTCTGATGAGCTGGCTGCTTAATCGCATTTCTAGTAAAAATACCACTATATGGAGTTCACCTATGACTACCAAAAGCACATGTAGAAGCCCCTGTAGCAGGATCAATAGTACTAGCAGCAGTCCTACTAAAACTTGGTGGATATGGAATAATGCGCATAATAGTAATGCTCGACCCACTATCAAAAGAGCTAGCCTACCCATTTATCATCTTGGCCCTCTGAGGCATTATTATAACTGGGTCAATTTGCCTTCGACAAACAGACCTGAAATCACTAATTGCCTACTCATCCGTAGGCCATATGGGACTAGTGGCAGGAGGAATCCTAATTCAAACCCCCTGAGGTTTCTCGGGAGCAATTATTCTCATAATTGCCCACGGACTAGCATCCTCAGCATTATTCTGTTTAGCCAACACAGCATACGAACGAACTCACAGCCGAACAATAGTCCTTGCCCGAGGATTACAAGTGATCTTCCCACTTACAGCAGTATGATGATTTATCGCCAATCTGGCCAACCTGGCATTACCACCACTACCCAACCTAATAGGAGAACTCATAATTATCACAACACTATTCAACTGATCCCCCTGAACAATCCTGCTTACCGGACTAGGAACCCTGATCACAGCTAGCTACTCCCTGTACATATTCCTCATATCACAACGAGGCCCAACACCAAACCACATCACAGGACTTCAACCGTTCCATACCCGGGAACATCTACTAATGACCCTTCACCTAATTCCCATCATCCTACTAGTGACAAAACCAGAACTTATATGAGGATGATGCTAC

>A._monticola_2

ATGCTAAAAGTACTAATCCCAACAATTATGTTATTCCCAACAATCTGACTGACATCCCCCAAGTGGCTATGAACAACCACAACCGCACACAGCCTCTTAATTGCTTTTACCAGCCTAACATGACTAAAATGAACATCCGAAACCGGATGAACCTCCTCCAACACATACCTGGCCACAGACCCACTGTCAACCCCCCTCCTAGTACTAACATGCTGATTACTCCCACTTATAATCCTAGCTAGCCAGAACCATATTAACCCTGAACCAATTAGCCGACAACGCTCATACATTATACTCCTCACCTCACTACAAACTTTTTTAATTATAGCATTCGGCGCCACAGAAATTATCATATTCTACATCATATTCGAAGCTACACTTATTCCAACCCTCATTATCATTACCCGATGAGGAAATCAAACCGAACGCCTCAATGCAGGAACCTATTTCTTATTCTACACCCTGGCAGGATCCCTCCCACTTCTAGTTGCCCTACTCCTTCTTCAACAAACCACCGGTACACTCTCAATATTAGTACTCCAATACTCACAACCTATACAACTCAATTCCTGAGGCCATACGCTCTGATGAGCTGGCTGCTTAATCGCATTTCTAGTAAAAATACCACTATATGGAGTTCACCTATGACTACCAAAAGCACATGTAGAAGCCCCTGTAGCAGGATCAATAGTACTAGCAGCAGTCCTACTAAAACTTGGTGGATATGGAATAATGCGCATAATAGTAATGCTCGACCCACTATCAAAAGAGCTAGCCTACCCATTTATCATCTTGGCCCTCTGAGGCATTATTATAACTGGGTCAATTTGCCTTCGACAAACAGACCTGAAATCACTAATTGCCTACTCATCCGTAGGCCATATGGGACTAGTGGCAGGAGGAATCCTAATTCAAACCCCCTGAGGTTTCTCGGGAGCAATTATTCTCATAATTGCCCACGGACTAGCATCCTCAGCATTATTCTGTTTAGCCAACACAGCATACGAACGAACTCACAGCCGAACAATAGTCCTTGCCCGAGGATTACAAGTGATCTTCCCACTTACAGCAGTATGATGATTTATCGCCAATCTGGCCAACCTGGCATTACCACCACTACCCAACCTAATAGGAGAACTCATAATTATCACAACACTATTCAACTGATCCCCCTGAACAATCCTGCTTACCGGACTAGGAACCCTGATCACAGCTAGCTACTCCCTGTACATATTCCTCATATCACAACGAGGCCCAACACCAAACCACATCACAGGACTTCAACCGTTCCATACCCGGGAACATCTACTAATGACCCTTCACCTAATTCCCATCATCCTACTAGTGACAAAACCAGAACTTATATGAGGATGATGCTAC

>A._paradoxus_1

ATGTTAAAAGTACTAATCCCCACAATCATATTATTCCCAACAATCTGACTAACTTCCCCCAAATGACTATGAACCACCACAACCGCACACAGTCTCCTAATCGCCCTTATCAGCCTAACTTGACTAAAATGAACATCCGAAACCGGATGGAACTCCTCTAACACATACCTGGCCACAGACCCATTATCAACCCCCCTCCTAGTACTAACATGCTGATTACTTCCACTTATAATTTTAGCCAGCCAAAACCACATCAGCCCCGAACCAACCACCCGACAACGCACATATATTACACTCCTTGCCTCACTACAAACTTTTTTAATCATAGCATTCGGCGCTACAGAAGTCATTATATTTTATATCATATTTGAAGCTACACTCATCCCAACCCTCATTATTATTACCCGATGAGGAAATCAAACTGAACGACTCAGCGCAGGAACCTACTTCCTATTCTATACCCTAGCGGGATCACTCCCACTTCTAGTTGCCCTACTCCTTCTCCAACAATCCACCGGCACTCTATCAATACTAGTACTTCAATACTCACAACCCCTACAACTTAACTCTTGAGGCCACACAATTTGATGAGCCGGCTGTCTGATCGCATTCCTAGTTAAAATACCACTATACGGAGTCCACCTATGACTGCCAAAGGCGCACGTAGAGGCCCCCGTGGCAGGATCTATGGTACTAGCAGCAGTTTTACTAAAACTCGGCGGATACGGAATGATACGTATAATAGTAATGCTCGACCCCCTCTCAAAAGAACTAGCTTACCCATTTATTATTTTAGCCCTATGAGGCATCATTATAACTGGGTCAATCTGCCTTCGACAAACAGACTTAAAATCACTAATTGCCTACTCATCCGTAGGCCACATAGGATTGGTAGCAGGAGGGATCCTAATTCAAACCCCATGAGGCTTCTCAGGAGCAATCATCCTAATAATTGCCCACGGACTAGCATCCTCAGCACTATTCTGCTTAGCCAACACAGCATATGAACGAACTCACAGCCGAACAATAGTCCTCGCCCGAGGACTTCAAATGATTTTTCCACTAACAGCAATCTGATGATTTATCGCTAATCTGGCCAACCTAGCACTACCACCGCTGCCAAACCTCATGGGAGAACTCATAATCATCACAACATTATTCAACTGATCGCCCTGAACAATTTTACTCACCGGGCTAGGAACATTAATTACAGCCAGCTACTCCTTGTATATATTCCTAATATCACAACGAGGTCCGACACCAAACCATATTACAGGACTCCAACCATTCCACACCCGAGAACATCTACTAATAACCCTACACTTAATTCCCATCATCCTCCTAGTAACAAAACCAGAGCTCATGTGAGGGTGATGCTAC

>A._paradoxus_2

ATGCTAAAAGTACTTGTCCCCACAATTATACTATTTCCAACAATTTGACTAACTTCCCCCAAGTGACTATGGACAACTACAACTACACATAGCCTCCTAATCGCTCTTACTAGCCTAACTTGACTAAAATGGACATCCGAAACCGGATGAACCTCCTCCAATACATACCTAGCCACAGACCCCTTATCAACCCCCCTCCTAGTACTAACATGCTGATTACTCCCACTCATAATTCTAGCCAGCCAAAACCACATCAGCCCTGAACCGATTGCCCGACAACGCACGTACATTATGCTTCTCGCCTCATTACAAACCTTCTTAATCATAGCATTCGGCGCTACAGAAGTCATTATATTCTACATCATATTTGAAGCCACACTTATCCCAACTCTTATCATTATCACCCGGTGAGGAAATCAAACCGAACGGCTTAATGCGGGAACTTATTTTCTGTTCTATACCCTAGCAGGATCACTCCCACTTCTCGTTGCCCTTCTCCTCCTCCAACAATCCACCGGCACCCTATCAATATTAGTGCTTCAATACTCACAACCCCTACAACTCAACTCCTGAGGCCATATGATCTGATGAGCTGGCTGCCTGATCGCATTCCTAGTCAAAATACCACTATATGGGGTTCATCTATGATTACCAAAAGCGCACGTAGAAGCCCCCGTGGCAGGATCTATAGTACTAGCAGCAGTTTTACTAAAACTCGGCGGGTACGGAATAATACGCATGATAGTAATACTCGACCCCTTATCAAAAGAACTAGCTTATCCATTTATTATTCTAGCCCTATGAGGCATCATTATAACTGGATCAATCTGCCTCCGACAAACGGACCTAAAATCATTAATTGCTTACTCATCCGTAGGTCATATAGGATTGGTAGCAGGAGGAATCCTAATTCAAACCCCATGAGGCTTCTCAGGAGCAATCATCCTTATAATTGCCCACGGATTAGCATCCTCAGCATTATTCTGTTTGGCCAATACAGCATATGAACGAACCCACAGTCGAACAATGGTCCTCGCCCGAGGACTACAAATGATCTTTCCACTAACAGCAGTCTGATGATTCACCGCTAATCTAGCCAACCTAGCACTACCACCACTGCCAAACCTAATAGGAGAACTCATAATCATCACAACATTGTTCAACTGATCCCCTTGAACAATCTTACTCACCGGACTAGGGACACTAATTACAGCCAGCTATTCCTTATACATATTCCTTATATCACAACGAGGCCCAACACCAAACCATATCACAGGACTTCAACCATTCCACACTCGAGAACATTTACTAATAACCCTACACTTAATCCCTATCGTCCTGCTAGTTACAAAACCAGAGCTCATGTGAGGATGATGCTAC

>A._parallens_1

ATGCTAAAAGTATTAGTCCCTACAATTATACTATTCCCAACAATCTGACTAACTTCCCCTAAATGACTATGAACAACTACAACCGCACATAGCCTCCTGATTGCTCTTACCAGCTTAACTTGACTAAAGTGAACATCCGAAACCGGGTGAGCCTCCTCCAATACATACCTAGCCACAGACCCCTTATCAACCCCCCTCCTAGTACTAACATGCTGACTACTCCCACTCATAATTCTAGCCAGCCAAAACCACATCAGCCCCGAACCAATTGCCCGACAACGCACATACATCATACTTCTCGCCTCATTACAAACCTTCTTAATCATAGCATTTGGCGCTACAGAAGTCATTATATTTTACATCATATTTGAAGCCACACTTATTCCAACTCTTATCATCATCACCCGATGAGGAAATCAAACTGAACGACTTAGTGCGGGAACCTATTTTCTGTTCTACACCCTAGCAGGATCACTCCCGCTTCTCGTCGCCCTCCTCCTCCTCCAACAATCCACCGGCACCCTCTCAATATTAGTACTTCAGTACTCACAACCCCTACAACTCAACACCTGGGGCCACATGGTCTGATGAGCTGGCTGCCTAATCGCATTCCTAGTCAAAATACCCCTATATGGAGTCCATCTATGACTACCAAAAGCGCACGTAGAAGCCCCCGTGGCAGGATCTATGGTATTAGCAGCAGTTTTACTAAAGCTCGGCGGGTACGGGATAATACGCATAATAGTAATGCTCGACCCCTTATCAAAAGAACTAGCTTATCCATTTATTATCCTAGCCCTATGAGGCATCATTATAACTGGATCAACCTGCCTCCGACAAACAGACCTAAAGTCATTAATTGCCTACTCATCCGTAGGCCATATAGGATTAGTGGCAGGAGGGATCCTAGTTCAAACCCCATGGGGCTTCTCAGGAGCAATCATCCTTATAATTGCCCACGGATTAGCGTCTTCAGCATTATTCTGTCTAGCTAACACAGCATATGAACGAACCCATAGTCGAACGATGGTCCTCGCCCGAGGATTACAAATGATCTTTCCACTAGCAGCAGTCTGATGATTTACCGCTAATCTAGCCAACTTAGCACTACCACCACTGCCAAACCTCATAGGGGAACTCATAATCATCACAACATTATTTAACTGATCCCCCTGAACAATCTTACTTACTGGACTAGGAACACTAATTACAGCCAGCTACTCTCTATACATGTTCCTCATATCACAACGGGGCCCAACACCAGACCACATCACAGGACTTCAACCATTTCACACCCGAGAACATTTACTAATAACCCTGCACTTAATTCCTATCATCCTGCTAGTGACAAAACCAGAGCTTATATGAGGATGATGTTAC

>A._parallens_2

ATGCTAAAAGTACTAATCCCTACAATTATACTATTCCCAACAATCTGACTAACTTCCCCTAAATGACTATGAACAACTACAACCTCACATAGCCTCCTGATTGCTCTTACCAGCTTAACTTGACTAAAGTGAACATCCGAAACCGGATGAACCTCCTCTAATACATACCTAGCCACAGACCCCTTATCAACCCCCCTCCTAGTACTAACATGCTGATTACTCCCACTCATAATTCTAGCCAGCCAAAACCACATCAGCCCCGAACCAATTGCCCGACAACGCACGTACATTATACTTCTCGCCTCATTACAAACCTTCTTAATCATAGCATTTGGCGCTACAGAAGTCATTATATTTTACATCATATTTGAAGCCACACTTATTCCAACTCTTATTATCATCACCCGATGAGGAAATCAAACTGAACGACTTAATGCGGGAACCTATTTTCTGTTCTACACCCTAGCGGGATCACTCCCGCTTCTCGTCGCCCTCCTCCTCCTCCAACAATCCACCGGCACCCTCTCAATATTAGTACTTCAATACTCACAACCCCTACAACTCAACACCTGGGGCCATATGGTCTGATGAGCTGGCTGCCTAATCGCATTCCTGGTCAAAATACCTCTATATGGAGTCCATCTATGACTACCAAAAGCGCACGTAGAGGCCCCCGTGGCAGGATCTATAGTACTAGCAGCAGTTTTACTAAAGCTCGGCGGATACGGGATAATACGCATAATAGTGATGCTCGACCCCTTATCAAAAGAACTAGCTTATCCATTTATTATCCTAGCCCTATGAGGCATCATTATAACTGGATCAATCTGCCTTCGACAAACAGACCTAAAGTCATTAATTGCCTACTCATCCGTGGGCCATATGGGATTAGTGGCAGGAGGGATCCTAGTTCAAACCCCATGAGGCTTCTCAGGAGCAATCATCCTTATAATTGCCCACGGATTAGCGTCTTCAGCATTATTCTGTCTAGCTAACACAGCATATGAACGAACCCATAGTCGAACAATAGTCCTCGCCCGAGGATTACAAGTGATCTTTCCACTAACAGCAGTCTGATGATTTACCGCTAATCTAGCCAACTTAGCACTACCGCCACTGCCAAACCTTATAGGAGAACTCATAATCATCACAACATTATTTAACTGGTCCCCCTGAACAATCTTACTTACCGGACTAGGAACACTAATTACAGCCAGCTACTCCCTGTATATGTTCCTCATATCACAACGAGGCCCAATACCAAACCACATTACAGGACTTCAACCATTTCACACCCGAGAACATTTACTAATAACCCTGCACTTAATTCCTATCATCCTGCTAGTAACAAAACCAGAGCTTATATGAGGATGATGTTAC

>A._parallens_3

ATGCTAAAAGTACTAATCCCTACAATTATACTATTCCCAACAATCTGACTAACTTCCCCTAAATGACTATGAACAACTACAACCGCACACAGCCTCCTGATTGCTCTTACCAGCTTAACTTGACTAAAGTGAACATCCGAAACCGGGTGAGCCTCCTCCAATACATACCTAGCCACAGACCCCTTATCAACCCCCCTCCTAGTACTAACATGCTGACTACTCCCACTCATAATTCTAGCCAGCCAAAACCACATCAGCCCCGAACCAATTGCCCGACAACGCACATACATTATACTTCTCGCCTCATTACAAACCTTCTTAATCATGGCATTTGGCGCTACAGAAATCATTATATTTTACATCATATTTGAAGCCACACTTATTCCAACTCTTATCATCATCACCCGATGGGGAAATCAAACTGAACGACTTAATGCGGGAACCTATTTTCTGTTCTACACCCTAGCAGGATCACTCCCGCTTCTCGTCGCCCTCCTCCTCCTCCAACAATCCACCGGCACCCTCTCAATATTAGTACTTCAATACTCACAACCCCTACAACTCAACACCTGAGGCCACATGGTCTGATGGGCTGGCTGCCTAATCGCATTCCTAGTCAAAATACCCCTATATGGAGTCCATCTATGACTACCAAAAGCGCACGTAGAAGCCCCCGTGGCAGGATCTATGGTACTAGCAGCAGTTTTACTAAAGCTCGGCGGGTACGGAATAATACGCATAATAGTAATGCTCGACCCCTTATCAAAAGAACTAGCTTATCCATTTATTATCCTAGCCCTATGAGGCATCATTATAACCGGATCAATCTGCCTCCGACAGACGGACCTAAAGTCATTAATTGCCTACTCATCCGTAGGCCATATAGGATTAGTTGCAGGAGGAATCCTAGTTCAGACCCCATGAGGCTTCTCAGGGGCAATCATCCTTATAATTGCCCACGGATTAGCATCTTCAGCATTATTCTGTCTAGCTAACACAGCATATGAACGAACCCATAGTCGAACAATAGTCCTCGCCCGAGGACTACAAATGATCTTTCCACTAACAGCAGTCTGATGATTTACCGCTAATCTAGCCAACTTAGCACTACCACCACTGCAAAACCTTATAGGGGAACTCATAATCATCACAACATTATTTAATTGATCCCCCTGAACAATCTTACTTACTGGACTAGGAACACTAATTACAGCCAGCTACTCTCTATATATGTTCCTCATATCACAACGGGGCCCAACACCAAACCACATCACAGGACTTCAACCATTTCACACCCGAGAACATTTACTAATAACCCTGCACTTAATTCCCATCATCCTGCTAGTAACAAAACCAGAACTTATATGGGGATGATGTTAC

>A._parallens_4

ATGCTAAAAGTACTAATCCCTACAATTATACTATTCCCAACAATCTGACTAACTTCCCCTAAATGACTATGAACAACTACAACCGCACATAGCCTCCTGATTGCTCTTACCAGCTTAACTTGACTAAAGTGAACATCCGAAACCGGGTGAGCCTCCTCCAATACATACCTAGCCACAGACCCCTTATCAACCCCCCTCCTAGTACTAACATGCTGACTACTCCCACTCATAATTCTAGCCAGCCAAAACCACATCAGCCCCGAACCAATTGCCCGACAACGCACATACATTATACTTCTCGCCTCATTACAAACCTTCTTAATCATAGCATTTGGCGCTACAGAAATCATTATATTTTACATCATATTTGAAGCCACACTTATTCCAACTCTTATCATCATCACCCGATGGGGAAATCAAACTGAACGACTTAATGCGGGAACCTATTTTCTGTTCTACACCCTAGCAGGATCACTCCCGCTTCTCGTCGCCCTCCTCCTCCTCCAACAATCCACCGGCACCCTCTCAATATTAGTACTTCAATACTCACAACCCCTACAACTCAACACCTGAGGCCACATGGTCTGATGGGCTGGCTGCCTAATCGCATTCCTAGTCAAAATACCCCTATATGGAGTCCATCTATGACTACCAAAAGCGCACGTAGAAGCCCCCGTGGCAGGATCTATGGTACTAGCAGCAGTTTTACTAAAGCTCGGCGGGTACGGAATAATACGCATAATAGTAATGCTCGACCCCTTATCAAAAGAACTAGCTTATCCATTTATTATCCTAGCCCTATGAGGCATCATTATAACCGGATCAATCTGCCTCCGACAAACGGACCTAAAGTCATTAATTGCCTACTCATCCGTAGGCCATATAGGATTAGTTGCAGGAGGAATCCTAGTTCAGACCCCATGAGGCTTCTCAGGGGCAATCATCCTTATAATTGCCCACGGATTAGCATCTTCAGCATTATTCTGTCTAGCTAACACAGCATATGAACGAACCCATAGTCGAACAATAGTCCTCGCCCGAGGACTACAAATGATCTTTCCACTAACAGCAGTCTGATGATTTACCGCTAATCTAGCCAACTTAGCACTACCACCACTGCCAAACCTTATAGGGGAACTCATAATCATCACAACATTATTTAATTGATCCCCCTGAACAATCTTACTTACTGGGCTAGGAACACTAATTACAGCCAGCTACTCTCTATATATGTTCCTCATATCACAACGGGGCCCAACACCAAACCACATCACAGGACTTCAACCATTTCACACCCGAGAACATTTACTAATAACCCTGCACTTAATTCCCATCATCCTGCTAGTAACAAAACCAGAACTTATATGGGGATGATGTTAC

>A._spinifer

ATGTTAAAAGTACTAATCCCCACAATCATATTATTCCCAACAATTTGATTAACCTCCCCTAAATGACTATGAACAACCACAACTGCACACAGTCTCCTAATCGCTCTTATTAGCCTAACCTGACTAAAATGAACATCCGAAACCGGATGGAGCTCCTCTAACACATACCTAGCCACAGACCCATTATCAACCCCCCTCCTAGTACTAACATGCTGATTACTCCCACTTATAATTTTAGCCAGCCAAAATCACATCAGCCCTGAACCAACCACCCGACAACGCACATATATTACTCTCCTTACCTCACTACAAACTTTTTTAATCATAGCATTCGGCGCTACGGAGGTCATTATATTTTATATTATATTTGAAGCTACACTCATCCCAACCCTCATTATCATTACCCGATGAGGAAATCAAACTGAACGACTCAATGCAGGAACCTATTTTCTATTCTATACCCTAGCAGGATCACTCCCACTTCTAGTTGCCCTACTCCTTCTCCAACAATCCACCGGCACCCTATCAATACTAGTGCTTCAATACTCACAGCCCTTGCAGCTCAACTCTTGAGGCCACACAATCTGATGAGCTGGCTGTCTGATCGCATTCCTAGTTAAAATACCACTATATGGAGTTCACCTGTGACTACCAAAGGCGCACGTAGAAGCCCCCGTAGCAGGATCCATAGTACTAGCAGCAGTTTTATTAAAACTCGGCGGATACGGAATAATACGCATAATAGTAATGCTCGACCCACTGTCAAAAGAGCTAGCCTATCCATTTATTATTTTAGCCCTATGAGGCATCATTATAACTGGATCAATCTGCCTTCGACAAACAGACTTAAAGTCATTAATTGCCTACTCATCCGTAGGCCACATAGGATTAGTAGCAGGAGGGATCCTAATTCAAACCCCATGAGGCTTCTCAGGAGCAATTATCCTAATAATTGCCCATGGACTAGCATCCTCAACACTATTCTGTTTGGCCAACACAGCATATGAACGAACTCACAGCCGAACAATAATCCTTGCCCGAGGACTTCAAATGATTTTTCCACTAACGGCAGTCTGATGATTTATCGCTAATCTGGCTAACCTAGCGCTACCACCACTACCAAACCTCATAGGAGAACTTATAATCATTACAACATTATTCAACTGATCCCCCTGAACAATCCTACTCACCGGGCTAGGAACATTAATTACAGCCAGCTATTCCTTATACATATTCCTAATATCACAACGAGGCCCAGCACCAAACCATATTACAGGACTCCAACCATTCCACACCCGAGAACATCTACTAATAACCCTACACTTAATCCCCATTATCCTACTAATAACAAAACCAGAACTTATGTGAGGATGATGCTAC

>A._stenotaeniatus

ATGTTAAAAGTACTAATCCCCACAATCATATTATTCCCAACAATTTGATTAACTTCCCCTAAATGACTATGAACAACCACAACTGCACACAGTCTCCTAATCGCTCTTATTAGCCTAACCTGACTAAAATGAACATCCGAAACCGGATGGAACTCCTCTAACACATACCTAGCCACAGACCCATTATCAACCCCCCTCCTAGTACTAACATGCTGATTACTCCCACTTATAATTTTAGCCAGCCAAAATCACATCAGCCCTGAACCAACCACCCGACAACGCACATATATTACTCTCCTTACCTCACTACAAACTTTTTTAATCATAGCATTCGGCGCTACAGAGGTCATTATGTTTTATATTATATTTGAAGCTACACTCATCCCAACCCTCATTATTATTACCCGATGAGGAAATCAAACTGAACGACTCAATGCAGGGACCTATTTTCTATTCTATACCCTGGCAGGATCACTTCCACTTCTAATTGCCCTACTCCTTCTCCAACAATCCACCGGCACCCTATCAATACTAGTACTTCAATACTCACAGCCCTTGCAGCTCAACTCTTGAGGCCACACAATCTGATGAGCTGGCTGTCTGATCGCATTCCTAGTTAAAATACCACTATATGGCGTTCACCTATGACTACCAAAGGCGCACGTAGAAGCCCCCGTAGCAGGATCCATAGTACTAGCAGCAGTTTTATTAAAACTCGGCGGATACGGAATAATACGCATAATAGTAATGCTCGACCCACTGTCAAAAGAGCTAGCCTATCCATTTATTATTTTAGCCCTATGAGGCATCATCATAACCGGGTCAATCTGCCTTCGACAAACAGACTTAAAGTCATTAATTGCCTACTCATCCGTAGGCCATATGGGACTAGTAGCAGGAGGAATCCTAATTCAAACCCCATGAGGCTTCTCAGGAGCAATTATCCTAATAATTGCCCATGGACTAGCATCCTCAACACTATTCTGTTTGGCCAACACAGCATATGAACGAACTCACAGCCGAACAATAATCCTTGCCCGAGGACTTCAAGTGATTTTTCCACTAACGGCAGTCTGATGATTTATCGCTAATCTGGCTAACCTAGCACTACCACCACTACCAAACCTCATAGGAGAACTTATAATCATTACAACATTATTCAACTGATCCCCCTGAACAATCCTACTCACCGGGCTAGGAACATTAATTACAGCCAGCTATTCCTTATACATATTCCTAATATCACAACGAGGCCCAGCACCAAACCATATTACAGGACTCCAACCATTCCACACCCGAGAACATCTACTAATAACCCTACACTTAATCCCCATTATCCTACTAATGACAAAACCAGAACTTATGTGAGGATGATGCTAC

>A._wenchowensis_1

ATGCTAAAAGTACTTGTCCCCACAATTATACTATTTCCAACAATTTGACTAACTTCCCCCAAATGACTATGAACAACTACAACCACACATAGCCTCTTAATTGCTCTTATTAGCCTAACTTGACTAAAATGAACATCCGAAACCGGGTGGGCCTCCTCCAATATATACCTAGCCGCAGATCCCTTATCAACCCCCCTCCTAGTACTAACATGCTGATTACTCCCACTCATAATTCTAGCCAGCCAAAACCACATCAGCCCTGAACCAATTGCCCGACAACGCACATATATTATGCTCCTCACCTCATTACAAACCTTTTTAATTATAGCATTCGGCGCCACAGAAGTGATTATATTCTACATCATATTTGAAGCCACACTTATCCCAACTCTTATTATTATTACCCGTTGAGGGAATCAAACTGAGCGACTCAATGCGGGAACTTATTTTCTGTTCTATACCCTAGCAGGGTCACTACCCCTCCTCGTTGCCCTGCTCCTCCTCCAACAATCCACCGGCACCCTATCAATATTAGTGCTTCAATACTCACAACCCCTACAACTTGACTCCTGAGGCCACACGATCTGGTGAGCTGGCTGCCTGATTGCATTTCTAGTCAAAATACCACTATACGGAGTTCATCTATGACTGCCAAAAGCGCACGTAGAAGCCCCCGTGGCAGGATCTATAGTCCTAGCAGCAGTTTTACTGAAACTTGGTGGATACGGAATAATACGAATGATGGTAATGCTTGACCCCCTATCTAAAGAACTAGCTTATCCATTTATTATTCTAGCCCTGTGGGGCATTATTATAACTGGGTCAATCTGCCTCCGACAAACGGACCTAAAGTCATTAATTGCCTACTCATCCGTAGGCCATATAGGGTTAGTAGCAGGAGGAATCCTAATTCAAACCCCATGGGGCTTTTCAGGAGCAATCATCCTTATAATTGCCCACGGATTAGCATCTTCAGCATTATTCTGTTTAGCCAACACAGCATATGAACGAACCCACAGCCGAACAATAGTACTTGCCCGAGGACTACAAGTGATCTTTCCACTAACAGCAGTCTGATGATTCATAGCTAATCTAGCCAACCTAGCACTACCACCACTACCAAACCTTATGGGGGAACTCATAATTATCACAACATTATTCAACTGATCCCCCTGAACAATCCTACTTACCGGACTAGGGACACTAATTACAGCCAGCTACTCCCTATACATATTCCTTATATCACAACGAGGCCCAACACCAAACCACATCTCAGGGCTCCAACCGTTCCACACCCGAGAACATTTACTGATAACCATACACTTAATTCCTATTATCCTACTCGTGACAAAACCAGAGCTTATGTGAGGATGATGCTAC

>A._wenchowensis_2

ATGCTAAAAGTACTTATTCCCACAATCATACTATTTCCAACAATTTGACTAACTTCCCCCAAATGACTATGAACAACTACAACCACACATAGCCTCTTAATTGCCCTTATTAGCCTAACTTGACTAAAATGAACATCCGAAACCGGATGGGCCTCCTCCAATATATACCTAGCCACAGACCCCTTATCAACCCCCCTCCTAGTACTAACGTGCTGATTACTCCCACTCATAATTCTAGCCAGTCAAAACCATATTAGCCCCGAACCAATTGCCCGACAACGTACATATATTATGCTCCTCACCTCATTACAAACCTTTTTAATTATAGCATTCGGTGCCACAGAAATCATTATATTTTACATCATGTTCGAAGCCACACTTATCCCAACTCTTATTATTATCACCCGTTGGGGAAATCAGACTGAACGACTCAATGCGGGGACTTATTTTCTGTTCTACACCCTGGCAGGATCATTACCCCTCCTCGTCGCCCTGCTCCTCCTCCAACAATCCACCGGCACCCTATCAATATTAGTGCTTCAATACTCACAACCCCTGCAACTCAACTCCTGAGGCCACACAATCTGATGAGCTGGCTGCCTAATTGCATTTCTAGTCAAAATACCACTATACGGAGTTCATCTGTGACTGCCAAAAGCGCACGTTGAAGCCCCCGTGGCAGGATCTATAGTCCTAGCAGCAGTTTTACTAAAACTTGGCGGATATGGAATAATACGAATGATAGTAATGCTTGACCCCCTATCAAAAGAACTGGCCTACCCATTTATTATCCTAGCTCTATGAGGCATCATTATAACTGGGTCAATCTGCCTCCGACAAACAGACCTAAAGTCATTAATCGCCTACTCATCCGTAGGCCATATGGGGTTAGTGGCAGGAGGAATCCTAATTCAAACCCCATGAGGCTTCTCAGGGGCAATCATCCTTATAATTGCCCACGGATTAGCATCTTCAGCATTGTTCTGTTTGGCTAACACAGCATATGAACGAACCCACAGTCGAACAATAGTACTTGCCCGAGGACTACAAGTGATCTTTCCACTAACAGCAGTCTGATGATTTGTAGCAAATCTAGCTAACCTAGCACTGCCACCACTGCCAAACCTTATAGGGGAACTCATAATCATCACAACATTATTCAACTGATCCCCCTGAACAATCCTACTCACCGGACTAGGGACACTAATTACAGCCAGCTACTCCCTATACATATTCCTTATATCACAACGAGGCCCAACGCCAAACCACATCTCAGGCCTCCAACCGTTCCACACTCGAGAACACTTACTGATAATCATACACTTAATCCCTATTATCCTGCTAGTGACAAAACCAGAGCTTATATGAGGATGATGCTAC

>A._wuyiensis

ATGTTAAAAGTACTAATCCCCACAATCATATTATTCCCAACAATCTGACTAACTTCCCCCAAATGACTATGAACGACCACAACCGCACACAGTCTCCTAATTGCCCTTATCAGCCTAACTTGACTAAAATGAACATCCGAAACCGGATGAAACTCCTCTAACACATACCTGGCCACAGACCCATTATCAACCCCCCTTCTAGTACTAACATGCTGATTACTCCCACTTATAATTTTAGCCAGCCAAAACCACATCAGCCCCGAACCAATCACCCGACAACGCACATATATTACACTCCTTGCCTCACTACAAACTTTTTTAATTATAGCATTCGGCGCTACAGAAGTCATTATATTTTATATCATATTTGAAGCTACACTCATCCCAACCCTCATTATTATTACCCGATGAGGAAATCAAACTGAACGACTCAGCGCAGGAACCTACTTCCTATTCTATACCCTAGCGGGATCACTCCCACTTCTAGTTGCCCTACTCCTTCTCCAGCAATCCACCGGCACTCTATCAATGCTAGTACTTCAATACTCACAACCCCTACAACTCAACTCTTGAGGCCACACAATTTGATGAGCCGGCTGTCTGATCGCATTCCTAGTTAAAATACCACTATATGGAGTCCACCTATGACTGCCAAAGGCGCACGTAGAAGCCCCCGTGGCAGGATCTATAGTACTAGCAGCAGTTTTACTAAAACTCGGCGGATACGGAATGATACGTATAATAGTAATGCTCGACCCCCTCTCAAAAGAACTAGCTTACCCATTTATTATTTTAGCCCTATGAGGCATCATTATAACTGGGTCAATCTGCCTTCGACAAACAGACTTAAAATCACTAATTGCCTACTCATCCGTAGGCCACATGGGATTGGTAGCAGGAGGGATCCTAATTCAAACCCCATGAGGCTTCTCAGGAGCAATCATCCTAATAATTGCTCACGGACTAGCATCCTCAGCACTATTCTGCTTGGCCAACACAGCATATGAACGAACTCACAGCCGAACAATGGTCCTCGCCCGAGGACTTCAAGTGATTTTTCCACTAACAGCAATCTGATGATTCATCGCTAATCTGGCCAACCTAGCACTGCCACCACTGCCAAACCTCATGGGAGAACTCATAATCATCACAACATTATTCAACTGATCGCCCTGAACAATTTTACTCACCGGGCTAGGAACATTAATTACAGCCAGCTACTCCTTGTATATATTCCTAATATCACAACGAGGTCCAACACCAAACCATATTACAGGACTTCAACCATTCCACACCCGAGAACATCTACTAATAACCCTACACTTAATTCCCATCATCCTCCTAGTAACAAAACCAGAGCTCATGTGAGGATGATGCTAC

>A._yunnanensis_1

ATGCTAAAAGTACTAATCCCAACAATTATATTATTCCCAACAATCTGATTAACGTCCCCTAAATGACTGTGAACAACTACAACCGCACACAGCCTCCTAATTGCTTTTACTAGCCTAACATGACTAAAATGAACATCTGAAACCGGATGAACCTCCTCCAACACATACCTGGCCACAGACCCATTATCAACCCCCCTCCTAGTACTAACATGTTGATTACTTCCACTTATAATCTTAGCCAGCCAAAACCATATTAACCCGGAACCAATCAGCCGACAACGCTCATATATTATACTCCTCGCCTCACTACAAACTTTTTTAATTATAGCATTCGGCGCCACAGAAATTATTATATTCTACATTATATTTGAAGCTACACTTATTCCAACCCTCATCATCATTACCCGATGAGGAAATCAAACCGAACGCCTCAATGCAGGAACCTATTTCTTATTCTATACCTTAGCAGGGTCACTTCCACTTCTAGTTGCCTTACTCCTCCTTCAACAATCTACTGGTACGCTCTCAATATTAGTACTTCAATATTCACAACCTATACAACTCAATTCCTGAGGCCACACGCTCTGATGAGCTGGCTGCCTAATCGCATTTTTAGTTAAAATACCACTATATGGGGTTCACCTGTGACTACCAAAAGCACATGTAGAAGCCCCCGTAGCAGGATCAATGGTCCTAGCAGCAGTCCTACTAAAACTTGGCGGATATGGAATAATGCGCATAATAGTGATACTTGACCCCCTATCAAAAGAACTCGCTTATCCATTCATCATTTTAGCCCTCTGAGGCATTATTATAACAGGATCAATCTGCCTTCGACAAACAGACCTGAAATCACTAATTGCCTACTCATCCGTAGGCCACATAGGCCTCGTAGCAGGAGGAATCCTAATTCAAACCCCATGAGGCTTCTCAGGGGCAATCATCCTTATAATTGCCCACGGACTAGCATCCTCAGCATTATTCTGTCTGGCCAACACAGCGTATGAACGAACCCATAGCCGAACAATAGTCCTTGCCCGAGGACTACAAGTGATTTTCCCGCTGACAGCAGTATGATGATTCATCGCTAACCTAGCTAACCTAGCACTACCACCACTGCCTAACCTAATAGGAGAACTTATAATTATCACAACATTATTCAACTGATCCCCTTGAACAATCCTGCTCACCGGACTAGGAACACTGATCACAGCCAGCTACTCCTTATACATGTTCCTCATGTCACAACGAGGCCCAACACCAAGCCACATTACAGGACTCCAACCGTTCCACACCCGGGAACACTTACTAATAACCCTTCACCTAATTCCCATCATCCTACTAGTGACAAAACCAGAGCTTATATGAGGATGATGCTAC

>A._yunnanensis_2

ATGCTAAAAGTACTAATCCCAACAATTATATTATTCCCAACAATCTGATTAACATCCCCCAAATGACTGTGAACAACCACAACCGCACACAGCCTCCTAATTGCTTTTACTAGCCTAACATGACTAAAATGAACATCTGAAACCGGATGAACCTCCTCCAACACATACCTGGCCACAGACCCATTATCAACCCCCCTCCTAGTGTTAACATGTTGATTACTTCCACTTATAATCTTAGCCAGCCAAAACCACATTAACCCGGAACCAATTAGCCGACAACGCTCATATATTATACTCCTCGCCTCACTACAAACTTTTTTAATTATAGCATTCGGCGCCACAGAAATTATTATATTCTACATTATATTTGAAGCCACACTTATTCCAACCCTCATCATCATTACCCGATGAGGAAATCAAACCGAACGCCTCAATGCAGGAACCTATTTCTTATTCTATACCTTAGCAGGGTCACTTCCACTTCTAGTTGCCTTACTACTCCTTCAACAATCTACTGGTACGCTCTCAATATTAGTACTTCAATATTCACAACCTATACAACTCAACTCCTGAGGCCACACGCTCTGATGAGCTGGCTGCCTAATCGCATTTTTAGTTAAAATACCACTATATGGAGTTCACCTATGACTACCAAAAGCACATGTAGAAGCCCCCGTAGCAGGATCAATGGTCCTAGCAGCAGTCCTACTAAAACTTGGCGGATACGGAATGATACGCATAATAGTAATACTTGACCCCCTATCAAAAGAACTCGCTTATCCATTCATCATTTTAGCCCTCTGAGGCATTATTATAACCGGGTCAATCTGCCTTCGACAAACAGACCTGAAGTCACTAATTGCCTACTCATCCGTAGGCCACATGGGCCTCGTAGCAGGAGGAATCCTAATTCAAACCCCATGAGGCTTCTCAGGGGCAATCATCCTTATAATTGCCCACGGACTAGCATCCTCAGCATTATTCTGTCTGGCCAACACAGCATATGAACGAACCCATAGCCGAACAATAGTCCTTGCCCGAGGACTACAAGTGATTTTTCCACTGACAGCAGTATGATGATTCATCGCTAACCTAGCTAACCTAGCACTACCACCACTGCCTAACCTAATAGGAGAACTTATAATTATTACAACATTATTCAACTGATCCCCTTGAACAATCCTGCTCACCGGACTAGGAACGCTGATCACAGCCAGCTACTCCTTATACATGTTCCTCATATCACAACGAGGCCCGACACCAAACCACATTACAGGACTTCAACCGTTCCACACCCGAGAACACTTACTAATAACCCTTCACCTAATTCCCATCATCCTACTAGTGACAAAACCAGAGCTTATATGAGGATGATGCTAC

>Onychostoma_barbatulum

ATGCTAAAAGTACTGATCCCTACGATTATATTATTCCCAACAATTTGACTAACTTCCCCCAAATGACTGTGAACAACCACAACTGCACACAGCCTCCTAATTGCCCTTACCAGCCTGACCTGACTAAAATGAACATCCGAAACCGGATGGACCTCCTCCAACACATACCTAGCCGCAGACCCATTATCAACCCCTCTCCTAGTCCTAACATGCTGATTACTCCCCCTTATAATCTTAGCCAGCCAAAACCACATCAACCCAGAACCAATCAACCGACAACGTACATATATTATACTTCTCACTTCATTACAAGCCTTTTTAATTATAGCATTCGGCGCCACAGAAGTCATTATATTCTACATCATATTTGAAGCCACACTTATCCCAACCCTAATTATTATTACCCGATGGGGAAATCAAACCGAACGCCTCAATGCAGGAACCTATTTCCTGTTTTATACCCTAGCAGGATCACTCCCACTCCTAGTTGCCCTGCTCCTTCTTCAACAATCCACCGGTACCTTATCAATGTTAGTACTTCAATATTCACAACCCTTGCAACTCAACTCCTGAGGCCACACAATCTGATGAGCCGGCTGTCTAATCGCATTTCTAGTTAAAATACCACTATATGGGGTCCACCTGTGATTACCAAAAGCGCACGTAGAAGCTCCTGTGGCGGGATCCATGGTCCTAGCAGCAGTTCTGCTGAAACTCGGCGGGTATGGAATAATACGCATAATAGTAATGCTTGACCCCCTATCAAAAGAACTAGCTTACCCATTCATCATCTTAGCTCTCTGAGGTATTATTATAACCGGATCAATCTGCTTACGACAAACAGATCTAAAATCACTAATTGCCTATTCATCCGTAGGTCACATAGGACTAGTAGCAGGAGGGATCCTAACTCAAACCCCATGAGGCTTCTCAGGTGCAATCATTCTTATAATTGCCCACGGACTAGCATCCTCAGCACTATTCTGCCTAGCCAACACAGCATATGAACGAACCCACAGCCGAACAATAATCCTTGCCCGAGGACTACAAGTGATCTTTCCATTAACAACAGTCTGATGATTCACCGCCAATCTGGCCAACCTAGCACTACCACCACTACCAAACCTAATAGGAGAACTCATAATTATTACCACATTATTTAACTGATCACCTTGAACAATACTACTCACCGGACTGGGGACATTAATTACAGCCAGCTATTCCCTGTACATATTTCTTATATCACAACGAGGCTCAGCACCAAACCACATCACAGGACTCCAACCTTTCCACACTCGGGAACACCTACTACTAACCCTACACTTAACCCCGATCATCCTACTAGTAACAAAACCAGAGCTTATGTGAGGATGGTGCTAC

>Onychostoma_meridionale

ATGCTAAAAGTACTAATCCCCACAATTATACTATTCCCAACAATTTGACTAGCCTCCCCTAAATGATTATGAACAACCACAACCGCACATAGCCTTCTAATTGCCCTCACCAGCCTAACCTGACTAAAATGAACATCTGAAACCGGATGAGCCTCCTCTAACACGTACCTCGCCACAGACCCCTTATCAACCCCCCTCCTAGTCTTAACATGCTGATTACTTCCACTTATAATTTTAGCCAGCCAAAACCACATCAACCCCGAACCAATTAGCCGACAACGCACATATATTACCCTCCTCACTTCACTACAAACTTTTTTAATTTTAGCATTCGGCGCCACAGAAATCATCATATTCTACATCATATTTGAAGCCACACTTATTCCAACCCTTATTATTATTACCCGGTGAGGAAATCAAACCGAACGACTCAATGCAGGGACCTATTTTCTATTTTACACCCTAGCAGGATCACTCCCGCTCCTAGTCGCCCTACTCCTCCTTCAACAATCCACCGGTACCCTATCAATACTGGTACTTCAATATTCACAACCTCTACCACTCAGCTCCTGAGGCCACACAATCTGATGAGCCGGATGCCTAATCGCATTTTTAGTCAAAATACCACTATATGGAGTTCACCTCTGATTACCAAAAGCACATGTAGAAGCCCCCGTAGCAGGATCTATGGTTCTAGCAGCAGTTTTACTAAAACTTGGCGGTTACGGAATAATGCGCATAATAGTAATGCTTGACCCCCTATCAAAAGAGTTAGCCTACCCATTTATCATCCTAGCCCTATGGGGTATTATTATAACCGGATCGATCCGCTTACGACAAACAGACTTAAAATCATTAATTGCCTACTCATCCGTAGGCCATATAGGACTAGTAGCAGGAGGCATTTTAATTCAAACCCCATGAGGATTCTCAGGAGCAATCATTCTCATAATTGCCCACGGATTAGCATCCTCAGCACTATTCTGCCTAGCCAACACAGCATATGAACGAACCCACAGCCGGACAATAGTCCTTGCCCGAGGACTTCAAGTAATCTTCCCATTAACAGCAGTCTGATGATTCATTGCTAACCTAGCCAACCTAGCACTACCACCACTACCAAACCTAATGGGGGAACTTATAATCATCACAACATTATTCAACTGATCCCCCTGAACAATCTTACTCACCGGACTAGGAACACTAATCACAGCCAGCTATTCCCTGTACATGTTCCTCATATCACAACGAGGCCCAACACCAAATCACATCACAGGCCTCCAACCATTTCATACCCGGGAACACCTACTAATAACCATACACTTAATTCCAATCATTCTACTGGTAACAAAGCCAGAACTTATATGAGGATGATGCTAC

>Onychostoma_gerlachi

ATGCTAAAAGTACTAATCCCCACAATCATAATATTCCCAACAATTTGACTAACCTCCCCAAAATGACTATGAACAACCACAACCGCACATAGTCTCCTAATTGCCCTCACCAGCCTAACCTGACTAAAATGAACATCCGAAACCGGGTGAACCTCCTCTAACACATACCTCGCCACAGACCCGCTATCAACCCCTCTCCTAGTCCTAACATGTTGACTACTTCCACTTATAATTTTGGCCAGCCAAAACCACATCAACCCCGAACCAATTAGCCGACAACGCACATATATTACACTCCTCACTTCACTGCAAACTTTTTTAATTATAGCATTCGGCGCCACAGAAATCATCATATTTTATATTATGTTTGAAGCCACACTAATCCCAACTCTTATCATTATTACCCGGTGAGGAAATCAAACCGAACGACTCAATGCAGGGACCTATTTTCTGTTTTACACCTTAGCAGGATCGCTCCCGCTCCTAGTCGCCCTACTACTCCTTCAACAATCCACTGGTACCCTATCAATACTAGTACTTCAATATTCACAACCCCTACAACTCAGCTCCTGAGGCCATACAATCTGATGAGCCGGATGCCTAATCGCATTTTTAGTTAAAATACCACTATACGGAGTCCACCTTTGACTTCCAAAAGCACACGTAGAAGCCCCCGTGGCAGGATCTATGGTTCTAGCAGCAGTTTTACTAAAACTTGGCGGATACGGAATGATACGCATAATAGTAATGCTTGACCCCCTATCAAAAGAACTAGCCTACCCGTTCATTATCCTTGCCCTATGGGGTATTATTATAACCGGATCAATCTGCCTACGACAAACAGACCTAAAATCATTAATCGCCTACTCATCCGTCGGCCACATAGGACTAGTAGCAGGAGGCATTCTAATTCAAACCCCATGAGGCTTCTCAGGGGCAATCATTCTCATAATTGCCCACGGATTAGCATCCTCAGCACTATTCTGCTTAGCCAATACAGCATATGAACGAACCCACAGCCGAACAATAGTCCTTGCCCGAGGACTGCAAGTGATCTTTCCACTAACAGCAGTCTGATGATTCATTGCTAATCTAGCCAACCTAGCACTACCACCACTGCCAAACCTAATGGGAGAACTTATGATCATCACGACATTATTCAACTGATCCCCCTGAACAATCTTACTCACCGGATTAGGAACACTAATTACAGCCAGCTATTCCCTATATATGTTCCTTATATCACAACGAGGCCCAACACCAAACCACATCACAGGCCTCCAACCATTTCATACCCGAGAACACCTACTAATAACCATACACTTAATTCCAATCATCCTACTGGTAACAAAACCAGAACTTATATGAGGATGATGCTAC

>Spinibarbus_denticulatus

ATGTTAAAAGTATTAATCCCCACAATTATACTATTCCCAACAATCTGACTAACTTCCCCTAAATGACTGTGAACAACCACAACTACACACAGCCTCCTAATTGCTTTCATTAGCCTAACATGACTAAAATGGACATCCGAAACCGGATGAAGCTCCTCCAATATATATCTGGCCACAGACCCATTATCAACCCCACTCCTAGTACTAACATGTTGATTACTTCCACTTATAATCCTAGCCAGCCAAAACCACATTAATCCAGAACCAATTAGCCGACAGCGCCTATATATTACACTCCTTGCCTCACTACAAACCTTCTTAATTATAGCATTTGGCGCTACAGAAATTATTATATTCTACATTATATTTGAAGCCACACTTATCCCAACCCTTATTATTATTACTCGATGAGGAAATCAAACCGAACGACTCAATGCAGGAACCTACTTCCTATTTTACACTTTAGCCGGATCACTCCCACTTTTAGTTGCCCTACTCCTCCTCCAACAATCCACTGGAACACTATCAATATTGGTACTTCAATACTCACAACCCCTACAACTCAACTCCTGAGCCCACATAATCTGATGGGCCGGCTGCCTAATCGCATTTTTAGTCAAAATACCATTGTACGGGGTTCACTTGTGATTACCAAAAGCACATGTAGAAGCCCCTGTAGCAGGATCAATAGTCCTAGCTGCAGTCCTACTAAAACTCGGCGGATACGGAATAATACGTATAATAGTAATACTAGATCCCCTATCAAAAGAGCTAGCCTATCCATTCATCATTTTAGCCCTCTGAGGCATTATCATGACAGGATCAATCTGCCTCCGACAAACAGACCTAAAGTCTCTAATTGCCTACTCATCTGTAAGTCATATAGGACTAGTGGCAGGAGGAATCCTAATCCAAACCCCATGGGGATTTTCAGGAGCAATCATTCTAATAATTGCCCACGGACTAGTATCCTCAGCACTATTCTGCCTGGCCAACACAGCATACGAACGAACCCACAGCCGAACAATAATCCTTGCCCGAGGACTACAAATGATTTTTCCCCTAACCGCAATATGATGATTTATTGCCAACCTGGCTAACCTAGCCCTCCCTCCACTACCTAACCTAATAGGAGAACTCATGATCATCACAACATTATTTAATTGATCCCCATGAACAATTCTACTCACCGGACTAGGGACATTAATTACAGCCGCCTACTCCTTATACATATTTCTCATGTCACAACGAGGACCAACACCAAACCACATCATAGGACTCCAACCATTCCACACCCGAGAACACCTGCTAATAACCCTACATCTAATTCCCGTTATCCTACTAGTAACAAAACCAGAACTTATATGAGGATGATGCTAT

>Spinibarbus_hollandi

ATGCTAAAAGTACTAATCCCCACAATTATACTATTCCCAACAATCTGACTTACTTCCCCTAAATGACTATGAACAACCACAACTACACACAGCCTCCTAATTGCTTCCATTAGCCTAACATGACTAAAATGGACATCCGAAACCGGATGAGCCTCCTCCAACATATACCTGGCCACAGACCCATTATCAACCCCACTTCTAGCACTAACATGCTGATTACTACCACTTATAATTCTGGCCAGCCAAAACCACATTAATCCAGAGCCAATTAGCCGACAGCGCCTGTATATTACACTCCTTGCCTCACTACAAACCTTCTTAATTATAGCATTTGGCGCCACAGAAATTATTATATTCTACATCATATTTGAGGCCACACTTATCCCAACCCTTATTATTATCACTCGATGAGGAAATCAAACCGAACGACTCAATGCAGGAACCTACTTCCTATTTTACACCTTAGCAGGGTCACTCCCACTTTTAGTTGCCCTACTCCTCCTCCAACAAGCCACTGGAACACTATCAATATTGGTGCTTCAATATTCACAGCCCTTACAGCTCAACTCCTGAGGCCACATAATCTGGTGGGCTGGCTGCCTAATCGCATTTTTAGTCAAAATACCACTATATGGGGTTCACCTGTGATTACCAAAAGCGCATGTAGAAGCCCCTGTAGCAGGATCAATAGTCCTAGCAGCAGTTCTACTAAAACTTGGCGGATATGGAATAATACGTATAATAGTAATACTAGACCCCTTATCAAAAGAACTAGCCTACCCATTCATCATTCTAGCCCTCTGAGGCATTATCATGACAGGATCAATTTGCCTCCGACAAACAGACCTAAAGTCTCTAATTGCCTACTCATCTGTAAGCCATATAGGATTAGTAGCAGGAGGAATCCTAATCCAAACCCCATGGGGATTTTCAGGGGCAATTATTCTAATGATTGCCCATGGACTAGTATCCTCAGCACTATTTTGCCTGGCCAACACAGCATATGAACGAACCCACAGCCGAACAATAATCCTTGCCCGAGGACTACAAATGATTTTTCCCTTAACCACAATATGATGATTCATCGCCAACCTGGCTAACCTAGCCCTCCCTCCACTCCCTAACCTAATAGGAGAACTCATGATCATCACAACATTATTTAATTGATCCCCATGAACAATTCTACTCACCGGACTAGGAACATTAATTACAGCCGGCTACTCCTTATACATATTTCTTATATCACAACGAGGGCCAACACCAAACCATATCATAGGGCTCCAACCATTTCACACCCGAGAACATCTCCTAATAGCCCTACACCTAATCCCCGTTATTCTACTAGTAACAAAACCAGAACTTATATGAGGATGATGCTAT

>Spinibarbus_sinensis

ATGCTAAAAGTATTAATTCCCACAATTATGCTATTCCCAACAATTTGATTAACCTCCCCTAAATGACTGTGAACAACCACAACCGCCCACAGCCTCCTAATTGCTTTCATTAGCCTAACATGACTAAAATGGACATCCGAAACCGGATGGGCTTCCTCCAACACATACCTGGCCACAGACCCATTATCAACCCCCCTCCTAGTACTAACATGCTGATTACTTCCACTTATAATTTTAGCTAGCCAAAACCACATTAACCCCGAACCAGTTAGCCGACAACGCTCATATATTATACTCCTCGCCTCACTACAAACCTTTTTAATTATAGCATTCGGAGCTACAGAAATCATTATATTCTATATTATATTTGAAGCTACACCTATCCCAACCCTTATTATTATTACCCGGTGAGGAAACCAAACCGAACGACTTAACGCAGGAACCTATTTCTTATTCTATACTTTAGTAGGATCACTCCCACTTCTAGTTGCTTTACTCCTCCTTCAACAATCCACAGGAACGCTATCAATGTTGGTACTTCAATATTCACAACCCCTGCAACTCAATTCTTGAGGTCACATAATCTGATGAGCCGGCTGCCTAATCGCATTCTTAGTCAAAATACCATTATATGGAGTCCACCTATGACTACCAAAAGCGCATGTAGAGGCCCCCGTAGCAGGGTCAATAGTCCTAGCAGCAGTCTTACTAAAACTCGGAGGATACGGAATAATACGCATGATGGTGATACTAGACCCCCTATCAAAAGAACTAGCCTACCCATTCATCATTTTAGCCCTCTGGGGCATTATTATAACCGGGTCAATTTGTCTTCGACAAACAGACCTGAAATCACTAATTGCCTATTCATCTGTGAGCCACATGGGCCTAGTAGCAGGAGGAATCCTAATCCAAACCCCGTGGGGATTTTCAGGGGCAATCATTCTAATAATTGCCCACGGACTAGTATCCTCAGCACTATTCTGCCTAGCCAACACAGCATACGAACGAACCCACAGCCGAACAATAGTTCTTGCCCGAGGATTACAAGTGATTTTTCCATTAACCGCGGTATGATGATTCATTGCCAATCTAGCCAACCTAGCACTCCCACCACTGCCCAACTTAATAGGGGAACTCATAATTATTACAACATTATTCAACTGATCCCCATGAACAATTGTACTTACCGGACTAGGAACATTAATTACAGCCGGCTACTCCCTATACATATTCCTTATATCCCAACGAGGTCCAACACCAAACCACATTACAGGACTACAGCCATTTCACACCCGAGAACACCTACTAATAACTTTACACCTGATCCCCGTCATCCTACTAGTGACAAAACCAGAACTCATATGAGGATGATGCTAT

**ND4L**

>A._barbodon

ATGACACCCACACATTTTAGCTTTAGCTCAGCATTCATTCTAGGACTAATAGGATTAGCATTTCACCGTACCCATCTACTCTCAGCTCTCCTCTGCTTAGAGGGAATGATACTATCCTTATTTATTGCACTAGCCCTATGAGCACTACAATTCGAATCTACGGGGTTCTCAACAGCCCCTATGTTACTCTTAGCTTTCTCTGCTTGTGAAGCAAGCACTGGCCTAGCACTACTAGTTGCCACAGTTCGTACTCACGGAACTAACCGACTACAAAGCCTTAACCTCCTACAATGC

>A._beijiangensis_1

ATGACACCCGCACATTTCAGCTTTAGCTCAGCATTTATTCTGGGCTTAATAGGATTAGCATTTCACCGAGCCCACCTACTTTCTGCACTCCTATGCTTAGAAGGAATAATACTATCCCTGTTTATTGCACTAGCCCTATGGGCATTACAGTTCAAGGTTACAGGATTCTCAACAGCCCCCTTACTACTCCTAGCTTTTTCTGCTTGCGAAGCTAGCACCGGCCTAGCACTGCTAGTTGCCACAGTCCGGACTCACGGAACTAACCGACTACAAAGCCTTAATCTTTTACAATGT

>A._beijiangensis_2

ATGACACCCACACATTTCAGCTTTAGCTCAGCATTTATTCTGGGCCTAATAGGATTAGCATTTCACCGAGCCCACCTACTTTCTGCACTCCTATGCTTAGAAGGAATAATACTATCCCTGTTTATTGCACTAGCCCTATGGGCATTACAGTTCGAGGCCACAGGGTTCTCAACAGCTCCCTTACTACTCCTAGCTTTTTCTGCTTGCGAAGCTAGCACCGGCCTAGCACTGCTAGTTGCCACAGTCCGTACTCACGGAACTAACCGACTACAAAGCCTTAATCTTTTACAATGT

>A._fasciatus

ATGACACCCGCACATTTCAGCTTTAGCTCAGCATTTATCCTAGGCCTAATAGGACTAGCATTCCACCGAACCCATCTGCTTTCCGCACTCCTATGCTTAGAGGGAATAATATTATCCCTGTTCATCGCACTAGCCCTATGAGCATTACAATTCGAAGCTACAGGATTCTCAACAGCCCCCCTACTGCTCCTAGCTTTTTCTGCCTGTGAAGCAAGCACCGGCCTAGCATTACTAGTTGCCACAGTCCGCACCCACGGAACTAATCGCCTACAAAGCCTGAACCTTCTACAATGC

>A._hemispinus

ATGACACCCGCACATTTCAGCTTTAGCTCAGCATTTATTTTAGGCCTAATAGGGCTAGCATTCCACCGAACTCACCTGCTTTCCGCACTCCTATGCTTAGAAGGAATAATATTATCCCTGTTCATTGCATTAGCCCTATGGGCATTACAATTCGAAGCCACAGGATTTTCAACAGCCCCCATGCTACTCTTGGCTTTTTCTGCTTGTGAAGCAAGCACCGGTCTAGCACTGCTAGTTGCCACAGTTCGTACCCACGGTACCAACCGACTACAAAGCCTTAACCTCCTACAATGC

>A._iridescens

ATGACACCCGCACATTTTAGCTTTAGCTCAGCATTCATTCTAGGACTAATAGGATTAGCATTTCACCGTACCCACCTGCTCTCAGCTCTCCTCTGCTTAGAGGGAATGATACTATCCTTATTTATTGCACTAGCCCTGTGAGCACTACAATTCGAATCTACAGGGTTCTCAACAGCCCCTATGCTACTCTTAGCTTTCTCTGCTTGTGAAGCAAGCACTGGCCTAGCACTACTAGTTGCCACAGTTCGTACTCACGGAACCAACCGACTACAAAGCCTTAACCTCCTACAATGC

>A._jishouensis

ATGACACCCGCACATTTCAGCTTTAGCTCAGCATTTATTCTAGGCCTAATAGGACTAGCATTACACCGAACTCATCTGCTTTCCGCACTCCTATGCTTAGAAGGAATAATACTATCCCTGTTCATTGCGCTAGCCCTATGGGCACTACAATTCGAAGCCACAGGATTCTCAACAGCCCCTATACTACTCCTAGCTTTTTCTGCTTGTGAAGCAAGCACCGGTCTAGCACTGCTAGTTGCCACAGTTCGCACCCACGGAACTAATCGACTACAAAGCCTTAACCTTCTACAATGC

>A._kreyenbergii_1

ATGACACCCGCACATTTCAGCTTTAGCTCAGCATTTATTCTAGGCCTAATAGGGCTAGCATTCCACCGAACCCATCTACTTTCCGCACTCCTCTGCTTAGAGGGAATAATATTATCCCTGTTCATTGCACTAGCCCTGTGAGCACTACAATTCGAAGCCACAGGATTCTCAACAGCCCCTATACTACTCTTAGCTTTCTCTGCTTGTGAAGCAAGCACTGGCCTAGCATTACTAGTTGCCACAGTTCGCACCCACGGAACTAATCGCCTACAAAGCCTAAACCTTCTACAATGC

>A._kreyenbergii_2

ATGACACCCGCACATTTCAGCTTTAGCTCAGCATTTATTCTAGGCCTAATAGGGCTAGCATTCCACCGAACCCATCTACTTTCCGCGCTCCTATGCTTAGAGGGAATAATGTTATCCCTGTTCATTGCACTAGCCCTGTGAGCACTACAATTCGAGGCCACAGGATTCTCAACAGCCCCTATACTGCTCCTAGCTTTCTCTGCTTGTGAAGCAAGCACTGGCCTAGCATTACTAGTTGCCACAGTTCGCACCCACGGAACTAATCGCCTACAAAGCCTAAACCTTCTACAATGC

>A._longipinnis

ATGACACCCGCACATTTTAGCTTTAGCTCAGCATTCATTCTAGGACTAATAGGATTAGCATTTCACCGTACCCACCTGCTCTCAGCTCTCCTCTGCTTAGAGGGAATGATACTATCCTTATTTATTGCACTAGCCCTGTGAGCACTACAATTCGAATCTACAGGGTTCTCAACAGCCCCTATGCTACTCTTAGCTTTCTCTGCTTGTGAAGCAAGCACGGGCATACCACAACTAGTTGCCACAGTTCGAACTCACGGAACTAACCGACTACAAAGCCTTAACCTCCTACAATGC

>A._monticola_1

ATGACACCCGCACATTTTAGCTTCAGCTCAGCATTTATTCTAGGCCTAATAGGACTAGCATTTCACCGAACCCACCTACTCTCGGCACTTCTATGCCTAGAAGGAATAATACTATCCCTATTCATTGCACTAGCCCTATGAGCACTACAATTTGAATCTACAGGATTCTCAACAGCCCCCATGCTACTCCTAGCTTTTTCTGCCTGTGAAGCTAGCACCGGCCTAGCACTGCTAGTTGCCACAGTTCGTACTCACGGAACTAACCGATTACAAAGCCTTAACCTTCTACAATGC

>A._monticola_2

ATGACACCCGCACATTTTAGCTTCAGCTCAGCATTTATTCTAGGCCTAATAGGACTAGCATTTCACCGAACCCACCTACTCTCGGCACTTCTATGCCTAGAAGGAATAATACTATCCCTATTCATTGCACTAGCCCTATGAGCACTACAATTTGAATCTACAGGATTCTCAACAGCCCCCATGCTACTCCTAGCTTTTTCTGCCTGTGAAGCTAGCACCGGCCTAGCACTGCTAGTTGCCACAGTTCGTACTCACGGAACTAACCGATTACAAAGCCTTAACCTTCTACAATGC

>A._paradoxus_1

ATGACACCCACACATTTCAGCTTTAGCTCAGCATTTATCCTAGGCCTAATAGGATTAGCATTTCACCGAACCCACTTACTCTCCGCACTCCTGTGCTTAGAAGGAATAATATTATCCCTATTCATCGCACTAGCCTTATGAGCACTACAATTCGAGGCTACAGGATTCTCAACAGCCCCCCTACTACTCCTAGCTTTTTCTGCTTGCGAAGCTAGCACCGGCCTAGCACTGCTAGTTGCCACAGTCCGTACTCACGGAACTAACCGACTACAAAGCCTCAATCTTTTACAATGT

>A._paradoxus_2

ATGACACCCGCACATTTCAGCTTTAGCTCAGCATTTATTCTAGGCCTAATAGGACTAGCATTACACCGAACTCATCTGCTTTCCGCACTCCTATGCTTAGAAGGAATAATACTATCCCTGTTCATTGCGCTAGCCCTATGGGCACTACAATTCGAAGCCACAGGATTCTCAACAGCCCCTATACTACTCCTAGCTTTTTCTGCTTGTGAAGCAAGCACCGGTCTAGCACTGCTAGTTGCCACAGTTCGCACCCACGGAACTAATCGACTACAAAGCCTTAACCTTCTACAATGC

>A._parallens_1

ATGACACCCGCACATTTCAGCTTTAGCTCAGCATTTATTCTAGGCCTGATAGGGCTAGCATTCCACCGAACTCACCTGCTTTCCGCACTCCTATGCTTAGAAGGAATAATACTATCCCTGTTCATTGCATTAGCCCTATGGGCATTACAATTCGAAGCCACAGGATTTTCAACAGCCCCTATACTACTCTTGGCTTTTTCTGCTTGTGAAGCAAGCACCGGTCTAGCACTGCTAGTTGCCACAGTTCGTACCCACGGTACCAACCGACTACAAAGCCTTAACCTCCTACAATGC

>A._parallens_2

ATGACACCCGCACATTTCAGCTTTAGCTCAGCATTTATTCTAGGCCTAATAGGACTGGCATTCCACCGAACTCACCTGCTTTCCGCACTCCTATGCTTAGAAGGAATAATACTATCCCTGTTCATTGCGTTAGCCCTGTGGGCATTACAATTCGAAGCCACAGGATTTTCAACAGCCCCTATACTACTCTTGGCTTTTTCTGCTTGTGAAGCAAGCACCGGTCTAGCACTGCTAGTTGCCACAGTTCGTACCCACGGTACCAACCGACTACAAAGCCTTAACCTCCTACAATGC

>A._parallens_3

ATGACACCCGCACATTTCAGCTTTAGCTCAGCATTTATTCTAGGCCTAATAGGACTAGCATTCCACCGAACTCACCTGCTTTCCGCACTCCTATGCTTAGAAGGAATAATACTATCCCTATTCATTGCGTTAGCCCTATGGGCATTACAATTCGAAGCCACAGGATTTTCAACAGCCCCTATACTGCTCTTGGCTTTTTCTGCTCGTGAAGCAAGCACCGGTCTAGCACTGCTAGTTGCCACAGTTCGTACCCACGGTACCAACCGACTACAAAGCCTTAACCTCCTACAATGC

>A._parallens_4

ATGACACCCGCACATTTCAGCTTTAGCTCAGCATTTATTCTAGGCCTAATAGGGCTAGCATTCCACCGAACTCACCTGCTTTCCGCACTCCTATGCTTAGAAGGAATAATACTATCCCTATTCATTGCATTAGCCCTATGGGCATTACAATTCGAAGCCACAGGATTTTCAACAGCCCCTATACTGCTCTTGGCTTTTTCTGCTTGTGAAGCAAGCACCGGTCTAGCACTGCTAGTTGCCACAGTTCGTACCCACGGTACCAACCGACTACAAAGCCTTAACCTCCTACAATGC

>A._spinifer

ATGACACCCACACATTTCAGCTTTAGCTCAGCATTTATTCTGGGCCTAATAGGATTAGCATTTCACCGAGCCCACCTACTTTCTGCACTCCTATGCTTAGAAGGAATAATACTATCCCTGTTTATTGCACTAGCCCTGTGGGCACTACAGTTCGAGGCCACAGGGTTTTCAACAGCTCCCTTACTACTCCTAGCTTTTTCTGCTTGTGAAGCTAGCACCGGCCTAGCACTGCTAGTTGCCACAGTCCGTACTCACGGAACTAACCGACTACAAAGCCTTAATCTTTTACAATGT

>A._stenotaeniatus

ATGACACCCACACATTTCAGCTTTAGCTCAGCATTTATTCTGGGCCTAATAGGATTAGCATTTCACCGAGCCCACCTACTTTCTGCACTCCTATGCTTAGAAGGAATAATACTATCCCTGTTTATTGCACTAGCCCTGTGGGCACTACAGTTCGAGGCCACAGGGTTTTCAACGGCTCCCTTACTACTCCTAGCTTTTTCTGCTTGTGAAGCTAGCACCGGCCTAGCACTGCTAGTTGCCACAGTCCGTACTCACGGAACTAACCGACTACAAAGCCTTAATCTTTTACAATGT

>A._wenchowensis_1

ATGACACCCGCACATTTCAGCTTTAGCTCAGCATTTGTCCTAGGCCTAATAGGACTAGCACTCCACCGAACCCATCTGCTTTCTGCACTCCTATGCCTAGAGGGAATAATATTATCCCTATTCATTGCACTAGCCCTGTGGGCGTTACAATTCGAGGCTACAGGATTCTCAACAGCCCCCATACTACTCTTAGCTTTTTCTGCTTGTGAAGCAAGCACCGGCCTAGCATTACTAGTTGCCACAGTTCGCACCCACGGAACTAACCGCCTACAAAGCCTAAACCTTCTACAATGC

>A._wenchowensis_2

ATGACACCCGCACATTTCAGCTTTAGCTCAGCATTTATCCTAGGCCTAATAGGGCTAGCATTCCACCGAACCCACCTGCTTTCCGCACTTCTATGCTTAGAGGGAATAATATTATCCCTGTTCATCGCACTAGCCCTGTGAGCATTACAATTCGAAGCCACAGGATTCTCAACAGCCCCCATACTACTCCTAGCTTTTTCTGCCTGTGAAGCAAGCACCGGCCTAGCATTACTAGTTGCCACAGTTCGCACCCATGGAACTAATCGCCTACAAAGCCTAAACCTTCTACAATGC

>A._wuyiensis

ATGACACCCACACATTTCAGCTTTAGCTCAGCATTTATCCTAGGCCTAATAGGATTAGCATTTCACCGAACCCACTTACTCTCCGCACTCCTATGCTTAGAAGGAATAATATTATCCCTATTCATCGCACTAGCCTTATGAGCGCTACAATTTGAGGCTACAGGATTCTCAACAGCCCCCCTACTACTCCTAGCTTTTTCTGCTTGTGAAGCTAGCACCGGCCTAGCACTGCTAGTTGCCACAGTCCGTACTCACGGAACTAACCGACTACAAAGCCTCAATCTTTTACAATGT

>A._yunnanensis_1

ATGACACCCGCACATTTTAGCTTTAGCTCAGCATTTATTCTAGGTCTAATAGGACTAGCATTTCACCGAACCCACTTACTATCAGCACTTCTATGCCTAGAAGGAATAATATTGTCCCTATTTATCGCACTAGCCCTATGAGCACTACAATTTGAATCTACAGGATTCTCAACAGCCCCTATGCTACTCCTAGCTTTTTCTGCTTGTGAAGCAAGCACCGGCCTAGCACTGCTAGTTGCCACAGTTCGTACTCACGGAACTAACCGACTACAAAGCCTCAACCTCCTACAATGC

>A._yunnanensis_2

ATGACACCCGCACATTTTAGCTTTAGCTCAGCATTTATTCTAGGTCTAATAGGACTAGCATTTCACCGAACCCACCTACTATCAGCACTTTTATGCCTAGAAGGAATAATATTATCCCTATTTATTGCACTAGCCCTATGGGCACTACAATTTGAATCTACAGGATTCTCAACAGCCCCTATGCTACTCCTAGCTTTTTCTGCTTGTGAAGCAAGCACCGGCCTAGCACTGCTAGTTGCCACAGTTCGTACTCACGGAACTAACCGACTACAAAGCCTCAACCTCTTACAATGC

>Onychostoma_barbatulum

ATGACACCCGCACATTTTAGCTTTAGCTCAGCATTTATTCTAGGTCTAATAGGATTAGCATTTCACCGAACCCATCTGCTCTCTGCACTTCTATGCTTAGAAGGAATAATGCTGTCATTATTTATTGCACTGGCCCTATGAGCTCTACAATTTGAATCTACGGGATTCTCAACAGCCCCTATGCTACTTTTAGCCTTTTCTGCTTGTGAAGCTAGCACCGGCCTAGCACTACTAGTTGCCACAGTCCGTACTCACGGAACTAACCGTCTACAAAGCCTTAATCTTCTGCAATGC

>Onychostoma_meridionale

ATGACACCCACACATTTTAGCTTTAGCTCAGCATTTATCTTAGGTCTGATAGGATTAGCATTTCACCGAACCCACCTACTCTCAGCACTCCTCTGCTTAGAAGGAATAATACTATCCCTATTTATTGCACTAGCCCTGTGGGCGCTACAATTTGAATCCACCGGATTCTCAACAGCCCCCATGCTACTACTAGCTTTTTCCGCCTGCGAGGCAAGCACCGGCCTAGCTCTACTAGTTGCTACAGTTCGCACTCACGGGACCAACCGACTACAAAGCCTCAACCTCCTACAATGC

>Onychostoma_gerlachi

ATGACACCCACACATTTCAGCTTTAGCTCAGCATTCATCCTAGGTCTGATAGGGTTAGCATTTCACCGAACCCACCTGCTCTCAGCACTCCTCTGCTTAGAAGGAATAATATTATCCCTATTTATTGCACTAGCCCTGTGGACACTACAATTTGAATCCACTGGATTCTCAACAGCCCCTATGCTACTCTTAGCTTTTTCTGCTTGTGAAGCTAGCACCGGCCTAGCACTTCTAGTTGCTACAGTTCGCACTCACGGAACTAACCGACTACAAAGCCTCAACCTTCTACAATGC

>Spinibarbus_denticulatus

ATGACACCCGTACATTTTAGCTTTACCTCAGCATTTATTCTAGGCCTAATAGGACTAGCATTTCACCGAACCCACCTTCTCTCAGCCCTCCTATGCTTAGAAGGAATAATATTATCTTTGTTTATTGCACTCGCCCTATGAGCACTACAATTTGAATCCACAGGATTTTCAACAGCCCCTATACTACTCCTAACCTTTTCTGCCTGCGAAGCAAGCACTGGCTTAGCACTACTAGTTGCTACTGCTCGCACTCACGGAACTGACCGTCTACAAAACCTCAATCTCCTACAATGT

>Spinibarbus_hollandi

ATGACACCCGTACATTTTAGCTTTACCTCAGCATTTATTCTAGGCCTAATAGGACTAGCATTTCACCGAACCCATCTTCTCTCAGCCCTCCTATGCTTAGAAGGGATAATATTATCTTTGTTTATTGCACTCGCCCTATGAGCACTACAATTCGAATCCACAGTATTTTCAACAGCCCCTATACTACTTCTAACCTTTTCTGCTTGTGAAGCAAGCACTGGCTTAGCACTACTAGTTGCTACTGCCCGCACCCATGGGACTGACCGGTTACAAAACCTTAACCTCCTACAATGC

>Spinibarbus_sinensis

ATGACACCCGTACATTTTAGCTTTAGCTCAGCATTTATCCTAGGCCTAATAGGACTAGCATTCCACCGAACCCATTTACTCTCAGCACTCCTGTGCTTAGAAGGAATAATACTATCCCTGTTTATTGCACTGGCCCTATGAGCATTACAATTTGAATCTACAGGATTCTCAACAGCCCCTATATTACTCTTAGCCTTCTCTGCTTGTGAAGCAAGCACCGGTCTGGCACTACTAGTTGCCACTGCTCGCACCCACGGAACTGACCGACTACAAAACCTCAATCTTCTACAATGC

**ND5**

>A._barbodon

ATGACACTAATCATACACTCGTCTCTCCTCCTAACCTTTCTCATCCTACTACACCCACTACTTACCACACTTAAACCCAACCAACAAGAATCCAACATAGCAGAAACTACTAAGACCGCCATTAGCTCCGCATTCTTCATCAGCCTCCTGCCACTTACAATTTTTCTAACCCAAGGAACAGAAAGCATCGTAACAAACTGACAATGAATAAATACCCAAACATTTGACGTAAACATCAGCTTTAAATTCGACCACTACTCCTTAGTTTTTATCCCAATCGCCCTATACGTCACCTGATCAATCCTCGAGTTTTCACTATGATACATACACTCTGACCCCAACATTAACCGGTTCTTCAAATATCTACTAGTATTCCTAGTAGCTATGATTATCCTAGTCTCAGCTAATAACATCTTTCAACTATTCATTGGCTGGGAAGGAGTAGGAATTATATCGTTCCTCCTCATCGGGTGATGATATGGCCGGGCAGATGCTAACACAGCAGCCCTTCAAGCCGTTATTTACAATCGAGTAGGAGACATCGGACTAATTATAACCACAGCTTGGTTCGCAATAAACCTTAACTCCTGAGAAATTCAACAAATCTTAACACTATCAAAAGACTTTAACATAACATTTCCCCTAATTGGGCTCATCTTAGCAGCAACAGGAAAGTCCGCCCAATTCGGCCTCCACCCTTGACTTCCAGCCGCCATAGAGGGCCCTACCCCAGTATCTGCCCTACTACACTCAAGCACTATAGTTGTTGCAGGAATCTTCCTACTAATCCGTTTTCACCCCCTCACAGAAAATAACCAGCTGGCATTAACCACCTGCCTATGCCTTGGAGCACTAACCTCACTATTCGCAGCCACCTGCGCCCTCACCCAAAATGATATCAAAAAAATTGTGGCTTTCTCAACATCAAGCCAGCTAGGCCTAATAATAGTTGCAATTGGATTAAACCAACCACAACTAGCATTCCTCCATATCTGCACCCATGCCTTCTTCAAAGCCATGTTATTCCTATGCTCAGGTTCAATTATTCATAGCCTAAACGACGAACAAGACATCCGAAAAATAGGAGGCCTATTTAATACCATACCCACCACCTCTGCCTACTTCACAATCGGCAGCCTAGCCTTAACAGGAACCCCCTTCCTAGCAGGATTCTTTTCAAAAGACGCAATCATTGAAGCCCTAGGTACCTCCTATCTTAACGCCTGAGCCCTAACCCTAACACTAATTGCTACATCATTCACTGCAGTTTATAGCTTCCGTCTAGTATACTTCGTAATCATGGGAGCCCCACGATTTCTACCCCTATGCCCAATTAACGAAAACAATCCACTAGTAATTAACCCCCTCAAACGACTTGCCTGAGGAAGCATCACCGCGGGACTTATCATCACCCAAAACCTCACACCAGTAAAAACACCAATCATAACAATACCCGCCCCCCTAAAAATAGCAGCCCTCCTAGTAACAATTATAGGCCTATTAACAGCCATAGAACTGGCTAATATAACAAGTAAGCAAGTAAAAATTACCCCAACAATTCGTACACACCACTTCTCAAACATACTAGGATTCTTCCCCATAACCGTACATCGAATTGTTCCAAAACTTAAACTTATCCTAGGACAATCAGCCGCTACCCAACTTGATAAAACATGACTCGAAGCAATTGGGCCAAAAGGCCTAGCATTAACACAAAAGAACATGGCAAAAGCTACAAATAATATCTCACGAGGAATAATTAAAACATACCTAACCATCTTCCTCCTAACCCTAATCTTAACCACCACCCTAGTACTTCTTTAA

>A._beijiangensis_1

ATGACACTAATCATACACTCATCCCTTCTCCTCACCTTTCTTATTCTTCTCCACCCACTACTCACTACACTCAACCCAGACCAAAAAGGGTCTAACATGGCAGAAACCACCAAAACTGCCATCAGCACCGCCTTCTTCATTAGCCTCCTACCTCTTGTAATTTTCCTGGCCCAAGGAACAGAAAGCATCGTAACAAACTGACAATGAATAAACACCCAAACATTTGATGTAAATATCAGCTTTAAATTCGACCACTACTCCCTAATCTTCATCCCAATTGCCCTATATGTCACTTGATCAATCCTTGAATTTTCACTATGATATATGCACTCCGACCCCAACATCAACCAATTCTTCAAATATCTCCTCGTATTCTTAGTGGCCATAATCGTCCTAGTCTCAGCTAACAACATTTTCCAACTATTTATTGGTTGAGAGGGAGTAGGGATTATATCATTCCTCCTCATCGGATGATGATATGGCCGAACAGACGCTAATACAGCCGCCCTCCAAGCCGTTATTTATAACCGAGTAGGAGACATCGGACTAATTATAACTATAGCTTGGTTCGCAATAAACCTCAACTCCTGAGAAATTCAACAAACCCTGACATTATCAAAAGACTTTAACATAACACTTCCCCTAATTGGACTCATCTTAGCAGCAACAGGAAAATCCGCCCAATTCGGCCTTCACCCCTGACTCCCAGCCGCTATAGAAGGCCCTACCCCAGTATCTGCCCTACTCCACTCAAGCACCATGGTTGTTGCAGGAGTCTTCCTATTAATCCGCTTTCATCCCCTTATAGAAAATAACCAACTGGCACTAACAACTTGTCTCTGCCTTGGAGCACTAACCTCATTATTCGCAGCCACCTGCGCCCTCACCCAAAATGACATCAAGAAAATTGTAGCTTTCTCAACATCAAGCCAACTAGGCCTAATAATGGTTGCAATCGGACTAAACCAACCACAACTAGCATTCCTTCACATCTGCACCCACGCCTTCTTCAAAGCTATACTATTCCTGTGTTCAGGGTCAATTATTCACAGCCTAAATGATGAACAAGACATCCGAAAGATGGGAGGCCTACTCAACATAATGCCTACCACCTCTACTTACTTTACAATCGGCAGCCTAGCCTTGACAGGTACCCCATTCCTAGCAGGATTCTTCTCAAAAGACGCGATCATTGAAGCCCTAAATACCTCATCCCTAAACGCCTGAGCCCTTGTCCTAACGCTAATTGCTACCGCATTCACGGCAGTATATAGCTTCCGTCTACTACACCTTGTAATCATAGGAGCCCCCCGATTCCTGCCCCTATGCCCTATTAATGAAAACAACCCATTAGTAATTAACCCCCTCAAACGACTTGCCTGGGGAAGCATCACCGCAGGATTTATTATTATTCACAATCTTATGCCAATAAAAACACCAGTCATAACTATACCAACCTCCTTAAAAATAGCAGCTATCCTAGTAACAATTGCAGGTCTGCTGACAGCCATAGAACTGGCCAACATAACAACTAAACAAGTAAAAATTATCCCAACAACTTATTCACACCACTTCTCAAATATACTGGGATTCTTCCCCACAATTATACACCGGCTTATTCCTAAACTCAAACTTACCCTGGGACAATCAGCCGCCACCCAACTCGACAAAACATGGCTAGAATCGATCGGACCTAAAGGCCTGGCACTAGCACAAAAAAACATAGCAAAAGCCACAAATAACATCTCACGAGGAATAATTAAAACGTATCTAACCATTTTCCTCTTAACCCTAATCCTAGCCACTATCTTAATCTCACTTTAA

>A._beijiangensis_2

ATGACACTAATCATACACTCATCCCTTCTCCTCACCCTTCTTATTCTTCTCCACCCACTACTCACTACACTCAACCCAGACCAACAAGGGTCTAACATGGCAGAAACCACCAAAACTGCCATCAGCACCGCCTTCTTCGTTAGCCTCCTACCTCTTGTAATTTTTCTAGCCCAAGGAACAGAAAGCATCGTAACAAACTGACAATGGATAAACACCCAAACATTTGACGTAAACATCAGCTTTAAATTCGACCACTACTCCCTAATCTTCATCCCAATTGCCCTATATGTCACTTGATCAATCCTTGAATTCTCACTATGATACATGCACTCCGACCCCAACATCAACCAATTCTTCAAATATCTCCTCGTATTCTTAATAGCCATAATCATCCTAGTCTCAGCTAACAACATTTTCCAACTATTCATTGGTTGAGAAGGAGTAGGGATTATATCATTCCTCCTCATCGGATGATGATACGGCCGGACAGACGCTAATACAGCCGCCCTCCAAGCCGTTATTTACAACCGAGTGGGAGACATCGGACTAATTATAACTATAGCTTGGTTCGCAATAAACCTCAACTCCTGAGAAATTCAACAAATCCTGACATTATCAAAAGACTTTAACATAACACTTCCCCTAATTGGGCTCATCTTAGCAGCAACAGGAAAATCCGCCCAATTCGGCCTTCACCCCTGACTCCCAGCCGCCATAGAAGGCCCTACCCCAGTATCTGCCCTACTCCACTCAAGCACCATGGTTGTTGCAGGAGTCTTCCTATTAATCCGCTTTCACCCTCTTATGGAAAATAACCAACTAGCACTAACAACTTGTCTCTGCCTTGGAGCACTAACCTCATTATTTGCAGCCACCTGCGCCCTCACTCAAAATGACATCAAGAAAATTGTAGCTTTCTCAACATCAAGCCAGCTAGGCCTAATAATAGTTGCAATCGGACTAAACCAACCACAACTAGCATTCCTTCACATCTGCACCCACGCCTTCTTCAAAGCTATACTATTCCTGTGTTCAGGATCAATTATTCACAGCCTGAATGATGAACAAGACATCCGAAAGATGGGAGGCCTACTCAACATAATGCCTGCCACCTCTGCTTACTTCACAATCGGTAGCCTAGCCTTAACAGGTACCCCATTCCTAGCAGGATTCTTCTCAAAAGACGCAATCATTGAAGCCCTAAATACCTCATCCCTAAACGCCTGAGCCCTTGTCCTAACGCTAATTGCTACCGCATTCACGGCAGTATATAGCTTCCGTCTACTACACCTTGTAATCATAGGAGCCCCCCGATTCCTACCCCTATGCCCTATTAATGAAAACAACCCATTGGTAATTAACCCCCTCAAACGACTTGCCTGAGGAAGCATCACCGCAGGATTTATTATTACTCATAATCTTATGCCAATAAAAACACCAGTCATAACTATACCAACCTCCTTAAAGATAGCAGCTATCCTAGTAACAATTGCAGGTCTGCTGACAGCCATAGAACTGGCTAACATAACAAGTAAACAAGTAAAAATTATCCCAACAACTTATACACACCACTTCTCAAATATACTGGGATTCTTCCCCACAATTATACACCGACTTATCCCCAAACTCAAACTTACCCTAGGACAGTCAGCCGCCACCCAACTCGACAAAACATGACTAGAAACGATCGGACCAAAAGGCCTGGCACTAGCACAAAAAAACATAGCAAAAGCTACAAATAACATCTCCCGAGGAATAATTAAAACGTATCTAACCATCTTCCTCTTAACCCTAATCCTAGCCACTATCTTAATCTCACTTTAA

>A._fasciatus

ATGACACTAATCATACACTCGTCCCTTCTCCTAACCCTCCTTATCTTACTTCACCCACTACTCACCACACTTAATCCAAACCAACAAGAGTCTAATATGGCAAAAACCACTAAAACTGCCATTAGCTCAGCCTTCCTCATCAGCCTCCTGCCCCTTACAATTTTCCTGGCTCAAGGAGCAGAAAGTATCGTAACAAACTGACAATGAATAAACACCCAAACATTCGATGTAAACATCAGCTTTAAATTTGATCACTACTCCTTAATCTTTATCCCAATTGCCTTATACGTCACCTGATCAATTCTTGAATTTTCACTCTGATACATGCACTCCGACCCCAACATCAACCAATTCTTTAAATACCTACTTGTATTCTTAGTAGCCATAATTATCTTAGTCTCAGCTAACAATATCTTCCAACTATTCATCGGCTGAGAAGGAGTAGGAATCATGTCCTTCCTTCTCATCGGGTGATGATATGGCCGGGCGGATGCTAACACAGCCGCCCTCCAAGCCGTTATTTATAACCGAGTAGGGGACATCGGACTAATTATAGCTATAGCCTGGTTCGCAATAAACCTCAACTCCTGAGAAATCCAACAAATCTTAACTCTATCAAAAGACTTTGATATAACACTTCCCCTAATTGGACTCATCTTAGCAGCAACAGGAAAATCCGCCCAATTTGGCCTTCACCCCTGACTCCCAGCCGCCATGGAAGGCCCTACCCCAGTGTCTGCCCTACTCCACTCAAGCACCATGGTTGTTGCAGGAATCTTCCTGCTAATCCGCCTTCACCCTCTCATAGAGAATAATCAACTGGCACTGACAACTTGTCTCTGCCTTGGAGCACTAACCTCGCTATTCGCAGCCACCTGCGCCCTCACCCAAAATGATATCAAAAAAATTGTAGCTTTCTCAACATCAAGCCAGCTAGGCCTAATAATAGTCGCAATCGGGTTAAACCAACCACAACTAGCATTCCTTCATATCTGCACCCACGCCTTTTTCAAGGCTATACTATTCCTGTGCTCAGGGTCGATTATTCACAGCCTAAATGACGAACAAGACATCCGAAAGATAGGAGGCCTGCTCGACATTATGCCCACCACCTCTACTTACTTCACAATTGGTAGCCTAGCCCTAACAGGAACCCCCTTCTTAGCAGGGTTCTTCTCAAAAGACGCAATCATCGAGGCCCTAAACTCTTCATCCCTAAACGCCTGAGCCCTTATCCTGACATTAATTGCCACATCTTTTACAGCTGTATATAGTTTCCGCCTAGTATATTTTGTAATCATAGGAGCTCCCCGATTCCTGCCCCTATGCCCGATTAACGAAAACAACCCATTAGTAATCAACCCCCTCAAACGACTTGCCTGAGGAAGCATCACCGCAGGACTAATTATCACCCAAAACTTTTTACCCATAAAAACACCAGTTATAACAATGCCAACCCCCTTAAAAATAGCGGCTCTCCTAGTAACAATTACGGGCCTGCTAACAGCCATAGAACTCGCCAACATAACAAGTAAACAAGTGAAGATTACCCCAATAACCCACACACATCACTTCTCAAACATACTAGGATTCTTCCCTATAATTACACATCGACTAATCCCAAAACTTAAACTCACCCTGGGACAATCAGCCGCCACCCAACTCGACAAAACGTGGCTCGAAACAATTGGACCAAAAGGCCTAGCACTAACACAAAAAAACATAGCAAAAGCCACAAATAACATCTCACGAGGAATAATTAAAACATACCTAACCATCTTCCTCTTAACTCTAATCCTAGCCACCATCCTAACTTCACTTTAA

>A._hemispinus

ATGACACTAATCATACACTCGTCCCTTCTCCTAACCCTTCTTATTCTACTCCACCCACTAATCACCACACTTAACCCAAACCAACAAGGATCAAATATGGCAGACATCACTAAAACTGCCATTAGTTTAGCCTTCTTCACCAGCCTCTTACCTCTTACAATTTTCCTAACCCAGGGGGCAGAAAGCATCGTAACAAACTGACAATGAATAAACACCCAAACATTTGATGTAAACATCAGCTTTAAATTTGACCACTACTCCTTGATCTTTATCCCTATTGCCTTATACGTCACCTGATCAATCCTTGAATTCTCGCTATGATACATGCACTCCGACCCCAACATCAACCAATTCTTTAAGTACCTACTTGTATTCTTAGTAGCCATAATTATCCTAGTTTCAGCTAACAACATCTTCCAACTATTTATCGGCTGAGAAGGGGTTGGAATTATATCTTTCCTACTTATCGGATGATGATATGGCCGGGCGGATGCTAACACAGCCGCCCTCCAAGCCGTTATTTACAACCGAGTAGGGGACATCGGACTAATTATAACCATAGCCTGGTTCGCAATAAACCTCAACTCCTGAGAAATTCAACAAATCCTTACTCTATCAAAAGATTTTAATATAACACTTCCCCTAATTGGACTCATCTTAGCAGCAACAGGAAAATCCGCCCAATTTGGCCTTCACCCCTGACTTCCAGCCGCCATAGAAGGCCCTACCCCAGTATCTGCCCTACTTCACTCAAGCACCATGGTCGTTGCGGGAATCTTTCTACTAATCCGCTTTCACCCCCTTATAGAAAATAATCAACTACTACTGACAACCTGTCTCTGTCTCGGAGCACTAACCTCACTATTCGCAGCCACCTGCGCCCTTACCCAAAATGACATCAAAAAAATTGTAGCTTTCTCAACATCAAGCCAGCTGGGCCTAATAATAGTCGCAATCGGACTAAACCAACCACAACTAGCATTCCTCCATATCTGTACCCACGCCTTTTTCAAAGCTATATTATTTCTGTGCTCAGGATCAATTATTCACAGCCTAAATGACGAACAAGACATCCGAAAAATAGGAGGCCTACTCAACATTATACCTACCACCTCTACTTATTTCACAATCGGCAGCTTAGCCCTAACAGGGACCCCCTTCCTAGCAGGGTTCTTCTCAAAAGACGCAATCATTGAAACCCTAAACTCCTCATCCCTAAACGCCTGAGCCCTTATCCTGACATTAATTGCCACATCTTTCACAGCTGTATATAGTTTCCGCCTAGTATATTTTGTAATCATAGGGGCCCCCCGATTCCTACCCCTATGCCCGATCAACGAAAACAACCCATTAGTAATTAACCCCCTCAAACGACTTGCCTGAGGAAGCATCACCGCAGGACTAATTATTACCCAAAACTTTTTACCAATAAAAACACCCGTTATAACAATACCAACCCCCCTAAAAATAGCGGCTCTCCTAGTAACAATTGTAGGCCTGCTGACAGCCATAGAACTGGCCAACATAACAAGTAAACAGGTGAAAATCACCCCAACAACCCATACACACCACTTCTCCAATATACTAGGATTTTTCCCCATAGTTATGCATCGACTAGTCCCAAAACTCAAACTCACCCTAGGGCAGTCAGCCGCCACCCAACTCGACAAAACATGACTCGAAACAATTGGACCAAAAGGCCTAGCACTAACACAAAAAAGTATAGCAAAAGCTACAAATAATATCTCACGAGGAATAATTAAAACATATCTAACCATCTTCCTCCTAACCCTAATCTTAGCCACTATCCTGGTTTCACTTTAA

>A._iridescens

ATGACACTAATCATACACTCGTCCCTCCTCCTAACCTTTCTCATTCTACTACACCCACTACTTACCACACTTAAACCCAACCAACAAGAATCCAACATAGCAGAAACTACTAAGACCGCCATTAGCTCCGCATTCTTCATCAGCCTCCTACCACTTACAATTTTTCTAACCCAAGGAACAGAAAGCATCGTGACGAACTGACAATGAATAAATACCCAAACATTTGACGTAAACATCAGCTTTAAATTCGACCACTATTCCTTAATTTTTATCCCAATCGCCTTATACGTCACCTGATCAATCCTCGAGTTTTCACTATGATACATACACTCTGACCCCAACATTAACCGGTTCTTCAAATATTTACTAGTATTCCTAGTAGCTATAATTATCCTGGTCTCAGCTAATAACATCTTTCAACTATTCATTGGCTGAGAAGGAGTAGGAATTATATCATTCCTCCTTATCGGGTGATGATATGGCCGGGCAGATGCTAACACAGCAGCCCTTCAGGCCGTTATTTACAATCGAGTAGGAGACATCGGACTAATTATAACCACAGCTTGGTTCGCAATAAACCTTAACTCCTGAGAAATTCAACAAATCTTAACACTATCAAAAGACTTTAACATAACATTTCCCCTAATTGGGCTCATCTTAGCAGCAACAGGAAAATCCGCCCAATTCGGCCTCCACCCTTGACTCCCAGCCGCCATAGAAGGCCCTACCCCAGTGTCTGCCCTACTCCACTCAAGCACTATAGTTGTTGCAGGAATCTTCCTATTAATCCGTTTTCACCCCCTCACAGAAAATAACCAGCTGGCATTAACCATCTGCCTCTGCCTTGGAGCACTAACCTCATTATTCGCAGCCACCTGCGCCCTCACCCAAAATGATATCAAAAAAATTGTGGCTTTCTCAACATCAAGCCAGCTAGGCCTAATAATAGTCGCAATTGGATTAAACCAACCACAACTAGCATTCCTCCATATCTGCACCCATGCCTTCTTCAAAGCCATGTTATTCCTGTGCTCAGGTTCAATTATTCATAGCCTAAACGATGAACAAGACATCCGAAAAATAGGAGGCCTATTTAACACCATACCCACCACCTCTGCCTACTTCACAATCGGCAGCCTAGCCTTAACAGGAACCCCCTTCCTGGCAGGATTCTTCTCAAAAGACGCAATCATTGAAGCCCTAGGTACCTCCTATCTTAACGCCTGAGCCCTAACCCTAACACTAATTGCTACATCATTCACTGCAGTCTATAGCTTCCGTCTAGTATACTTCGTAATCATGGGAGCCCCACGATTTCTACCCCTATGCCCAATTAACGAAAACAATCCACTAGTAATTAACCCCCTCAAGCGACTTGCCTGAGGAAGCATCACCGCAGGACTTATCATCACCCAAAACCTCACACCAATAAAAACACCAATCATAACAATACCCGCCCCCCTAAAAATAGCAGCCCTCCTAGTAACAATTATAGGCCTATTAACAGCCATAGAACTGGCTAACATAACAAGTAAACAAGTAAAAATTACCCCAACAATCCATACACACCACTTCTCAAATATACTAGGATTCTTCCCCATAACTGTACATCGAATTGTTCCAAAACTTAAACTTATCCTAGGACAATCAGCCGCTACCCAACTCGATAAAACATGACTCGAAGCAATTGGGCCAAAAGGCCTAGCACTAACACAAAAGAACATGGCAAAAACTACAAATAATATCTCACGAGGAATGATTAAAACATACTTAACCATCTTCCTCCTAACCTTAATCTTAGCCACCACCCTAGTACTTCTTTAA

>A._jishouensis

ATGACACTAATCATACACTCGTCCCTCCTCCTAACCCTTATTATCCTACTCCACCCACTACTCACCACACTTAACCCAAACCAACAAGGATCTAATATAGCAAAAACCACTAAAACTGCCATTAGTTTAGCCTTCTTCATCAGCCTCCTACCTCTTACAATTTTCCTAGCCCAGGGGGCAGAAAGCATCGTAACAAACTGACAATGAATAAATACCCAAACATTTGATGTAAACATCAGCCTTAAATTTGATCACTACTCCTTAATCTTTATCCCAATTGCCTTATATGTCACCTGATCAATCCTTGAATTCTCACTATGATACATGCACTCCGACCCCAACATCAACCAATTCTTTAAATATCTACTTGTATTCTTAGTAGCCATAATTATTTTAGTCTCAGCTAATAACATCTTCCAACTATTCATTGGCTGGGAGGGAGTTGGAATTATATCTTTCCTGCTCATCGGATGATGGTATGGCCGGGCAGATGCAAACACAGCTGCCCTCCAAGCCGTTATTTATAACCGAGTAGGAGACATCGGATTAATTATAACTATAGCCTGGTTCGCAATAAACCTCAACTCCTGAGAGATCCAACAAATCCTAACCCTATCAAAAGACTTTGACATAACACTTCCCCTAATTGGACTCATCTTAGCAGCAACGGGAAAATCCGCCCAGTTTGGCCTTCACCCTTGACTCCCAGCCGCCATAGAAGGCCCTACCCCAGTATCTGCCCTACTCCACTCAAGCACCATGGTTGTTGCAGGAATTTTCCTACTAATCCGCTTCCACCCTCTTATAGAAAACAACCAACTAGCATTGACAACTTGTCTCTGTCTTGGAGCACTAACCTCACTATTCGCAGCCACCTGCGCCCTCACCCAAAATGACATCAAAAAAATCGTAGCCTTCTCAACATCAAGCCAACTAGGCCTAATAATAGTCGCAATCGGATTAAATCAACCACAGCTAGCATTCCTTCATATCTGCACCCACGCCTTTTTCAAAGCTATATTATTCCTGTGTTCGGGATCAATTATTCACAGCCTAAATGACGAGCAAGACATCCGAAAAATAGGAGGCCTACTCAATACCATACCCACCACCTCTACTTATTTCACAATTGGCAGCCTAGCCCTAACGGGGACCCCCTTCCTAGCAGGGTTCTTCTCAAAAGACGCAATCATTGAAACCCTAAACTCCTCATCCCTAAACGCCTGAGCCCTTGTCCTGACATTAATTGCCACATCTTTTACAGCTGTATATAGTTTCCGTCTAGTATATTTTGTAATCATAGGAGCTCCCCGATTCCTACCCCTATGCCCGATCAACGAAAACAACCCATTAGTAATTAACCCCCTCAAGCGACTTGCCTGAGGAAGCATCACCGCAGGACTAATTATCACCCAAAACTTTTTACCAATAAAAACACCCGTTATAACAATACCAACCCCCTTAAAAATAGCGGCTCTCCTAGTAACAATTACAGGCCTGCTGACAGCCATAGAACTAGCCAACATAACAAGCAAACAAGTGAAAGTTACCCCAACAACTCACACACACCACTTCTCAAATATACTAGGATTCTTCCCTATAATTATTCATCGACTAATCCCAAAGCTTAAACTCACCCTAGGACAATCAGCCGCCACCCAACTCGACAAAACATGGCTCGAAACAATTGGACCAAAAGGCCTAGCGCTAACGCAAAAAAGCATAGCAAAAGCCACAAATAACATCTCACGGGGAATAATTAAGACGTACCTAACCATCTTCCTTTTAACCCTAACCCTAGCCGCTATCCTAATTTCACTTTAA

>A._kreyenbergii_1

ATGACACTAATCATACACTCGTCCCTTCTCTTAACCCTCCTTATTTTACTCCACCCACTACTCACCACACTCAACCCAAACCAACAAGACCCTAATATAGCAAAAACTACTAAAACTGCCATTAGCTCAGCCTTCTTCATTAGCCTCCTACCTCTTACAATTTTCCTGGCCCATGGAACAGAAAGCATCGTAACAAACTGGCAATGAATAAACACCCAAACATTCGACGTAAACATCAGCTTTAAATTTGATCACTACTCCCTAATCTTTATCCCAATTGCCTTATACGTCACCTGATCTATTCTTGAATTTTCACTATGATATATGCACTCCGACCCCAACATCAACCAATTCTTTAAGTATCTACTCGTGTTCTTAGTAGCCATAATTATTTTAGTCTCAGCTAACAACATCTTCCAACTATTTATTGGTTGAGAAGGGGTAGGAATCATATCCTTCCTTCTCATTGGGTGGTGATATGGCCGAGCGGATGCTAACACAGCTGCCCTCCAAGCCGTTATTTATAACCGAGTAGGGGACATCGGACTAATTATAACTATAGCCTGATTCGCAATAAACCTAAACTCCTGAGAAATCCAACAAATCTTAACCTTATCAAAAGACTTTAATATGACACTTCCCCTAGTTGGGCTCATCTTAGCAGCAACAGGGAAATCCGCCCAATTTGGCCTTCACCCCTGACTTCCAGCCGCCATAGAAGGCCCTACCCCAGTGTCTGCCCTACTCCACTCAAGTACTATGGTCGTTGCAGGAATCTTCCTACTAATCCGCTTTCACCCCCTCATAGAAAATAATCAACTGGCACTGACAACTTGTCTCTGCCTTGGAGCACTAACCTCACTATTCGCAGCCACCTGCGCCCTAACCCAAAATGATATCAAAAAAATTGTAGCTTTCTCAACATCAAGCCAACTGGGCCTAATGATAGTCGCAATCGGATTAAATCAACCACAACTAGCATTCCTTCATATCTGCACCCACGCCTTTTTCAAGGCTATACTATTCCTGTGCTCAGGATCAATTATTCACAGCCTAAACGACGAGCAAGACATCCGGAAAATGGGAGGCCTGCTCAATATTATACCCACCACCTCTACTTACTTTACAATTGGTAGCCTAGCCCTAACGGGAACCCCCTTCCTAGCAGGGTTCTTCTCAAAAGACGCGATCATCGAAGCCCTAAACTCTTCATCCCTAAACGCCTGAGCCCTTATCCTGACATTAATTGCCACATCTTTTACAGCTGTATATAGTTTCCGCCTAGTATATTTTGTAATCATAGGAGCCCCCCGATTCCTACCCCTATGCCCGATTAACGAAAACAACCCATTAGTAATTAACCCCCTCAAACGACTTGCCTGAGGAAGCATTACCGCAGGACTAATTATTACCCAGAACTTTTTACCAATAAAAACACCAGTTATAACAATACCAACCCCCTTAAAAATAGCGGCCCTCCTAGTAACAATTATAGGCCTGCTAACAGCCATAGAACTAGCCAATATAACAAGTAAACAAGTGAAAATTACCCCAATAACCCACACACATCACTTCTCAAATATACTAGGATTCTTCCCTATAATTACACATCGACTAGTCCCAAAACTTAAACTCACCCTAGGACAATCAGCCGCCACCCAACTCGACAAAACATGGCTCGAAACAATTGGGCCAAAAGGCCTAGCACTTACACAAAAAAACATAGCAAAAGCCACAAATAACATCTCACGAGGAATAATTAAAACATACCTAACCATCTTCCTCTTAACTCTAATCTTGGCCACTATCCTAATTTCACTTTAG

>A._kreyenbergii_2

ATGACACTAATCATACACTCGTCCCTTCTCTTAACCCTCCTTATCTTACTCCACCCACTTCTCACCACACTCAACCCAAACCAACAAGAGTCTAACATAGCAAAAACCACTAAAACTGCCATTAGCTTGGCCTTCTTCATTAGCCTCCTACCCCTTACAATTTTTCTGGCCCAAGGAACAGAAAGCATCGTAACAAACTGACAGTGAATAAACACCCAAACGTTCGACGTAAACATCAGCTTTAAATTTGATCATTACTCCCTAATCTTTATCCCAATTGCCTTATACGTCACCTGATCAATTCTTGAATTTTCACTATGATATATGCACTCCGACCCCAACATCAACCAATTCTTTAAATACCTACTCGTATTCTTAGTAGCCATAATTATCTTAGTCTCAGCTAACAACATCTTCCAACTATTTATTGGCTGAGAGGGAGTAGGAATCATATCCTTCCTTCTCATCGGATGGTGATATGGCCGGGCGGATGCTAACACAGCTGCCCTCCAAGCCGTTATTTATAACCGAGTAGGAGACATCGGACTAATTATAACTATAGCCTGGTTCGCAATAAACCTCAACTCCTGAGAAATCCAACAAATCTTAACCCTATCAAAAGACTTTAATATAACACTTCCCCTAGTTGGGCTCATCTTAGCAGCAACAGGAAAATCCGCCCAGTTTGGCCTTCACCCCTGACTCCCAGCCGCCATAGAAGGCCCTACCCCAGTGTCTGCCCTACTCCACTCAAGTACCATGGTCGTTGCAGGAATCTTCCTACTAATCCGCTTTCACCCCCTCATAGAAAACAATCAACTGGCACTGACAACTTGTCTCTGCCTCGGAGCACTAACCTCATTATTCGCAGCCACCTGCGCCCTAACCCAAAATGATATCAAGAAAATTGTAGCTTTCTCAACATCAAGCCAGCTGGGCCTAATGATAGTCGCAATCGGGTTAAATCAACCACAACTAGCATTCCTTCATATCTGCACCCACGCCTTTTTCAAGGCTATACTATTCCTGTGTTCAGGGTCAATTATTCACAGCCTAAATGACGAACAGGACATCCGAAAAATAGGAGGCCTGCTCAACATTATACCCACCACCTCTACTTACTTTACAATTGGTAGCCTAGCCCTGACGGGAACCCCCTTCCTAGCAGGGTTCTTCTCAAAAGACGCAATCATCGAAGCCCTAAACTCTTCATCCCTAAACGCCTGAGCCCTCATCCTGACATTAATTGCCACATCTTTTACAGCTGTATATAGTTTCCGCCTAGTATATTTTGTAATCATAGGAGCCCCCCGATTCCTACCCCTATGCCCGATTAACGAAAACAACCCATTAGTAATTAACCCCCTCAAACGACTTGCCTGAGGGAGCATCACCGCAGGACTAATTATTACCCAAAACTTTTTACCAATAAAAACACCAGTTATAACAATACCAACCCCCTTAAAAATAGCGGCCCTCCTAGTAACAATTGCAGGCCTGCTAACGGCCATAGAACTGGCTAACATAACAAGTAAACAAGTGAAAATTACCCCAATAACCCACACACACCACTTCTCAAACATACTAGGATTCTTCCCTATAGTCACACATCGACTAATCCCCAAACTTAAGCTCACCCTAGGACAATCAGCCGCCACCCAACTCGACAAAACATGGCTCGAAACAATTGGGCCAAAAGGCCTAGCACTTACACAAAAAAACATAGCAAAAGCCACAAATAACATCTCACGAGGAATAATTAAAACATACCTAACCACCTTCCTCTTAACTCTAATCTTGGCCACTATCCTAGTCTCACTTTAA

>A._longipinnis

ATGACACTAATCATACACTCGTCCCTCCTCCTAACCTTTCTCATTCTACTACACCCACTACTTACCACACTTAAACCCAACCAACAAGAATCCAATATAGCAGAAACTACTAAGACCGCCATTAGCTCCGCATTCTTCATCAGCCTCCTACCACTTACAATTTTCCTAACCCAAGGAACAGAAAGCATCGTGACGAACTGACAATGAATAAATACCCAAACATTTGACGTAAACATCAGCTTTAAATTCGACCACTATTCCTTAATTTTTATCCCAATCGCCTTATACGTCACCTGATCAATCCTCGAGTTTTCACTATGATACATACACTCTGACCCCAACATTAACCGGTTCTTCAAATATTTACTAGTATTCCTAGTAGCTATAATTATCCTAGTCTCAGCTAATAACATCTTTCAACTATTCATTGGCTGAGAAGGAGTAGGAATTATATCATTCCTCCTTATCGGGTGGTGATATGGCCGGGCAGATGCTAACACAGCAGCCCTTCAGGCCGTTATTTACAATCGAGTAGGAGACATCGGACTAATTATAACCACAGCTTGGTTCGCAATAAACCTTAACTCCTGAGAAATTCAACAAATCTTAACACTATCAAAAGACTTTAACATAACATTTCCCCTAATTGGGCTCATCTTAGCAGCAACAGGAAAATCCGCCCAATTCGGCCTCCACCCTTGACTCCCAGCCGCCATAGAAGGCCCTACCCCAGTGTCTGCCCTACTCCACTCAAGCACTATAGTTGTTGCAGGAATCTTCCTATTAATCCGGTTTCACCCCCTCACAGAAAATAACCAGCTGGCATTAACCATCTGCCTCTGCCTTGGAGCACTAACCTCATTATTCGCAGCCACCTGCGCCCTCACCCAAAATGATATCAAAAAAATTGTGGCTTTCTCAACATCAAGCCAGCTAGGCCTAATAATAGTCGCAATTGGATTAAACCAACCACAACTAGCATTCCTCCATATCTGCACCCATGCCTTCTTCAAAGCCATGTTATTCCTGTGCTCAGGTTCAATTATTCATAGCCTAAACGATGAACAAGACATCCGAAAAATAGGAGGCCTATTTAACACCATACCCACCACCTCTGCCTACTTCACAATCGGCAGCCTAGCCTTAACAGGAACCCCCTTCCTGGCAGGATTCTTCTCAAAAGACGCAATCATTGAAGCCCTAGGTACCTCCTATCTTAACGCCTGAGCCCTAACCCTAACACTAATTGCTACATCATTCACTGCAGTTTATAGCTTCCGTCTAGTATACTTCGTAATCATGGGAGCCCCACGATTTCTACCCCTATGCCCAATTAACGAAAACAATCCACTAGTAATTAACCCCCTCAAGCGACTTGCCTGAGGAAGCATCACCGCAGGACTTATCATCACCCAAAACCTCACACCAATAAAAACACCAATCATAACAATACCCGCCCCCCTAAAAATAGCAGCCCTCCTAGTAACAATTATAGGCCTATTAACAGCCATAGAACTGGCTAACATAACAAGTAAACAAGTAAAAATTACCCCAACAATCCATACACACCACTTCTCAAATATACTAGGATTCTTCCCCATAACTGTACATCGAATTGTTCCAAAACTTAAACTTATCCTAGGACAATCAGCCGCTACCCAACTCGATAAAACATGACTCGAAGCAATTGGGCCAAAAGGCCTAGCACTAACACAAAAGAACATGGCAAAAACTACAAATAATATCTCACGAGGAATGATTAAAACATACTTAACCATCTTCCTCCTAACCTTAATCTTAGCCACCACCCTAGTACTTCTCTAA

>A._monticola_1

ATGACATTAATCATACACTCATCCCTCCTCTTAACCTTTCTCATCCTACTTCACCCACTGCTCACCACACTTAACCCCCACCAACAAGAACTTCATATAGCAAAAACCACCAAAACCGCCATTAGCTCCGCATTCTTCATCAGCCTCCTACCACTCACAATCTTTCTAACTCAGGGAACAGAAAGCATTGTAACAAACTGACAATGAATAAACACCCAAACATTCGACGTAAATATTAGCTTTAAATTTGACCACTACTCCCTAATTTTTATCCCAATTGCCTTATACGTCACCTGATCAATCCTTGAATTTTCACTGTGGTATATACACTCCGACCCCAACATCGACCGATTCTTTAAATACTTACTCATCTTCTTAGTAGCCATAATTATTCTAGTCTCAGCTAACAACATCTTTCAACTATTTATTGGCTGAGAAGGAGTAGGAATTATGTCATTTCTACTCATCGGGTGATGATACGGGCGGACAGACGCCAACACAGCAGCCCTACAAGCTGTCATCTATAACCGAGTAGGAGATATCGGACTAATTTTAACCACAGCCTGATTCGCAATAAACCTCAACTCCTGAGAAATTCAACAAATCCTAACACTATCAAAAGACTTTGACATAACCCTCCCTCTAATTGGACTTATCTTAGCCGCAACAGGAAAATCAGCCCAATTTGGCCTTCACCCCTGACTCCCAGCCGCCATAGAGGGCCCTACCCCAGTATCTGCCCTACTCCACTCCAGCACTATAGTTGTTGCAGGAATCTTCCTACTAATCCGCTTCCACCCCCTTATGGAAAATAACCAGCTAGTCCTAACAATCTGTCTTTGCCTTGGAGCACTAACCTCGCTATTCTCAGCCACCTGCGCCCTAACCCAAAATGATATTAAAAAAATCGTAGCTTTCTCAACAGCGAGTCAGCTAGGCCTAATAATAGTCGCAATTGGACTTAACCAACCACAACTAGCGTTCCTCCACATCTGCACCCATGCCTTTTTCAAGGCCATATTATTCCTGTGCTCAGGATCAATTATCCACAGCCTAAACGACGAACAAGATATCCGAAAAATAGGAGGCCTATTTAATATTATACCTGCTACCTCAACCTACTTTACAATCGGCAGCCTAGCCCTGACAGGAACCCCATTCCTAGCAGGGTTCTTCTCAAAAGACGCAATTATTGAAGCCCTAAACACCTCTTATCTTAACGCCTGAGCCCTAACCCTCACACTAATCGCTACTTCATTTACTGCAGTATATAGCTTCCGTCTAGTATACTTCGTAATCATAGGAGCCCCACGATTCTTACCCCTATGTCCAATTAACGAAAACAATCCACTAGTAATCAATCCCCTTAAACGACTTGCCTGAGGAAGTATCACTGCAGGACTTATTATTACTCAAAACCTTCTACCAATAAAAACACCCATCATAACAATACCAACCGCCCTAAAAACAGCAGCCCTCATAGTAACAATTATAGGCCTACTAACAGCCATAGAATTAGCCAACATGACAAGTAAACAAGTAAAAATTACCCCAACAATCCCCACACACCATTTCTCAAACATATTAGGATTCTTCCCAATAGTTGTACACCGACTCATTCCAAAACTTAAACTTACTCTAGGACAATCAGCCGCCACCCAACTAGACAAAACATGGCTCGAAACAATTGGACCAAAAGGCCTAGCACAAACACAAAAAACTATGGCAAAAGCCACAAATAACATTTCACGAGGAATAATCAAGACATATCTAACCATCTTCCTTCTGACTCTAACCATAGCCTCCATCATAATTCTCCTTTAA

>A._monticola_2

ATGACATTAATCATACACTCATCCCTCCTCTTAACCTTTCTCATCCTACTTCACCCACTGCTCACCACACTTAACCCCCACCAACAAGAACTTCATATAGCAAAAACCACCAAAACCGCCATTAGCTCCGCATTCTTCATCAGCCTCCTACCACTCACAATCTTTCTAACTCAGGGAACAGAAAGCATTGTAACAAACTGACAATGAATAAACACCCAAACATTCGACGTAAATATTAGCTTTAAATTTGACCACTACTCCCTAATTTTTATCCCAATTGCCTTATACGTCACCTGATCAATCCTTGAATTTTCACTGTGGTATATACACTCCGACCCCAACATCGACCGATTCTTTAAATACTTACTCATCTTCTTAGTAGCCATAATTATTCTAGTCTCAGCTAACAACATCTTTCAACTATTTATTGGCTGAGAAGGAGTAGGAATTATGTCATTTCTACTCATCGGGTGATGATACGGGCGGACAGACGCCAACACAGCAGCCCTACAAGCTGTCATCTATAACCGAGTAGGAGATATCGGACTAATTTTAACCACAGCCTGATTCGCAATAAACCTCAACTCCTGAGAAATTCAACAAATCCTAACACTATCAAAAGACTTTGACATAACCCTCCCTCTAATTGGACTTATCTTAGCCGCAACAGGAAAATCAGCCCAATTTGGCCTTCACCCCTGACTCCCAGCCGCCATAGAGGGCCCTACCCCAGTATCTGCCCTACTCCACTCCAGCACTATAGTTGTTGCAGGAATCTTCCTACTAATCCGCTTCCACCCCCTTATGGAAAATAACCAGCTAGTCCTAACAATCTGTCTTTGCCTTGGAGCACTAACCTCGCTATTCTCAGCCACCTGCGCCCTAACCCAAAATGATATTAAAAAAATCGTAGCTTTCTCAACAGCGAGTCAGCTAGGCCTAATAATAGTCGCAATTGGACTTAACCAACCACAACTAGCGTTCCTCCACATCTGCACCCATGCCTTTTTCAAGGCCATATTATTCCTGTGCTCAGGATCAATTATCCACAGCCTAAACGACGAACAAGATATCCGAAAAATAGGAGGCCTATTTAATATTATACCTGCTACCTCAACCTACTTTACAATCGGCAGCCTAGCCCTGACAGGAACCCCATTCCTAGCAGGGTTCTTCTCAAAAGACGCAATTATTGAAGCCCTAAACACCTCTTATCTTAACGCCTGAGCCCTAACCCTCACACTAATCGCTACTTCATTTACTGCAGTATATAGCTTCCGTCTAGTATACTTCGTAATCATAGGAGCCCCACGATTCTTACCCCTATGTCCAATTAACGAAAACAATCCACTAGTAATCAATCCCCTTAAACGACTTGCCTGAGGAAGTATCACTGCAGGACTTATTATTACTCAAAACCTTCTACCAATAAAAACACCCATCATAACAATACCAACCGCCCTAAAAACAGCAGCCCTCATAGTAACAATTATAGGCCTACTAACAGCCATAGAATTAGCCAACATGACAAGTAAACAAGTAAAAATTACCCCAACAATCCCCACACACCATTTCTCAAACATATTAGGATTCTTCCCAATAGTTGTACACCGACTCATTCCAAAACTTAAACTTACTCTAGGACAATCAGCCGCCACCCAACTAGACAAAACATGGCTCGAAACAATTGGACCAAAAGGCCTAGCACAAACACAAAAAACTATGGCAAAAGCCACAAATAACATTTCACGAGGAATAATCAAGACATATCTAACCATCTTCCTTCTGACTCTAACCATAGCCTCCATCATAATTCTCCTTTAA

>A._paradoxus_1

ATGACATTAATCATACACTCATCCCTCCTCCTCACTCTTCTTATCCTACTCCACCCACTACTTACCACACTTAACCCAGACCAACAAGAGTCTAACATGGCAGAAACCACCAAAACTGCCATCAGCACCGCCTTCTTCATTAGCCTCTTTCCCCTTATAATTTTCCTAGCCCAAGGGACAGAAAGCATTGTAACAAGCTGACAATGAATAAACACCCAAACATTCGATGTAAACATCAGCTTTAAGTTTGACCACTACTCCTTAATCTTTATCCCAATTGCCCTATACGTCACCTGATCAATCCTTGAATTCTCACTATGATACATACACTCTGACCCCAACATTAACCGATTCTTTAAGTATCTCCTCGTATTCTTAGTAGCCATAATCATCTTAGTCTCAGCTAATAACATTTTCCAACTATTTATTGGTTGGGAAGGGGTAGGGATTATATCATTCCTCCTCATTGGATGATGATACGGTCGGGCGGATGCTAACACAGCTGCCCTCCAAGCCGTCATTTACAACCGGGTAGGGGACATCGGGCTAATTATAACTATAGCCTGGTTCGCAATAAACCTCAACTCCTGAGAAATTCAACAAATCCTGACACTATCAAAAGACTTTAACATGACGCTTCCCCTAATTGGGCTCATCTTAGCAGCAACAGGAAAATCCGCCCAATTCGGCCTGCACCCCTGACTCCCAGCCGCCATAGAAGGCCCTACCCCAGTATCTGCCCTACTCCACTCCAGCACCATAGTCGTTGCAGGAGTCTTCCTACTAATCCGCTTTCACCCCCTCATAGAAAATAATCAACTAGCACTAACAACTTGCCTCTGTCTTGGAGCATTAACCTCACTATTCGCAGCCACCTGCGCCCTCACCCAAAATGATATCAAAAAAATCGTAGCTTTCTCAACATCAAGCCAACTAGGCCTAATAATGGTCGCAATCGGACTAAATCAACCACAACTAGCATTCCTTCACATCTGCACCCACGCCTTCTTTAAAGCAATACTATTCCTGTGTTCGGGATCAATTATCCACAGCCTAAATGACGAGCAAGACATCCGAAAAATAGGGGGCCTACTCAACACCATACCTGCCACCTCTACCTACTTCACAATTGGCAGCCTAGCCCTGACAGGAACCCCATTCCTAGCAGGATTCTTCTCAAAAGACGCAATCATCGAAGCCCTAAATACCTCATCCCTAAACGCCTGAGCCCTTGTCCTAACACTAATTGCTACCGCATTCACAGCAGTATATAGCTTCCGCCTACTATACCTCGTAATCATAGGAGCCCCCCGATCCCTGCCCCTATGCCCTATTAATGAAAACAACCCATTAGTAATCAACCCCCTCAAACGACTTGCCTGAGGAAGTATCACCGCAGGGTTTATTATTACTCAAAATCTTCTACCAATAAAAACACCAGTTATAACTATACCAACCTCCCTAAAAATAGCAGCTATCCTAGTGACAATCGTAGGTCTACTGACAGCCATAGAACTGGCTAACATAACAAGCAAACAAGTAAAAATTACCCCAACAATTCACACACACCACTTCTCAAATATACTAGGATTCTTCCCCACAATTGTTCACCGACTTATCCCAAAGCTTAAACTTACCCTAGGACAGTCAGCCGCCACCCAACTCGACAAAACATGACTAGAAGCAATCGGGCCGAAAGGCCTGGCGTTAACACAAAAAAATATAGCAAAGGCTACAAATAACATCTCACGAGGAATAATTAAAACATATCTAACCATTTTCCTCTTAACCCTAACCTTAGCCGCTATCTTAATCTCGCTCTAA

>A._paradoxus_2

ATGACACTAATCATACACTCGTCCCTCCTCCTAACCCTTATTATCCTACTCCACCCACTACTCACCACACTTAACCCAAACCAACAAGGATCTAATATGGCAAAAACCACTAAAACTGCCATTAGTTTAGCCTTCTTCATCAGCCTCCTACCTCTTACAATTTTCCTAGCCCAGGGGGCAGAAAGCATCGTAACAAACTGACAATGAATAAATACCCAAACATTTGATGTAAACATCAGCCTTAAATTTGATCACTACTCCTTAATCTTTATCCCAATTGCCTTATATGTCACCTGATCAATCCTTGAATTCTCACTATGATACATGCACTCCGACCCCAACATCAACCAATTCTTTAAATATCTACTTGTATTCTTAGTAGCCATAATTATTTTAGTCTCAGCTAATAACATCTTCCAATTATTCATTGGCTGGGAAGGAGTTGGAATTATATCTTTCCTGCTCATCGGATGATGATATGGCCGGGCAGATGCAAACACAGCTGCCCTCCAAGCCGTTATTTATAACCGAGTAGGAGACATCGGATTAATTATAACTATAGCCTGGTTCGCAATAAACCTCAACTCCTGAGAGATCCAACAAATCCTAACCCTATCAAAAGACTTTGACATAACACTTCCCCTAATTGGACTCATCTTAGCAGCAACGGGAAAATCCGCCCAGTTTGGCCTTCACCCTTGACTCCCAGCCGCCATAGAAGGCCCTACCCCAGTATCTGCCCTACTCCACTCAAGCACCATGGTTGTTGCAGGAATTTTCCTACTAATCCGCTTCCACCCTCTTATAGAAGACAACCAACTAGCATTGACAACTTGTCTCTGTCTTGGAGCACTAACCTCACTATTCGCAGCCACCTGCGCCCTCACCCAAAATGACATCAAAAAAATCGTAGCCTTCTCAACATCAAGCCAACTAGGCCTAATAATAGTCGCAATCGGATTAAATCAACCACAGCTAGCATTCCTTCATATCTGCACCCACGCCTTTTTCAAAGCTATATTATTCCTGTGTTCGGGATCAATTATTCACAGCCTAAATGACGAGCAAGACATCCGAAAAATAGGAGGCCTACTCAATACCATACCCACCACCTCTACTTATTTCACAATTGGCAGCCTAGCCCTAACGGGGACCCCCTTCCTAGCAGGGTTCTTCTCAAAAGACGCAATCATTGAAACCCTAAACTCCTCATCCCTAAACGCCTGAGCCCTTGTCCTGACATTAATTGCCACATCTTTTACAGCTGTATATAGTTTCCGTCTAGTATATTTTGTAATCATAGGAGCTCCCCGATTCCTACCCCTATGCCCGATCAACGAAAACAACCCATTAGTAATTAACCCCCTCAAGCGACTTGCCTGAGGAAGCATCACCGCAGGACTAATTATCACCCAAAACTTTTTACCAATAAAAACACCCGTTATAACAATACCAACCCCCTTAAAAATAGCGGCTCTCCTAGTAACAATTACAGGCCTGCTGACAGCCATAGAACTAGCCAACATAACAAGCAAACAAGTGAAAGTTACCCCAACAACTCACACACACCACTTCTCAAATATACTAGGATTCTTCCCTATAATTATTCATCGACTAATCCCAAAGCTTAAACTCACCCTAGGACAATCAGCCGCCACCCAACTCGACAAAACATGGCTCGAAACAATTGGACCAAAAGGCCTAGCGCTAACGCAAAAAAGCATAGCAAAAGCCACGAATAACATCTCACGAGGAATAATTAAGACGTACCTAACCATCTTCCTTTTAACCCTAACCCTAGCCGCTATCCTAATTTCACTTTAA

>A._parallens_1

ATGACACTAATCATACACTCGTCCCTTCTCCTAACTCTTCTTATTCTACTCCACCCACTACTCACCACACTTAACCCAGACCAACAAGGATCAAATATGGCAAACATCACTAAAACTGCCATTAGTTTAGCCTTCTTCACCAGCCTCTTACCTCTTACAATTTTCCTAACCCAGGGGGCAGAAAGCATCGTAACAAACTGACAATGAATAAACACCCAAACATTTGATGTAAACATCAGCTTTAAATTTGACCACTACTCCTTAATCTTTATCCCTATTGCCTTATACGTCACCTGATCAATCCTTGAATTCTCGCTATGGTACATGCACTCCGACCCCAACATCAACCAATTCTTCAAGTACCTACTTGTATTCTTAGTAGCCATAATTATCCTAGTTTCAGCTAACAACATCTTCCAACTATTTATCGGCTGAGAAGGGGTTGGAATTATATCTTTCCTACTTATCGGATGATGATATGGCCGGGCGGATGCTAACACAGCCGCCCTCCAAGCCGTTATTTACAACCGAGTAGGAGACATCGGACTAATTATAACTATAGCCTGGTTCGCAATAAACCTCAACTCCTGAGAAATCCAACAAATCCTAACTCTATCAAAAGATTTTAACATAACACTTCCCCTAATTGGACTCATCTTAGCAGCAACGGGAAAATCCGCCCAATTTGGCCTTCACCCCTGACTTCCAGCCGCTATAGAAGGCCCTACCCCAGTATCTGCCCTACTTCACTCAAGCACCATGGTCGTTGCGGGAATCTTTCTACTAATCCGCTTTCACCCCCTTATAGAAAATAATCAATTACTGCTGACAACCTGTCTCTGTCTCGGAGCACTGACCTCACTATTCGCAGCCACCTGCGCCCTTACCCAAAATGACATCAAAAAAATTGTAGCTTTCTCAACATCAAGCCAGCTGGGCCTAATAATAGTCGCAATCGGACTAAATCAACCACAACTAGCATTCCTCCATATCTGCACCCACGCCTTTTTCAAAGCTATATTATTTCTGTGCTCAGGATCAATTATTCACAGCCTAAATGACGAACAAGACATCCGAAAAATAGGAGGCCTACTCAACATTATACCTGCCACCTCTACTTATTTCACAATCGGCAGCCTAGCCCTAACAGGGACCCCCTTCCTAGCAGGGTTCTTCTCTAAAGACGCGATTATTGAAACCCTAAACTCCTCATCCCTAAACGCCTGAGCCCTTATCCTGACATTAATTGCCACATCTTTCACAGCTGTATATAGTTTCCGTCTAGTATATTTTGTAATCATGGGGGCCCCCCGATTCCTGCCCCTATGCCCGATCAACGAAAACAACCCATTAGTGATTAACCCCCTCAAGCGACTTGCCTGAGGAAGCATCACCGCAGGACTAATTATCACCCAAAACTTTTTACCAATAAAAACACCCGTTATAACGATACCAACCCCCTTAAAAATAGCGGCTCTCCTAGTAACAGTTGTAGGCCTGCTAACAGCCATAGAACTGGCCAACATAACAAGTAAACAAGTGAAAATCACCCCAACAACCCACACACACCACTTCTCCAATATACTAGGATTTTTCCCCATAGTTGTGCATCGACTAGTCCCAAAACTCAAGCTCACCCTAGGGCAGTCAGCCGCCACCCAACTCGATAAAACATGACTTGAGACGATTGGACCAAAAGGCCTAGCACTAACACAAAAAGGTATAGCAAAAGCTACAAACAATATCTCACGAGGAATAATTAAAACATATCTAACCATCTTCCTCCTAACCCTAATCTTAGCCACTATCCTAGTCTCACTTTAA

>A._parallens_2

ATGACACTAATCATACACTCGTCCCTTCTCCTAACCCTTCTTATTCTACTCCACCCACTACTCACCACACTTAACCCAAACCAACAAGGATCAAATATGGCAAAGATCACTAAAACTGCCATTAGTTTAGCCTTCTTCACCAGCCTCTTACCTCTTACAATTTTCCTAGCCCAAGGGGCAGAGAGCATCGTAACAAACTGACAATGAATAAACACCCAAACATTTGATGTAAACATCAGCTTTAAATTTGACCACTACTCCTTAATCTTTATCCCTATTGCCCTATACGTCACCTGATCAATCCTTGAATTCTCGCTATGGTACATGCACTCCGACCCCAACATCAACCAATTCTTTAAATACCTACTTGTATTCTTAGTAGCCATAATTATCCTAGTCTCAGCTAACAACATCTTCCAACTATTTATCGGCTGAGAAGGGGTCGGAATTATATCTTTCCTACTTATCGGATGATGATACGGCCGGGCGGATGCTAACACAGCCGCCCTCCAAGCCGTTATTTACAACCGAGTAGGAGACATCGGACTAATTATAACTATAGCCTGGTTCGCAATAAACCTCAACTCCTGAGAAATCCAACAAATCCTAACTCTATCAAAAGATCTTAACATAACACTTCCCCTAATTGGACTCATCTTAGCAGCAACAGGAAAATCCGCCCAATTTGGCCTTCACCCCTGACTTCCAGCCGCCATAGAAGGCCCTACCCCAGTATCTGCCCTACTTCACTCAAGCACCATGGTCGTTGCGGGAATCTTTCTACTAATCCGCTTTCACCCCCTTATAGAAAATAATCAACTACTGCTGACAACCTGTCTCTGTCTCGGAGCACTAACCTCACTATTCGCAGCCACCTGCGCCCTTACCCAAAATGACATCAAAAAAATCGTAGCTTTCTCAACATCAAGCCAGCTGGGCCTAATAATAGTCGCAATCGGACTAAATCAACCACAACTAGCATTCCTCCATATCTGTACCCACGCCTTCTTTAAAGCTATGTTATTTCTGTGCTCAGGGTCAATTATTCACAGCCTAAATGACGAACAAGACATCCGAAAAATAGGGGGCCTACTCAACATTATACCTACCACCTCCACTTATTTCACAATCGGCAGCCTAGCCCTAACAGGGACCCCCTTCCTAGCAGGGTTCTTCTCAAAGGACGCAATCATTGAAACCCTAAACTCCTCATCCCTAAACGCCTGAGCCCTTATCCTGACATTAATTGCCACATCTTTCACAGCTGTATATAGTTTCCGTCTAGTATATTTTGTAATCATAGGAGCCCCCCGATTCCTACCCCTATGCCCGATCAACGAAAACAACCCATTAGTGATTAACCCCCTCAAACGACTTGCCTGAGGAAGCATCACCGCAGGACTAATTATTACCCAAAACTTTCTACCAATAAAAACACCCGTTATAACAATACCGACCCCCTTAAAAATAGCGGCCCTCCTAGTAACAATTGTAGGCCTGCTGACAGCCATAGAACTGGCCAACATAACAAGCAAACAAGTGAAAATCACCCCAACAACCCACACACACCACTTCTCCAATATACTAGGATTTTTCCCCATAGTTGTGCATCGACTAGTCCCAAAACTCAAACTCACCCTAGGGCAGTCAGCCGCCACCCAACTCGACAAGACATGACTCGAAACAATTGGGCCAAAAGGCCTAGCACTAACACAAAAAAGTATAGCAAAAACTACAAATAATATCTCACGAGGAATAATTAAAACATATCTAACCATCTTCCTCCTAACCCTAATCTTAGCCACTATCCTAGTCTCACTTTAA

>A._parallens_3

ATGACACTAATCATACACTCGTCCCTTCTCCTAACCCTTCTTATTCTACTCCACCCACTACTCACCACACTTAACCCAGACCAACAAGGATCAAATATGGCAAACATCACTAAAACTGCCATTAGTCTAGCCTTCTTCACCAGCCTCTTACCTCTTACAATTTTCCTAACCCAAGGGGCAGAGAGCATCGTAACAAACTGACAATGAATAAACACCCAAACATTTGATGTAAACATCAGCTTTAAATTTGACCACTACTCCTTAATCTTTATCCCTATTGCCTTATACGTCACCTGATCAATCCTTGAATTCTCGCTATGGTACATGCACTCCGACCCCAACATCAACCAATTCTTTAAATACCTACTTGTATTCTTAGTAGCCATAATTATTCTAGTTTCAGCTAACAACATCTTCCAACTATTTATCGGCTGAGAAGGGGTTGGAATTATATCTTTCCTACTTATCGGATGATGATATGGCCGGGCGGATGCTAACACAGCCGCCCTCCAAGCCGTTATTTACAACCGAGTAGGAGACATCGGACTAATTATAACTATAGCCTGGTTCGCAATAAACCTCAACTCCTGAGAAATCCAACAAATCCTAACTCTATCAAAAGATTTTAACATAACACTTCCCCTAATTGGACTCATCTTAGCGGCGACGGGAAAATCCGCCCAGTTTGGCCTTCACCCCTGACTCCCAGCCGCTATAGAAGGCCCTACCCCAGTATCTGCCCTACTTCACTCAAGCACCATGGTCGTTGCGGGAATCTTTCTACTAATCCGCTTTCACCCCCTTATAGAAAATAATCAATTACTGCTAACAACCTGTCTCTGCCTCGGAGCACTAACCTCACTATTCGCAGCCACCTGCGCCCTCACCCAAAATGACATCAAAAAAATTGTAGCTTTCTCAACATCAAGCCAGCTGGGCCTAATAATAGTCGCAATCGGACTAAATCAACCACAGCTAGCATTCCTCCATATCTGCACCCACGCCTTTTTCAAAGCTATATTATTTCTGTGCTCAGGATCAATTATTCACAGCCTAAATGATGAACAAGACATCCGAAAAATAGGAGGCCTACTCAACATTATACCTGCCACCTCTACTTATTTCACAATCGGCAGCCTAGCCCTAACGGGGACCCCCTTCCTAGCAGGGTTCTTCTCTAAAGACGCGATCATTGAAACCCTAAACTCCTCATCCCTAAACGCCTGAGCCCTTATCCTGACATTAATTGCCACATCTTTCACAGCTGTATATAGTTTCCGTCTAGTATATTTTGTAATCATGGGAGCCCCCCGATTCCTACCCCTATGCCCAATCAACGAAAACAACCCATTACTGATTAACCCCCTCAAGCGACTTGCCTGAGGAAGCATCACCGCAGGACTAATTATCACCCAAAACTTTTTACCAATAAAAACACCCGTTATAACAATACCAACCCCCTTAAAAATAGCGGCTCTCCTAGTAACAGTTGTAGGCCTGCTGACAGCCATAGAACTGGCCAACATAACAAGTAAACAAATGAAAATCACCCCAACAACCCACACACACCACTTCTCTAATATACTAGGATTTTTCCCCATAGTTGTGCATCGACTAGTCCCAAAACTCAAACTCACCCTAGGGCAGTCAGCCGCCACCCAACTCGACAAAACATGACTCGAGACCATTGGACCAAAAGGCCTAGCACTAACACAAAAAGGTATAGCAAAAGCTACAAATAATATCTCACGAGGAATAATTAAAACATATCTAACCATCTTCCTCCTAACCCTAACCTTAGCCACTATCCTAGTCTCACTTTAA

>A._parallens_4

ATGACACTAATCATACACTCGTCCCTTCTCCTAACCCTTCTTATTCTACTCCACCCACTACTCACCACACTTAACCCAAACCAACAAGGATCAAATATGGCAAACATCACTAAAACTGCCATTAGTCTAGCCTTCTTCACCAGCCTCTTACCTCTTACAATTTTCCTGACCCAAGGGGCAGAAAGCATCGTAACAAACTGACAATGAATAAACACCCAAACATTTGATGTAAACATCAGCTTTAAATTTGACCACTACTCCTTAATCTTTATCCCTATTGCCTTATACGTCACCTGATCAATCCTTGAATTCTCGCTATGGTACATGCACTCCGACCCCAACATCAACCAATTCTTTAAGTACCTACTTGTATTCTTAGTAGCCATAATTATCCTAGTTTCAGCTAACAACATCTTCCAACTATTTATCGGCTGAGAAGGGGTTGGAATTATATCTTTCCTACTTATCGGATGATGATATGGCCGGGCGGATGCTAACACAGCCGCCCTCCAAGCCGTTATTTACAACCGAGTAGGGGACATCGGACTAATTATAACTATAGCCTGGTTCGCAATAAACCTCAACTCCTGAGAAATCCAACAAATCCTAACTCTATCAAAAGATTTTAACATAACACTTCCCCTAATTGGACTCATCTTAGCAGCAACAGGAAAATCCGCCCAGTTTGGCCTTCACCCCTGACTTCCAGCCGCTATAGAAGGCCCTACCCCAGTATCTGCCCTACTTCACTCAAGCACCATGGTCGTTGCGGGAATTTTTCTACTAATCCGCTTTCACCCCCTTATAGAAAATAATCAATTACTGCTGACAACCTGTCTCTGTCTCGGAGCACTAACCTCACTATTCGCAGCCACCTGCGCCCTTACCCAAAATGATATCAAAAAAATTGTAGCTTTCTCAACATCAAGCCAGCTGGGCCTAATAATGGTCGCAATCGGACTAAATCAACCACAACTAGCATTCCTCCATATCTGCACCCACGCCTTTTTCAAAGCTATATTATTTCTGTGCTCAGGATCAATTATTCACAGCCTAAATGACGAACAAGACATCCGAAAAATAGGAGGCCTACTCAACATTATACCTGCCACCTCTACTTATTTCACAATCGGCAGCCTAGCCCTAACGGGGACCCCCTTCCTAGCAGGGTTCTTCTCTAAAGACGCGATCATTGAAACCCTAAACTCCTCATCCCTAAACGCCTGAGCCCTTATCCTGACATTAATTGCCACATCTTTCACAGCTGTATATAGTTTCCGTCTAGTATATTTTGTAATCATGGGAGCCCCCCGATTCCTACCCCTATGCCCGATCAACGAAAACAACCCATTAGTGATTAACCCCCTCAAGCGACTTGCCTGAGGAAGCATCACCGCAGGACTAATTATCACCCAAAACTTTTTACCAATAAAAACTCCCGTTATAACAATACCAACCCCCTTAAAAATAGCGGCTCTCCTAGTAACAGTTGTAGGCCTGCTGACAGCCATAGAACTGGCCAACATAACAAGTAAACAAGTGAAAATCACCCCAACAACCCACACACACCACTTCTCCAATATACTAGGATTTTTCCCCATAGTTGTGCATCGACTAGTCCCAAAACTCAAACTCACTCTAGGGCAGTCAGCCGCCACCCAACTCGACAAAACATGACTCGAGACCATTGGGCCAAAAGGCCTAGCACTGACACAAAAAGGTATAGCAAAAGCTACAAATAATATCTCACGAGGAATAATTAAAACATATCTAACCATCTTCCTCCTAACCCTAATCTTAGCCACTATCCTAGTCTCACTTTAA

>A._spinifer

ATGACATTAATCATACACTCATCCCTTCTCCTCACCCTTCTTATCCTACTCCACCCACTACTCACTACACTCAACCCAGACCAACAAGGGTCTAACATAGCAGAAACCACCAAAACTGCCATCAGCACCGCTTTCTTCACTAGCCTCCTACCTCTTGTAATCTTCCTGGCCCAAGGAACAGAAAGCATCGTAACAAACTGACAATGAATAAACACCCAAACATTTGACGTAAATATCAGCTTTAAATTCGACCACTACTCCTTAATCTTCATCCCAATTGCCCTATATGTCACTTGATCAATCCTTGAATTCTCACTATGATACATGCACTCCGACCCCAACATCAACCAATTCTTCAAATATCTCCTCGTATTCTTAGTAGCCATAATCATCCTAGTCTCAGCTAACAACGTTTTCCAACTATTCATTGGTTGAGAGGGGGTAGGAATTATATCATTCCTCCTTATCGGATGATGATACGGCCGGGCAGACGCTAATACAGCCGCCCTCCAAGCCGTTATTTACAACCGAGTGGGAGACATCGGACTAATTATAACTACAGCTTGGTTCGCAATAAACCTCAACTCCTGAGAAATTCAACAAATCCTGACATTATCAAAAGACTTTAACATAACACTTCCCCTAATTGGACTCATCTTAGCAGCAACAGGAAAATCCGCCCAATTCGGCCTTCACCCCTGACTCCCAGCCGCTATAGAAGGCCCTACCCCAGTATCTGCCCTACTCCACTCAAGCACCATGGTTGTTGCAGGAGTCTTCCTATTAATCCGCTTTCACCCCCTTATAGAAAATAATCAACTGGCACTAACAACTTGTCTCTGCCTTGGAGCACTAACCTCATTATTCGCAGCCACCTGCGCCCTCACCCAAAATGACATCAAGAAAATTGTAGCTTTCTCAACATCAAGCCAGCTAGGCCTAATAATGGTTGCAATCGGACTAAACCAGCCACAACTAGCATTCCTTCACATCTGCACCCACGCCTTCTTCAAAGCTATACTATTCCTGTGTTCAGGATCAATTATTCACAGCCTGAATGATGAACAAGACATCCGAAAGATGGGAGGCTTACTCAACATAATGCCCGCCACCTCTACTTACTTTACAATCGGCAGCCTAGCCTTAACAGGTACCCCATTCCTAGCGGGATTCTTCTCAAAAGACGCAATCATTGAAGCCCTAAATACCTCATCCCTAAACGCCTGAGCCCTTGTCCTAACGCTAATTGCTACCGCATTCACGGCAGTATATAGCTTCCGTCTACTTCACCTTGTAATCATAGGAGCCCCCCGATCCCTACCCCTATGCCCTATCAATGAAAACAACCCATTAGTAATTAACCCCCTCAAACGACTTGCCTGAGGAAGCATCACTGCGGGATTTATTATTACTCATAATCTTCTACCAATAAAAACACCAGTCATAACTATACCAACCTCATTAAAAATAGCAGCTATCCTAGTAACAATTGCAGGTCTGCTGACAGCCATAGAACTAGCCAACATGACAAGTAAACAAGTAAAAATTATCCCAACAACTCACACACACCACTTCTCAAACATGCTAGGATTCTTCCCCACAATTATACACCGACTTATTCCCAAACTCAAACTTACCCTAGGGCAGTCAGCCGCCACCCAACTCGACAAAACATGACTAGAAACGATCGGACCAAAAGGCCTGGCACTAGCACAAAAAAACATAGCAAAAGTTACAAATAACATCTCACGAGGACTAATTAAAACGTACCTAACTATCTTCCTCTTAACCTTAATCCTAGCCACTATCTTAATTTCACTTTAA

>A._stenotaeniatus

ATGACATTAATCATACACTCATCCCTTCTCCTCACCCTTCTTATCCTACTCCACCCACTACTCACTACACTCAACCCAGACCAACAAGGGTCTAACATAGCAGAAACCACCAAAACTGCCATCAGCACCGCTTTCTTCACTAGCCTCCTACCTCTTGTAATTTTCCTGGCCCAAGGAACAGAGAGCATCGTAACAAACTGACAATGAATAAACACCCAAACATTTGACGTAAATATCAGCTTTAAATTCGACCACTACTCCTTAATCTTCATCCCAATTGCCCTGTATGTCACTTGATCAATCCTTGAATTCTCACTATGATACATGCACTCCGACCCCAACATCAACCAATTCTTCAAATATCTCCTCGTATTCTTAGTAGCCATAATCATCCTAGTCTCAGCTAACAACATTTTCCAACTATTCATTGGTTGAGAGGGGGTAGGAATTATATCATTCCTCCTTATCGGATGATGATACGGCCGGGCAGACGCTAATACGGCCGCCCTCCAAGCCGTTATTTATAACCGAGTGGGAGACATCGGACTAATTATAACTACAGCTTGGTTCGCAATAAACCTCAACTCCTGAGAAATTCAACAAATCCTGACATTATCAAAAGACTTTAACATAACACTTCCCCTAATTGGACTCATCTTAGCAGCAACAGGAAAATCCGCCCAATTCGGCCTTCACCCCTGACTCCCAGCCGCTATAGAAGGCCCTACCCCAGTATCTGCCCTACTCCACTCAAGCACCATGGTTGTTGCAGGAATCTTCCTATTAATCCGCTTTCACCCCCTTATAGAAAATAATCAACCGGCACTAACAACTTGTCTCTGCCTTGGAGCACTAACCTCATTATTCGCAGCCACCTGCGCCCTCACCCAGAATGACATCAAGAAAATTGTAGCTTTCTCAACATCAAGCCAGCTAGGCCTAATAATGGTTGCAATCGGACTAAACCAACCACAACTAGCATTCCTTCACATCTGCACCCACGCCTTCTTCAAAGCTATACTATTCCTGTGTTCAGGATCAATTATTCACAGCCTGAATGATGAACAAGACATCCGAAAGATGGGAGGCCTACTCAACATAATGCCCGCCACCTCTACTTACTTTACAATCGGCAGCCTAGCCTTAGCAGGTACCCCATTCCTAGCGGGATTCTTCTCAAAAGACGCGATCATTGAAGCCCTAAATACCTCATCCCTAAACGCCTGAGCCCTTGTCCTAACGCTAATTGCTACCGCATTCACGGCAGTATATAGCTTCCGTCTACTACACCTTGTAATCATAGGAGCCCCCCGATCCCTGCCCCTATGCCCTATTAATGAAAACAACCCATTAGTAATTAACCCCCTCAAACGACTTGCCTGAGGAAGCATCACCGCAGGATTTATTATTACTCACAATCTTCTACCAATAAAAACACCAGTCATAACTATACCAACCTCCTTAAAAATAGCAGCTATCCTAGTAACAATTGCAGGTCTGCTGACAGCCATAGAACTAGCCAACATAACAAGTAAACAAGTAAAAATTATCCCAACAACTTACACACACCACTTCTCAAATATGCTAGGATTCTTCCCCACAATTATACACCGACTTATTCCCAAACTCAAACTTACCCTAGGGCAGTCAGCCGCCACCCAACTCGACAAAACATGGCTAGAAACGATCGGACCAAAAGGCCTGGCACTAGCACAAAAAAACATAGCAAAAGCTACAAATAACATCTCACGAGGACTAATTAAAACGTACCTAACTATCTTCCTCTTAACCCTAGTCCTAACCACTATCTTAATTTCACTTTAA

>A._wenchowensis_1

ATGACACTAATCATACACTCGTCCCTTCTCCTAACCCTCCTTATTTTACTCCACCCACTACTTACCACACTCAGCCCAAACCAACAAGAATCTAATATAGCAAAAACCACTAAAACTGCCATTAGCTCAGCCTTCTTCATCAGCCTCCTACCTCTCACAATTTTCCTAGCTCAAGGAACAGAAAGCATCGTGACAAACTGACAATGAATAAACACCCAAACATTCGATGTAAACATCAGCTTTAAATTTGATCACTACTCCTTAATCTTTATCCCAATTGCCTTATACGTCACCTGATCAATTCTTGAATTTTCACTGTGGTATATGCACTCCGACCCTAACATCAACCAATTCTTTAAGTACCTACTTGTGTTCTTAGTAGCCATAGTTATCTTAGTCTCAGCTAACAATATCTTCCAACTATTCATCGGCTGGGAAGGGGTAGGAATCATATCCTTCCTTCTTATCGGGTGATGATACGGCCGAGCGGATGCTAACACAGCTGCCCTCCAAGCCGTTATTTACAACCGAGTGGGAGACATCGGACTAATTATAACTATAGCCTGATTCGCAATAAACCTCAACTCCTGAGAAATCCAACAAATCTTAACCCTATCAAAAGACTTTAATATAACACTTCCCCTAATTGGACTCATCTTGGCAGCAACAGGAAAATCCGCTCAATTTGGCCTTCACCCCTGACTCCCAGCCGCCATAGAAGGCCCTACCCCAGTGTCTGCCCTACTCCACTCGAGCACCATAGTCGTTGCAGGGATCTTCCTACTAATCCGCTTTCACCCTCTCATAGAAAATAATCAACTGGCACTGACAACTTGTCTCTGCCTTGGAGCACTTACCTCACTATTCGCAGCCACCTGCGCCCTCACCCAAAATGATATCAAAAAAATTGTAGCTTTCTCAACATCAAGCCAGCTAGGCCTAATAATAGTAGCAATCGGGTTAAATCAACCACAACTGGCATTCCTTCATATCTGCACCCACGCCTTTTTCAAGGCTATACTATTCCTGTGCTCAGGATCAATTATTCACAGCCTAAATGATGAACAAGACATCCGAAAAATAGGAGGCCTACTCAACATTATACCCACCACCTCTACTTACTTCACAATTGGTAGCCTAGCCCTAACAGGAACCCCCTTCCTGGCAGGGTTCTTCTCAAAAGACGCAATCATCGAAACCCTAAACTCTTCATCCCTAAACGCCTGAGCCCTTATCCTGACATTAATTGCCACATCTTTTACAGCTGTATATAGTTTTCGCCTAGTATATTTTGTAATCATAGGAGCTCCCCGATTCCTGCCCCTATGCCCGATTAACGAAAACAACCCATTAGTAATTAACCCCCTCAAACGACTTGCCTGAGGAAGCATCACCGCAGGACTAATTATCACCCAAAACTTCTTACCAATAAAAACACCAGTTATAACAATACCAACCCCCTTAAAAATAGCGGCTCTACTAGTAACAATTGCAGGCCTGCTGACAGCCATAGAACTAGCCAACATAACAAGTAAACAAGTGAAGATCACCCCCATAACCCACACACACCACTTCTCAAACATATTAGGATTCTTCCCTATGATCACACATCGACTAATCCCAAAACTTAAACTTACCCTAGGACAATCAGCCGCCACCCAACTCGACAAGACATGACTCGAAGCAATTGGACCAAAAGGCCTAGCATTAACACAAAAGAATATAGCAAAAGCCACAAATAACATCTCACGAGGAATAATTAAAACATACCTAACCATCTTCCTATTAACCCTAATCCTGGCCACCATCCTAATTTCACTTTAG

>A._wenchowensis_2

ATGACACTAATCATACACTCGTCCCTTCTCCTAACCCTCCTTATTTTACTCCACCCACTGCTCACCACACTTAATCCAAACCAACAAGAATCTAATATAGCAAAAACCACTAAAACTGCCATTAGCTCAGCCTTCCTCATCAGCCTCCTGCCTCTCACAATTTTCCTGGCTCAAGGAACAGAAAGCATCGTAACAAACTGACAATGAATAAACACCCAAACATTCGATGTAAACATCAGCTTTAAATTTGATCACTACTCCTTAATCTTTATCCCAATTGCCCTATATGTCACCTGATCAATTCTTGAATTTTCACTCTGATACATGCACTCCGACCCCAACATCAACCAATTCTTTAAATATCTACTTGTATTCTTAGTAGCCATAATTATCTTAGTTTCAGCTAACAACATCTTCCAACTATTCATCGGCTGAGAGGGAGTAGGAATTATATCCTTCCTTCTCATCGGGTGGTGATATGGCCGGGCGGATGCTAACACAGCCGCCCTCCAAGCCGTTATTTACAACCGAGTGGGGGACATCGGACTAATCATAACTATAGCCTGATTCGCAATAAACCTCAACTCCTGAGAAATCCAACAAATCTTAACCCTATCAAAAGACTTTGATATGACCCTTCCCCTAATTGGACTCATCTTAGCAGCAACAGGAAAATCCGCCCAATTTGGCCTCCACCCCTGACTTCCAGCCGCCATAGAAGGCCCTACCCCAGTGTCTGCCCTACTCCACTCAAGCACCATGGTTGTTGCAGGAATCTTCCTACTAATCCGCTTTCACCCTCTCATAGAGAATAATCAACTGGCACTAACAACTTGCCTCTGCCTTGGAGCACTAACCTCGCTATTCGCAGCCACCTGCGCCCTTACCCAAAATGATATCAAAAAAATTGTAGCTTTCTCAACATCAAGCCAGCTAGGCCTAATAATAGTCGCAATCGGGTTAAATCAACCACAACTAGCATTCCTTCATATCTGCACCCACGCCTTTTTCAAAGCTATACTATTCCTGTGTTCAGGGTCAATTATTCACAGCCTAAATGACGAACAAGACATCCGTAAAATAGGAGGCCTACTCAACATTATGCCCACCACCTCTACTTACTTCACAATTGGTAGCCTAGCCCTAACAGGAACCCCCTTCCTAGCAGGGTTCTTCTCAAAAGACGCAATCATCGAAGCCCTAAACTCTTCATCCCTAAACGCCTGAGCCCTCGTCCTGACATTAATTGCCACATCTTTTACAGCTGTATATAGTTTTCGCCTAGTATATTTTGTAATCATAGGAGCTCCCCGATTCCTACCCCTATGCCCGATTAACGAAAACAACCCATTAGTAATTAACCCCCTCAAACGACTTGCCTGAGGAAGCATCACCGCAGGACTAATTATCACCCAAAACTTTTTACCAATAAAAACACCAGTTATAACAATACCAACCCCCTTAAAAATAGCGGCTCTCCTAGTAACAATTGTAGGCCTGCTAACAGCCATAGAACTAGCCAACATAACAAGTAAACAAGTGAAGATTACCCCAATAACCCACACACATCACTTCTCAAACATACTAGGATTCTTCCCTATAATTACACATCGACTAATCCCAAAACTTAAACTCACCCTAGGACAATCAGCCGCCACCCAGCTCGACAAAACATGGCTCGAAACAATTGGGCCAAAAGGCCTGGCACTAACACAAAAAAATATAGCAAAAGCCACAAATAACATCTCACGAGGAATAATTAAAACATACCTAACCATCTTCCTCTTAACCCTAATCCTAGCCACCATCCTAATTTCACTTTAA

>A._wuyiensis

ATGACATTAATCATACACTCATCCCTCCTCCTCACTCTTCTTATCCTACTCCACCCACTACTTACCACACTTAACCCAGACCAACAAGGGTCTAACATGGCAGAAACCACCAAAACTGCCATCAGCACCGCCTTCTTCGTTAGCCTCTTACCCCTTATAATTTTCCTAGCCCAAGGGACAGAAAGCATTGTAACAAGCTGACAATGAATAAACACCCAAACGTTCGATGTAAACATCAGCTTTAAGTTTGACCACTACTCCTTAATCTTTACCCCAATTGCCCTATACGTCACCTGATCAATCCTTGAATTCTCACTATGATATATACACTCTGACCCCAACATTAACCGATTCTTTAAGTATCTCCTCGTATTCTTAGTAGCCATAATCATCTTAGTCTCAGCTAATAACATTTTCCAACTATTTATTGGTTGAGAAGGAGTAGGGATTATATCATTCCTCCTCATTGGATGATGATACGGCCGGGCAGATGCTAACACAGCTGCCCTCCAAGCCGTCATTTACAACCGAGTAGGAGACATCGGACTAATTATAACTATAGCCTGGTTCGCAATAAACCTCAACTCCTGAGAAATTCAACAAATCTTGACACTATCAAAAGACTTTAACATGACACTTCCCCTAATTGGGCTCATCTTAGCAGCAACAGGAAAATCCGCCCAATTCGGCCTGCACCCCTGACTCCCAGCCGCCATAGAAGGCCCTACCCCAGTATCTGCCCTACTCCACTCCAGCACCATGGTCGTTGCAGGAGTCTTCCTACTAATCCGCTTTCACCCCCTCATAGAAAATAATCAACTAGCACTAACAACTTGCCTCTGTCTTGGAGCATTAACCTCACTATTCGCAGCCACCTGCGCCCTCACCCAAAATGATATCAAAAAAATCGTAGCTTTCTCAACATCAAGCCAACTAGGCCTAATAATGGTCGCAATCGGACTAAATCAACCACAACTAGCATTCCTTCACATCTGCACCCACGCCTTCTTTAAAGCAATACTATTCCTGTGTTCAGGATCAATTATCCACAGCCTAAATGACGAGCAAGACATCCGAAAAATAGGGGGCCTACTCAACACCATACCTGCCACCTCTACCTACTTCACAATTGGCAGCCTAGCCCTAACAGGAACCCCATTCCTAGCAGGATTCTTCTCAAAAGACGCAATCATCGAAGCCCTAAATACCTCATCCCTAAACGCCTGAGCCCTTGTCCTAACACTAATTGCTACCGCATTCACGGCAGTATATAGCTTCCGCCTACTATACCTCGTAATCATAGGAGCCCCCCGATCCCTACCCCTATGCCCTATTAATGAAAACAACCCATTAGTAATCAACCCCCTCAAACGACTTGCCTGAGGAAGCATCACCGCAGGATTTATTATTACTCAAAATCTTCTACCAATAAAAACACCAGTTATAACTATACCAACCTCCCTAAAAATAGCAGCTATCCTAGTGACAATCGCAGGTCTACTGACAGCCATAGAACTGGCTAACATAACAAGCAAACAAGTAAAAATTACCCCAACAATTCACACACACCACTTCTCAAATATACTAGGATTCTTCCCCACAATTGTACACCGACTTATCCCAAAACTTAAACTTACCCTAGGACAGTCAGCCGCCACCCAACTCGACAAAACATGACTAGAAACAATCGGGCCAAAAGGCCTGGCATCAACACAAAAAAATATAGCAAAAGCTACGAATAACATCTCACGAGGAATAATTAAAACATATCTAACCATTTTCCTCTTAACCCTAACCTTAGCCACTATCTTAATCTCACTCTAA

>A._yunnanensis_1

ATGACATTAATCATACACTCGTCCCTCCTCCTAACCTTTCTCATCCTACTTCACCCACTACTTACCACACTTAACCCCCGCCAACAAGAACTACACATAGCAAAAACCACCAAAACCGCCATCAGCTCCGCATTCTTTATCAGTCTCCTGCCACTTACAATTTTCCTAACTCAGGGGACAGAAAGCATTGTAACAAACTGACAATGAATAAACACCCAAACATTTGACGTAAACGTTAGCTTTAAGTTTGATCACTACTCCCTAATTTTTATTCCGATTGCCTTATACGTCACCTGATCAATCCTTGAATTTTCACTATGGTACATACACTCTGACCCTAACATCGACCGATTCTTTAAATACTTACTAGTTTTCTTAGTAGCCATAATTATTCTAGTCTCAGCCAACAACATTTTCCAACTATTTATTGGCTGAGAGGGGGTAGGAATTATATCATTTCTACTCATCGGATGGTGGTATGGACGGACAGATGCCAATACAGCAGCCCTCCAAGCCGTTATTTATAACCGGGTAGGAGACATCGGACTAATTTTAACCACAGCTTGGTTCGCAATAAACCTCAACTCCTGAGAAATTCAACAAATCCTAACACTATCAAAAGACTTTGACACAACCCTTCCCCTAATTGGACTCATCTTAGCAGCAACTGGAAAATCAGCCCAATTTGGCCTTCACCCTTGACTCCCAGCCGCCATAGAAGGCCCTACCCCAGTGTCTGCCCTACTCCATTCAAGTACTATGGTTGTTGCAGGAATCTTCTTACTAATCCGCTTCCACCCCCTCATAGAAGACAATCAACTAGCCCTAACAATCTGTCTATGCCTTGGAGCACTAACCTCTTTATTCTCAGCCACTTGCGCCCTAACCCAAAATGATATCAAAAAAATTGTAGCTTTCTCAACAGCAAGCCAACTAGGCCTAATAATGGTCGCAATTGGATTAAACCAACCACAACTAGCATTCCTCCACATCTGCACTCATGCCTTTTTCAAGGCCATATTATTCCTATGCTCAGGGTCAATCATCCACAGCCTAAACGACGAGCAAGACATCCGAAAAATAGGGGGCCTATTTAACATCATACCTGCTACCTCAACCTACTTCACAATCGGCAGCCTGGCCCTAACAGGAACCCCATTCCTAGCGGGATTCTTCTCAAAAGACGCAATCATTGAAGCCCTGGGCACCTCTTATCTTAACGCCTGAGCCCTAACCCTAACACTAATTGCCACATCATTCACCGCTGTATATAGCTTCCGACTAGTATACTTCGTAATCATAGGAGCCCCACGATTCCTTCCCCTATGTCCAATTAACGAAAACAACCCACTAGTAATTAACCCCCTTAAACGACTTGCCTGAGGAAGCATCATTGCAGGGCTCATTATTACCCAAAACCTCTTACCAATAAAAACACCAATTATAACAATACCAACCGCCCTAAAAACAGCAGCCCTCATAGTAACAATTGTGGGCCTACTAACAGCCATAGAACTAGCCAACATAACAAGCAAACAAGTAAAAATTACCCCAACAATCCCCACACATCACTTCTCAAACATATTAGGATTCTTCCCAACAGTCGTACACCGACTCATCCCAAAGCTTAAACTTACCCTGGGACAATCAACCGCCACCCAACTCGACAAGACATGGCTCGAAACAGTTGGACCAAAAGGCCTAGCTCAAACACAAAAAACCATGGCAAAAGCCACAAACAATATCTCACGAGGAATAATTAAGACGTACCTAACCATCTTCCTCCTAACCCTAATTATAGCCTCCATCCTAGTCCTTCTTTAA

>A._yunnanensis_2

ATGACATTAATCATACACTCGTCCCTCCTCCTAACCTTTCTCATCCTACTTCACCCACTACTTACCACACTTAACCCCCACCAACAAGAATTACACATAGCAAAAACCACCAAAACCGCCATCAGCTCCGCATTCTTTATCAGCCTACTGCCACTTACAATTTTCCTAACTCAGGGGACAGAAAGCATTGTAACAAACTGACAATGAATAAACACCCAAACATTTGACGTAAACGTTAGCTTTAAGTTTGATCACTACTCCCTAATTTTTATTCCAATTGCCTTATACGTCACCTGATCAATCCTTGAATTTTCACTATGATACATACACTCTGACCCTAACATCGACCGATTCTTTAAATACTTACTAGTTTTCTTAGTAGCTATAATTATTCTAGTCTCAGCCAACAACATTTTCCAACTATTTATTGGCTGAGAAGGGGTAGGAATTATATCATTTCTACTCATCGGATGGTGGTATGGACGGACAGATGCCAATACAGCAGCCCTCCAAGCCGTTATTTATAACCGGGTAGGGGACATCGGACTAATTTTAACCACAGCTTGGTTCGCAATAAACCTCAACTCCTGAGAAATTCAACAAATCCTAACATTATCAAAAGACTTTGACACAACCCTTCCCCTAATTGGACTCATCTTAGCAGCAACAGGAAAATCAGCCCAATTTGGCCTTCACCCTTGACTCCCCGCCGCCATAGAAGGCCCTACCCCAGTGTCTGCCCTACTCCATTCAAGTACCATGGTTGTTGCAGGAATCTTCTTACTAATCCGCTTCCACCCCCTCATAGAAGACAATCAACTAGCCCTAACAATCTGTCTATGCCTTGGAGCACTAACCTCTTTATTCTCAGCCACTTGCGCCCTAACCCAAAATGATATCAAAAAAATTGTAGCTTTCTCAACAGCAAGCCAACTAGGCCTGATAATGGTCGCAATTGGATTAAACCAACCACAACTAGCATTCCTCCATATCTGCACTCATGCCTTTTTCAAGGCCATATTATTCCTATGCTCAGGGTCAATTATCCACAGCCTAAACGACGAGCAAGACATCCGAAAAATAGGGGGCCTATTTAACATCATACCTGCTACCTCAACCTACTTCACAATCGGCAGCCTGGCCCTAACAGGAACCCCATTCCTAGCGGGGTTCTTCTCAAAAGACGCAATCATTGAAGCCCTGGGCACCTCTTATCTTAACGCCTGAGCCCTGACCCTAACACTAATTGCCACATCATTCACCGCAGTATATAGCTTCCGACTAGTATACTTCGTAATCATAGGAGCCCCGCGATTCCTTCCCCTATGTCCAATTAACGAAAACAACCCACTAGTAATTAACCCCCTTAAACGACTTGCCTGAGGAAGCATCACTGCAGGACTCATTATTACCCAAAACCTCTTACCAATAAAAACACCAATCATGACAATACCAACCACCCTAAAAACAGCAGCCCTCATAGTAACAATTGTGGGCCTACTAACAGCCATAGAACTAGCTAACATAACAAGCAAACAAGTAAAAATTACCCCAACAATCCCTACACATCACTTCTCAAACATATTAGGATTCTTCCCAACAGTCGTACACCGACTCATCCCAAAGCTTAAACTTACCCTAGGGCAATCAGCCGCCACCCAGCTCGACAAGACATGGCTCGAAACAATTGGACCAAAAGGTCTAGCTGAAACACAAAAAACCATGGCAAAAGCCACAAACAATATCTCACGAGGAATAATTAAGACATACCTAACCATCTTCCTCCTAACCCTGATTATGGCCTCCATCCTAGTCCTTCTTTAA

>Onychostoma_barbatulum

ATGACACTAATCATACACTCATCCCTCCTCCTAACCTTTCTTATTCTACTTCACCCCCTACTTACCACACTAAACCCCAACCAACAAGAATCCAAAATGGCAGAGACCACCAAAACCGCTATTAGCTCCGCATTCTTCATCAGCCTCCTACCACTTTTAATTTTCCTAACCCAAGGAACAGAAAGTATCGTAACGAACTGACAATGAATAAATACCCAAACATTTGACGTAAACATTAGCTTTAAATTTGACCACTACTCCCTAATCTTTATTCCAATCGCCCTGTACGTTACCTGGTCAATCCTTGAATTTTCACTATGATACATACACTCTGACCCCAACATCAATCGATTCTTTAAATACCTACTCGTATTCTTAGTAGCCATAATTATTCTAGTCTCAGCTAACAACATCTTCCAACTATTTATTGGCTGAGAGGGGGTAGGAATTATGTCATTCCTACTCATTGGATGATGATACGGACGAGCAGATGCCAACACAGCAGCCCTTCAAGCCGTTATCTACAACCGAGTAGGAGACATTGGACTAATTATAACCACAGCTTGATTCGCAATAAACATTAACTCCTGAGAAATTCAACAAATCCTAATTTTATCAAAAGACTTTAACATAACCCTCCCCCTAGCTGGTCTAATCTTAGCAGCCACAGGAAAATCTGCACAGTTCGGCCTACACCCCTGACTCCCAGCCGCCATAGAAGGCCCTACCCCAGTATCTGCCCTACTCCATTCCAGCACCATAGTTGTTGCAGGGGTATTCCTTCTAATCCGCTTCCACCCCCTCATAGAAGATAACCAATTAGCCCTAACAACATGTCTCTGCCTTGGAGCACTAACTTCACTATTCGCAGCCACCTGCGCCCTCACCCAAAATGACATCAAAAAAATTGTAGCTTTCTCAACATCAAGCCAGCTAGGCCTAATAATAGTCGCAATCGGACTAAACCAACCCCAGCTAGCATTCCTACACATCTGTACTCACGCCTTTTTCAAAGCCATGTTATTCCTATGCTCAGGATCAATCATCCACAGCCTAAACGACGAACAAGACATCCGAAAAATGGGAGGTTTGTTTAACACTATGCCCGCCACCTCTACCTACTTCACAATCGGCAGCCTAGCTCTTACAGGAACCCCATTCTTAGCAGGATTCTTCTCAAAAGACGCAATCATTGAAGCCCTCAACACCTCTCATCTTAACGCCTGAGCCCTAACCCTAACACTAATTGCTACATCCTTCACCGCAGTATATAGCTTCCGTCTAGTGTACTTTGTTATTATGGGAGCCCCACGATTCCTTCCCCTATGCCCAATTAACGAAAACAACCCACTAGTAATTAATCCCCTCAAACGACTTGCTTGAGGAAGCATCATCGCGGGACTTGTTATTACCCAAAACCTTCTACCAATAAAAACACCAATTATAACAATACCAACTACCCTAAAAATAGCAGCTCTCATTGTAACAATTATAGGCCTACTAACAGCCATAGAACTGACTAACATAACAAGTAAACAAGTAAAAATTATCCCAACAATTAACACACACCATTTCTCAAATATACTGGGATTCTTCCCCATGACAGTACACCGACTCATCCCAAAGCTTAAACTTACCCTAGGGCAATCAGCCGCCACCCAGCTCGACAAAACATGACTTGAAATAATTGGACCAAAAGGCCTGGCACTAACCCAAAAAAGTATGGCAAAAACCACAAATAATATTTCACGAGGAATAATCAAAACATACCTAACCATCTTCCTCCTAACCCTAACCTTGACTACCATTATAATTCTCCTCTAA

>Onychostoma_meridionale

ATGACACTAATCATACACTCATCCCTCCTATTAACCTTCCTCATCCTACTTCACCCACTACTTACCACATTAAACCCCAACCAAAAAGAAGCCAAAATGGCAGAGACTACCAAGACTGCTATCAGCTCCGCATTCTTCATAAGCCTTCTACCACTTACGATCTTCCTAGCCCAAGGAACAGAAAGCATCGTAACGAACTGACAATGAATAAACACCCAAACATTTGACGTAAATATCAGCTTTAAGCTTGACCACTACTCCCTAGTCTTTATTCCAATTGCCCTATACGTCACTTGATCAATCCTCGAATTTTCACTATGATACATACACTCTGACCCCAACATTGACCGATTCTTCAAATACCTCCTTGTATTCCTAGTAGCCATAATCATACTAGTATCAGCTAACAACATCTTCCAACTATTCATTGGCTGAGAAGGGGTAGGGATTATGTCATTTCTACTCATCGGGTGATGGTACGGACGAGCAGACGCTAATACAGCAGCCCTCCAAGCCGTTATCTACAACCGTGTAGGAGATATCGGATTAATTATAACCACAGCTTGATTTGCAATAAACACTAACTCCTGAGAAATCCAACAAATCTTAGCACTATCAAAAGACTTTGATATAACACTCCCCCTGTTTGGCCTTGCCTTAGCTGCAACAGGGAAATCCGCTCAATTCGGCCTCCACCCCTGACTCCCGGCCGCCATAGAGGGCCCCACCCCAGTATCTGCCCTACTCCACTCTAGCACCATGGTCGTTGCAGGAGTATTCCTTCTAATCCGCTTCCACCCCCTCATGGAGAACAACCAACTAGTCCTGACTACCTGTCTCTGCCTCGGAGCATTAACCTCGCTATTTGCAGCCACCTGCGCCCTCACCCAAAATGATATCAAAAAAATCGTAGCTTTTTCAACATCAAGCCAACTAGGCCTGATAATAGTTGCAATTGGACTAAACCAACCTCAACTAGCATTCTTCCACATCTGCACCCACGCTTTTTTCAAGGCCATACTATTCCTGTGCTCAGGATCAATTATTCATAGCCTAAATGATGAACAAGACATCCGAAAAATAGGGGGCCTATTAAATATTATGCCCGCCACCTCCGCCTACTTCACAATCGGCAGCCTAGCCCTCACAGGAACCCCGTTCCTAGCAGGATTCTTCTCGAAAGACGCAATTATTGAAGCCCTAAACACCTCTTATCTTAACGCCTGAGCCCTAACCCTAACACTAATCGCCACATCATTCACCGCAGTATATAGCTTCCGCCTAGTTTATTTCGTAGTTATAGGGGCCCCGCGATTCCTCCCCCTGTGCCCGATTAACGAAAACAACCCACTAGTGATTAACCCCCTTAAGCGACTTGCCTGAGGAAGCATCATCGCAGGACTCATCATTACTCAAAACCTCCTGCCAATAAAAACACCAGTCATGACAATATCGCCCACCTTAAAAATAGCAGCCCTCATTGTAACAGCCATAGGACTATTAACAGCCATAGAATTAACCAACATAACAAGCAAGCAAGTAAAAATTACCCCAACAATCCACGCACACCACTTCTCAAACATACTAGGATTCTTCCCTATGACAGTGCACCGACTTATTCCAAAACTTAAACTCACCCTAGGACAATCGGCCGCCACCCAGCTCGACAAAACATGGCTCGAAACAATTGGACCAAAAGGCCTAGCACTAGCACATAAAAATATGGCAAAAACCACAAACAACATCTCACGGGGAATAATTAAAACATACCTAACTATCTTTCTTATAACCCTAGTTTTAGCCACCATCTTAACGCTCCTCTAA

>Onychostoma_gerlachi

ATGACACTAATCATACACTCATCCCTCCTCTTAACTTTTCTCATCCTACTCCACCCACTACTCACCGCGCTAAATCCTAACCAAAAAGAATCCAAAATGGCAGATAAAACCAAAACTGCTATCAGCTCTGCATTCTTCATAAGCCTCCTACCACTTACGATTTTCCTAACCCAAGGGACAGAAAGCATCGTAACTAACTGACAATGAATAAACACCCAAACATTTGACGTAAACATCAGCTTTAAGCTTGACCACTACTCCCTAGTCTTTATTCCAATCGCCCTATACGTCACATGATCAATCCTCGAATTTTCATTATGATACATACACTCTGACCCCAACATTGACCGATTCTTCAAATACCTTCTTGTATTCTTAGTAGCCATAATTATACTAGTATCAGCTAACAACATCTTTCAACTATTCATTGGCTGAGAAGGGGTAGGAATTATGTCATTCCTACTCATCGGATGATGATACGGACGGGCAGACGCTAACACGGCAGCCCTCCAAGCCGTTATTTACAACCGTGTAGGAGACATCGGATTAATTATAACTATGGCTTGATTTGCAATAAACATTAACTCCTGAGAAATCCAACAAATCTTAGTATTATCAAAAGACTTTGATATAACACTCCCCCTAATTGGCTTTGCCTTAGCTGCAACAGGAAAATCCGCCCAATTCGGCCTCCACCCCTGACTCCCGGCCGCCATAGAGGGCCCTACCCCAGTATCTGCCCTACTACACTCTAGTACCATGGTCGTTGCAGGAGTATTCCTTCTAATCCGCTTCCACCCCCTCATGGAAAACAACCAACTAGTCCTAACTATCTGTCTCTGCCTCGGAGCACTAACCTCACTATTTGCAGCCACCTGCGCTCTAACCCAAAATGACATCAAAAAAATCGTAGCTTTTTCGACATCAAGCCAACTAGGCCTGATAATAGTTGCAATCGGGCTAAACCAACCTCAACTAGCATTCTTCCACATCTGCACCCACGCTTTTTTCAAGGCCATATTATTCCTGTGCTCAGGATCAATTATTCATAGCCTAAATGACGAACAAGACATCCGAAAAATAGGAGGCCTATTAAACATCATACCTGCCACCTCTGCCTACTTCACAATCGGCAGCCTGGCCCTCACAGGAACCCCATTCCTAGCAGGATTCTTCTCAAAAGACGCAATTATTGAAGCCCTAAACACCTCTTACCTTAACGCCTGAGCCCTAACCCTAACACTAATCGCCACATCATTCACCGCAGTATATAGCTTCCGCCTAGTGTACTTTGTAATCATAGGAGCCCCACGATTCCTCCCCCTGTGCCCAATTAACGAAAACAACCCACTAGTAATTAACCCCTTAAAACGACTTGCCTGAGGAAGCATCATCGCAGGACTCATCATCACTCAAAACCTCCTACCAATAAAAACACCAATCATGACAATACCGCCCGCTTTAAAAATAGCAGCCCTCATTGTAACAGTCATGGGACTATTAACAGCCATAGAATTAACCAATATAACAAGCAAACAAGTAAAAATTACCCCAACAATCCACATACACCATTTCTCAAATATACTGGGATTCTTCCCCATAACAGTACACCGACTTATTCCAAAACTTAAACTCACCTTAGGGCAATCAGCCGCCACTCAACTCGACAAAACATGACTAGAAACAATTGGACCAAAAGGCTTAGCGCTAACACAAAAAAATATGGCAAAAACCACAAATAACATCTCACGAGGAATGATCAAAACATACCTAACTATCTTCCTTATAACCCTAGTTTTAGCCACCGTCCTAACTCTCCTCTAA

>Spinibarbus_denticulatus

ATGACACTAATAATACACTCATCGCTTCTCCTAATCTTTTTCATCTTAACTTACCCATTATTCAATATGCTAAGCTCTGATCAACAAGAATCTGACATAGCAAAAATAGTCAAAACTGCCGTCAGCTCTGCATTCTTCACCAGCCTACTACCACTTATAGTTTTTCTAAACCTAAAAACAGAAGGCATCATTACGAATTGACAATGAATAAACACCCAAACATTTGACGTAAACATTAGCCTTAAATTTGATCACTACTCCTTAATCTTTGTCCCAATTGCCCTATATGTTACCTGGTCAATTCTAGAATTTGCATTATGGTATATACACTCTGACCCTAATATTAATCGATTTTTCAAATACTTACTCACATTCTTAGTAGCCATAATTATTTTAGTCACAGCCAACAACATATTCCAACTATTTATTGGCTGAGAGGGAGTAGGAATTATATCATTTCTACTTATTGGGTGATGACACGGACGAGCAGATGCTAACACAGCAGCCCTCCAAGCTGTTATTTACAACCGAACAGGGGATATTGGGCTAATCTTAACCATGGCCTGATTTGCAATAAACCTTAATTCCTGGGAAATCCAACAAATTTTTACCTTATCGAAAAACTTCAATATAACAATTCCTCTAATAGGACTTATCTTAGCAGCAACAGGAAAATCGGCCCAATTCGGCCTACATCCTTGACTCCCTTCTGCCATGGAGGGCCCTACGCCAGTATCCGCCCTACTCCACTCCAGCACCATGGTCGTTGCAGGAATCTTCCTACTAATTCGTCTTCACCCCTTAATAGAAAACAACAACCTAGCACTAACAATTTGCCTCTGCTTAGGAGCACTCACCACACTATTTACAGCTACTTGTGCCTTGACCCAAAATGACATCAAAAAAATCGTAGCTTTCTCAACATCCAGCCAACTAGGCCTAATAATAGTCACAATTGGGCTAAACCAGCCACAACTAGCATTTCTCCACATTTGCACACACGCATTCTTTAAAGCCATACTATTCTTATGCTCCGGATCAATTATCCACAGCCTAAATGATGAACAAGACATCCGAAAAATAGGAGGCCTCCACAACTTAATACCTGCCACTTCAACCTACCTTACAATTGGTAGCTTAGCACTAACAGGAACCCCATTCCTAGCCGGATTCTTCTCAAAAGATGCTATTATTGAAGCCCTAAACACCTCTTACCTTAACGCCTGAGCCCTAACCCTTACACTAATTGCCACATCATTTACCGCAGTCTACAGCTTCCGAGTAGTATTCTTCGTAACTATGGGATCTCCCCGATTCCTGCCACTATCCCCCATCAACGAGAATAATCCACTAGTAATCAACCCAATCAAACGACTTGCCTGAGGAAGCATTATTGCAGGACTAATCATCACACAAAACTTCCCACCAATGAAAACACCAATTATAACAATACCAACTACCCTAAAAATAGCAGCCCTCATAGTAACAATCGCAGGCCTATTAATAGCCATAGAACTAACCAATATAACAAGCAAACAAGTAAAAATTACCCCAATAATTCCCGCACATCACTTTTCAAATATATTAGGATTCTTCCCTGCAATTACTCACCGACTTCTCCCAAAGCTTAAGCTCACCCTAGGACAATCAACCGCCACCCAACTCGACAAAACATGACTCGAAGCCATTGGACCAAAAGGTTTAGCACTAACTCAGATAACTATAGCAAAACTTACAAATGACACCTCACAAGGAATAATTAAAACATACCTAACCATTTTTCTCCTAACCTTCACTTTAGCTATCACCCTAACCCTCCTCTAA

>Spinibarbus_hollandi

ATAACACTAATAATACACTCATCACTTCTTCTAATCTTTTTCATCCTAATTTATCCACTACTCACCATACTAACCTCCGATCAACAACAATCTGACATAGCAAAAATAGTCAAAACTGCTGTCAGCTCCGCATTCTTCACCAGCCTACTACCACTTATAATTTTTCTAAACCTAAAAACAGAAGGCATCATTACGAATTGACAATGAATAAATACCCAAACATTTGACGTAAACATTAGCCTTAAGTTTGACCATTACTCCTTAATCTTCGTCCCAATCGCCCTGTATGTTACTTGATCAATTCTAGAATTTGCGTTATGGTATATACACTCTGACCCTAATATTAATCGATTTTTCAAATACTTACTCACATTCTTAGTAGCCATAATTATTTTAGTCACAGCTAACAACATATTTCAACTATTTATTGGCTGAGAAGGAGTAGGAATTATATCATTTCTACTTATTGGATGATGGCACGGACGAGCGGATGCTAATACAGCAGCTCTCCAAGCTGTTATTTATAACCGAACAGGAGATATTGGACTAATCTTAACCATGGCCTGGTTTGCAATAAACCTTAATTCCTGGGAAATACAACAAATTTTTACCTTATCGAAAACCTTCAATATAACAATTCCTCTAATAGGACTTATCTTAGCGGCAACAGGAAAATCGGCCCAATTCGGCCTTCACCCCTGACTTCCTTCTGCCATGGAGGGCCCCACCCCAGTATCTGCCCTACTCCATTCAAGTACTATGGTTGTTGCAGGAATCTTCCTACTAATCCGCCTCCACCCCCTCATAGAAAATAACCAATTAGCCTTAACAACCTGCCTTTGCCTCGGAGCACTAACCTCACTATTTACAGCCACTTGCGCCCTGACCCAAAATGACATCAAAAAAATTGTAGCTTTCTCCACATCAAGTCAGCTAGGTTTAATAATAGTCACAATTGGGTTAAACCAGCCACAACTAGCGTTCCTCCATATTTGTACCCACGCCTTCTTTAAAGCCATACTATTTTTATGCTCAGGGTCAATCATTCACAGTCTAAACGATGAACAAGACATCCGAAAAATAGGGGGACTACTTAACACTATACCCATCACCTCAACCTATTTCACAATCGGCAGCCTAGCCCTAACAGGAACCCCATTTCTAGCAGGGTTCTTTTCAAAAGATGCAATTATTGAAGCCCTAAACACCTCCTATTTAAACGCCTGAGCCCTAATCCTAACACTTACAGCCACATCATTTACCGCAGTGTACAGCTTCCGACTAGTATATTTTGTGATTATAGGAACCCCACGATTCCCATCTTTATTACCAATTAATGAGAATGACCCACTAATAATTAACTCCATTAAACGACTTGCCTGAGGAAGCATCATCGCAGGACTAATCATCACACAAAACTTCCCACCAATAAAAACACCAATCATAACAATACCGACTCCCCTAAAAATAGCAGCCCTCATAGTAACAATCGCAGGCCTACTAATAGCTATAGAACTGACCAACATAACAAGCAAACAAGTAAAAATTACCCCAATAATCCCCGCACATCACTTTTCAAATATATTAGGATTCTTCCCCGCAATTACTCACCGACTTCTCCCAAAACTTAAACTCACCCTGGGACAATCAACCGCCACCCAGCTCGACAAAACATGACTCGAAGCCATTGGACCAAAAGGTTTAGCACTAACTCAGATAACTATGGCAAAACTTACAAATGATACCTCCCAAGGAATAATTAAAACATACCTAACCATTTTTCTCCTAACCTTCACTTTAGCCATTACCCTAACCCTCCTCTAA

>Spinibarbus_sinensis

ATGACACTAATTATACACTCATCACTTCTTCTAATCTTTTTCATCTTAATATACCCACTACTCACCACACTAAACCCCAACCAACAAAAATCCAACATAGCAGAAATAACCAAAACTGCCGTTAGTTCCGCATTCTTCGTCAGCCTCCTACCACTTATAATCTTTCTAAACCTAAAAACAGAAGGTATCATTACGAACTGACAATGAATAAACACCCAAACATTTGACGTAAATATTAGCTTTAAATTCGACCACTATTCCCTAATCTTTGTTCCAATTGCCCTATACGTTACCTGATCAATTCTAGAGTTCGCATTATGATACATACACTCTGACCCCAATATTAACCGATTCTTTAAATATTTACTCACATTTTTAGTAGCCATAATTATTCTAGTCACAGCTAATAATATATTTCAACTATTTATTGGCTGAGAAGGAGTAGGAATTATATCCTTCCTACTTATCGGATGATGACATGGACGAGCAGATGCTAACACAGCAGCCCTTCAAGCTGTCATCTATAACCGAGTAGGAGATATTGGACTAATTATAACCATAGCCTGACTTGCAGTAAACCTCAACTCCTGAGAAATCCAACAAATTTTTACCTTATCAAAAAACTTTGACATAACAATTCCTCTAATAGGACTTGCCTTGGCGGCAACAGGAAAATCAGCCCAATTTGGCCTCCACCCCTGACTTCCATCCGCCATAGAGGGCCCCACGCCAGTATCTGCCCTACTACATTCAAGTACTATAGTTGTTGCAGGAATTTTCCTACTAATCCGTCTCCACCCCCTCATAGAGAATAACCAACTAGCCCTGACAATCTGTCTCTGCCTTGGAGCACTAACCTCACTATTTACAGCCACTTGCGCCTTAACCCAGAATGATATCAAAAAAGTTGTAGCTTTCTCAACATCAAGCCAATTAGGTCTAATAATAGTTACAATCGGATTAAATCAACCACAATTAGCATTTCTTCACATCTGCACCCACGCCTTTTTCAAAGCTATACTATTCTTGTGCTCAGGATCAATTATTCATAGCCTAAACGATGAACAAGACATCCGAAAAATAGGAGGCCTATTCAACATTATACCCGCCACCTCAACCTACTTCACAATCGGTAGCTTAGCCCTAACAGGAACCCCGTTCCTAGCAGGGTTCTTCTCAAAAGACGCAATCATTGAAGCCCTAAACACCTCTTACCTAAACGCCTGAGCCCTAACCTTAACACTAATCGCCACATCATTTACCGCGGTATACAGCTTCCGACTAGTATATTTTGTAGTCATAGGAACCCCGCGATTCCTGCCCCTATCACCAATCAACGAAAACAACCCACTAGTAATCAATTCTATTAAACGACTTGCCTGAGGAAGCATCATCGCAGGACTTATTATTACACAAAACTTTCCGCCAATAAAAACGCCAATCATAACAATACCAACTACCCTAAAAATAGCAGCCCTCTTAGTGACAATCACGGGCCTACTAGTAGCCATAGAATTAGCCAACATAACAAGCAAGCAAGTAAAAATTATCCCAATTATTCCCACACACCACTTTTCAAACATATTAGGATTCTTCCCCGCAATTATCCACCGGCTCCTTCCAAAACTTAAACTCACCCTAGGGCAGTCAGCCGCTACCCAACTCGACAAAACATGACTCGAAGCCATTGGACCAAAAAGCCTGGCACTAACACAAATAACCATGGCAAAATTTACAAACGACATCTCACGAGGAATAATTAAAACATACCTAACTATCTTCCTTTTAACCCTAATCTTAGCTACTATCCCAATTCTCCTTTAA

**ND6**

>A._barbodon

ATGACCTATTTTATGTCCTTGTTATTGATGGCTCTGGTTGCGGGTTTAGTTGCTGTTGCTTCTAATCCTACGCCTTATTTTGCTGCATTTGGTTTAGTAATTGCGGCTGGGGTTGGGTGTGGGGTTTTGGTGGGCCATGGGGGTTCTTTTTTATCGCTGGTTCTTTTTTTGATTTATTTAGGGGGGATGCTCGTGGTTTTTGCCTATTCGGCAGCTTTAGCTGCTGAGCCTTTCCCAGAGGCTTGAGGAAGTCGTTCTGTGTTGGGTTATGTTTTAGTTTATTTGTTAGGGGTTGGGTTAATGGCGGGGGTATTCTGAGGGGGTTGATATGAAGGCTCATGGGCGGTTGTAGACGGGTTGAAAGAGTTTTCTGTGTCGCGTGGCGATATAAGTGGGGTGGCCGTGATGTACTCGTTTGGTGGGGGTATGCTAGTTATTTGTGCTTGGGTGTTGCTTTTGACTTTGCTTGTTGTATTAGAGCTTACTCGTGGCTTAAGTCGTGGGACGTTACGAGCGGTT

>A._beijiangensis_1

GTGACATTTGTTCTGGTTTTATTTTTAATTGTTTTAATTCTAGGTTTAATTGCTGTTTCTTCTAATCCTGCACCTTATTTTGCAGCGTTTGGTTTAGTGGTCGCGGCAGGGGCTGGGTGTGGGATTTTGGTGAATTATGAGGGCTCTTTTTTATCACTAGTTCTTTTTCTAATTTATTTAGGGGGGATGCTTGTAGTTTTCGCCTATTCGGCAGCTTTAGCTGCTGAGCCTTTTCCAAAAGCTTGAGGCAGTCTCTCTGTGCTCGGTTATGTTTTAATTTATTTAGTTGGGGTTGTTGTGGTAGCGGGGTATCATTGGGATGGTTGATACCAAAGCTCGTGAATGATTGGGGAGGAGGCACATGAGTTCTCTGTTGTACATGCTGATATTAGTGGGGTGGCTGTGTTATATTCATCTGGTGGGGCTGTATTAATTACTTGTGCTTGGGTCTTGCTTTTGACTCTGTTTGTTGTGTTGGAGCTTACTCGTGGTCTGAGTCGGGGGACGCTTCGGGCAGTT

>A._beijiangensis_2

ATGACATTTGTTCTGGTTTTATTTTTAATTGTTTTAATCCTAGGTTTAATTGCTGTTGCTTCTAATCCTGCACCTTATTTTGCAGCGTTTGGTTTAGTGGTTGCGGCAGGGGCTGGGTGCGGGATCTTGGTGAGTTATGGGGGCTCTTTTTTATCACTAGTTCTTTTTCTAATTTATTTAGGGGGGATGCTCGTAGTTTTCGCCTATTCGGCAGCTTTAGCTGCTGAGCCTTTTCCAAAAGCTTGAGGCAGTCTCTCTGTGCTCGGTTATGTTGTAATTTATTTAGTTGGGGTTGTTGTGGTAGCGGGGTATTATTGGGATGGTTGATACCAAAGCTCGTGAATGATTGGGGAGGAGGCACATGAGTTCTCTGTTGTAGGTGCTGATATTAGTGGGGTGGCTGTGTTATATTCATCTGGTGGGGCTGTATTAATTACTTGCGCTTGGGTCTTGCTTTTGACTTTGTTTGTTGTGTTGGAGCTTACTCGTGGTCTGAGCCGGGGGACGCTTCGGGCGGTT

>A._fasciatus

ATGATCTTTATTCTGGTTTTGTTCTTAATTGGTTTAATTCTGGGCTTAATCGCTGTTGCTTCTAATCCTACGCCCTATTTCGCTGCGTTTGGGTTAGTAATTGCGGCGGGGGTTGGGTGTGGGATTTTGGTGGGCCATGGGGGCTCTTTTTTATCACTAGTTCTTTTTCTAATCTATCTAGGGGGGATGCTCGTTGTTTTTGCCTATTCGGCAGCTTTAGCTGCTGAGCCCTTCCCGGAGGCTTGAGGTAGTCGTACTGTGCTTGGTTATGTCTTAGCTTATTTAGTTGGGGTTGGTTTAGTAGCAGGGTTCTTTTGAGAGGGTTGGTTTGAGGGTTCGTGGACGGTTGTGGATGGGCTAAAAGAGTTTTCTGTTTTACGTGCTGATATCAGCGGGGTGGCTGTGATGTACTCATTTGGTGGGGGGATATTAATTATCTGCGCTTGGGTGTTGCTTTTGACCTTGCTTGTTGTGTTGGAGCTTACTCGTGGTCTGAGCCGGGGGACGTTACGGGCGGTT

>A._hemispinus

ATGATTTTTATTCTGGTTTTGTTCTTAATTGGTCTAATTCTGGGTTTAATTGCCGTCGCTTCTAATCCAACGCCTTATTTTGCTGCGTTTGGGTTAGTAATTGCGGCGGGGGTTGGGTGTGGGATTCTGGTGGGCCATGGGGGCTCTTTTCTATCACTAGTTCTTTTTTTAATTTATTTAGGGGGAATGCTCGTTGTCTTTGCCTATTCGGCAGCTTTAGCTGCTGAGCCTTTTCCGGAAGCTTGGGGGAGTCGTACTGTGTTTGGTTACGTTTTAGTTTATTTAGTTGGGGTTGGTTTAGTAGCGGGGTTTTTCTGGGAGGGCTGATTTGAGGGCTCATGGACAGTTGTGGATGGGTTAAAAGAGTTTTCTGTTTTACGTGCCGATGTTAGTGGGGTGGCTGTAATGTACTCATTTGGTGGGGGTATGTTAATTATTTGTGCTTGGGTGTTGCTTTTGACTTTGCTTGTTGTGTTGGAGCTTACTCGTGGTTTAAGCCGGGGGACGTTGCGGGCGGTT

>A._iridescens

ATGACCTATTTTATGTCCTTGTTATTGATGGCTCTGGTTGCGGGTTTAATTGCTGTTGCTTCTAATCCTACGCCTTATTTTGCTGCATTTGGTTTAGTAATTGCGGCTGGGGTTGGGTGTGGGGTTTTGGTGGGCCATGGGGGTTCTTTTTTATCGCTGGTTCTTTTTCTGATTTATTTAGGGGGGATGCTCGTGGTTTTTGCCTATTCGGCAGCTTTAGCTGCTGAGCCTTTCCCGGAGGCTTGAGGAAGTCGTTCTGTGTTGGGTTATGTTTTAGTTTATTTATTAGGGGTTGGGTTAATGGCTGGGGTATTCTGAGGGGGTTGATATGAAGGTTCATGGGCGGTTGTAGACGGGCTGAAAGAGTTTTCTGTGTCGCGTGGCGATATAAGTGGGGTGGCCGTGATGTACTCGTTTGGTGGGGGTATGCTAGTTATTTGTGCTTGGGTGTTGCTTTTGACTTTGCTTGTTGTATTAGAGCTTACTCGTGGCCTAAGTCGTGGGACGTTACGAGCGGTT

>A._jishouensis

ATGATCTTTATTCTGGTTTTGTTCTCAATTGGTCTACCTCTGGGTCTAGTCGCCGTCGCTTCTAATCCTACGCCTTATTTTGCTGCGTTTGGATTAGTAATTTCGGCGGGGGTTGGGTGTGGGCTTTTGGTGGGCCATGGGGGCTCTTTTTTATCATTAGTTCTTTTTTTAATTTATTTAGGGGGGATGCTCGTTGTTTTTGCCTATTCGGCAGCTTTGGCTGCTGAGCCTTTCCCGGAAGCTTGGGGTAGTCGTGCTGTGTTAGGTTATGTTTTAGTTTATTTAGTTGGGGTTAGTTTAGTAGCAGGGTTTTTCTGAGAGGGTTGATTTGAGGGTTCATGGACGGTTGTGGATGGATTAAAAGAGTTTTCTGTTTTACGTGCCGATATTAGTGGGGTGGCTGTAATGTACTCATTTGGTGGAGGCATGTTAATTATTTGCGCTTGGGTATTACTTTTGACCCTGCTTGTTGTGTTGGAGCTTACCCGTGGTTTGAGTCGGGGGACGTTACGGGTGGTT

>A._kreyenbergii_1

ATGATCTTTATTCTGGTTTTGTTTTTAATTGGTCTGATTCTGGGTCTAATCGCTGTCGCTTCTAATCCTACGCCCTATTTTGCTGCGTTTGGGTTGGTAATTGCGGCGGGGGTTGGGTGTGGGATTTTGGTGGGCCATGGGGGCTCTTTTTTATCACTAGTTCTTTTTTTAATTTATTTAGGGGGGATGCTCGTTGTTTTTGCCTATTCGGCAGCTTTAGCTGCTGAGCCCTTTCCGGAAGCTTGGGGTAGTCGTACTGTGCTTGGTTATGTTTTAGTTTATCTAGTTGGGGTTAGTTTAGTAGCAGGGTTCTTTTGAGAGGGTTGGTTTGAGGGCTCATGGACGGTTGTGGACGGGCTAAAAGAGTTTTCTGTATTACGTGCTGATATCAGCGGGGTGGCTGTAATGTACTCATTTGGTGGGGGTATGTTAGTTATTTGTGCCTGGGTGTTGCTTCTGACCTTGCTTGTTGTGTTGGAGCTTACTCGTGGTCTGAGTCGGGGCACGCTACGGGCGGTC

>A._kreyenbergii_2

ATGATCTTTATTCTGGTTTTGTTTTTAATTGGTCTAATTCTGGGTCTGATCGCTGTCGCTTCTAATCCTACGCCCTATTTTGCTGCGTTTGGGTTAGTAATTGCGGCGGGGGTTGGGTGTGGGATTTTGGTGGGCCATGGGGGCTCTTTTTTATCATTAGTTCTTTTTTTAATTTATCTAGGGGGGATGCTCGTCGTTTTTGCCTATTCGGCAGCTTTAGCTGCTGAGCCCTTTCCGGAAGCTTGGGGTAGTCGTGCTGTGCTTGGTTATGTCCTAGTTTATTTAGTCGGGGTTGGCTTAGTAGCAGGGTTCTTCTGAGAGGGTTGGTTCGAGGGTTCATGGACGGTTGTGGACGGGTTAAAAGAGTTTTCTGTATTACGTGCTGATATCAGCGGGGTGGCTGTAATGTATTCGTTTGGCGGGGGCATGTTAATTATTTGTGCTTGGGTGTTGCTTTTGACCTTGCTTGTTGTGTTGGAGCTTACTCGTGGTCTGAGTCGGGGCACGTTACGGGTGGTT

>A._longipinnis

ATGACCTATTTTATGTCCTTGTTATTGATGGCTCTGGTTGCGGGTTTAGTTGCTGTTGCTTCTAATCCTACGCCTTATTTTGCTGCATTTGGTTTAGTAATTGCGGCTGGGGTTGGGTGTGGGGTTTTGGTGGGCCATGGGGGTTCTTTTTTATCGCTGGTTCTTTTTCTGATTTATTTAGGGGGGATGCTCGTGGTTTTTGCCTATTCGGCAGCTTTAGCTGCTGAGCCTTTCCCAGAGGCTTGAGGAAGTCGTTCTGTTGTGGGTTATGTTTTAGTTTATTTATTAGGGGTTGGGTTAATGGCTGGGGTATTCTGAGGGGGTTGATATGAAGGTTCATGGGCGGTTGTAGACGGGTTGAAAGAGTTTTCTGTGTCGCGTGGCGATATAAGTGGGGTGGCCGTGATGTACTCGTTTGGTGGAGGTATGCTAGTTATTTGTGCTTGGGTGTTGCTTTTGACTTTGCTTGTTGTATTAGAGCTTACTCGTGGCTTAAGTCGTGGGACGTTACGAGCGGTT

>A._monticola_1

ATGACCTATTTTATGTTTATGTTATTGATGGCTTTGGTTGTGGGTTTAGTTGCTGTGGCATCTAATCCTACGCCATACTTTGCTGCGTTAGGTTTGGTAGTTGCAGCTGGGGTTGGGTGTGGGGTTTTGGTGGGCCATGGGGGTTCTTTTTTATCGTTAGTTCTTTTTCTAATTTATTTAGGGGGGATGCTCGTGGTTTTTGCCTATTCGGCAGCTTTAGCTGCTGAGCCTTTCCCGGAGGCTTGAGGTAGTCGTTCTGTGTTGGGTTATGTTTTGGTTTATTTACTAGGGGTTAGTCTGGTAGCGGGGTTGTTTTGAGGGGGGTGATACGAGGGTTCATGGACGGTTGTGGACGGGTTAAAAGAATTCTCTGTTTTGCGTGGTGATGTTAGTGGTGTGGCTGTAATATATTCGTTTGGTGGGGGGATGTTAGTTATTTGCGCTTGGGTGTTGCTTTTGACTTTGCTTGTTGTGTTAGAGCTTACTCGTGGTTTAAGTCGTGGGACTTTGCGTGCGGTT

>A._monticola_2

ATGACCTATTTTATGTTTATGTTATTGATGGCTTTGGTTGTGGGTTTAGTTGCTGTGGCATCTAATCCTACGCCATACTTTGCTGCGTTAGGTTTGGTAGTTGCAGCTGGGGTTGGGTGTGGGGTTTTGGTGGGCCATGGGGGTTCTTTTTTATCGTTAGTTCTTTTTCTAATTTATTTAGGGGGGATGCTCGTGGTTTTTGCCTATTCGGCAGCTTTAGCTGCTGAGCCTTTCCCGGAGGCTTGAGGTAGTCGTTCTGTGTTGGGTTATGTTTTGGTTTATTTACTAGGGGTTAGTCTGGTAGCGGGGTTGTTTTGAGGGGGGTGATACGAGGGTTCATGGACGGTTGTGGACGGGTTAAAAGAATTCTCTGTTTTGCGTGGTGATGTTAGTGGTGTGGCTGTAATATATTCGTTTGGTGGGGGGATGTTAGTTATTTGCGCTTGGGTGTTGCTTTTGACTTTGCTTGTTGTGTTAGAGCTTACTCGTGGTTTAAGTCGTGGGACTTTGCGTGCGGTT

>A._paradoxus_1

ATGACATTTGTTCTGGTTTTATTTTTAATTGTTCTGATTTTAGGCCTAATTGCTGTTTCTTCTAATCCTGCGCCTTATTTTGCGGCGTTTGGTTTAGTAGTTGCGGCGGGGGCTGGGTGTGGGATTTTGGTGGGCCATGGGGGCTCTTTTTTATCACTAGTTCTTTTTCTAATTTATTTAGGGGGGATACTTGTAGTTTTTGCTTATTCGGCAGCCTTAGCTGCTGAGCCTTTTCCGGAAGCTTGAGGTAGTCTTTCTGTGCTTGGTTATGTTTTAGTTTATTTAGTTGGGGTTGGTGTGGTAGCGGGGTATAATTGAGATGGTTGATACCAGAGCGCATGGATGATTGCGGAGGGGGCGGAAGAGTTCTCTGTTACACGTGCTGATATCAGTGGGGTGGCTGTGTTATATTCATCTGGTGGGGCTGTATTAGTCACTTGCGCTTGGGTCTTGCTTCTGACTCTGTTTGTTGTGTTAGAGCTTACTCGTGGTTTAAGTCGGGGGACGCTTCGGGCGGTT

>A._paradoxus_2

ATGATCTTTATTCTGGTTTTGTTCTCAATTGGTCTACCTCTGGGTCTAGTCGCCGTCGCTTCTAATCCTACGCCTTATTTTGCTGCGTTTGGATTAGTAATTTCGGCGGGGGTTGGGTGTGGGATTTTGGTGGGCCATGGGGGCTCTTTTTTATCATTAGTTCTTTTTTTAATTTATTTAGGGGGGATGCTCGTTGTTTTTGCCTATTCGGCAGCTTTGGCTGCTGAGCCTTTCCCGGAAGCTTGGGGTAGTCGTGCTGTGTTAGGTTATGTTTTAGCTTATTTAGTTGGGGTTAGTTTAGTAGCAGGGTTTTTCTGAGAGGGTTGATTTGAGGGTTCATGGACGGTTGTGGATGGATTAAAAGAGTTTTCTGTTTTACGTGCCGATATTAGTGGGGTGGCTGTAATGTACTCATTTGGTGGAGGCATGTTAATTATTTGCGCTTGGGTATTACTTTTGACCCTGCTTGTTGTGTTGGAGCTTACTCGTGGTTTGAGTCGGGGTACGTTACGGGTGGTT

>A._parallens_1

ATGATCTTTATTCTGGTTTTGTTCTTAATTGGTCTAATTCTGGGTCTAATCGCTGTCGCTTCTAATCCAACGCCTTATTTTGCTGCGTTTGGGTTAGTAATTGCGGCGGGGGTTGGGTGTGGGATTCTGGTGGGCCATGGGGGCTCTTTTCTATCACTAGTTCTTTTTTTAATTTATTTAGGGGGAATGCTCGTTGTCTTTGCCTATTCGGCAGCTTTAGCTGCTGAGCCTTTTCCGGAAGCTTGGGGCAGTCGTACTGTGTTTGGTTATGTTTTAGTTTATTTAGTTGGGGTTGGTTTAGTAGCGGGGTTTTTCTGGGAGGGCTGATTTGAGGGCTCATGAACGGTTGTGGATGGGCTAAAAGAGTTTTCCGTTTTACGTGCCGATGTTAGTGGGGTGGCTGTAATGTACTCATTTGGCGGGGGCATGTTAATTATTTGTGCTTGGGTATTGCTTTTGACTTTGCTTGTTGTGTTGGAGCTTACTCGCGGTTTGAGTCGGGGGACGCTGCGGGCGGTT

>A._parallens_2

ATGATCTTTATTCTGGTTTTGTTCTTAATTGGTCTAACTCTGGGTCTAATCGCCGTCGCTTCTAATCCAACGCCTTACTTTGCTGCGTTTGGGTTAGTAATTGCGGCGGGGGTTGGGTGTGGGATTCTGGTGGGCCATGGGGGCTCTTTTCTATCACTAGTTCTTTTTTTAATTTATTTAGGGGGAATGCTCGTTGTTTTTGCCTATTCGGCAGCTTTAGCTGCTGAGCCTTTCCCGGAAGCTTGGGGCAGTCGTACTGTGTTTGGCTATGTTTTAGTTTATGTAGTGGGGGTTGGTCTAGTAGCGGGGTTTTTCTGGGAGGGGTGATTTGAGGGCTCATGGACGGTTGTGGATGGGTTAAAAGAGTTTTCTGTTTTACGTGCCGATGTTAGTGGGGTGGCTGTAATGTACTCATTTGGTGGGGGCATGTTAATTATTTGTGCTTGGGTGCTGCTTTTGACTTTGCTTGTCGTGTTGGAGCTTACTCGTGGTTTGAGTCGGGGGACGCTGCGGGCGGTT

>A._parallens_3

ATGATCTTTATTCTGGTTTTGTTCTTAATTGGTCTCATTCTGGGTCTAATCGCCGTCGCTTCTAATCCAACGCCTTATTTTGCTGCGTTTGGGTTAGTAATTGCGGCGGGGGTTGGGTGTGGGATTCTGGTGGGCCATGGGGGCTCTTTTCTATCACTAGTTCTTTTTTTAATTTATTTAGGGGGAATGCTCGTTGTTTTTGCCTATTCGGCAGCTTTAGCTGCTGAGCCTTTCCCGGAAGCTTGGGGCAGTCGTACTGTGTTTGGTTATGTTTTAGTTTATTTAGTCGGGGTCGGTTTACTAGCGGGGTTTTTCTGGGAGGGCTGATTTGAGGGTTCATGGACGGTTGTAGATGGGCTAAAAGAGTTTTCTGTTTTACGTGCCGATGTTAGTGGGGTGGCTGTAATGTACTCATTTGGTGGGGGCATGTTAATTATTTGTGCTTGGGTGTTGCTTTTGACTTTGCTTGTTGTGTTGGAGCTTACTCGTGGTTTGAGTCGGGGGACGCTGCGGGCGGTT

>A._parallens_4

ATGATCTTTATTCTGGTTTTGTTCTTAATTGGTCTAATTCTGGGTCTAATCGCCGTCGCTTCTAATCCAACGCCTTATTTTGCTGCGTTTGGGTTAGTAATTGCGGCGGGGGTTGGGTGTGGGATTCTGGTGGGCCATGGGGGCTCTTTTCTATCACTAGTTCTTTTTTTAATTTATTTAGGGGGAATGCTCGTTGTTTTTGCCTATTCGGCAGCTTTAGCTGCTGAGCCTTTCCCGGAAGCTTGGGGCAGTCGTACTGTGTTTGGTTATGTTTTAGTTTATTTAGTCGGGGTTGGTTTAGTAGCGGGGTTTTTCTGGGAGGGCTGATTTGAGGGCTCATGGACGGTTGTGGACGGGCTAAAAGAGTTTTCTGTTTTACGTGCCGATGTTAGTGGGGTGGCTGTAATGTACTCATTTGGTGGGGGCATGTTAATTATTTGTGCTTGGGTGTTGCTTTTGACTTTGCTTGTTGTGTTGGAGCTTACTCGTGGTTTGAGTCGGGGGACGCTGCGGGCGGTT

>A._spinifer

ATGACATTTGTTCTGGTTTTATTTTTAATTGTTCTAATTCTAGGTTTAATTGCTGTTGCTTCTAATCCTGCACCTTATTTTGCAGCGTTTGGTTTAGTGATTGCAGCAGGGGCTGGGTGTGGGATTTTGGTGGGTTATGGGGGCTCTTTTTTATCATTAGTTCTTTTTCTAATTTATCTAGGAGGGATGCTTGTAGTTTTCGCCTATTCGGCAGCTTTAGCTGCTGAGCCTTTCCCGGAAGCTTGAGGTAGTCTTTCTGTGCTTGGTTATGTTGTAGTTTATTTAGTTGGGGTTATTGTGGTAGCGGGGTATTATTGGGATGGTTGATACCAAAGCTCGTGAATGACTGGGGAGGGGGCACATGAGTTCTCTGTTGTACGTGTTGATATTAGTGGGGTGGCTGTGCTATATTCATCTGGTGGGGCTGTATTAATTATTTGTGCTTGGGTCTTGCTTTTGACTTTGTTTGTTGTGTTGGAGCTTACTCGTGGTCTAAGTCGGGGGACGCTTCGGGCGGTT

>A._stenotaeniatus

ATGACATTTGTTCTGGTTTTATTTTTAATTATTCTAATTCTAGGTTTAATTGCTGTTGCTTCTAATCCTGCACCTTATTTTGCAGCGTTTGGTTTAGTGATTGCAGCAGGGGCTGGGTGTGGGATTTTGGTGGGTTATGGGGGCTCTTTTTTATCATTAGTTCTTTTTCTAATTTATCTAGGGGGGATGCTTGTAGTTTTCGCCTATTCGGCAGCTTTAGCTGCTGAGCCTTTCCCGGAAGCTTGAGGTAGTCTTTCTGTGCTTGGTTATGTTGTAGTTTATTTAGTTGGGGTTATTGTGGTAGCGGGGTATTATTGGGATGGTTGATACCAAAGCTCGTGAATGACTGGGGAGGGGGCACATGAGTTCTCTGTTGTACGTGTTGATATTAGTGGGGTGGCTGTGTTATATTCATCTGGTGGGGTTGTATTAATTATTTGTGCTTGGGTCTTGCTTTTGACTTTGTTTGTTGTGTTGGAGCTTACTCGGGGTTTAAGTCGGGGGACGCTTCGGGCGGTT

>A._wenchowensis_1

ATGATCTTTATTCTGGTTTTGTTCTTAATTGGTCTAATTCTGGGTCTAATCGCCGTCGCTTCTAATCCCACGCCTTATTTTGCTGCTTTTGGGTTAGTAATTGCGGCGGGGGTTGGGTGTGGGATTTTGGTGGGCCATGGGGGCTCTTTTTTATCATTAGTTCTTTTTTTAATTTATCTAGGGGGGATGCTCGTCGTTTTTGCCTATTCGGCAGCTTTGGCTGCTGAGCCCTTCCCGGAAGCTTGAGGTAGTCGTACTGTGCTTGGTTATGTTTTAGTTTATTTAGTTGGGGTTGTTTTAGTAGCAGGGTTCTTTTGAGAGGGTTGGTTTGAGGGTTCGTGGACGGTCGTGGACGGGTTGAAAGAGTTTTCTGTTTTACGTGCTGATGTCAGCGGGGTGGCTGTGATGTACTCATTTGGTGGGGGCATATTAATTATTTGTGCTTGGGTGCTGCTTTTGACCCTGCTTGTTGTGTTGGAGCTTACCCGTGGTTTGAGTCGGGGGACGCTACGGGTGGTC

>A._wenchowensis_2

ATGATCTTTATTCTGGTTTTGTTCTTAATTGGTCTAATTCTGGGTCTAATCGCTGTCGCTTCTAATCCTACGCCCTATTTTGCTGCGTTTGGGTTAGTAATTGCGGCGGGGGTTGGGTGTGGGATTTTGGTGGGCCATGGGGGCTCTTTTTTATCATTAGTTCTCTTTTTAATTTATTTAGGGGGGATGCTCGTTGTTTTTGCCTATTCGGCAGCTTTAGCTGCTGAGCCCTTCCCGGAAGCTTGAGGTAGTCGTACTGTGCTTGGTTATGTTTTAGTTTATTTAGTTGGGGTTAGTTTAGTGGCAGGGTTCCTTTGAGAGGGTTGGTTCGAGGGTTCGTGGACGGTTGTGGATGGGCTAAAAGAGTTTTCTGTTTTACGTGCTGATATCAGCGGGGTGGCTGTGATGTACTCATTTGGTGGGGGCATACTGATTATTTGTGCCTGGGTGCTGCTTTTGACCTTGCTTGTTGTGTTGGAGCTTACTCGTGGTCTGAGTCGGGGGACACTACGGGCGGTT

>A._wuyiensis

ATGACATTTGTTCTGGTTTTATTCTTAATTGTTCTGATTCTAGGCCTAATTGCTGTTTCTTCTAATCCTGCGCCTTATTTTGCGGCGTTTGGTTTAGTAGTTGCGGCGGGGGCTGGGTGTGGGATTTTGGTGGGCCATGGGGGCTCTTTTTTATCACTAGTTCTTTTTCTAATTTACTTAGGGGGGATACTTGTAGTTTTCGCTTATTCGGCAGCCTTAGCTGCTGAGCCTTTTCCGGAAGCTTGAGGTAGTCTTTCTGTGCTTGGTTATGTTTTAGTTTATTTAGTTGGGGTTGGTGTGGTAGCGGGGTATAATTGAGATGGTTGATACCAGAGCGCATGGATAATTGCGGAGGGGGCGGAAGAGTTCTCTGTTACACGTGCTGATATTAGTGGGGTGGCTGTGTTATATTCATCTGGTGGGGCTGTATTAGTCACTTGCGCCTGGGTCTTGCTTCTGACTCTGTTTGTTGTGTTAGAGCTTACTCGTGGTTTAAGTCGGGGGACGCTTCGGGCGGTT

>A._yunnanensis_1

ATGACTTATTTTATGTTCTTATTATTAATAGCTCTGGTTGTGGGTTTAGTTGCTGTTGCATCTAATCCTACACCCTATTTTGCTGCGTTAGGTTTGGTAGTTGCGGCTGGGGTCGGGTGTGGAGTTTTGGTGGGCCATGGGGGTTCTTTTTTATCATTAGTTCTTTTTCTAATTTATTTAGGGGGGATGCTCGTGGTTTTTGCCTATTCGGCAGCTTTAGCTGCTGAGCCTTTTCCAGAGGCTTGAGGTAGTCGTTCTGTGTTGGGCTATGTTTTAGTTTATTTATTAGGGGTTGGTCTAATGGCGGGGTTATTTTGAGGAGGATGATACGAGGGTTCGTGGACGGTTGTGGATGGGTTGAAAGAATTTTCTGTTTTGCGTGGTGATGTTAGTGGTGTGGCTGTGATATATTCGTTTGGTGGGGGCATGCTAGTTATTTGTGCTTGGGTGTTGCTTTTAACCTTGCTTGTTGTGCTAGAGCTTACCCGTGGTCTAAGTCGTGGGACTTTGCGTGCGGTT

>A._yunnanensis_2

ATGACTTATTTTATGTTCTTATTATTAATAGCTCTGGTTTTGGGTTTAGTTGCTGTTGCATCTAATCCTACACCCTATTTTGCTGCGTTAGGTTTGGTAGTTGCGGCTGGGGTCGGGTGTGGAGTTTTGGTGGGCCATGGGGGTTCTTTTTTATCATTAGTTCTTTTTCTAATTTATTTAGGGGGGATGCTCGTGGTTTTTGCCTATTCGGCAGCTTTAGCTGCTGAGCCTTTTCCAGAGGCTTGAGGTAGTCGTTCTGTGTTGGGCTATGTTTTAGTTTATTTATTAGGGGTTGGTCTGGTGGCGGGATTATTTTGAGGAGGATGATATGAGGGTTCGTGGACGGTTGTGGACGGGTTGAAAGAATTTTCTGTTTTGCGTGGTGATGTTAGTGGTGTGGCTGTGATATATTCGTTTGGTGGGGGCATGCTAGTTATTTGTGCTTGGGTGTTGCTTTTAACCTTGCTTGTTGTGCTAGAGCTTACCCGTGGTCTAAGTCGTGGGACTTTGCGTGCGGTT

>Onychostoma_barbatulum

ATGACCTATTTTATGTCTTTATTATTAATAGCTTTGGTTGTGGGTTTAGTTGCTGTTGCTTCTAATCCTACACCTTACTTTGCTGCGTTGGGTTTAGTAGTTGCGGCTGGGGTTGGGTGTGGGGTTTTGGTGGGCCATGGGGGTTCCTTTTTATCATTAGTTCTTTTTCTAATTTATTTAGGGGGAATACTTGTGGTTTTTGCCTATTCGGCAGCTTTGGCTGCTGAGCCTTTCCCGGAGTCTTGGGGTAGTCGTTCTGTGCTGGGTTATGTTTTGGTTTATTTACTGGGGGCTGGTCTAATGGCGGGGTTATTTTGAGGGGGTTGGTATGAAGGTTCGTGGGTGGTTGTAGATGGGTTGAAAGAGTTTTCTGTTTTGCGTGGTGATGTAGGTGGTGTAGCTGTAATATATTCGTTTGGTGGGGGTATGTTGGTTATTTGTGCTTGGGTGTTGCTTTTGACTTTATTAGTTGTGTTAGAGCTTACTCGTGGTTTAAGTCGTGGGACTCTGCGTGCAGTT

>Onychostoma_meridionale

ATGACCTATTTTATGTCCTTGTTGTTAATAGCTTTGGTTGTGGGTTTAGTTGCTGTTGCCTCTAACCCTACGCCTTATTTTGCTGCGTTAGGTTTAGTGGTTGCGGCTGGGGTTGGGTGTGGGGTTTTGGTGGGCCATGGGGGCTCTTTTTTATCGTTAGTTCTTTTTTTAATTTATTTAGGGGGAATGCTCGTAGTTTTTGCCTATTCGGCAGCTTTGGCTGCTGAGCCCTTTCCAGAGGCTTGGGGTGCTCGTTCTGTGTTGGGCTATGTTTTAGTTTATTTACTGGGGGCTGGTTTGGCGGCGGGGTTATTTTGAGGGGGTTGGTACGAGGGTTCGTGAACAGTTGTGGATGGGTTGAAAGAATTTTCTGTTTTTCGTGGAGATGTTAGTGGTGTAGCTGTAATGTACTCGTTTGGTGGGGGTATGCTGGTTATTTGCGCTTGGGTGTTGCTTTTAACTTTGCTTGTTGTGTTGGAGCTTACTCGTGGTCTGAGTCGTGGGACCTTACGTGTGGTT

>Onychostoma_gerlachi

ATGACCTATTTTATGTCTTTATTGTTAATAGCTTTGGTTGTGGGTTTAGTTGCTGTTGCTTCTAATCCTACACCCTATTTTGCTGCGTTAGGTTTGGTGGTTGCGGCCGGGGTTGGGTGCGGGGTTTTGGTGGGCCATGGGGGTTCTTTTTTATCGTTAGTTCTTTTTTTAATTTATTTAGGGGGAATGCTTGTAGTTTTTGCCTATTCGGCAGCTTTGGCTGCTGAACCCTTTCCAGAGGCTTGGGGTGCTCGTTCTGTGCTGGGCTATGTTTTAGTTTATTTACTGGGGGTTGGTTTAGTGGCAGGGTTATTTTGAGGGGGTTGATATGAGGGTTCGTGGACAGTAGTAGATGGGCTGAAAGAATTTTCTGTTTTTCGTGGTGATATTAGTGGTGTAGCTGTGATATACTCGTTTGGCGGGGGCATGCTAGTTATTTGTGCTTGGGTATTACTTTTAACCCTGCTTGTTGTGTTGGAGCTTACTCGTGGTCTGAGTCGTGGGACCCTGCGTGCGGTT

>Spinibarbus_denticulatus

ATGACCTATTTTATGTTTTTATTATTGGTGGCTTTGGTTATAGGTTTAGTTGCTGTTGCTTCTAATCCTACGCCATACTTTGCTGCGCTTGGTTTGGTTGTTGCAGCTGGGGTTGGGTGTGGGGTTTTGGCTGGTCATGGGGGCTCTTTTTTATCATTAGTCCTTTTTTTAATTTATTTAGGGGGGATGCTTGTAGTTTTTGCCTATTCGGCAGCTTTAGCTGCTGAGCCTTTTCCAGAGGCTTGGGGCAGTCGTTCTGTATTGGGCTATGTTTTAGTTTATTTATTAGGGGTTGGTTTGGCGGCGGAGTTATTTTGAGGGGGTTGATATGAGGGTTCATGGGTGGTTGTGGACGGGTTGAAAGAATTTTCTGTCTTGCGGGGTGATATTAGTGGTGTGGCTGTAATATATTCGTCTGGTGGAATTATGTTGGTTATTTGTGCTTGGGTATTGCTTTTAACTTTGCTTGTTGTGTTGGAAATTACTCGTGGTTTAAGTCGTGGAAGTCTTCGTGCGGTT

>Spinibarbus_hollandi

ATGACCTATTTTATGTTTTTGTTATTGGTGGCCCTGGTTATAGGTTTAGTTGCTGTTGCTTCTAATCCTACGCCGTATTTTGCTGCGCTTGGTTTGGTCGTTGCAGCTGGGGTTGGGTGTGGGGTTTTGGCTGGCCATGGGGGCTCCTTTTTATCACTAGTTCTTTTTTTAATTTACTTAGGGGGGATGCTTGTAGTTTTTGCTTATTCGGCAGCTTTAGCTGCCGAACCTTTTCCAGAGGCTTGGGGTAACCGCTCTGTGTTAGGTTATGTTTTGGTTTATTTATTAGGGGTTAGTTTGGCGGCGGAGATATTTTTGGGGGGTTGATATGAGGGTTCGTGAGTGGTGGTGGACGGGTTGAAAGAATTTTCTGTTTTGCGGGGCGATATTAGTGGTGTGGCTATAATATATTCATCTGGTGGGGTCATGTTGGTTATTTGTGCTTGGGTGTTGCTTTTAACTTTGCTTGTTGTATTGGAGATTACTCGTGGTTTAAGTCGTGGGGGTCTTCGTGCGGTT

>Spinibarbus_sinensis

ATGACCTATTTTATGTTTTTGTTACTAGTAGCTTTGGTTATAGGCTTAGTTGCTGTTGCTTCTAATCCTACTCCTTATTTTGCTGCGCTTGGTTTGGTTGTTGCAGCTGGGGTTGGGTGTGGAATTTTGGTTAGTCATGGAGGTTCTTTTCTGTCATTGGCCCTCTTTTTAATTTATTTAGGAGGGATGCTTGTAGTTTTTGCTTATTCGGCAGCTTTGGCCGCTGAGCCTTTTCCAGAGGCTTGGGGTAGTCGTTCTGTGTTGGGTTATGTTTTAGTTTATCTATTAGGGGTTAGTTTAGTGGCAGGGTTATTTTGAGGGGGTTGATATGAGGGTTCATGGGTGGTTGTGGATGGGTTGAAGGAATTTTCTGTTTTGCGTGGGGATATTAGTGGTGTGGCTGTAATATATTCGTCTGGTGGGGGTATACTAGTTGTTTGTGCTTGGGTATTGCTTTTAACACTGCTTGTTGTGCTAGAGCTTACTCGTGGTCTTAGTCGTGGGACTCTTCGTGCGGTT
