# Supplementary material for: Natural selection drives the evolution of mitogenomes in Acrossocheilus
Source: PLoS One. 2022 Oct 13;17(10):e0276056. doi: 10.1371/journal.pone.0276056 (PMC9560497; doi:10.1371/journal.pone.0276056)
Supplement: Attachment — Submitted filename: Response to Reviewers.docx [file pone.0276056.s005.docx]

PONE-D-22-15319

Natural selection drives the evolution of mitogenomes in Acrossocheilus

PLOS ONE

Dear Dr. Gao,

Thank you for submitting your manuscript to PLOS ONE. After careful consideration, we feel that it has merit but does not fully meet PLOS ONE’s publication criteria as it currently stands. Therefore, we invite you to submit a revised version of the manuscript that addresses the points raised during the review process.

Please submit your revised manuscript by Sep 29 2022 11:59PM. If you will need more time than this to complete your revisions, please reply to this message or contact the journal office at [plosone@plos.org](mailto:plosone@plos.org). When you're ready to submit your revision, log on to https://www.editorialmanager.com/pone/ and select the 'Submissions Needing Revision' folder to locate your manuscript file.

Please include the following items when submitting your revised manuscript:

- A rebuttal letter that responds to each point raised by the academic editor and reviewer(s). You should upload this letter as a separate file labeled 'Response to Reviewers'.
- A marked-up copy of your manuscript that highlights changes made to the original version. You should upload this as a separate file labeled 'Revised Manuscript with Track Changes'.
- An unmarked version of your revised paper without tracked changes. You should upload this as a separate file labeled 'Manuscript'.

If you would like to make changes to your financial disclosure, please include your updated statement in your cover letter. Guidelines for resubmitting your figure files are available below the reviewer comments at the end of this letter.

If applicable, we recommend that you deposit your laboratory protocols in protocols.io to enhance the reproducibility of your results. Protocols.io assigns your protocol its own identifier (DOI) so that it can be cited independently in the future. For instructions see: <https://journals.plos.org/plosone/s/submission-guidelines#loc-laboratory-protocols>. Additionally, PLOS ONE offers an option for publishing peer-reviewed Lab Protocol articles, which describe protocols hosted on protocols.io. Read more information on sharing protocols at <https://plos.org/protocols?utm_medium=editorial-email&utm_source=authorletters&utm_campaign=protocols>.

We look forward to receiving your revised manuscript.

Kind regards,

Bi-Song Yue, Ph.D
Academic Editor
PLOS ONE

Journal requirements:

When submitting your revision, we need you to address these additional requirements.

1. Please ensure that your manuscript meets PLOS ONE's style requirements, including those for file naming. The PLOS ONE style templates can be found at

https://journals.plos.org/plosone/s/file?id=wjVg/PLOSOne_formatting_sample_main_body.pdf and

<https://journals.plos.org/plosone/s/file?id=ba62/PLOSOne_formatting_sample_title_authors_affiliations.pdf>

2. In your Data Availability statement, you have not specified where the minimal data set underlying the results described in your manuscript can be found. PLOS defines a study's minimal data set as the underlying data used to reach the conclusions drawn in the manuscript and any additional data required to replicate the reported study findings in their entirety. All PLOS journals require that the minimal data set be made fully available. For more information about our data policy, please see http://journals.plos.org/plosone/s/data-availability.

Upon re-submitting your revised manuscript, please upload your study’s minimal underlying data set as either Supporting Information files or to a stable, public repository and include the relevant URLs, DOIs, or accession numbers within your revised cover letter. For a list of acceptable repositories, please see http://journals.plos.org/plosone/s/data-availability#loc-recommended-repositories. Any potentially identifying patient information must be fully anonymized.

Important: If there are ethical or legal restrictions to sharing your data publicly, please explain these restrictions in detail. Please see our guidelines for more information on what we consider unacceptable restrictions to publicly sharing data: http://journals.plos.org/plosone/s/data-availability#loc-unacceptable-data-access-restrictions. Note that it is not acceptable for the authors to be the sole named individuals responsible for ensuring data access.

We will update your Data Availability statement to reflect the information you provide in your cover letter.

[Note: HTML markup is below. Please do not edit.]

Reviewers' comments:

Reviewer's Responses to Questions

**Comments to the Author**

1. Is the manuscript technically sound, and do the data support the conclusions?

The manuscript must describe a technically sound piece of scientific research with data that supports the conclusions. Experiments must have been conducted rigorously, with appropriate controls, replication, and sample sizes. The conclusions must be drawn appropriately based on the data presented.

Reviewer #1: Partly

Reviewer #2: Partly

2. Has the statistical analysis been performed appropriately and rigorously?

Reviewer #1: N/A

Reviewer #2: Yes

3. Have the authors made all data underlying the findings in their manuscript fully available?

The [PLOS Data policy](http://www.plosone.org/static/policies.action#sharing) requires authors to make all data underlying the findings described in their manuscript fully available without restriction, with rare exception (please refer to the Data Availability Statement in the manuscript PDF file). The data should be provided as part of the manuscript or its supporting information, or deposited to a public repository. For example, in addition to summary statistics, the data points behind means, medians and variance measures should be available. If there are restrictions on publicly sharing data—e.g. participant privacy or use of data from a third party—those must be specified.

Reviewer #1: Yes

Reviewer #2: Yes

4. Is the manuscript presented in an intelligible fashion and written in standard English?

PLOS ONE does not copyedit accepted manuscripts, so the language in submitted articles must be clear, correct, and unambiguous. Any typographical or grammatical errors should be corrected at revision, so please note any specific errors here.

Reviewer #1: No

Reviewer #2: No

5. Review Comments to the Author

Please use the space provided to explain your answers to the questions above. You may also include additional comments for the author, including concerns about dual publication, research ethics, or publication ethics. (Please upload your review as an attachment if it exceeds 20,000 characters)

Reviewer #1: 1. The current work cannot fully support "ATP6 was significantly correlated with a mean diurnal temperature range" and "Ka/Ks ratio of COX2 was strongly associated with several precipitation-related variables". The authors are required to provide further analysis or sufficient evidence to support these views.

Answer: We have revised the manuscript and adopted a euphemism.
2. The authors declared that their work provides a foundation for integrating the mitochondrial genome into future investigations of how these species may respond to ongoing global climatic changes. I don’t understand how did they conclude that?

Answer: We have revised this sentence to “Based on this, we believe that our study provides a new insight into the role of the mitochondrial genome of *Acrossocheilus* species in adaptation to different environments.”
3. The author selected 25 species with the closest relationship to construct a phylogenetic tree, just for selection analysis? When the author performed phylogenetic analysis and Ka/Ks analysis, it is necessary to expand the research scope of species, and compare the differences between different taxa in combination with the traits of species. Obviously, this research is not enough.

Answer: We have revised the manuscript. We included 25 published mitochondrial genome sequences from 14 species in NCBI. The remaining species, although reported, are difficult to sample and accurately identify due to their rarity and existence of synonyms with species that included in this study (*A. rendahli* may as a synonym for *A. yunnanensis*) [12]. Besides, there are some species that may in fact belong to other genera, such as, *A.malacopterus* may belong to the genus *Onychostoma* and *A. ikedai* should probably belong to the genus *Poropuntius* [12]. Therefore, only 25 published mitochondrial genome sequences from 14 species in this genus were included in this study. Moreover, in another study, we are trying to address the taxonomic confusion of species not included in this study using the method of molecular delimitation of species.

[12]. Yuan LY. Taxonomic Revision of Chinese Species of the Cyprinid Genus Acrossocheilus (Teleostei: Cypriniformes). M.Sc. Thesis, Nanchang University. 2005. Available from: https://kns.cnki.net/kcms/detail/detail.aspx?dbcode=CMFD&dbname=CMFD0506&filename=2006022941.nh&uniplatform=NZKPT&v=SzhvHT2d3k6d13FM3lvMO5bpIg77SwIPRiq0vxjXRGcmjD6JnQ8_-LnSzQBqH_Ci
4. Lines (78-82), please provide a bibliography or citation source.

Answer: We have added citations and references to the manuscript (doi: 10.1186/1471-2164-9-119; doi: 10.1111/mec.12240; doi: 10.1111/jzs.12079).
5. Many researches have focused on the environmental adaptation of organisms. A previously published mitochondrial study (DOI: https://doi.org/10.1186/s12862-021-01803-y) can provide a reference for this study.

Answer: Thank you for your reference.
6. It is recommended that the results of the selection analysis should be presented graphically.

Answer: We have displayed the results of the selection analysis in a graph, named Fig1.
7. Line 307, "<<" should be "<".

Answer: We have revised the manuscript.
8. Lines (309-311) “The differing mutation rates (μRelative) that we detected across different genes may result from differences in the strength of purifying selection due to functional constraints”. Is there sufficient evidence to support this conclusion? If yes, please list it. If not, please delete it.

Answer: We have added citations and references to the manuscript (doi: 10.1016/j.ympev.2015.11.008; doi: 10.1016/j.tig.2007.03.008; doi: 10.1093/oxfordjournals.molbev.a004014).

Reviewer #2: This is fine study, and some data is valuable. However, I am not quite sure that the current study fits to the journal scope and standard because this is very general study which is not something new.

1. “This genus has 26 species, which have a center of diversity (21 species) in southern China and commonly occur in middle and/or lower reaches of river drainage”. But in this paper, the authors only chose 25 published sequences of 14 species to analyze. It is better to add more new sequenced species to confirming the findings.
Answer: We have revised the manuscript. We included 25 published mitochondrial genome sequences from 14 species in NCBI. The remaining species, although reported, are difficult to sample and accurately identify due to their rarity and existence of synonyms with species that included in this study (*A. rendahli* may as a synonym for *A. yunnanensis*) [12]. Besides, there are some species that may in fact belong to other genera, such as, *A.malacopterus* may belong to the genus *Onychostoma* and *A. ikedai* should probably belong to the genus *Poropuntius* [12]. Therefore, only 25 published mitochondrial genome sequences from 14 species in this genus were included in this study. Moreover, in another study, we are trying to address the taxonomic confusion of species not included in this study using the method of molecular delimitation of species.

[12]. Yuan LY. Taxonomic Revision of Chinese Species of the Cyprinid Genus Acrossocheilus (Teleostei: Cypriniformes). M.Sc. Thesis, Nanchang University. 2005. Available from: https://kns.cnki.net/kcms/detail/detail.aspx?dbcode=CMFD&dbname=CMFD0506&filename=2006022941.nh&uniplatform=NZKPT&v=SzhvHT2d3k6d13FM3lvMO5bpIg77SwIPRiq0vxjXRGcmjD6JnQ8_-LnSzQBqH_Ci.

2. In this paper, although the phylogenetic tree with high bootstrap, if we add other species, the topology will change, and the authors did not describe the taxonomy of the genus in the introduction.

Answer: Our phylogenetic analyses produced identical topologies with previous studies [38]. Moreover, we have added the distribution of this genus in different temperature zones in the introduction.

[38]. Yuan LY, Liu XX, Zhang E. Mitochondrial Phylogeny of Chinese Barred Species of the Cyprinid Genus Acrossocheilus Oshima, 1919 (Teleostei: Cypriniformes) and Its Taxonomic Implications. Zootaxa. 2015; 4059: 151–168. https://doi.org/10.11646/zootaxa.4059.1.8.

6. PLOS authors have the option to publish the peer review history of their article ([what does this mean?](https://journals.plos.org/plosone/s/editorial-and-peer-review-process#loc-peer-review-history)). If published, this will include your full peer review and any attached files.


If you choose “no”, your identity will remain anonymous but your review may still be made public.


**Do you want your identity to be public for this peer review?** For information about this choice, including consent withdrawal, please see our [Privacy Policy](https://www.plos.org/privacy-policy).

Reviewer #1: No

Reviewer #2: No

[NOTE: If reviewer comments were submitted as an attachment file, they will be attached to this email and accessible via the submission site. Please log into your account, locate the manuscript record, and check for the action link "View Attachments". If this link does not appear, there are no attachment files.]

While revising your submission, please upload your figure files to the Preflight Analysis and Conversion Engine (PACE) digital diagnostic tool, <https://pacev2.apexcovantage.com/>. PACE helps ensure that figures meet PLOS requirements. To use PACE, you must first register as a user. Registration is free. Then, login and navigate to the UPLOAD tab, where you will find detailed instructions on how to use the tool. If you encounter any issues or have any questions when using PACE, please email PLOS at [figures@plos.org](mailto:figures@plos.org). Please note that Supporting Information files do not need this step.

*In compliance with data protection regulations, you may request that we remove your personal registration details at any time.* [*(Remove my information/details)*](https://www.editorialmanager.com/pone/login.asp?a=r)*. Please contact the publication office if you have any questions.*
